# Childhood vaccines and antibiotic use in low- and middle-income countries

**DOI:** 10.1038/s41586-020-2238-4

**Published:** 2020-04-29

**Authors:** Joseph A. Lewnard, Nathan C. Lo, Nimalan Arinaminpathy, Isabel Frost, Ramanan Laxminarayan

**Affiliations:** 1 Division of Epidemiology, School of Public Health, University of California, Berkeley, Berkeley, CA USA; 2 Division of Infectious Diseases and Vaccinology, School of Public Health, University of California, Berkeley, Berkeley, CA USA; 3 Center for Computational Biology, College of Engineering, University of California, Berkeley, Berkeley, CA USA; 4 Department of Medicine, University of California, San Francisco, San Francisco, CA USA; 5 School of Public Health, Imperial College London, London, UK; 6 Center for Disease Dynamics, Economics & Policy, New Delhi, India; 7 Princeton Environmental Institute, Princeton University, Princeton, NJ USA

**Keywords:** Bacterial infection, Viral infection, Epidemiology

## Abstract

Vaccines may reduce the burden of antimicrobial resistance, in part by preventing infections for which treatment often includes the use of antibiotics^1–4^. However, the effects of vaccination on antibiotic consumption remain poorly understood—especially in low- and middle-income countries (LMICs), where the burden of antimicrobial resistance is greatest^5^. Here we show that vaccines that have recently been implemented in the World Health Organization’s Expanded Programme on Immunization reduce antibiotic consumption substantially among children under five years of age in LMICs. By analysing data from large-scale studies of households, we estimate that pneumococcal conjugate vaccines and live attenuated rotavirus vaccines confer 19.7% (95% confidence interval, 3.4–43.4%) and 11.4% (4.0–18.6%) protection against antibiotic-treated episodes of acute respiratory infection and diarrhoea, respectively, in age groups that experience the greatest disease burden attributable to the vaccine-targeted pathogens^6,7^. Under current coverage levels, pneumococcal and rotavirus vaccines prevent 23.8 million and 13.6 million episodes of antibiotic-treated illness, respectively, among children under five years of age in LMICs each year. Direct protection resulting from the achievement of universal coverage targets for these vaccines could prevent an additional 40.0 million episodes of antibiotic-treated illness. This evidence supports the prioritization of vaccines within the global strategy to combat antimicrobial resistance^8^.

## Abstract (web summary)

Pneumococcal and rotavirus vaccines have reduced antibiotic consumption substantially among children under five years old in low- and middle-income countries; however, this effect could be doubled if all countries were to implement vaccination programmes and meet universal vaccine coverage targets.

## Main

Antimicrobial resistance (AMR) poses a substantial and growing threat to global health and economic well-being^9^. Populations of LMICs suffer the greatest morbidity and mortality associated with AMR, and stand to be disproportionately affected by projected increases in this burden over future decades^10^. Antibiotic use is a key risk factor for human colonization or infection with drug-resistant organisms at scales spanning individuals, communities and countries^11^. Increasing rates of antibiotic consumption in LMICs^12^ have highlighted tension between the need to ensure access to life-saving antibiotics globally, and the need to curb resistance selection^13^. Although it has been widely hypothesized that vaccines may reduce AMR burden by preventing infections that drive antibiotic consumption^1^, few real-world studies provide evidence of this effect^14^.

Acute respiratory infection (ARI) and diarrhoea are the leading causes of antibiotic use among children in LMICs^15^. Because few diagnostic tools exist to guide management of ARI and diarrhoea, antibiotic treatment of these conditions is generally informed by suspicion rather than confirmation of disease aetiology. Pneumococcal conjugate vaccines (PCVs) against 10 and 13 serotypes of *Streptococcus pneumoniae* and live attenuated rotavirus vaccines target the predominant causes of ARI and diarrhoea among children, respectively^6,16^. These vaccines have recently been introduced into routine paediatric immunization programmes of countries across the globe^17^. Although appropriate for children experiencing pneumococcal ARI, antibiotic treatment is not warranted for rotavirus-attributable diarrhoea. To date, no studies have addressed the impact of PCV10/13 and rotavirus vaccines on antibiotic use in LMICs^5^.

We analysed data from demographic and health surveys to estimate: (i) the incidence of antibiotic treatment for ARI and diarrhoea among children in LMICs; (ii) the effectiveness of PCV10/13 and rotavirus vaccines in preventing antibiotic use associated with ARI and diarrhoea among children in these settings; and (iii) the proportion of antibiotic-treated ARI and diarrhoea cases attributable to vaccine-serotype *S. pneumoniae* and rotavirus, respectively. We used these results to quantify the incidence of antibiotic consumption among children in LMICs prevented by current uses of PCV10/13 and rotavirus vaccines, and the additional consumption that could be averted by achieving universal vaccine coverage targets^18^.

## Vaccine effects against antibiotic use

We first assessed whether PCV10/13 and rotavirus vaccines are effective in preventing antibiotic-treated illnesses among children in LMICs. We undertook a case–control study comparing the odds of previous vaccination among children under five years of age reported to have experienced (cases) or not to have experienced (controls) ARI and diarrhoea in the two weeks preceding the standardized household surveys. End points for our analysis were (i) all cases of disease, (ii) cases for which medical care or advice was sought, and (iii) cases receiving antibiotic treatment. Data were available on 65,815 children from surveys conducted from 2015 onward in Afghanistan, Angola, Armenia, Burundi, Ethiopia, Haiti, Lao People’s Democratic Republic, Malawi, Nepal, Pakistan, the Philippines, Senegal, Sierra Leone, South Africa, Tajikistan, the United Republic of Tanzania, Uganda and Zimbabwe (Supplementary Tables 1, 2; earlier surveys did not collect children’s history of PVC10/13 and rotavirus vaccination). To eliminate confounding effects driven by any association between the access of children to vaccines and the likelihood of disease or antibiotic treatment, we matched cases and asymptomatic controls from the same countries for age and survey date (each within one month), urbanicity, within-country wealth quintile and receipt of pentavalent vaccine doses (a potential confounder of ARI analyses, and a marker of vaccine and/or healthcare access). We also conducted planned subgroup analyses for children aged 0–23 months and aged 24–59 months due to well-documented differences in ARI and diarrhoea aetiology across age groups^6,7^.

**Fig. 1:**
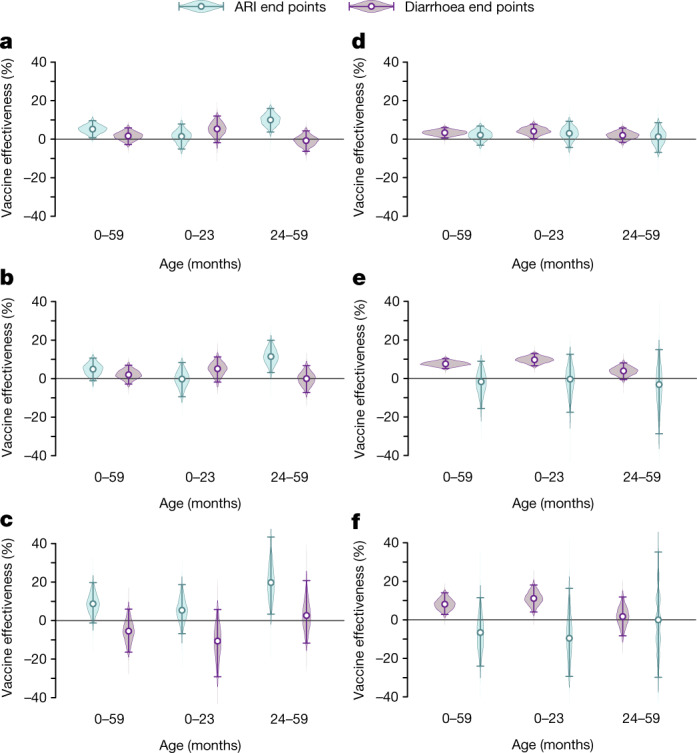
Effectiveness of pneumococcal and rotavirus vaccines against illness and antibiotic treatment. **a**–**f**, The estimated effectiveness against ARI and diarrhoea end points of PCV10/13 (**a**–**c**) and rotavirus vaccines (**d**–**f**) for all cases (**a**, **d**), cases for which treatment or advice was sought (**b**, **e**) and cases that were treated with antibiotics (**c**, **f**). Estimates were calculated as one minus the matched odds ratio and are shown as vaccine effectiveness. Analyses matched children with each end point to asymptomatic controls on the basis of country, age (within 1 month), visit timing (within 1 month), wealth quintile (country-specific), urbanicity and pentavalent vaccine doses received. The population available for analysis included 5,342 ARI cases (of whom 3,294 sought treatment or advice and 1,913 received antibiotics) and 57,856 controls without ARI; and 9,944 diarrhoea cases (of whom 7,382 sought treatment or advice and 1,437 received antibiotics) and 40,059 controls without diarrhoea (Supplementary Tables 1, 2). Points and lines indicate median estimates and 95% confidence intervals, respectively. We estimated vaccine effectiveness against negative-control end points (PCV10/13 effect against diarrhoea; rotavirus vaccine effect against ARI) to assess residual confounding as a validation step. PCV10/13 exposure was defined as ≥3 doses received. Because all countries in this analysis used Rotarix in their national immunization program, we defined ≥2 doses as a full rotavirus vaccination series. Numerical estimates can be found in Supplementary Tables 3–5. Quantiles are estimated through 2,000 independent draws from the distribution of estimates.

Children who received at least three PCV10/13 doses experienced 8.7% lower odds of antibiotic-treated ARI than unvaccinated children (Fig. 1 and Supplementary Table 3), although the 95% confidence interval for this estimate included the possibility of no effect (–1.3–19.7% reduction (values in parentheses indicate 95% confidence intervals throughout); two-sided *P* = 0.079). The estimated reduction was driven primarily by the prevention of cases in children aged 24–59 months; within this age group, vaccination was associated with a 19.7% (3.4–43.4%) reduction in cases of antibiotic-treated ARI. By contrast, effects of PCV10/13 were not clearly evident during the first 2 years of life, when less than 5% of cases of ARI are attributable to vaccine-serotype pneumococci^7^. To test that our findings are the result of vaccine-conferred protection and not confounding factors, we also estimated PCV10/13 effectiveness against diarrhoea and antibiotic-treated diarrhoea. Although these outcomes would not be expected to be causally related to PCV10/13 receipt, their association with previous vaccination may be prone to similar confounding pathways. We did not find strong evidence of associations between negative-control outcomes and receipt of PCV10/13, validating our finding that PCV10/13-conferred protection reduces the incidence of antibiotic-treated ARI.

Similarly, children who received a full rotavirus vaccine series (at least two doses for countries included in this study) experienced 8.1% (2.8–14.1%) lower odds of antibiotic-treated diarrhoea at ages of 0–59 months than their unvaccinated counterparts (Fig. 1 and Supplementary Tables 4, 5). During the first 2 years of life, we estimated 11.4% (4.0–18.6%) effectiveness of rotavirus vaccination against antibiotic-treated diarrhoea. Protection was not evident in children aged 24–59 months, when rotavirus is a less common cause of diarrhoea among children in LMICs^6^. The absence of associations between rotavirus vaccination and negative-control ARI end points provided validation for our finding that vaccine-conferred protection against rotavirus reduces the risk to children of antibiotic-treated diarrhoea.

## Analysing aetiological fractions using vaccine effects

We next developed a simple model using the vaccine effectiveness estimates described above to investigate the proportions of antibiotic-treated ARI and diarrhoea attributable to vaccine-serotype pneumococci and rotavirus, respectively (Methods). The model took these inputs together with pooled estimates of vaccine efficacy against disease caused by the targeted pathogens based on meta-analyses of previous studies (Supplementary Table 7). Because rotavirus vaccine efficacy beyond the second year of life is uncertain (and likely to be low)^19,20^, and because we did not identify strong evidence of vaccine-conferred protection against antibiotic-treated diarrhoea in children aged 24–59 months, we limited our assessment of rotavirus-attributable antibiotic use to children aged 0–23 months.

**Fig. 2:**
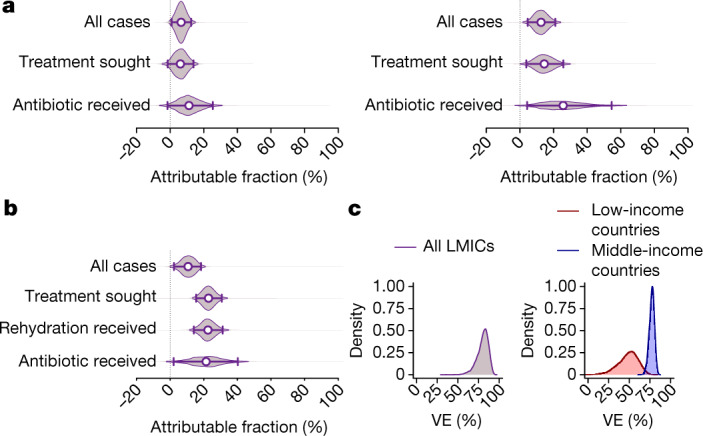
Estimates of the attributable fraction for vaccine-preventable infections. **a**, **b**, We illustrate estimates of pathogen-specific attributable fractions of vaccine-serotype pneumococci for children aged 0–59 months (**a**, left) and 24–59 months (**a**, right), and rotavirus for children aged 0–23 months (**b**). **c**, We also illustrate distributions of vaccine efficacy estimates against infections involving the vaccine-targeted organism, as estimated using meta-analyses (Supplementary Table 7); these estimates provided a basis for computing the attributable fraction (Supplementary Tables 1, 2). We considered PCV efficacy against vaccine-serotype invasive pneumococcal disease for the primary analysis (left). A secondary analysis (Supplementary Table 8) used PCV efficacy against culture-confirmed pneumococcal vaccine-serotype acute otitis media. For rotavirus (right), we stratified estimates of human monovalent rotavirus vaccine (Rotarix) efficacy against rotavirus gastroenteritis in children aged 0–23 months in middle-income countries and low-income countries to account for the differential efficacy in these settings, and obtained a pooled estimate of the attributable fraction weighted by the number of children residing in middle-income countries and low-income countries (Methods). VE, vaccine effectiveness. Points and lines indicate median estimates and 95% confidence intervals, respectively. Quantiles are obtained through 2,000 independent draws from the distribution of estimates.

We estimated that vaccine-serotype pneumococci would account for 10.9% (–1.6–25.4%) of antibiotic-treated ARI cases among children under five years old in PCV10/13-naive LMIC populations (Fig. 2 and Supplementary Table 8); the negative lower limit of this confidence interval resulted from propagation of uncertainty in the estimated effect of PCV10/13 against antibiotic-treated ARI in children aged 0–59 months, and reflects the possibility that vaccine-targeted pneumococcal serotypes are not a significant cause of antibiotic consumption. Among children aged 24–59 months, we estimated that vaccine-serotype pneumococci cause 24.8% (4.3–54.6%) of antibiotic-treated ARI cases.

We similarly estimated that rotavirus would cause 21.6% (2.1–40.3%) of antibiotic-treated diarrhoea among children aged 0–23 months in unvaccinated LMIC populations. Consistent with the findings of diarrhoea aetiology studies undertaken across differing LMICs^6,21^, we obtained similar estimates of the rotavirus-attributable fraction in analyses stratified by the income status of each country, in which we accounted for variation in rotavirus vaccine efficacy across settings (Methods and Supplementary Tables 7, 8). We estimated that 21.3% (0.0–42.4%) and 22.7% (4.5–60.6%) of antibiotic-treated diarrhoea cases would be attributable to rotavirus among unvaccinated children in middle-income and low-income countries, respectively.

## Antibiotic use in LMICs

To understand these estimates of vaccine effectiveness and pathogen-attributable fractions in terms of absolute antibiotic consumption, we estimated the incidence of ARI-related antibiotic use among children aged 24–59 months, and of diarrhoea-related antibiotic use among children aged 0–23 months. We analysed data from household surveys covering 944,173 children across 77 countries from 2006 to 2018 (Methods). We used the resulting estimates to extrapolate country-level incidence rates for 58 additional countries without recent household surveys, aided by data on 405 national-level health, socioeconomic and demographic economic indicators from the World Bank Open Data catalogue (https://data.worldbank.org/) (Supplementary Tables 9, 10).

**Fig. 3:**
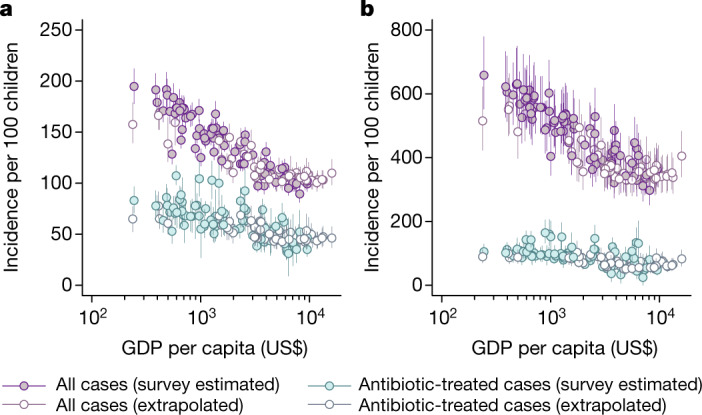
Estimated incidence across countries of ARI and diarrheal illnesses per 100 children. **a**, **b**, We estimate country-specific incidence of ARI and antibiotic-treated ARI in children aged 24–59 months (**a**) and diarrhoea and antibiotic-treated diarrhoea in children aged 0–23 months (**b**). Points and lines indicate median estimates for an individual country together with accompanying 95% confidence intervals. Estimates are obtained from analyses of Demographic Health Surveys and Multiple Indicator Cluster Surveys, comprising 944,173 children across 77 countries, as well as extrapolations based on 405 health, nutrition and population indicators for all LMICs. Points plotted in white are extrapolated from estimates based on household survey data (see Methods). Estimates for individual countries are provided in Supplementary Tables 11–13. Quantiles are obtained through 5,000 independent draws from the distribution of estimates.

Estimated incidence rates of all cases and antibiotic-treated cases of ARI and diarrhoea were the highest in the poorest countries, and the lowest in the wealthiest countries (Fig. 3). Our estimates of country-specific ARI incidence in children aged 24–59 months ranged from 89.5 to 194.8 episodes per 100 children annually across all LMICs (Fig. 3 and Supplementary Table 11; we present further estimates of antibiotic-treated ARI in children aged 0–59 months in Supplementary Table 12). For children aged 24–59 months, the estimated proportion of episodes treated with antibiotics ranged from 30.0% to 69.4%, yielding incidence rates of antibiotic-treated ARI of between 31.2 and 107.3 cases per 100 children annually. For diarrhoea in children aged 0–23 months, we estimated that country-specific incidence rates ranged from 297.5 to 658.4 episodes per 100 children annually. Accounting for antibiotic treatment in an estimated 7.7–37.7% of diarrhoea episodes, annual rates of antibiotic-treated diarrhoea ranged from 24.6 to 164.5 episodes per 100 children. Country-specific estimates are included in Supplementary Tables 11–19.

## Pathogen-attributable antibiotic use

**Fig. 4:**
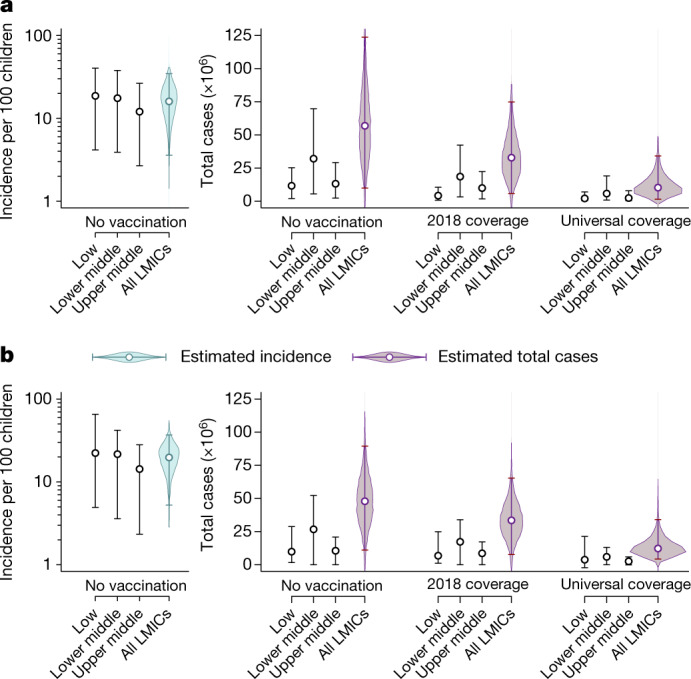
Total vaccine-preventable antibiotic consumption and incidence per 100 children. **a**, **b**, We estimated the incidence and total number of antibiotic-treated ARI and diarrhoea episodes attributable to PCV10/13-serotype pneumococci in children aged 24–59 months (**a**) and rotavirus in children aged 0–23 months (**b**), respectively. Left, incidence in the absence of vaccination. Right, the corresponding total number of cases, the total number of cases under 2018 vaccine coverage levels and under universal vaccine coverage. Estimates were stratified by income status (low income; lower middle income; upper middle income). Points indicate median estimates, with superimposed lines indicating 95% confidence intervals; violin plots illustrate the distribution around estimated incidence and total cases. Numerical estimates are provided in Supplementary Tables 20–22, 24–26. Quantiles are obtained through 5,000 independent draws from the distribution of estimates.

Multiplying these incidence rate estimates by pathogen-specific attributable fractions provides the burden of antibiotic-treated ARI and diarrhoea targeted by PCV10/13 and rotavirus vaccines. In the absence of vaccination, we estimated that vaccine-serotype pneumococci would cause 15.9 (2.8–34.7) antibiotic-treated ARI episodes per 100 children aged 24–59 months annually in LMICs, translating to a total of 56.9 (9.9–123.8) million antibiotic-treated ARI episodes (Fig. 4 and Supplementary Table 20). We estimated that 11.7 (2.0–24.8) million episodes occur annually in countries in which PCV10/13 has not been implemented. Analyses comprising all children aged 0–59 months residing in LMICs yielded similar burden estimates (Supplementary Table 21).

We estimated that rotavirus would cause 19.7 (4.6–36.8) antibiotic-treated diarrhoea episodes annually per 100 children aged 0–23 months in LMICs in the absence of vaccination, resulting in a total of 47.9 (11.1–89.5) million antibiotic-treated diarrhoea episodes (Fig. 4 and Supplementary Table 22). Of this burden, we estimated that 20.4 (3.4–38.7) million episodes occur annually in settings without rotavirus vaccination.

## Vaccine-preventable antibiotic use

The ‘direct’ protection conferred by the immunological effects of vaccines on their recipients provides a lower-bound estimate of the impact achievable by immunization programmes, as it excludes ‘indirect’ protection resulting from reduced transmission of the vaccine-targeted pathogens^22^. As of 2018, an estimated 66.8% of age-eligible children in countries with PCV programmes in place received a full series of at least three PCV10/13 doses^23^ (Supplementary Table 23). Under these coverage levels, we estimate that direct effects of PCV10/13 prevent 23.8 (4.2–52.0) million episodes of antibiotic-treated ARI annually among children aged 24–59 months in LMICs (Fig. 4 and Supplementary Tables 24, 25). This effect represents 42.4% (32.7–47.5%) of all antibiotic-treated ARI episodes that vaccine-serotype pneumococci would be expected to cause among children aged 24–59 months in LMICs, in the absence of PCV10/13 use. We estimated that an additional 21.7 (3.8–47.5) million episodes of antibiotic-treated ARI could be prevented by the direct effects of vaccination if PCV10/13 coverage were expanded to reach all children aged 24–59 months in LMICs. This residual burden corresponds to 38.7% (30.0–43.4%) of all antibiotic-treated ARI episodes that we estimated to be attributable to vaccine-serotype pneumococci among children aged 24–59 months in LMICs.

Roughly 77.3% of age-eligible children in LMICs in which rotavirus vaccination has been implemented received a full rotavirus vaccine series as of 2018^23^. We estimated that direct effects of rotavirus vaccination currently prevent 13.6 (3.6–23.7) million episodes of antibiotic-treated diarrhoea annually among children aged 0–23 months in LMICs (Supplementary Table 26). This effect reflects only 31.0% (17.7–35.2%) of all antibiotic-treated diarrhoea episodes that we would expect rotavirus to cause annually among children aged 0–23 months who live in LMICs, in the absence of vaccination. We estimated that an additional 18.3 (4.2–32.6) million episodes of antibiotic-treated diarrhoea could be prevented annually by the direct effects of rotavirus vaccination among children aged 0–23 months under a scenario of universal vaccine coverage, representing 42.1% (14.6% to 50.7%) of all antibiotic use that we estimated to be attributable to rotavirus in this population.

## Discussion

We found that vaccines against *S. pneumoniae* and rotavirus reduce antibiotic consumption among children in LMICs. In the absence of these vaccines, we estimated that PCV10/13-serotype pneumococci and rotavirus would cause 56.9 million and 47.9 million episodes of antibiotic-treated ARI and diarrhoea, respectively, among the age groups at greatest risk for these infections. Current PCV10/13 and rotavirus immunization programmes prevent an estimated 37.4 million episodes of antibiotic-treated illnesses annually through direct vaccine-conferred protection among children under five years of age in LMICs. Within this population, an additional 40.0 million episodes can be prevented through improvements in vaccine coverage in settings in which PCV10/13 and rotavirus vaccines are currently implemented, and through the introduction of these vaccines in countries in which children currently do not receive them.

It is helpful to consider the effects that we estimate alongside those of other interventions aiming to reduce unnecessary antibiotic consumption. Infrastructural improvements that address water, sanitation and hygiene have been prioritized in LMIC action plans to combat AMR^8,9^. However, randomized trials reporting suboptimal effects of such interventions on disease incidence^24^ suggest that these approaches may present limited opportunities to prevent antibiotic-treated illness. Although certain antimicrobial stewardship interventions are feasible in LMICs^25^, the need to tailor such interventions to country-specific contexts hinders the development, adoption and assessment of universal strategies^26^. Recent aetiological studies reveal that most antibiotic courses administered to cases of ARI and diarrhoea among children in LMICs are unnecessary or inappropriate^7,27^, but few diagnostic tools exist to inform the clinical management of these infections. This shortage of alternative approaches to optimize antibiotic treatment in LMICs underscores the potential value of vaccination as a component of strategies to reduce AMR.

We used the recent introduction of PCV10/13 and rotavirus vaccines into paediatric immunization programmes as a natural means to estimate antibiotic use attributable to the vaccine-targeted pathogens. Strengths of our study include the opportunity to estimate the direct effects of the vaccines and disease burden using individual-level data from consistently designed surveys; statistical power for the estimation of small effect sizes in our case–control study of more than 60,000 children across 16 countries; and our ability to validate vaccine effectiveness estimates against negative-control end points. However, certain limitations should be considered. First, we relied on mother-reported disease outcomes and antibiotic use rather than a standardized clinical assessment. Although an imperfect data source^28^, maternal reports have been validated for the measurement of acute illness, care-seeking and antibiotic receipt among children in LMICs in previous studies^15,29–31^. Risk factor data in household surveys provided an opportunity to correct for potential differences in reporting thresholds for these conditions across settings—a limitation of previous burden studies based on survey data^32,33^. Second, our vaccine-analysis method permitted us to estimate pathogen-attributable antibiotic use only within the strata for which we had high-quality estimates of vaccine efficacy, as well as evidence of effect of the vaccines in our case–control study. For this reason, our findings centre on the age groups at greatest risk for pneumococcal ARI and rotavirus diarrhoea, among all children under five years of age who live in LMICs^6,7^. Finally, our focus on the direct effects of the vaccines, which we were able to estimate through a case–control study, may lead to underestimation of the population-level impact of vaccination programmes. Indirect effects of PCV10/13 and rotavirus vaccines have also been reported in LMICs in which these vaccines have been implemented in routine paediatric immunization programmes^34–37^.

**Extended Data Fig. 1:**
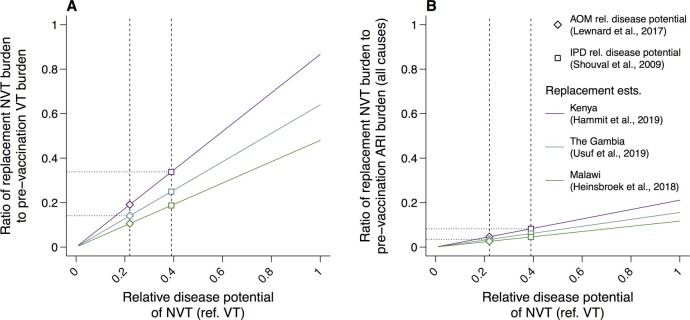
Potential effect of pneumococcal serotype replacement. **a**, We compare the maximum estimate of the replacement disease associated with increased carriage of nonvaccine-type (NVT) pneumococci to the prevented burden of disease associated with vaccine-type (VT) pneumococci based on the approach described in the Methods section ‘Potential reduction in PCV10/13 effects owing to serotype replacement’. We plot the ratio of replacement-attributable NVT disease as a function of the relative pathogenicity of NVT and VT pneumococci. As this ratio may differ according to disease end point, the measure presented in **a** can be interpreted as end point agnostic. Estimates from two previous studies^64, 65^ of acute otitis media (AOM) and invasive pneumococcal disease (IPD) provide a range of 0.218–0.387 for the relative pathogenicity of NVT compared with VT pneumococci in these conditions. We plot estimates based on serotype replacement observations from three carriage studies in LMICs with continuous, prospective surveillance in place before and after PCV10/13 implementation^35, 66, 70^. **b**, We next illustrate the estimated ratio of replacement-attributable antibiotic-treated ARI to all-cause antibiotic-treated ARI among children aged 24–59 months, based on our estimates of the fraction of antibiotic-treated ARI attributable to VT pneumococci (Fig. 2). On the basis of the input values from the carriage and disease studies cited^35, 64–66, 70^, we infer that the maximum extent of antibiotic-treated ARI that replacement serotypes would account for would decrease to between 2.6% and 8.2% of the pre-vaccination incidence of all-cause antibiotic-treated ARI; this range is well below the estimated reduction associated with protection against VT pneumococci.

Several further considerations inform the interpretation of the effects of PCV10/13 on antibiotic consumption. Acute otitis media, sinusitis and other upper respiratory infections that are potentially attributable to pneumococci account for a substantial share of antibiotic use among infants within high-income settings^38^. These conditions may not be captured with high sensitivity by the ARI definition used in household surveys^39,40^. As a result, our use of an ARI end point is likely to underestimate the antibiotic consumption that is preventable by PCV10/13. By contrast, replacement of vaccine-targeted pneumococcal serotypes by non-vaccine serotypes may reduce the long-term effect of PCV10/13 on various pneumococcal disease end points^41^. However, the association of vaccine-targeted serotypes with conditions such as pneumonia and otitis media^42,43^, which drive antibiotic consumption, suggests a limited capacity of replacement serotypes to offset the effects of PCV10/13 on this consumption (Extended Data Fig. 1). In addition, the reported extent of vaccine-driven serotype replacement in pneumococcal carriage and disease has remained lower in LMICs^35,44,45^ than in high-income settings, for reasons that remain incompletely understood^46^.

Emerging recognition of the threat posed by AMR has led to global, regional and national action plans to address the increasing burden of resistant disease^8,47–49^. A lack of evidence on the effectiveness of strategies advocated in these plans—which span public education efforts, human and veterinary antimicrobial stewardship, sanitation and other interventions—constrains the ability of policymakers to allocate resources to approaches with the greatest effect. Our study demonstrates that PCV10/13 and rotavirus vaccines reduce antibiotic consumption among children in LMICs, and that a substantial volume of antibiotic consumption may be prevented by introducing and increasing the global coverage of these vaccines. This evidence supports the prioritization of childhood vaccines as part of a global strategy to combat AMR.

## Methods

### Household survey data

We used data from Demographic Health Surveys (DHS; rounds V–VII; https://www.dhsprogram.com) and Multiple Indicator Cluster Surveys (MICS; rounds 5, 6; http://mics.unicef.org/surveys) undertaken in various LMICs from 2006 to 2018. Design of the two surveys was coordinated to facilitate data comparison and joint analyses across countries and over time. In brief, a two-stage probability sampling approach was used to select geographical clusters within countries or provinces of countries, and to select households within clusters. Survey questionnaires addressed household composition as well as risk factors, health outcomes and healthcare utilization among household occupants; original survey instruments can be viewed at https://dhsprogram.com/publications/publication-DHSG4-DHS-Questionnaires-and-Manuals.cfm (for DHS data) and https://mics.unicef.org/surveys (for MICS data).

Mothers were asked to answer survey questions on behalf of children under five years of age. Health information collected about children included history of ARI symptoms (defined as short, rapid breathing or difficult breathing that was chest-related or occurring with cough) and diarrhoea (defined as the occurrence of three loose stools in a 24-h period) in the 2 weeks preceding the survey. Mothers were asked about care seeking at public-sector or private-sector hospitals or clinics, pharmacies or doctor’s offices, and receipt of drugs including antibiotic pills, syrups or injections, antimalarials, and analgesics or antipyretics. For diarrhoea, mothers were also asked about children’s receipt of antimotility drugs, intravenous feeding, zinc and oral rehydration. Vaccination status (including antigens, doses and date of receipt) was collected from vaccination cards. Round VII of DHS and round 6 of MICS were the first to include PCV10/13 and rotavirus vaccination; thus, the case–control study (detailed below) included data from DHS round VII and MICS round 6 only.

### Case–control study

We estimated PCV10/13 effectiveness against ARI-related end points and rotavirus vaccine effectiveness against diarrhoea-related end points in an individually matched, case–control study to compare counterfactual outcomes among individuals encountering similar conditions of transmission, as well as other exposures (the matching procedure is described below). End points were: occurrence of ARI and diarrhoea symptoms; occurrence of ARI and diarrhoea symptoms for which medical treatment or advice was sought (from a source other than a traditional practitioner); and occurrence of ARI and diarrhoea symptoms for which antibiotic pills, syrups or injections were received. For diarrhoea analyses, we also considered an end point of diarrhoea symptoms resulting in oral rehydration using pre-packaged salts or fluid, or recommended homemade fluids or intravenous solution, among children who sought care (results are included in Supplementary Tables 4, 5). For the purposes of our analyses, children experiencing each end point in the preceding 2 weeks were defined as ‘cases’, and those who did not report symptoms were defined as controls.

Within each country, we matched cases and controls on the basis of age and visit timing (each within one month), within-country wealth quintile (a variable defined on the basis of country-specific household socioeconomic indicators), urbanicity (residence within a sampling cluster defined as urban or rural) and pentavalent vaccine doses received (to reduce confounding by healthcare access, including access to vaccination, and to control for any effects of diphtheria, pertussis and *Haemophilus influenzae* type b antigens on ARI). Healthcare utilization questions for the ARI end point did not distinguish whether care was received due to respiratory symptoms or due to fever. Therefore, to separate vaccine effects from antibiotic treatment associated with ARI symptoms (rather than treatment of fever cases that would be expected to occur independent of ARI symptoms, for example, due to malaria, typhoid or arboviruses), we also matched children on mother-reported fever and excluded those receiving antimalarials.

We matched cases and controls without replacement at a 1:3 ratio and undertook statistical inference in a resampling framework, generating 2,000 independent draws from the permutation distribution of case–control match assignments. Within each iteration, we estimated the matched odds ratio (OR_M_) using conditional logistic regression. Defining *Y* = 1 and *Y* = 0 as case and control status, respectively, and *V =* 1 and *V =* 0 as vaccinated and unvaccinated status, respectively, we took 1 − OR_M_(V|Y) to measure vaccine effectiveness.

### Assessment of residual confounding

The relatively recent implementation of PCV10/13 and rotavirus vaccines in the immunization programmes of countries may make pentavalent vaccine an imperfect surrogate of the access of children to these newer products. In particular, within-country geographical differences in access to PCV10/13 and rotavirus vaccines may introduce residual confounding if the differences in vaccine access are associated with geographical variation in disease burden.

Data from DHS and MICS include the subnational administrative regions in which children reside, although this variable must be interpreted with caution as the size and uniformity of subnational regions varies across countries (between 4 and 3,399 children were available from each region (median, 217 children), before accounting for match eligibility). Although it was not possible to match children at precise subnational resolutions for our case–control study, we used subnational administrative region designators to assess risk for residual confounding by geography. We computed the proportion of variance in ARI and diarrhoea outcomes, and in PCV10/13 and rotavirus vaccine receipt, explained by subnational region after accounting for all other matching factors included in our analysis. Specifically, we assessed the difference in the summed squared error of residuals from regression models treating disease end points and vaccination as outcome variables, which either included or did not include subnational administrative units as intercepts after controlling for age and visit timing (each as monthly factor variables), urbanicity, wealth quintile, mother-reported fever (only for analyses of ARI and PCV10/13) and pentavalent vaccine doses received. Low estimates of the proportion of variance explained by subnational region in either the exposure or outcome suggest minimal risk of confounding by geography. The results of this analysis are included in Supplementary Table 6. In addition, to assess whether negative-control analyses provided a sufficient opportunity to correct for residual confounding associated with access to these newer vaccines, we estimated the correlation in receipt of full PCV10/13 and rotavirus vaccine series among age-eligible children in countries where both vaccines were recommended. We describe the results of these analyses in the Supplementary Information section ‘Assessment of residual confounding’.

### Vaccine probe study

We formulated a simple model that enables the estimation of the proportion of episodes attributable to vaccine-targeted pathogens for each end point (for example, ARI symptoms or antibiotic-treated ARI symptoms) in the absence of vaccination. We defined *ρ* as the risk for a child to experience a given end point attributable to the vaccine-targeted pathogen in the 2-week recall period, *ω* as the risk of the same end point attributable to all other causes and *θ* as the relative risk of disease caused by the vaccine-targeted pathogen in a vaccinated individual relative to an unvaccinated individual, owing only to vaccine-conferred protection (such that 1 − *θ* measures vaccine efficacy against illness caused by the targeted pathogen). Under this framework

1 $${{\rm{OR}}}_{{\rm{M}}}(V|Y)=\frac{\Pr (V=1,Y=1){\rm{\Pr }}(V=0,Y=0)}{\Pr (V=0,Y=1){\rm{\Pr }}(V=1,Y=0)}=\frac{[\theta \rho +\omega ][1-\rho -\omega ]}{[1-\theta \rho -\omega ][p+\omega ]}=\frac{\theta \rho +\omega }{\rho +\omega }\times \frac{1-\rho -\omega }{1-\theta \rho -\omega }$$

Noting that $\frac{1-\rho -\omega }{1-\theta \rho -\omega }\approx 1$ for small *ρ* and *ω*

2 $${{\rm{O}}{\rm{R}}}_{{\rm{M}}}(V|Y)\approx \frac{\theta \rho +\omega }{\rho +\omega }$$

or in other words, we expected the matched odds ratio to provide a reasonable estimator for the relative hazard of each end point, given vaccination. Using equation (2), above

3a $$\rho \approx \frac{\omega (1-{{\rm{O}}{\rm{R}}}_{{\rm{M}}}(V|Y))}{{{\rm{O}}{\rm{R}}}_{{\rm{M}}}(V|Y)-\theta }$$

and

3b $$\frac{\rho }{\rho +\omega }\approx \frac{1-{{\rm{O}}{\rm{R}}}_{{\rm{M}}}(V|Y)}{1-\theta }$$

**Extended Data Fig. 2:**
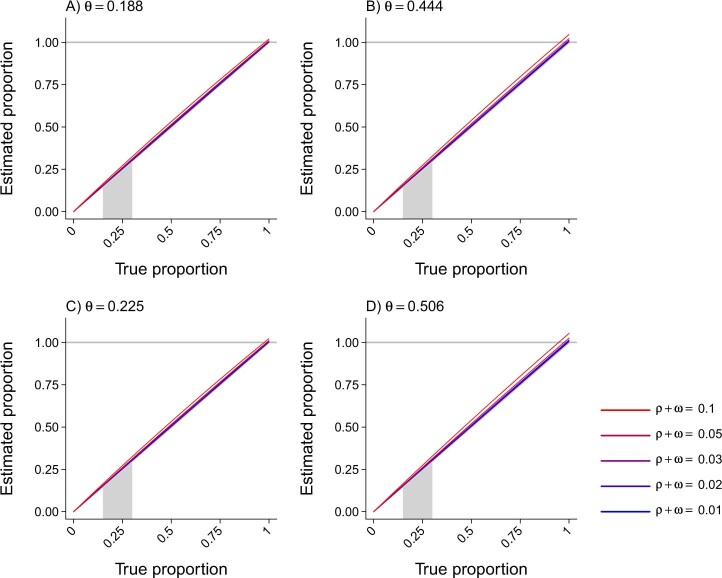
Bias that occurs when using the odds ratio to approximate the relative risk. **a**–**d**, We illustrate the degree of bias that occurs in attributable fraction estimates (indicated by the departure of the estimated proportion from the 1:1 diagonal) under differing parameterizations with respect to the true aetiological fraction and vaccine efficacy against the targeted infection. Values correspond to our meta-analytic estimates of PCV efficacy against vaccine-serotype invasive pneumococcal disease (**a**), PCV efficacy against vaccine-serotype acute otitis media (**b**), rotavirus vaccine efficacy against rotavirus gastroenteritis in middle-income countries (**c**) and rotavirus vaccine efficacy against rotavirus gastroenteritis in low-income countries (**d**). The range of 15–30% is highlighted in grey as plausible values for the proportion of disease attributable to vaccine-serotype pneumococci for children aged 24–59 months and rotavirus for children aged 0–23 months, based on previously published studies^6, 7^. Values are plotted on a blue-to-red ramp corresponding to increases in symptom prevalence (*ρ* + *ω*); values of 0.01, 0.02, 0.03, 0.05 and 0.1 correspond to incidence rates of 52, 104, 156, 261 and 521 episodes per 100 children annually (roughly the range of our all-cause ARI and diarrhoea incidence rate estimates); under the assumption of a 3-day duration of symptoms, the same prevalence values correspond to incidence rates of 122 to 1,217 episodes per 100 children annually. These outcomes suggest negligible bias in aetiological fractions over the range of plausible values for our analysis.

The above approximation relies on the assumption that OR_M_(*V*|*Y*) is similar to the relative risk of disease, given vaccination. We illustrate in Extended Data Fig. 2 the potential degree of bias under differing values of *θ*, *ρ* and *ω* that are concordant with our results and those of external studies assessing all-cause burden of ARI and diarrhoea among children in LMICs^15,50,51^. The degree of bias approaches zero under any of the following conditions: (i) the all-cause disease end point is rare (that is, *ρ* + *ω* ≈ 0); (ii) the attributable fraction is low (*ρ* ≪ *ω*); or (iii) the vaccine is highly efficacious against disease due to the pathogen of interest (*θ* ≈ 0).

### Estimation of the fraction attributable to rotavirus

Because previous studies have reported differential efficacy and effectiveness of rotavirus vaccines in socioeconomically distinct settings, we generated stratified estimates of OR_M_(*V*|*Y*) (in the case–control study) and *θ* (in the meta-analysis, as described below) for children aged 0–23 months residing in middle-income and low-income countries. We used these inputs to generate separate rotavirus-attributable fraction estimates associated with each end point for middle-income and low-income countries. We reconstructed the distribution of the rotavirus-attributable fraction for each end point across all LMIC settings (Fig. 2 and Supplementary Table 8) using the weighted average of paired draws from the independent distributions of the attributable fraction estimates of middle-income and low-income countries; weights were proportional to the total populations of children aged 0–23 months residing in middle-income and low-income countries.

To account for differential direct effects of the vaccine against rotavirus-attributable diarrhoea end points in middle-income and low-income countries, we used our stratified estimates of rotavirus vaccine efficacy, effectiveness and attributable fractions for all burden assessments.

### Meta-analysis of vaccine efficacy studies

We obtained estimates of *θ* for PCV10/13 and rotavirus vaccine using a meta-analysis of vaccine efficacy studies. Because *θ* has not been estimated for the effects of PCV10/13 against vaccine-serotype ARI, we took PCV efficacy against invasive disease caused by vaccine-serotype pneumococci as a primary estimate of protection, and PCV efficacy against acute otitis media caused by vaccine-serotype pneumococci as a lower-bound estimate, consistent with previous studies^52^. Estimates of PCV efficacy against these end points were aggregated from two previously published reviews^53,54^. We used estimates of efficacy against invasive pneumococcal disease from only low- and middle-income countries. Because such estimates were not available for PCV efficacy against acute otitis media, we did not restrict estimates for this end point on the basis of study setting.

For rotavirus, we used estimates of *θ* from studies that estimated the efficacy of the human pentavalent rotavirus vaccine (Rotarix) against rotavirus gastroenteritis in the first two years of life in LMICs, again aggregated in a previous systematic review^55^. Data were restricted to Rotarix studies because this was the only vaccine used in the national immunization programmes of countries included in the case–control study.

For both end points, we estimated pooled values of *θ* using inverse variance-weighted random effect models using log-transformed effect size estimates from each study.

### Risk factor analysis of household survey data

**Extended Data Fig. 3:**
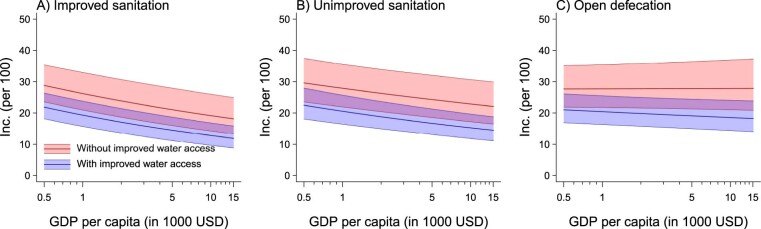
Fitted association of improved water and sanitation access with diarrhoea risk. **a**–**c**, We illustrate model-estimated rates of the incidence (Inc.) per 100 children of diarrhoea under differing conditions of access to improved water and sanitation conditions among at ages 0–23 months. Estimates account for interactions of sanitation and water access with GDP per capita. Shaded regions indicate 95% confidence intervals around estimates. True intercepts are dependent on the distribution of other individual and setting-level risk factors; plotted estimates do not account for the joint distribution of other risk factors with respect to the access of children to water and sanitation at the country level and GDP per capita. Sanitation conditions include improved (**a**) and unimproved sanitation (**b**) or open defaecation (**c**) in combination with or without improved water access. Model parameter estimates are presented in full in Supplementary Table 30. Estimates are obtained from analyses of DHS and MICS surveys comprising 377,665 children across 77 countries. Quantiles are obtained through 5,000 independent draws from the distribution of estimates.

We used a regression-based approach to estimate country-level rates of incidence of ARI and diarrhoea in the year 2016 in the absence of PCV10/13 and rotavirus vaccine use. Because previous studies have described marked variation in the clinical threshold for maternal or caregiver report of ARI and diarrhoea^28,32,33,56^, we aimed to reconstruct incidence rates on the basis of risk factor prevalence, controlling for region-level intercepts that arise from differential reporting of symptoms. We built Poisson regression models for the outcome of reporting of syndrome *Z* (signifying all-cause ARI or diarrhoea) for child *i* of the form

4 $${\rm{E}}({Z}_{i}|{X}_{i})={\pi }_{{\rm{R}}(i)}\times \exp \,(\alpha +\sum _{k}{\beta }_{k}{X}_{k,i}+{{\epsilon }}_{i})$$

where *α* represents a general intercept for the log-transformed incidence rate. Each variable *X*_*k*,*i*_ indicates the exposure of child *i* to the *k*th risk factor for syndrome *Z*, associated with a *β*_*k*_-fold increase in incidence; the ${{\epsilon }}_{i}$ errors are independent and identically distributed with mean zero among all children. The term *π*_R(*i*)_ indicates the probability of reporting syndrome *Z* given it truly occurred among children in the same region as child *i*. We extracted the risk factors listed in Supplementary Table 27 on the basis of their inclusion in both DHS and MICS datasets, and based on previous evidence of their relevance to the risk of ARI and/or diarrhoea for children^51,57,58^. In addition to variables included in DHS and MICS, we extracted the gross domestic product (GDP) per capita of each country in the survey year and in 2016. We included covariates in the model identified to have a statistically significant (*P* < 0.05) unadjusted association with the outcome variable that persisted in multivariable analyses, and tested for significant pairwise interactions among covariates. Risk factor estimates are shown in Extended Data Fig. 3 and Supplementary Tables 28–30 for ARI (for children aged 24–59 months and 0–59 months) and diarrhoea (for children aged 0–23 months).

We similarly modelled the probability (*H*) for ARI and diarrhoea to be treated with antibiotics using a Poisson regression model of the form

5 $${\rm{E}}({H}_{i}|{X}_{i})=\exp \,(\delta +\sum _{k}{\delta }_{k}{X}_{k,i}+{\eta }_{i})$$

**Extended Data Fig. 4:**
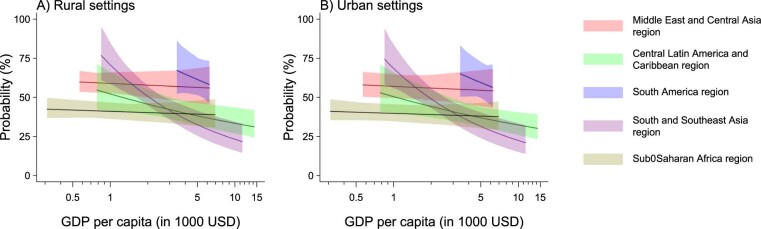
Fitted association of GDP per capita with antibiotic treatment of ARI, by region. **a**, **b**, We illustrate model-estimated probabilities of antibiotic treatment for cases of ARI among children aged 24–59 months in rural (**a**) and urban (**b**) settings associated with region and GDP per capita. Estimates for central and eastern Europe are omitted from plots as wide uncertainty intervals would obscure the illustration of associations in other settings. Shaded regions indicate 95% confidence intervals around the estimates. True intercepts are dependent on the distribution of other individual and setting-level risk factors; plotted estimates do not account for the joint distribution of other risk factors with respect to region and GDP per capita. Model parameters are included in Supplementary Table 31; estimates are nearly identical for children aged 24–59 months and 0–59 months (Supplementary Table 32). Estimates are obtained from analyses of DHS and MICS surveys comprising 566,508 children across 77 countries. Quantiles are obtained through 5,000 independent draws from the distribution of estimates.

**Extended Data Fig. 5:**
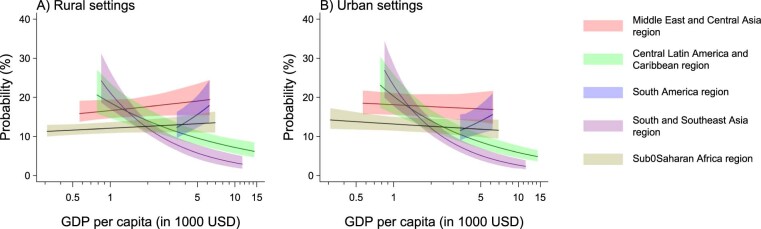
Fitted association of GDP per capita with antibiotic treatment of diarrhoea, by region. **a**, **b**, We illustrate model-estimated probabilities of antibiotic treatment for cases of diarrhoea among children aged 0–23 months in rural (**a**) and urban (**b**) settings associated with region and GDP per capita. Estimates for central and eastern Europe are omitted from plots as wide uncertainty intervals would obscure the illustration of associations in other settings. Shaded regions indicate 95% confidence intervals around estimates. True intercepts are dependent on the distribution of other individual and setting-level risk factors; plotted estimates do not account for the joint distribution of other risk factors with respect to region and GDP per capita. Model parameter estimates are provided in Supplementary Table 33. Estimates are obtained from analyses of DHS and MICS surveys comprising 377,665 children across 77 countries. Quantiles are obtained through 5,000 independent draws from the distribution of estimates.

We evaluated the same risk factors for inclusion as in our analyses of ARI and diarrhoea; here we defined *η*_*i*_ as mean-zero independent and identically distributed error terms. The individual risk factor estimates are shown in Extended Data Figs. 4, 5 and Supplementary Tables 31–33 for antibiotic-treated ARI and diarrhoea.

We limited analyses of ARI and diarrhoea end points to children who had received no PCV10/13 doses and no rotavirus vaccine doses, respectively. For DHS rounds V–VI and MICS round 5, which did not include PCV10/13 and rotavirus vaccine data collection, children were assumed not to have received PCV10/13 or rotavirus vaccine doses if countries had not yet implemented these vaccines in their immunization programmes at the time of the survey. Surveys undertaken in countries that had implemented PCV10/13 or rotavirus vaccines were excluded if information on the receipt of these vaccines among children was not collected.

Missing values in the outcome and risk factor variables were populated in five independent pseudo-datasets by multiple imputation using the Amelia II package in R^59^; in total, 11.4% of data cells were missing at the outset of analyses, including 0.6% and 2.4% of ARI and antibiotic-treated ARI observations, and 0.1% and 0.4% of diarrhoea and antibiotic-treated diarrhoea observations. Additional measures^12^ of country-level antibiotic access (Supplementary Table 27) were included due to scientific interest and a consideration that these covariates may aid imputation.

### Assumption of a Poisson outcome distribution

Translating the reported history of any ARI or diarrhoea in the preceding 2 weeks to a Poisson-distributed number of ARI or diarrhoea episodes required two assumptions: that episodes are acute (consistent with the nature of illness solicited by DHS and MICS household surveys) and that overall incidence rates are low (resulting in a low or negligible likelihood of ≥2 distinct episodes occurring in a single fortnightly period in which a child was reported to have experienced ARI or diarrhoea)^30^. The low frequency of diarrhoea lasting 14 days or longer in a previous cohort study in LMICs^21^ (accounting for 4.9% and 1.8% of all diarrhoea episodes in the first and second years of life, respectively) indicated that our assumption of an acute nature of mother-reported illness was appropriate for the diarrhoea outcome. Similarly, previous studies of respiratory infections among children in LMICs have reported short average durations of new-onset disease episodes, with 87% to 100% of episodes lasting ≤2 weeks^60–62^. For incidence rates similar to those estimated in our study (approximately 4 diarrhoea cases and 1.25 ARI cases per child per year), assuming a 7-day average duration of illness and a definition of 3 symptom-free days to distinguish separate disease episodes, the assumption of exponentially distributed inter-event times (concordant with Poisson-distributed counts) would yield a second new-onset diarrhoea case in only 4.3% of 2-week reporting periods with a first case identified, and a second new-onset ARI case in only 1.4% of reporting periods with a first case identified. This small bias, if applicable to our analyses, would be expected to lead to slight underestimation of true burden.

### Incidence rate estimation

From the fitted models, we generated standardized estimates of the hazard rates of ARI and diarrhoea for individual children for the year 2016. To correct for differential reporting, we took the lowest and highest estimated reporting probability from any region (*π*_R_) to provide lower and upper bounds, respectively, on true incidence rates.

We sampled from the multivariate normal distribution of (log-transformed) regression parameter estimates and model residuals, thus accounting for uncertainty in the effects of risk factors on event rates. To propagate uncertainty in our analyses driven by sampling variability in the surveyed population as well as our multiple imputation procedure, we repeated this analysis 1,000 times for each of 5 multiply imputed datasets. At each iteration, we resampled children according to survey weights before sampling ARI and diarrhoea outcomes stochastically based on individual-level risk factors. On the basis of the estimated incidence rate of ARI and diarrhoea of each child from these risk factors, we sampled a total number of annual cases assuming this value would be Poisson-distributed with respect to the underlying individual-specific rate. We used the same approach to sample the antibiotic treatment status of each case based on model-generated estimates of the probability of antibiotic treatment, given individual risk factors, under an assumed Bernoulli outcome distribution.

### Extrapolation of burden estimates to countries without household survey data

**Extended Data Fig. 6:**
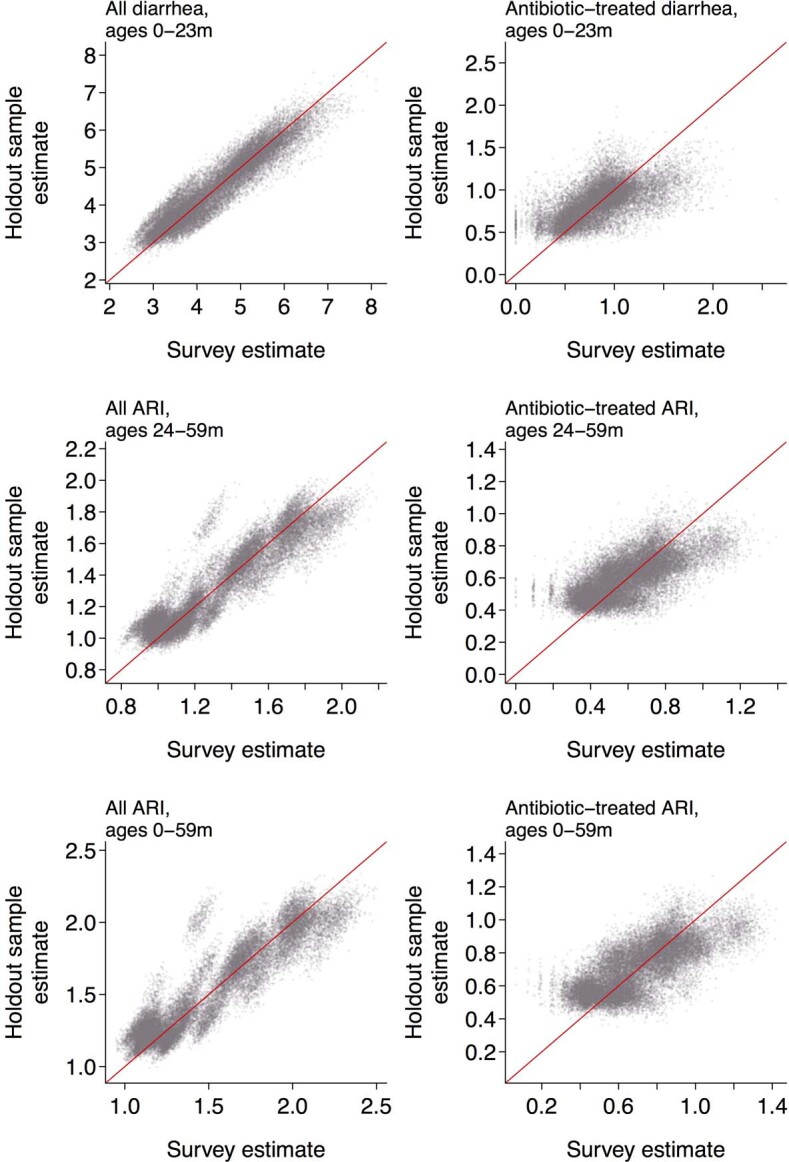
Out-of-sample performance of the extrapolated incidence estimation. We illustrate the extrapolated estimates of ARI and diarrhoea incidence and antibiotic-treated ARI and antibiotic-treated diarrhoea incidence against model-based estimates within the 10% holdout samples reserved to assess out-of-sample performance of predictive modelling. Red lines indicate expectations under the scenario of one-to-one correspondence between true and predicted out-of-sample values.

For the 58 LMICs not covered by DHS rounds V–VII or MICS rounds 5 and 6, we extrapolated rates of incidence of all cases and antibiotic-treated cases of ARI and diarrhoea on the basis of national-level variables aggregated as Health, Nutrition and Population Statistics by the World Bank (https://databank.worldbank.org/source/health-nutrition-and-population-statistics). For each of 5,000 draws from the distribution of country-specific incidence-rate and treatment probability estimates (1,000 draws each from 5 multiply imputed datasets), we fitted regression tree models using tenfold cross-validation to a randomly sampled 90% set of countries via stochastic gradient boosting using the caret package in R with default tuning parameters^63^. We saved predictions for the 10% ‘holdout’ set of countries to assess model performance (Extended Data Fig. 6). We resampled the 90/10 set of training and holdout countries at each iteration. We used fitted models to generate out-of-sample predictions for countries without DHS and MICS data.

### Estimating the vaccine-preventable disease burden

We multiplied independent draws from the distribution of our estimates of the proportion of antibiotic-treated ARI episodes and antibiotic-treated diarrhoea episodes attributable to vaccine-serotype pneumococci and rotavirus, respectively, by independent draws from the distribution of our estimates of country-level incidence rates for antibiotic-treated ARI (for children aged 24–59 months and 0–59 months) and antibiotic-treated diarrhoea (for children aged 0–23 months). We used our stratified estimates of rotavirus-attributable fractions (for low-income and middle-income countries) for all analyses of diarrhoea burden end points. For analyses estimating vaccine direct effects, we multiplied independent draws from the distribution of our estimates of PCV10/13 direct effects against antibiotic-treated ARI and rotavirus vaccine direct effects against antibiotic-treated diarrhoea, as obtained in the case–control study, by independent draws from the distribution of our estimates of country-level incidence rates for antibiotic-treated ARI (of children aged 24–59 months and 0–59 months) and antibiotic-treated diarrhoea (in children aged 0–23 months).

To generate estimates of burden across multiple countries (globally, or grouped by income strata), we estimated total cases in each country by multiplying independent draws of the incidence rate for each end point by the number of children in each age group (using World Bank estimates; https://databank.worldbank.org/home). For each sampled parameterization (defined by estimates of vaccine effectiveness, pathogen-attributable burden and incidence rates), we summed total cases across countries belonging to a stratum of interest and estimated incidence by dividing total summed cases by the population at risk.

### Potential reduction in PCV10/13 effects owing to serotype replacement

Replacement of vaccine-targeted pneumococcal serotypes by nonvaccine serotypes may partially offset the estimated effect of PCV10/13 on all-cause ARI end points. We assessed the extent to which this may alter our estimates by determining the maximal increase in nonvaccine-type antibiotic-treated ARI that could be expected under scenarios consistent with reported serotype replacement in pneumococcal carriage in LMIC settings.

We defined *p*_VT_(*t*) and *p*_NVT_(*t*) as the prevalence of carriage of vaccine-type (VT) pneumococci and nonvaccine-type (NVT) pneumococci at time *t*, and *r*_VT_ and *r*_NVT_ as the rates of progression of vaccine-type and nonvaccine-type pneumococci from carriage to ARI^42^. We used previous estimates of the relative incidence of vaccine-type and nonvaccine-type invasive pneumococcal disease per carrier^64^, and of vaccine-type and nonvaccine-type otitis media per carrier^65^, to supply bounds on *r*_VT_ and *r*_NVT_ for ARI, as this quantity has not previously been measured. We used data from three studies of pneumococcal carriage among children less than five years old before and after vaccine introduction in settings with long-term, continuous, prospective surveillance in place^7,44,66^ to supply pre-vaccination (*p*_VT_(0) and *p*_NVT_(0)) and post-vaccination (*p*_VT_(1) and *p*_NVT_(1)) estimates of carriage prevalence. We took [*p*_NVT_(1) − *p*_NVT_(0)]*r*_NVT_ to indicate the excess incidence of disease attributable to nonvaccine serotypes that would result from post-vaccination replacement of vaccine-targeted serotypes by nonvaccine serotypes.

We compared this excess post-vaccination incidence attributable to serotype replacement with two measures of pre-vaccination disease incidence. The ratio [*p*_NVT_(1) − *p*_NVT_(0)]*r*_NVT_/[*p*_VT_(0)*r*_VT_] compared the excess replacement-associated incidence of nonvaccine-serotype disease to the incidence of vaccine-serotype disease targetable by vaccination with PCV10/13. Second, defining AF as the fraction of all-cause disease incidence attributable to vaccine-type pneumococci, the ratio [*p*_NVT_(1) − *p*_NVT_(0)]*r*_NVT_AF/[*p*_VT_(0)*r*_VT_] compared the extent of replacement-driven nonvaccine-serotype disease incidence to all-cause incidence in the pre-vaccination era. These estimates are shown in Extended Data Fig. 1, using the attributable fraction estimates for all-cause antibiotic-treated ARI (as this was the primary end point of interest in our analyses).

In light of the assumptions informing our interpretation of PCV10/13 effectiveness against non-specific ARI end points in the case–control study, we consider the estimates provided by this analysis to represent an upper bound on the potential replacement-driven disease burden. Our estimates of vaccine-serotype pneumococcal attributable fractions based on the case–control study assumed no effect of PCV10/13 on disease not attributable to vaccine-serotype pneumococci. However, children receiving PCV10/13 in prelicensure or early-implementation studies in fact have been reported to experience higher risk of carriage of nonvaccine serotypes^67^ and resulting nonvaccine-type disease^68,69^. As such, odds ratio estimates in our case–control study may be interpretable as representing the ‘net’ reduction in disease risk among vaccinated children, accounting for both protection against vaccine-type pneumococci and increased acquisition of nonvaccine types.

### Reporting summary

Further information on research design is available in the Nature Research Reporting Summary linked to this paper.

## Online content

Any methods, additional references, Nature Research reporting summaries, source data, extended data, supplementary information, acknowledgements, peer review information; details of author contributions and competing interests; and statements of data and code availability are available at 10.1038/s41586-020-2238-4.

## Supplementary information

Supplementary Information This file contains Supplementary Text S1-S3 and Supplementary Table 1-33.

Reporting Summary

## Data Availability

MICS data are publicly available at http://mics.unicef.org/. DHS data are available upon request from https://dhsprogram.com/Data/. Files posted in the GitHub repository (http://github.com/joelewnard) include the reduced versions of the DHS and MICS datasets necessary to replicate analyses.

## Code availability

The analysis code necessary to replicate all parts of the analysis and for the generation of the figures is available from GitHub (http://github.com/joelewnard).
