# Supplementary material for: Childhood vaccines and antibiotic use in low- and middle-income countries
Source: Nature. 2020 Apr 29;581(7806):94–9. doi: 10.1038/s41586-020-2238-4 (PMC7332418; doi:10.1038/s41586-020-2238-4)
Supplement: Supplementary file 1 — This file contains Supplementary Text S1-S3 and Supplementary Table 1-33. [file 41586_2020_2238_MOESM1_ESM.pdf]

nature

https://doi.org/10.1038/s41586-020-2238-4

Supplementary information

Childhood vaccines and antibiotic use in low- and middle-income countries

In the format provided by the authors and unedited

Joseph A. Lewnard✉, Nathan C. Lo, Nimalan Arinaminpathy, Isabel Frost & Ramanan Laxminarayan

SUPPLEMENTARY INFORMATION

Childhood vaccines and antibiotic use in low and middle income countries

Joseph A. Lewnard[1,2,3,*], Nathan C. Lo[4], Nimalan Arinaminpathy[5], Isabel Frost[5,6], Ramanan Laxminarayan[6,7]

Affiliations:

1. Division of Epidemiology, School of Public Health, University of California, Berkeley, Berkeley, California
2. Division of Infectious Diseases & Vaccinology, School of Public Health, University of California, Berkeley, Berkeley, California
3. Center for Computational Biology, College of Engineering, University of California, Berkeley, Berkeley, California
4. Department of Medicine, University of California, San Francisco, San Francisco, California
5. School of Public Health, Imperial College, London, London, UK
6. Center for Disease Dynamics, Economics & Policy, New Delhi, India
7. Princeton Environmental Institute, Princeton University, Princeton, New Jersey, USA

* Correspondence to: jlewnard@berkeley.edu

Contents

Supplemental text

Text S1.	Assessment of residual confounding in the case-control study.	4
Text S2.	Regression analysis results.	4
Text S3.	Supplemental references.	5

Supplemental tables

Table S1.	Characteristics of children eligible for inclusion in case-control studies.	7
Table S2.	Sample sizes for case-control studies.	8
Table S3.	Effectiveness of ≥3 PCV10/13 doses against various endpoints.	9
Table S4.	Effectiveness of ≥2 rotavirus vaccine doses against various endpoints.	10
Table S5.	Effectiveness of ≥2 rotavirus vaccine doses against diarrhea and antibiotic-treated diarrhea among children ages 0-23 months in low- and middle-income countries.	11
Table S6.	Variance explained by subnational administrative groupings in the case-control study.	12
Table S7.	Meta-analysis of vaccine efficacy studies involving pathogen-specific endpoints.	13
Table S8.	Attributable fraction estimates.	14
Table S9.	Countries included in analyses.	15
Table S10.	World Bank indicator variables included in cross-validation analyses.	18
Table S11.	Country-specific estimates of the incidence of ARI and antibiotic-treated ARI among children ages 24-59 months preventable by 10- and 13-valent pneumococcal conjugate vaccines.	27
Table S12.	Country-specific estimates of the incidence of ARI and antibiotic-treated ARI among children ages 0-59 months preventable by 10- and 13-valent pneumococcal conjugate vaccines.	31
Table S13.	Country-specific estimates of the incidence of diarrhea and antibiotic-treated diarrhea among children ages 0-23 months preventable by rotavirus vaccination.	35
Table S14.	Upper-bound country-specific estimates of the incidence of ARI and antibiotic-treated ARI among children ages 24-59 months preventable by 10- and 13-valent pneumococcal conjugate vaccines.	39
Table S15.	Lower-bound country-specific estimates of the incidence of ARI and antibiotic-treated ARI among children ages 24-59 months preventable by 10- and 13-valent pneumococcal conjugate vaccines.	43
Table S16.	Upper-bound country-specific estimates of the incidence of ARI and antibiotic-treated ARI among children ages 0-59 months preventable by 10- and 13-valent pneumococcal conjugate vaccines.	47
Table S17.	Lower-bound country-specific estimates of the incidence of ARI and antibiotic-treated ARI among children ages 0-59 months preventable by 10- and 13-valent pneumococcal conjugate vaccines.	52
Table S18.	Upper-bound country-specific estimates of the incidence of diarrhea and antibiotic-treated diarrhea among children ages 0-23 months preventable by rotavirus vaccination.	56
Table S19.	Lower-bound country-specific estimates of the incidence of diarrhea and antibiotic-treated diarrhea among children ages 0-23 months preventable by rotavirus vaccination.	60

Table S20.	Total antibiotic consumption attributable to vaccine-serotype *Streptococcus pneumoniae* among children ages 24-59 months in LMICs, absent use of PCV10/13.	64
Table S21.	Total antibiotic consumption attributable to vaccine-serotype *Streptococcus pneumoniae* among children ages 0-59 months in LMICs, absent use of PCV10/13.	65
Table S22.	Total antibiotic consumption attributable to rotavirus among children ages 0-23 months in LMICs, absent use of rotavirus vaccines.	66
Table S23.	Country-level PCV10/13 and rotavirus vaccine coverage estimates.	67
Table S24.	Total antibiotic consumption preventable by 10- and 13-valent pneumococcal conjugate vaccines among children ages 24-59 months in LMICs.	70
Table S25.	Total antibiotic consumption preventable by 10- and 13-valent pneumococcal conjugate vaccines among children ages 0-59 months in LMICs.	71
Table S26.	Total antibiotic consumption preventable by rotavirus vaccination among children ages 0-23 months in LMICs.	72
Table S27.	Information extracted for estimation of ARI and diarrhea incidence.	73
Table S28.	Fitted model parameters for ARI incidence among children ages 24-59 months.	74
Table S29.	Fitted model parameters for ARI incidence among children ages 0-59 months.	75
Table S30.	Fitted model parameters for diarrhea incidence among children ages 0-23 months.	76
Table S31.	Fitted model parameters for antibiotic treatment of ARI among children ages 24-59 months.	77
Table S32.	Fitted model parameters for antibiotic treatment of ARI among children ages 0-59 months	78
Table S33.	Fitted model parameters for antibiotic treatment of diarrhea among children ages 0-23 months.	79

Text S1: Assessment of residual confounding in the case-control study.

We present in **Table S6** estimates of the proportion of residual variation in ARI and diarrhea outcomes, and in PCV10/13 and rotavirus vaccine receipt, accounted for by sub-national administrative region variables. After controlling for factors included in the matching analysis, we estimated that <2% of residual variation in each ARI endpoint (all ARI cases, ARI cases for which treatment or advice was sought, and antibiotic-treated ARI) is accounted for by sub-national region; we obtained similar estimates (<2% of residual variation explained) for all diarrhea endpoints (all diarrhea cases, diarrhea cases for which treatment/advice was sought, diarrhea cases receiving rehydration, and antibiotic-treated diarrhea cases). Of residual variation in vaccine receipt not accounted for by the matching factors, we estimated 8.9% of variation in PCV10/13 receipt and 2.1% of variation in rotavirus vaccine receipt is accounted for by sub-national region.

These findings should be interpreted cautiously given the likelihood of between-country differences in the size and uniformity of administrative regions. However, the low degree of unexplained variation in outcome and exposure variables suggests that geographic differences in disease burden and access to PCV10/13 and rotavirus vaccines, at least at the level of administrative regions, are unlikely to present an important source of residual confounding in the case-control study.

We also computed an 80% correlation coefficient between receipt of a full PCV10/13 series and receipt of a full rotavirus vaccine series among age-eligible children residing in countries where both vaccines were recommended. The high estimated correlation suggests similar causal pathways underlie receipt of each of these vaccines. This finding strengthens our ability to interpret any association between rotavirus vaccination and ARI endpoints, or between PCV10/13 vaccination and diarrhea endpoints, as a measure of residual confounding between vaccine access and disease endpoints not accounted for by the matching procedure. In light of the strong association between receipt of PCV10/13 and rotavirus vaccine, the null outcomes of our analyses addressing negative-control endpoints support the causal validity of our primary estimates of PCV10/13 effectiveness against ARI endpoints, and of rotavirus vaccine effectiveness against diarrhea endpoints.

Text S2: Regression analysis results.

To standardize our estimation of ARI and diarrhea burden, which may have been reported subjectively by mothers, we undertook regression analyses aiming to determine the conditional distribution of mother-reported ARI and diarrhea given various risk factors (as described in the **Methods**; section, "Risk factor analysis of household survey data"). Variables extracted from DHS/MICS surveys and other sources are listed in **Table S27**. The results of these analyses are presented in **Tables S28-S30** and **Extended data figures 3-5**. Here we briefly summarize the findings of these analyses as well as our interpretation of certain results.

Undernutrition (measured via weight-for-height Z scores) predicted higher risk of ARI (at ages 24-59 months and 0-59 months; **Tables S28-S29**) as well as diarrhea (at ages 0-23 months; **Table S30**). A decrease by one Z score in weight-for-height was associated with a nearly 10-fold greater increase in risk of diarrhea, as compared to the effect of the same decrease in weight-for-height on a child's risk of ARI. Children's risk of ARI declined with older age by a similar factor over the period from 24 to 59 months and from 0 to 59 months, whereas risk of diarrhea increased slightly with older age over the first two years of life. As compared to children residing in rural areas, those residing in urban areas experienced higher risk of both ARI and diarrhea. Incidence rates of both ARI and diarrhea declined over the survey years included in our study (2006-2018), and were lower in countries with higher GDP per capita.

Children residing in households using solid fuel sources (defined as coal/lignite, charcoal, wood, straw/shrubs/grass, animal dung, or agricultural crop residue) as the primary material for cooking experienced higher rates of ARI both at ages 24-59 months and over the first five years of life, overall; other household factors listed in **Table S27** were not found to predict risk of ARI. In addition, children with access to improved water and sanitation sources experienced lower rates of diarrhea at ages 0-23 months (**Extended data figure 3**). We found the effect of increases in countries' GDP per capita on children's risk of diarrhea to differ with respect to children's access to improved water and sanitation. Among children with access to improved water and sanitation, increases in GDP per capita were associated with marked declines in diarrhea incidence rates. These declines were less strongly evident among children either without improved water access or those relying on unimproved sanitation or open defecation. Access to electricity in the household also predicted lower risk of diarrhea at ages 0-23 months.

Among children whose mothers completed primary, secondary, and post-secondary education, we observed reductions in children's risk of both ARI and diarrhea in association with higher levels of maternal educational attainment. Unexpectedly, we estimated that children whose mothers had not completed primary education

experienced lower risk of both ARI and diarrhea, resulting in a discontinuity in this trend toward lower risk with higher levels of maternal education. Because previous studies aiming to validate DHS questionnaires[1,2] and other survey instruments[3] have reported lower reliability of mother-reported illness measures among children whose mothers were less educated, we assumed for simulation analyses that the estimated intercepts for rates of ARI and diarrhea among children whose mothers completed only primary education applied as well to children whose mothers had not completed any level of education.

We also assessed whether variables included in **Table S27** were associated with antibiotic receipt among children who experienced ARI and diarrhea. Children in wealthier households (defined by a country-specific household wealth index) were more likely to receive antibiotics for both ARI (at ages 24-59 months and 0-59 months) and diarrhea (**Tables S31-S33**). Children experiencing ARI who received antibiotics were younger than those who did not, and were more likely to have experienced fever concomitantly. Among children who experienced diarrhea, those receiving antibiotics were older, more likely to live in a rural setting, and more likely to have access to electricity in their household than diarrhea cases that did not receive antibiotics; in addition, antibiotic-treated diarrhea cases were more likely to be undernourished (**Table S33**). For both ARI and diarrhea, the likelihood of receiving antibiotics peaked among children whose mothers had completed secondary education. We are not aware of specific biases that could account for this trend in mother-reported antibiotic treatment of ARI and diarrhea cases, and thus did not apply corrections similar to those used in the analyses of ARI and diarrhea incidence when estimating country-specific incidence.

We also included data on retail sales of all antibiotics (measured in defined daily doses per 1000 population)[4] measured at the country or regional level. Although the association of this measure with antibiotic treatment was inconsistent, we noted greater probability of antibiotic treatment of ARI cases among countries with higher retail antibiotic sales in the Central Asia, Middle East, and South and Southeast Asia regions; for diarrhea, a similar association emerged in South American and South and Southeast Asian countries. We also identified regional differences in the association between countries' GDP per capita and the probability for ARI and diarrhea cases to receive antibiotics (**Extended data figures 4-5**). Unexpectedly, we estimated lower probabilities of antibiotic treatment of both ARI and diarrhea cases in later survey years. We note that our analysis cannot rule out certain confounding factors that may have contributed to this estimate, such as increased reporting of less-severe disease over time. Regardless of whether this finding was true or artefactual, controlling for the estimated temporal trend provided a basis for standardizing our estimates of the incidence of antibiotic-treated ARI and diarrhea from survey data collected over a wide (13-year) timespan.

Text S3: Supplemental references.

1. Hazir, T, *et al.* Measuring coverage in MNCH: a prospective validation study in Pakistan and Bangladesh on measuring correct treatment of childhood pneumonia. *PLoS Med.* **10**, e1001422 (2013).
2. Campbell, H. *et al.* Measuring coverage in MNCH: challenges in monitoring the proportion of young children with pneumonia who receive antibiotic treatment. *PLoS Med* **10**, e1001421 (2013).
3. Haaga, J.G. Reliability of retrospective survey data on infant feeding. *Demography* **25**, 307-14 (1988).
4. Center for Disease Dynamics, Economics & Policy. ResistanceMap. 2020. https://resistancemap.cddep.org/AntibioticUse.php. Date accessed: 7 February 2020.
5. Cutts, F. *et al.* Efficacy of nine-valent pneumococcal conjugate vaccine against pneumonia and invasive pneumococcal disease in The Gambia: Randomised, double-blind, placebo-controlled trial. *Lancet* (2005), doi:10.1016/S0140-6736(05)71876-6.
6. Klugman, K. P. *et al.* A trial of a 9-valent pneumococcal conjugate vaccine in children with and those without HIV infection. *N. Engl. J. Med*. **349**, 1341–1348 (2003).
7. Tregnaghi, M.W. *et al.* Efficacy of pneumococcal nontypable Haemophilus influenzae protein D conjugate vaccine (PHiD-CV) in young Latin American children: A double-blind randomized controlled trial. *PLoS Med*. 11, e1001657 (2014).
8. Eskola, J. *et al.* Efficacy of a pneumococcal conjugate vaccine against otitis media. *N Engl. J. Med.* **344**, 403–409 (2001).

9. Prymula, R. *et al.* Pneumococcal capsular polysaccharides conjugated to protein D for prevention of acute otitis media caused by both *Streptococcus pneumoniae* and non-typeable *Haemophilus influenzae*: A randomised double-blind efficacy study. *Lancet*. **367**, 740–748 (2006).

10. O'Brien, K. L. *et al.* Randomized, controlled trial efficacy of pneumococcal conjugate vaccine against otitis media Among Navajo and White Mountain Apache infants. *Pediatr. Infect. Dis. J.* **27**, 71–73 (2008).

11. Li, R. C. *et al.* Human rotavirus vaccine (RIX4414) efficacy in the first two years of life: A randomized, placebo-controlled trial in China. *Hum. Vaccines Immunother*. (2014), doi:10.4161/hv.26319.

12. Linhares, A. C. *et al.* Efficacy and safety of an oral live attenuated human rotavirus vaccine against rotavirus gastroenteritis during the first 2 years of life in Latin American infants: a randomised, double-blind, placebo-controlled phase III study. *Lancet*. **371**, 1181-1189 (2008).

13. Justino, M. C. A. *et al.* Oral live attenuated human rotavirus vaccine (RotarixTM) offers sustained high protection against severe G9P[8] rotavirus gastroenteritis during the first two years of life in Brazilian children. *Mem. Inst. Oswaldo Cruz*. **107**, 846–853 (2012).

14. Tregnaghi, M. W. *et al.* Human rotavirus vaccine is highly efficacious when coadministered with routine expanded program of immunization vaccines including oral poliovirus vaccine in Latin America. *Pediatr. Infect. Dis. J.* **30**; e103-8 (2011).

15. Madhi, S. A. *et al.* Effect of human rotavirus vaccine on severe diarrhea in African Infants. *N Engl. J Med.* **362**, 289–298 (2010).

16. Kotloff, K. L. *et al*. Burden and aetiology of diarrhoeal disease in infants and young children in developing countries (the Global Enteric Multicenter Study, GEMS): A prospective, case-control study. *Lancet.* **382**, 209–222 (2013).

17. O'Brien, K. L. *et al.*, Causes of severe pneumonia requiring hospital admission in children without HIV infection from Africa and Asia: the PERCH multi-country case-control study. *Lancet* (2019), doi:10.1016/s0140-6736(19)30721-4.

18. Lewnard, J. A. *et al.* Pan-serotype reduction in progression of *Streptococcus pneumoniae* to otitis media after rollout of pneumococcal conjugate vaccines. *Clin. Infect Dis.* **65**, 1853–1861 (2017).

19. Shouval D. S., *et al.* Serotype coverage of invasive and mucosal pneumococcal disease in Israeli children younger than 3 years by various pneumococcal conjugate vaccines. *Pediatr. Infect. Dis. J.* (2009). Doi:10.1097/Inf.0b013e31818e0e2e.

20. Hammitt, L. L. *et al.* Effect of ten-valent pneumococcal conjugate vaccine on invasive pneumococcal disease and nasopharyngeal carriage in Kenya: a longitudinal surveillance study. *Lancet* **393**, 2146–2154 (2019).

21. Usuf, E. *et al*. Persistence of nasopharyngeal pneumococcal vaccine serotypes and incrase of nonvaccine serotypes among vaccinated infants and their mothers 5 years after introduction of pneumococcl conjugate vaccine 13 in The Gambia. *Clin. Infect. Dis.* **68**, 1512–1521 (2019).

22. Heinsbroek, E. *et al.* Pneumococcal carriage in households in Karonga District, Malawi, before and after introduction of 13-valent pneumococcal conjugate vaccination. *Vaccine* **36**, 7369–7376 (2018).

Table S1. Characteristics of children eligible for inclusion in case-control studies.

Characteristic		ARI cases	ARI-negative controls	Diarrhea cases	Diarrhea-negative controls
Total children		*N*=5342	*N*=57856	*N*=9944	*N*=40059
Age					
	0-23 months	3263	38736	7676	26432
	24-59 months	2079	19120	2268	13627
Sex					
	Female	2502	28651	4639	20306
	Male	2840	29205	5305	19753
Residence					
	Rural	4031	41025	7376	28436
	Urban	1311	16831	2568	11623
Country					
	Afghanistan	1942	1282	– –	– –
	Angola	168	4835	996	4601
	Armenia	10	738	45	850
	Burundi	419	4695	1534	3756
	Ethiopia	211	4070	604	3752
	Haiti	– –	– –	678	1939
	Lao People's Dem. Rep.	54	3126	– –	– –
	Malawi	393	6408	2094	5088
	Nepal	32	1040	– –	– –
	Pakistan	733	3819	– –	– –
	Philippines	64	4163	– –	– –
	Senegal	254	4515	1255	3455
	Sierra Leone	179	6929	662	6916
	South Africa	36	1040	188	1141
	Tajikistan	205	4249	517	2355
	Tanzania	537	4338	751	4008
	Uganda	105	2609	– –	– –
	Zimbabwe			620	2198
Wealth quintile					
	1	1459	15107	2525	10401
	2	1225	13250	2341	9037
	3	1038	11517	2040	7986
	4	941	9405	1759	6509
	5	679	8577	1279	6126
Pentavalent vaccine doses					
	0	1589	13721	1288	8709
	1	76	761	145	652
	2	88	716	566	2574
	≥3	3589	42658	7945	28124
PCV10/13 doses					
	0	2224	18351	1243	8737
	1	– –	– –	100	493
	2	– –	– –	567	2537
	≥3	3118	39505	6759	23737
	Unavailable	– –	– –	1275	4555
Rotavirus vaccine doses					
	0	405	11118	2209	12039
	1	34	684	– –	– –
	≥2	1477	27354	7735	28020
Care received					
	No care	1754	– –	2556	– –
	Treatment/advice sought	3294	– –	7382	– –
	Rehydration received	– –	– –	5220	– –
	Antibiotic received	1913	– –	1437	– –

The eligible population for the case-control studies of PCV10/13 and rotavirus vaccines comprised individuals who had received either no vaccine doses or a full series (≥3 doses of PCV7/13; ≥2 doses of rotavirus vaccine).

Table S2: Sample sizes for case-control studies.

Exposure	Outcome	Population included in analyses					
		Ages 0-59 months		*Ages 0-23 months*		*Ages 24-59 months*	
		Cases	Controls	Cases	Controls	Cases	Controls
≥PCV10/13 doses							
	Primary endpoints						
	All ARI cases	2103-2135	6304-6401	1555-1583	4667-4753	539-558	1607-1677
	ARI for which treatment/advice was sought	1173-1197	3516-3591	880-901	2643-4713	289-301	857-895
	Antibiotic-treated ARI	367-379	1099-1137	292-304	878-916	73-76	210-229
	Negative control endpoints						
	All diarrhea cases	6072-6128	18208-18378	4496-4552	13474-13620	1562-1596	4701-4798
	Diarrhea for which treatment/advice was sought	4232-4271	12696-12813	3264-3301	9756-9880	962-979	2906-2972
	Antibiotic-treated diarrhea	898-907	2695-2724	732-739	2192-2224	166-168	489-516
≥2 rotavirus vaccine doses							
	Primary endpoints						
	All diarrhea cases	7011-7073	21031-21216	5090-5146	15266-15424	1905-1940	5727-5826
	Diarrhea cases for which care/advice was sought	5710-1763	17129-17291	4232-4279	12677-12823	1463-1488	4405-4498
	Diarrhea cases receiving rehydration	4358-4402	13077-13207	3277-3313	9808-9922	1077-1093	3233-3315
	Antibiotic-treated diarrhea	1300-1315	3901-3948	1019-1031	3048-3094	278-285	834-873
	Negative control endpoints						
	All ARI	4545-4602	13638-13807	3203-3254	9621-9749	1326-1362	3991-4082
	ARI for which treatment/advice was sought	737-761	2210-2283	585-610	1753-1829	146-156	439-471
	Antibiotic-treated ARI	335-344	671-688	275-284	549-571	59-61	113-126

We indicate the number of children included in analyses of vaccine effectiveness against each endpoint; the number of children is presented as a range across all sampled match lists. Due to the limited number of matches that could be assembled, we matched cases to two controls each for assessments of rotavirus vaccine effectiveness against antibiotic-treated ARI. All other analyses matched three controls to each case.

Table S3: Effectiveness of ≥3 PCV10/13 doses against various endpoints.

**Age group**	**Vaccine effectiveness**		**Validation**	
	Endpoint	*VE (95% CI), %*	*Negative-control endpoint*	*VE (95% CI), %*
Ages 0-59 months				
	All ARI cases	5.2 (0.8, 9.6)	All diarrhea cases	1.7 (–2.8, 5.8)
	ARI for which treatment/advice was sought	4.9 (–1.1, 10.7)	Diarrhea for which treatment/advice was sought	2.0 (–2.8, 7.0)
	Antibiotic-treated ARI	8.7 (–1.3, 19.7)	Antibiotic-treated diarrhea	–5.5 (–16.4, 6.0)
Ages 0-23 months				
	All ARI cases	1.5 (–5.1, 7.9)	All diarrhea cases	5.3 (–1.8, 12.0)
	ARI for which treatment/advice was sought	–0.2 (–9.4, 8.4)	Diarrhea for which treatment/advice was sought	5.1 (–1.8, 11.3)
	Antibiotic-treated ARI	5.4 (–6.7, 18.7)	Antibiotic-treated diarrhea	–10.6 (–29.1, 5.8)
Ages 24-59 months				
	All ARI cases	10.0 (3.7, 16.0)	All diarrhea cases	–0.7 (–6.3, 4.3)
	ARI for which treatment/advice was sought	11.5 (3.2, 19.9)	Diarrhea for which treatment/advice was sought	0.0 (–7.2, 6.8)
	Antibiotic-treated ARI	19.7 (3.4, 43.4)	Antibiotic-treated diarrhea	2.6 (–11.7, 20.7)

Vaccine effectiveness estimates are calculated as one minus the matched odds ratio and presented on the percentage scale. Analyses match children with the target endpoint to asymptomatic controls on country, age (within 1 month), visit timing (within 1 month), wealth quintile, urbanicity, and pentavalent vaccine doses received. Analyses of negative-control (diarrhea) endpoints match on rotavirus vaccine doses received, additionally. The population available for analyses included: 5,342 ARI cases (among whom 3,294 sought treatment/advice and 1,913 received an antibiotic) and 57,856 controls without ARI; and 9,944 diarrhea cases (of whom 7,382 sought treatment/advice and 1,437 received an antibiotic) and 40,059 controls without diarrhea (**Tables S1-S2**). Quantiles are derived via 2,000 independent draws from the distribution of estimates.

Table S4: Effectiveness of ≥2 rotavirus vaccine doses against various endpoints.

Age group	Vaccine effectiveness		Validation	
	Endpoint	*VE (95% CI), %*	*Negative-control endpoint*	*VE (95% CI), %*
Ages 0-59 months				
	All diarrhea cases	3.3 (0.6, 5.9)	All ARI cases	2.1 (–3.1, 6.8)
	Diarrhea cases for which care/advice was sought	7.6 (5.2, 10.2)	ARI for which treatment/advice was sought	–1.7 (–15.6, 9.0)
	Diarrhea cases receiving rehydration	7.0 (4.2, 9.7)	– –	– –
	Antibiotic-treated diarrhea	8.1 (2.8, 14.1)	Antibiotic-treated ARI	–6.6 (–24.0, 11.5)
Ages 0-23 months				
	All diarrhea cases	4.1 (0.2, 7.6)	All ARI cases	3.0 (–4.3, 9.3)
	Diarrhea cases for which care/advice was sought	9.7 (6.6, 12.8)	ARI for which treatment/advice was sought	–0.4 (–17.6, 12.6)
	Diarrhea cases receiving rehydration	9.6 (6.2, 12.9)	– –	– –
	Antibiotic-treated diarrhea	11.4 (4.0, 18.6)	Antibiotic-treated ARI	–9.5 (–29.4, 16.3)
Ages 24-59 months				
	All diarrhea cases	1.8 (–2.2, 5.5)	All ARI cases	1.2 (–6.9, 8.5)
	Diarrhea cases for which care/advice was sought	3.8 (–0.8, 8.1)	ARI for which treatment/advice was sought	–3.2 (–28.7, 15.0)
	Diarrhea cases receiving rehydration	2.0 (–3.5, 7.2)	– –	– –
	Antibiotic-treated diarrhea	1.9 (–8.0, 11.8)	Antibiotic-treated ARI	0.0 (–29.9, 35.2)

Vaccine effectiveness estimates are calculated as one minus the matched odds ratio and presented on the percentage scale. Analyses match children with the target endpoint to asymptomatic controls on country, age (within 1 month), visit timing (within 1 month), wealth quintile, urbanicity, and pentavalent vaccine doses received. Because all countries in this analysis use Rotarix in their national immunization program, we defined ≥2 doses as a full vaccination series. Analyses of negative-control (ARI) endpoints match on PCV doses received, additionally. The population available for analyses included: 5,342 ARI cases (among whom 3,294 sought treatment/advice and 1,913 received an antibiotic) and 57,856 controls without ARI; and 9,944 diarrhea cases (of whom 7,382 sought treatment/advice, 5,220 received rehydration, and 1,437 received an antibiotic) and 40,059 controls without diarrhea (**Tables S1-S2**). Quantiles are derived via 2,000 independent draws from the distribution of estimates.

Table S5: Effectiveness of ≥2 rotavirus vaccine doses against diarrhea and antibiotic-treated diarrhea among children ages 0-23 months in low- and middle-income countries.

Endpoint	Rotavirus vaccine effectiveness by setting (95% CI), %	
	Middle-income countries	*Low-income countries*
All diarrhea cases	8.7 (1.6, 14.8)	2.7 (–2.1, 6.9)
Diarrhea cases for which care/advice was sought	18.1 (12.1, 24.4)	6.9 (3.1, 10.4)
Diarrhea cases receiving rehydration	18.1 (11.0, 24.9)	6.8 (2.7, 10.6)
Antibiotic-treated diarrhea	16.6 (0.0, 32.1)	10.0 (2.0, 18.0)

Vaccine effectiveness estimates are calculated as one minus the matched odds ratio and presented on the percentage scale. Analyses match children with the target endpoint to asymptomatic controls on country, age (within 1 month), visit timing (within 1 month), wealth quintile, urbanicity, and pentavalent vaccine doses received. Because all countries in this analysis use Rotarix in their national immunization program, we defined ≥2 doses as a full vaccination series. The population available for analyses included 9,944 diarrhea cases (of whom 7,382 sought treatment/advice and 1,437 received an antibiotic) and 40,059 controls without diarrhea (**Tables S1**-**S2**). Quantiles are derived via 2,000 independent draws from the distribution of estimates.

Table S6. Variance explained by subnational administrative groupings in the case-control study.

Outcome		Sum of squared errors		Proportion of variance explained
		Accounting for country[1]	*Accounting for subnational region*[1]	
ARI endpoints				
	Any ARI case	2612.3	2563.2	1.9%
	ARI case for which treatment/advice was sought	1527.9	1499.2	1.9%
	Antibiotic-treated ARI case	607.3	602.5	0.8%
Diarrhea endpoints				
	Any diarrhea case	7373.3	7283.0	1.2%
	Diarrhea case for which treatment/advice was sought	5776.6	5712.9	1.1%
	Diarrhea case receiving rehydration	4314.3	4273.1	1.0%
	Antibiotic-treated diarrhea case	1328.7	1310.3	1.4%
Vaccine receipt				
	Full PCV10/13 series received	3676.0	3349.4	8.9%
	Full rotavirus vaccine series received	2754.9	2695.7	2.1%

We present the sum of squared errors (model residuals) for regression models defining each ARI and diarrhea endpoint, and vaccine receipt, as dependent variables, with the matching factors included in the case control analysis defined as predictors (see Methods, section “Assessment of residual confounding”). We compute the proportion of variance explained by accounting for subnational regions, beyond country alone, via the difference in summed squared errors for each model, divided by the summed squared errors for the model accounting for country only.

1. Models additionally control for age (by month), calendar time (by month), wealth quintile within country, urbanicity, and pentavalent vaccine doses received (each as intercepts); additionally, the ARI outcomes control for mother-reported fever, consistent with the primary case-control study.

Table S7. Meta-analysis of vaccine efficacy studies involving pathogen-specific endpoints.

Vaccine	Endpoint	Study (with citation)	Setting	Sample size	Efficacy estimate (95% CI), %[1]
PCV7/9/10/13	Invasive pneumococcal disease with vaccine-targeted serotype				
		Cutts et al.[5]	The Gambia	16,340	77 (51, 90)
		Klugman et al.[6]	South Africa	39,836	83 (23, 96)
		Tregnaghi et al.[7]	Latin America	20,496	100 (77.3, 100)[2]
		Pooled est.			81.2 (63.1, 90.5)
	Acute otitis media caused by vaccine-targeted serotype				
		Tregnaghi et al.[7]	Latin America	5,989	57 (44, 67)
		Eskola et al.[8]	Finland	1,580	55.7 (21.5, 75.0)
		Prymula et al.[9]	Finland	4,907	52.6 (35.0, 65.5)
		O'Brien et al.[10]	US (Navajo/White Mountain Apache)	856	64 (–34, 90)
		Pooled est.			55.6 (46.1, 63.4)
Monovalent human rotavirus vaccine (Rotarix)	Rotavirus gastroenteritis (middle-income countries)				
		Li et al.[11]	China	3,148	72.1 (54.1, 83.6)
		Linhares et al.[12]	Latin America	14,286	80.5 (71.3, 87.1)
		Justino et al.[13]	Brazil	630	72.3 (37.5, 89.1)
		Tregnaghi et al.[14]	Latin America	6,310	81.6 (54.4, 93.5)
		Madhi et al.[15]	South Africa	2,904	76.9 (56.0, 88.4)
		Pooled est.			77.5 (70.3, 83.0)
	Rotavirus gastroenteritis (low-income countries)				
		Madhi et al.[15]	Malawi	1,513	49.4 (19.2, 68.3)
		Pooled est.			49.4 (19.2, 68.3)

Pooled estimates are obtained from inverse variance-weighted random-effects meta-analysis models using log transformations of the input $1–VE$. We include the total sample size (including recipients of vaccine and placebo) of the per-protocol population of each study in the table; in total, estimates are based on data from: 76,672 children (PCV efficacy against invasive pneumococcal disease); 13,332 children (PCV efficacy against acute otitis media); 27,278 children (rotavirus vaccine efficacy in middle-income countries); and 1,513 children (rotavirus vaccine efficacy in low-income countries).

1. Estimates were included at the level of precision reported in the original studies. Quantiles are derived via 10,000 independent draws from the distribution of estimates.
2. A 99% efficacy estimate was used in order to accommodate the study in the pooled random effects model requiring a log transformation of the relative risk measure.

Table S8. Attributable fraction estimates.

Pathogen	Stratum	Vaccine efficacy (95% CI), %	Endpoint	Attributable fraction (95% CI), %
Vaccine-serotype *S. pneumoniae*	Ages 23-59 months			
		IPD: 81.2 (63.1, 90.5)	All ARI cases	12.5 (4.6, 21.1)
		IPD: 81.2 (63.1, 90.5)	ARI for which treatment/advice was sought	14.3 (3.8, 25.9)
		IPD: 81.2 (63.1, 90.5)	Antibiotic-treated ARI	24.8 (4.3, 54.6)
	Ages 23-59 months			
		AOM: 55.6 (46.1, 63.4)	All ARI cases	18.1 (10.8, 25.8)
		AOM: 55.6 (46.1, 63.4)	ARI for which treatment/advice was sought	20.7 (10.9, 31.3)
		AOM: 55.6 (46.1, 63.4)	Antibiotic-treated ARI	35.8 (15.7, 60.6)
	Ages 0-59 months			
		IPD: 81.2 (63.1, 90.5)	All ARI cases	6.5 (1.0, 12.5)
		IPD: 81.2 (63.1, 90.5)	ARI for which treatment/advice was sought	6.1 (–1.4, 13.9)
		IPD: 81.2 (63.1, 90.5)	Antibiotic-treated ARI	10.9 (–1.6, 25.4)
	Ages 0-59 months			
		AOM: 55.6 (46.1, 63.4)	All ARI cases	9.4 (1.4, 17.9)
		AOM: 55.6 (46.1, 63.4)	ARI for which treatment/advice was sought	8.9 (–2.1, 20.0)
		AOM: 55.6 (46.1, 63.4)	Antibiotic-treated ARI	15.8 (–2.3, 36.4)
Rotavirus	Ages 0-23 months, MICs			
		77.5 (70.3, 83.0)	All diarrhea cases	11.1 (1.9, 19.5)
		77.5 (70.3, 83.0)	Diarrhea cases for which care/advice was sought	23.5 (15.6, 31.9)
		77.5 (70.3, 83.0)	Diarrhea cases receiving rehydration	23.4 (14.2, 32.5)
		77.5 (70.3, 83.0)	Antibiotic-treated diarrhea	21.3 (0.0, 42.4)
	Ages 0-23 months, LICs			
		49.6 (18.7, 68.8)	All diarrhea cases	5.7 (–5.0, 20.1)
		49.6 (18.7, 68.8)	Diarrhea cases for which care/advice was sought	14.1 (5.7, 37.7)
		49.6 (18.7, 68.8)	Diarrhea cases receiving rehydration	14.1 (5.7, 37.7)
		49.6 (18.7, 68.8)	Antibiotic-treated diarrhea	22.7 (4.5, 60.6)
	Ages 0-23 months, all LMICs[1]			
		– –	All diarrhea cases	10.8 (2.3, 18.4)
		– –	Diarrhea cases for which care/advice was sought	22.8 (15.4, 30.8)
		– –	Diarrhea cases receiving rehydration	22.4 (14.1, 31.3)
		– –	Antibiotic-treated diarrhea	21.6 (2.1, 40.3)

Estimates indicate the proportion of ARI and diarrhea (each including all cases, cases for which treatment was sought, and antibiotic-treated cases) attributable to vaccine-serotype *S. pneumoniae* and rotavirus, respectively, estimated using the model in Eq. 3b and taking, as inputs, meta-analytic estimates of vaccine efficacy against disease due to the targeted pathogens as well as estimates of vaccine effectiveness against all-cause outcomes from the case-control study. Quantiles are derived via 10,000 independent draws from the distribution of estimates.

1. Estimated attributable fractions for diarrhea endpoints across all LMICs are obtained via a weighted average of attributable fraction estimates for MICs and LICs, with weights defined as the total population of children ages 0-23 months in MIC and LIC settings.

Table S9: Countries included in analyses.

Country	Data source	Survey years (if applicable)
**African Region**		
Algeria	Extrapolated	– –
Angola	DHS	2015-16
Benin	DHS	2008
Botswana	Extrapolated	– –
Burkina Faso	DHS	2010
Burundi	DHS	2016-17
Cabo Verde	Extrapolated	– –
Cameroon	DHS	2011
Cent. African Rep.	Extrapolated	– –
Chad	DHS	2014-15
Comoros	DHS	2012
Congo, Rep.	DHS	2011-12
Congo, Dem. Rep.	DHS	2013-14
Cote d'Ivoire	DHS	2011-12
Equatorial Guinea	Extrapolated	– –
Eritrea	Extrapolated	– –
Eswatini	DHS	2006-7
Ethiopia	DHS	2016
Gabon	DHS	2012
Gambia, The	DHS	2013
Ghana	DHS	2014
Guinea	DHS	2012
Guinea-Bissau	MICS	2014
Kenya	DHS	2014
Lesotho	DHS	2014
Liberia	DHS	2013
Madagascar	Extrapolated	– –
Malawi	DHS	2015-16
Mali	DHS	2012-13
Mauritania	MICS	2015
Mauritius	Extrapolated	– –
Mozambique	DHS	2015
Namibia	DHS	2013
Niger	DHS	2012
Nigeria	DHS	2013
Rwanda	DHS	2014-15
Sao Tome and Principe	DHS	2008-9
Senegal	DHS	2017
Sierra Leone	MICS	2017
South Africa	DHS	2016
South Sudan	Extrapolated	– –
Tanzania, United Rep.	DHS	2015-16
Togo	DHS	2013-14
Uganda	DHS	2016
Zambia	DHS	2013-14
Zimbabwe	DHS	2015
**Region of the Americas**		
Belize	MICS	2015-16
Bolivia, Plurinational State	DHS	2008
Brazil	Extrapolated	– –
Colombia	DHS	2009-10
Costa Rica	Extrapolated	– –
Cuba	MICS	2014
Dominica	Extrapolated	– –
Dominican Rep.	MICS	2013-14
Ecuador	Extrapolated	– –
El Salvador	MICS	2014
Grenada	Extrapolated	– –
Guatemala	DHS	2014-15
Guyana	DHS	2009
Haiti	DHS	2016-17
Honduras	DHS	2011-12
Jamaica	Extrapolated	– –
Mexico	MICS	2015
Nicaragua	Extrapolated	– –
Paraguay	MICS	2016

Peru	DHS	2012
St. Lucia	Extrapolated	– –
St. Vincent and the Grenadines	Extrapolated	– –
Suriname	Extrapolated	– –
Venezuela, Bolivarian Rep.	Extrapolated	– –
**Eastern Mediterranean Region**		
Afghanistan	DHS	2016-17
Djibouti	Extrapolated	– –
Egypt, Arab Rep.	MICS	2013-14
Iran, Islamic Rep.	Extrapolated	– –
Iraq	Extrapolated	– –
Jordan	DHS	2012
Lebanon	Extrapolated	– –
Libyan Arab Rep.	Extrapolated	– –
Morocco	Extrapolated	– –
Pakistan	DHS	2017-18
Palestinian Terr.	MICS	2014
Somalia	Extrapolated	– –
Sudan	MICS	2014
Tunisia	Extrapolated	– –
Yemen, Rep.	DHS	2013
**European Region**		
Albania	DHS	2017-18
Armenia	DHS	2015-16
Azerbaijan	DHS	2006
Belarus	Extrapolated	– –
Bosnia and Herzegovina	Extrapolated	– –
Bulgaria	Extrapolated	– –
Georgia	Extrapolated	– –
Kazakhstan	Extrapolated	– –
Kosovo	MICS	2013-14
Kyrgyzstan	DHS	2012
Moldova, Rep.	Extrapolated	– –
Montenegro	MICS	2013
North Macedonia	Extrapolated	– –
Romania	Extrapolated	– –
Russian Fed.	Extrapolated	– –
Serbia	Extrapolated	– –
Tajikistan	DHS	2017
Turkey	Extrapolated	– –
Turkmenistan	MICS	2015-16
Ukraine	Extrapolated	– –
Uzbekistan	Extrapolated	– –
**Southeast Asia Region**		
Bangladesh	DHS	2014
Bhutan	Extrapolated	– –
India	DHS	2015-16
Indonesia	DHS	2012
Maldives	DHS	2016-17
Myanmar	DHS	2015-16
Nepal	DHS	2017-18
Sri Lanka	Extrapolated	– –
Thailand	MICS	2015-16
Timor-Leste	DHS	2016
**Western Pacific Region**		
American Samoa	Extrapolated	– –
Cambodia	DHS	2014
China, People's Rep.	Extrapolated	– –
Fiji	Extrapolated	– –
Kiribati	Extrapolated	– –
Lao People's Dem. Rep.	MICS	2017
Malaysia	Extrapolated	– –
Marshall Islands	Extrapolated	– –
Micronesia, Fed.	Extrapolated	– –
Mongolia	MICS	2013-14
Nauru	Extrapolated	– –

Papua New Guinea	Extrapolated	– –
Philippines	DHS	2017
Samoa	Extrapolated	– –
Solomon Islands	Extrapolated	– –
Tonga	Extrapolated	– –
Tuvalu	Extrapolated	– –
Vanuatu	Extrapolated	– –
Viet Nam	MICS	2013-14

Analyses included data from the most recently conducted DHS or MICS survey for each country meeting the World Bank definitions of low income, lower middle income, and upper middle income in 2018. Where survey data were not available for a given country, incidence was estimated via cross-validation on the basis of socioeconomic indicator variables listed in **Table S9**.

Table S10: World Bank indicator variables included in cross-validation analyses.

Variable	Variable Code
Adolescent fertility rate (births per 1,000 women ages 15-19)	[SP.ADO.TFRT]
Adults (ages 15+) and children (0-14 years) living with HIV	[SH.HIV.TOTL]
Adults (ages 15+) and children (ages 0-14) newly infected with HIV	[SH.HIV.INCD.TL]
Adults (ages 15+) living with HIV	[SH.DYN.AIDS]
Adults (ages 15+) newly infected with HIV	[SH.HIV.INCD]
Age at first marriage, female	[SP.DYN.SMAM.FE]
Age at first marriage, male	[SP.DYN.SMAM.MA]
Age dependency ratio (% of working-age population)	[SP.POP.DPND]
Age dependency ratio, old	[SP.POP.DPND.OL]
Age dependency ratio, young	[SP.POP.DPND.YG]
Age population, age 0, female, interpolated	[SP.POP.AG00.FE.IN]
Age population, age 0, male, interpolated	[SP.POP.AG00.MA.IN]
Age population, age 01, female, interpolated	[SP.POP.AG01.FE.IN]
Age population, age 01, male, interpolated	[SP.POP.AG01.MA.IN]
Age population, age 02, female, interpolated	[SP.POP.AG02.FE.IN]
Age population, age 02, male, interpolated	[SP.POP.AG02.MA.IN]
Age population, age 03, female, interpolated	[SP.POP.AG03.FE.IN]
Age population, age 03, male, interpolated	[SP.POP.AG03.MA.IN]
Age population, age 04, female, interpolated	[SP.POP.AG04.FE.IN]
Age population, age 04, male, interpolated	[SP.POP.AG04.MA.IN]
Age population, age 05, female, interpolated	[SP.POP.AG05.FE.IN]
Age population, age 05, male, interpolated	[SP.POP.AG05.MA.IN]
Age population, age 06, female, interpolated	[SP.POP.AG06.FE.IN]
Age population, age 06, male, interpolated	[SP.POP.AG06.MA.IN]
Age population, age 07, female, interpolated	[SP.POP.AG07.FE.IN]
Age population, age 07, male, interpolated	[SP.POP.AG07.MA.IN]
Age population, age 08, female, interpolated	[SP.POP.AG08.FE.IN]
Age population, age 08, male, interpolated	[SP.POP.AG08.MA.IN]
Age population, age 09, female, interpolated	[SP.POP.AG09.FE.IN]
Age population, age 09, male, interpolated	[SP.POP.AG09.MA.IN]
Age population, age 10, female, interpolated	[SP.POP.AG10.FE.IN]
Age population, age 10, male, interpolated	[SP.POP.AG10.MA.IN]
Age population, age 11, female, interpolated	[SP.POP.AG11.FE.IN]
Age population, age 11, male, interpolated	[SP.POP.AG11.MA.IN]
Age population, age 12, female, interpolated	[SP.POP.AG12.FE.IN]
Age population, age 12, male, interpolated	[SP.POP.AG12.MA.IN]
Age population, age 13, female, interpolated	[SP.POP.AG13.FE.IN]
Age population, age 13, male, interpolated	[SP.POP.AG13.MA.IN]
Age population, age 14, female, interpolated	[SP.POP.AG14.FE.IN]
Age population, age 14, male, interpolated	[SP.POP.AG14.MA.IN]
Age population, age 15, female, interpolated	[SP.POP.AG15.FE.IN]
Age population, age 15, male, interpolated	[SP.POP.AG15.MA.IN]
Age population, age 16, female, interpolated	[SP.POP.AG16.FE.IN]
Age population, age 16, male, interpolated	[SP.POP.AG16.MA.IN]
Age population, age 17, female, interpolated	[SP.POP.AG17.FE.IN]
Age population, age 17, male, interpolated	[SP.POP.AG17.MA.IN]
Age population, age 18, female, interpolated	[SP.POP.AG18.FE.IN]
Age population, age 18, male, interpolated	[SP.POP.AG18.MA.IN]

Age population, age 19, female, interpolated	[SP.POP.AG19.FE.IN]
Age population, age 19, male, interpolated	[SP.POP.AG19.MA.IN]
Age population, age 20, female, interpolated	[SP.POP.AG20.FE.IN]
Age population, age 20, male, interpolated	[SP.POP.AG20.MA.IN]
Age population, age 21, female, interpolated	[SP.POP.AG21.FE.IN]
Age population, age 21, male, interpolated	[SP.POP.AG21.MA.IN]
Age population, age 22, female, interpolated	[SP.POP.AG22.FE.IN]
Age population, age 22, male, interpolated	[SP.POP.AG22.MA.IN]
Age population, age 23, female, interpolated	[SP.POP.AG23.FE.IN]
Age population, age 23, male, interpolated	[SP.POP.AG23.MA.IN]
Age population, age 24, female, interpolated	[SP.POP.AG24.FE.IN]
Age population, age 24, male, interpolated	[SP.POP.AG24.MA.IN]
Age population, age 25, female, interpolated	[SP.POP.AG25.FE.IN]
Age population, age 25, male, interpolated	[SP.POP.AG25.MA.IN]
AIDS estimated deaths (UNAIDS estimates)	[SH.DYN.AIDS.DH]
Antiretroviral therapy coverage (% of people living with HIV)	[SH.HIV.ARTC.ZS]
Antiretroviral therapy coverage for PMTCT (% of pregnant women living with HIV)	[SH.HIV.PMTC.ZS]
ARI treatment (% of children under 5 taken to a health provider)	[SH.STA.ARIC.ZS]
Birth rate, crude (per 1,000 people)	[SP.DYN.CBRT.IN]
Births attended by skilled health staff (% of total)	[SH.STA.BRTC.ZS]
Capital health expenditure (% of GDP)	[SH.XPD.KHEX.GD.ZS]
Cause of death, by communicable diseases and maternal, prenatal and nutrition conditions (% of total)	[SH.DTH.COMM.ZS]
Cause of death, by injury (% of total)	[SH.DTH.INJR.ZS]
Cause of death, by non-communicable diseases (% of total)	[SH.DTH.NCOM.ZS]
Children (0-14) living with HIV	[SH.HIV.0014]
Children (ages 0-14) newly infected with HIV	[SH.HIV.INCD.14]
Children orphaned by HIV/AIDS	[SH.HIV.ORPH]
Children with fever receiving antimalarial drugs (% of children under age 5 with fever)	[SH.MLR.TRET.ZS]
Community health workers (per 1,000 people)	[SH.MED.CMHW.P3]
Completeness of birth registration (%)	[SP.REG.BRTH.ZS]
Completeness of birth registration, female (%)	[SP.REG.BRTH.FE.ZS]
Completeness of birth registration, male (%)	[SP.REG.BRTH.MA.ZS]
Completeness of birth registration, rural (%)	[SP.REG.BRTH.RU.ZS]
Completeness of birth registration, urban (%)	[SP.REG.BRTH.UR.ZS]
Completeness of death registration with cause-of-death information (%)	[SP.REG.DTHS.ZS]
Comprehensive correct knowledge of HIV/AIDS, ages 15-24, female (2 prevent ways and reject 3 misconceptions)	[SH.HIV.1524.KW.FE.ZS]
Comprehensive correct knowledge of HIV/AIDS, ages 15-24, male (2 prevent ways and reject 3 misconceptions)	[SH.HIV.1524.KW.MA.ZS]
Comprehensive correct knowledge of HIV/AIDS, ages 15-49, female (2 prevent ways and reject 3 misconceptions)	[SH.HIV.KNOW.FE.ZS]
Comprehensive correct knowledge of HIV/AIDS, ages 15-49, male (2 prevent ways and reject 3 misconceptions)	[SH.HIV.KNOW.MA.ZS]
Condom use with non regular partner, % adults(15-49), female	[SH.CON.AIDS.FE.ZS]
Condom use with non regular partner, % adults(15-49), male	[SH.CON.AIDS.MA.ZS]
Condom use, population ages 15-24, female (% of females ages 15-24)	[SH.CON.1524.FE.ZS]
Condom use, population ages 15-24, male (% of males ages 15-24)	[SH.CON.1524.MA.ZS]
Consumption of iodized salt (% of households)	[SN.ITK.SALT.ZS]
Contraceptive prevalence, any methods (% of women ages 15-49)	[SP.DYN.CONU.ZS]
Contraceptive prevalence, modern methods (% of women ages 15-49)	[SP.DYN.CONM.ZS]
Current health expenditure (% of GDP)	[SH.XPD.CHEX.GD.ZS]
Current health expenditure per capita (current US$)	[SH.XPD.CHEX.PC.CD]
Current health expenditure per capita, PPP (current international $)	[SH.XPD.CHEX.PP.CD]
Death rate, crude (per 1,000 people)	[SP.DYN.CDRT.IN]

Demand for family planning satisfied by any methods (% of married women with demand for family planning)	[SH.FPL.SATI.ZS]
Demand for family planning satisfied by modern methods (% of married women with demand for family planning)	[SH.FPL.SATM.ZS]
Diabetes prevalence (% of population ages 20 to 79)	[SH.STA.DIAB.ZS]
Diarrhea treatment (% of children under 5 receiving oral rehydration and continued feeding)	[SH.STA.ORCF.ZS]
Diarrhea treatment (% of children under 5 who received ORS packet)	[SH.STA.ORTH]
Domestic general government health expenditure (% of current health expenditure)	[SH.XPD.GHED.CH.ZS]
Domestic general government health expenditure (% of GDP)	[SH.XPD.GHED.GD.ZS]
Domestic general government health expenditure (% of general government expenditure)	[SH.XPD.GHED.GE.ZS]
Domestic general government health expenditure per capita (current US$)	[SH.XPD.GHED.PC.CD]
Domestic general government health expenditure per capita, PPP (current international $)	[SH.XPD.GHED.PP.CD]
Domestic private health expenditure (% of current health expenditure)	[SH.XPD.PVTD.CH.ZS]
Domestic private health expenditure per capita (current US$)	[SH.XPD.PVTD.PC.CD]
Domestic private health expenditure per capita, PPP (current international $)	[SH.XPD.PVTD.PP.CD]
Exclusive breastfeeding (% of children under 6 months)	[SH.STA.BFED.ZS]
External health expenditure (% of current health expenditure)	[SH.XPD.EHEX.CH.ZS]
External health expenditure channeled through government (% of external health expenditure)	[SH.XPD.EHEX.EH.ZS]
External health expenditure per capita (current US$)	[SH.XPD.EHEX.PC.CD]
External health expenditure per capita, PPP (current international $)	[SH.XPD.EHEX.PP.CD]
Female headed households (% of households with a female head)	[SP.HOU.FEMA.ZS]
Female population 00-04	[SP.POP.0004.FE]
Female population 05-09	[SP.POP.0509.FE]
Female population 10-14	[SP.POP.1014.FE]
Female population 15-19	[SP.POP.1519.FE]
Female population 20-24	[SP.POP.2024.FE]
Female population 25-29	[SP.POP.2529.FE]
Female population 30-34	[SP.POP.3034.FE]
Female population 35-39	[SP.POP.3539.FE]
Female population 40-44	[SP.POP.4044.FE]
Female population 45-49	[SP.POP.4549.FE]
Female population 50-54	[SP.POP.5054.FE]
Female population 55-59	[SP.POP.5559.FE]
Female population 60-64	[SP.POP.6064.FE]
Female population 65-69	[SP.POP.6569.FE]
Female population 70-74	[SP.POP.7074.FE]
Female population 75-79	[SP.POP.7579.FE]
Female population 80+	[SP.POP.80UP.FE]
Fertility rate, total (births per woman)	[SP.DYN.TFRT.IN]
GNI per capita, Atlas method (current US$)	[NY.GNP.PCAP.CD]
Hospital beds (per 1,000 people)	[SH.MED.BEDS.ZS]
Human capital index (HCI) (scale 0-1)	[HD.HCI.OVRL]
Human capital index (HCI), female (scale 0-1)	[HD.HCI.OVRL.FE]
Human capital index (HCI), female, lower bound (scale 0-1)	[HD.HCI.OVRL.LB.FE]
Human capital index (HCI), female, upper bound (scale 0-1)	[HD.HCI.OVRL.UB.FE]
Human capital index (HCI), lower bound (scale 0-1)	[HD.HCI.OVRL.LB]
Human capital index (HCI), male (scale 0-1)	[HD.HCI.OVRL.MA]
Human capital index (HCI), male, lower bound (scale 0-1)	[HD.HCI.OVRL.LB.MA]
Human capital index (HCI), male, upper bound (scale 0-1)	[HD.HCI.OVRL.UB.MA]
Human capital index (HCI), upper bound (scale 0-1)	[HD.HCI.OVRL.UB]
Immunization, BCG (% of one-year-old children)	[SH.IMM.IBCG]
Immunization, DPT (% of children ages 12-23 months)	[SH.IMM.IDPT]

Immunization, HepB3 (% of one-year-old children)	[SH.IMM.HEPB]
Immunization, Hib3 (% of children ages 12-23 months)	[SH.IMM.HIB3]
Immunization, measles (% of children ages 12-23 months)	[SH.IMM.MEAS]
Immunization, Pol3 (% of one-year-old children)	[SH.IMM.POL3]
Incidence of HIV (% of uninfected population ages 15-49)	[SH.HIV.INCD.ZS]
Incidence of malaria (per 1,000 population at risk)	[SH.MLR.INCD.P3]
Incidence of tuberculosis (per 100,000 people)	[SH.TBS.INCD]
Increase in poverty gap at $1.90 ($ 2011 PPP) poverty line due to out-of-pocket health care expenditure (% of poverty line)	[SH.UHC.NOP1.ZG]
Increase in poverty gap at $1.90 ($ 2011 PPP) poverty line due to out-of-pocket health care expenditure (USD)	[SH.UHC.NOP1.CG]
Increase in poverty gap at $3.10 ($ 2011 PPP) poverty line due to out-of-pocket health care expenditure (% of poverty line)	[SH.UHC.NOP2.ZG]
Increase in poverty gap at $3.10 ($ 2011 PPP) poverty line due to out-of-pocket health care expenditure (USD)	[SH.UHC.NOP2.CG]
Infant and young child feeding practices, all 3 IYCF (% children ages 6-23 months)	[SH.STA.IYCF.ZS]
Labor force, female (% of total labor force)	[SL.TLF.TOTL.FE.ZS]
Labor force, total	[SL.TLF.TOTL.IN]
Life expectancy at birth, female (years)	[SP.DYN.LE00.FE.IN]
Life expectancy at birth, male (years)	[SP.DYN.LE00.MA.IN]
Life expectancy at birth, total (years)	[SP.DYN.LE00.IN]
Lifetime risk of maternal death (%)	[SH.MMR.RISK.ZS]
Lifetime risk of maternal death (1 in: rate varies by country)	[SH.MMR.RISK]
Literacy rate, adult female (% of females ages 15 and above)	[SE.ADT.LITR.FE.ZS]
Literacy rate, adult male (% of males ages 15 and above)	[SE.ADT.LITR.MA.ZS]
Literacy rate, adult total (% of people ages 15 and above)	[SE.ADT.LITR.ZS]
Literacy rate, youth male (% of males ages 15-24)	[SE.ADT.1524.LT.MA.ZS]
Literacy rate, youth total (% of people ages 15-24)	[SE.ADT.1524.LT.ZS]
Low-birthweight babies (% of births)	[SH.STA.BRTW.ZS]
Malaria cases reported	[SH.STA.MALR]
Male population 00-04	[SP.POP.0004.MA]
Male population 05-09	[SP.POP.0509.MA]
Male population 10-14	[SP.POP.1014.MA]
Male population 15-19	[SP.POP.1519.MA]
Male population 20-24	[SP.POP.2024.MA]
Male population 25-29	[SP.POP.2529.MA]
Male population 30-34	[SP.POP.3034.MA]
Male population 35-39	[SP.POP.3539.MA]
Male population 40-44	[SP.POP.4044.MA]
Male population 45-49	[SP.POP.4549.MA]
Male population 50-54	[SP.POP.5054.MA]
Male population 55-59	[SP.POP.5559.MA]
Male population 60-64	[SP.POP.6064.MA]
Male population 65-69	[SP.POP.6569.MA]
Male population 70-74	[SP.POP.7074.MA]
Male population 75-79	[SP.POP.7579.MA]
Male population 80+	[SP.POP.80UP.MA]
Malnutrition prevalence, height for age (% of children under 5)	[SH.STA.STNT.ZS]
Malnutrition prevalence, height for age, female (% of children under 5)	[SH.STA.STNT.FE.ZS]
Malnutrition prevalence, height for age, male (% of children under 5)	[SH.STA.STNT.MA.ZS]
Malnutrition prevalence, weight for age (% of children under 5)	[SH.STA.MALN.ZS]
Malnutrition prevalence, weight for age, female (% of children under 5)	[SH.STA.MALN.FE.ZS]
Malnutrition prevalence, weight for age, male (% of children under 5)	[SH.STA.MALN.MA.ZS]
Maternal leave benefits (% of wages paid in covered period)	[SH.MMR.WAGE.ZS]

Maternal mortality ratio (modeled estimate, per 100,000 live births)	[SH.STA.MMRT]
Maternal mortality ratio (national estimate, per 100,000 live births)	[SH.STA.MMRT.NE]
Mortality caused by road traffic injury (per 100,000 people)	[SH.STA.TRAF.P5]
Mortality from CVD, cancer, diabetes or CRD between exact ages 30 and 70 (%)	[SH.DYN.NCOM.ZS]
Mortality from CVD, cancer, diabetes or CRD between exact ages 30 and 70, female (%)	[SH.DYN.NCOM.FE.ZS]
Mortality from CVD, cancer, diabetes or CRD between exact ages 30 and 70, male (%)	[SH.DYN.NCOM.MA.ZS]
Mortality rate attributed to household and ambient air pollution (per 100,000 population)	[SH.STA.AIRP.P5]
Mortality rate attributed to household and ambient air pollution, age-standardized, female (per 100,000 female population)	[SH.STA.AIRP.FE.P5]
Mortality rate attributed to household and ambient air pollution, age-standardized, male (per 100,000 male population)	[SH.STA.AIRP.MA.P5]
Mortality rate attributed to unintentional poisoning (per 100,000 population)	[SH.STA.POIS.P5]
Mortality rate attributed to unintentional poisoning, female (per 100,000 female population)	[SH.STA.POIS.P5.FE]
Mortality rate attributed to unintentional poisoning, male (per 100,000 male population)	[SH.STA.POIS.P5.MA]
Mortality rate attributed to unsafe water, unsafe sanitation and lack of hygiene (per 100,000 population)	[SH.STA.WASH.P5]
Mortality rate, adult, female (per 1,000 female adults)	[SP.DYN.AMRT.FE]
Mortality rate, adult, male (per 1,000 male adults)	[SP.DYN.AMRT.MA]
Mortality rate, infant (per 1,000 live births)	[SP.DYN.IMRT.IN]
Mortality rate, infant, female (per 1,000 live births)	[SP.DYN.IMRT.FE.IN]
Mortality rate, infant, male (per 1,000 live births)	[SP.DYN.IMRT.MA.IN]
Mortality rate, neonatal (per 1,000 live births)	[SH.DYN.NMRT]
Mortality rate, under-5 (per 1,000)	[SH.DYN.MORT]
Mortality rate, under-5, female (per 1,000)	[SH.DYN.MORT.FE]
Mortality rate, under-5, male (per 1,000)	[SH.DYN.MORT.MA]
Net migration	[SM.POP.NETM]
Newborns protected against tetanus (%)	[SH.VAC.TTNS.ZS]
Number of deaths ages 5-14 years	[SH.DTH.0514]
Number of infant deaths	[SH.DTH.IMRT]
Number of maternal deaths	[SH.MMR.DTHS]
Number of neonatal deaths	[SH.DTH.NMRT]
Number of people pushed below the $1.90 ($ 2011 PPP) poverty line by out-of-pocket health care expenditure	[SH.UHC.NOP1.TO]
Number of people pushed below the $3.10 ($ 2011 PPP) poverty line by out-of-pocket health care expenditure	[SH.UHC.NOP2.TO]
Number of people spending more than 10% of household consumption or income on out-of-pocket health care expenditure	[SH.UHC.OOPC.10.TO]
Number of people spending more than 25% of household consumption or income on out-of-pocket health care expenditure	[SH.UHC.OOPC.25.TO]
Number of people who are undernourished	[SN.ITK.DEFC]
Number of surgical procedures (per 100,000 population)	[SH.SGR.PROC.P5]
Number of under-five deaths	[SH.DTH.MORT]
Number of weeks of maternity leave	[SH.MMR.LEVE]
Nurses and midwives (per 1,000 people)	[SH.MED.NUMW.P3]
Out-of-pocket expenditure (% of current health expenditure)	[SH.XPD.OOPC.CH.ZS]
Out-of-pocket expenditure per capita (current US$)	[SH.XPD.OOPC.PC.CD]
Out-of-pocket expenditure per capita, PPP (current international $)	[SH.XPD.OOPC.PP.CD]
People practicing open defecation (% of population)	[SH.STA.ODFC.ZS]
People practicing open defecation, rural (% of rural population)	[SH.STA.ODFC.RU.ZS]
People practicing open defecation, urban (% of urban population)	[SH.STA.ODFC.UR.ZS]
People using at least basic drinking water services (% of population)	[SH.H2O.BASW.ZS]
People using at least basic drinking water services, rural (% of rural population)	[SH.H2O.BASW.RU.ZS]
People using at least basic drinking water services, urban (% of urban population)	[SH.H2O.BASW.UR.ZS]
People using at least basic sanitation services (% of population)	[SH.STA.BASS.ZS]
People using at least basic sanitation services, rural (% of rural population)	[SH.STA.BASS.RU.ZS]
People using at least basic sanitation services, urban (% of urban population)	[SH.STA.BASS.UR.ZS]
People using safely managed drinking water services (% of population)	[SH.H2O.SMDW.ZS]

People using safely managed drinking water services, rural (% of rural population)	[SH.H2O.SMDW.RU.ZS]
People using safely managed drinking water services, urban (% of urban population)	[SH.H2O.SMDW.UR.ZS]
People using safely managed sanitation services (% of population)	[SH.STA.SMSS.ZS]
People using safely managed sanitation services, rural (% of rural population)	[SH.STA.SMSS.RU.ZS]
People using safely managed sanitation services, urban (% of urban population)	[SH.STA.SMSS.UR.ZS]
People with basic handwashing facilities including soap and water (% of population)	[SH.STA.HYGN.ZS]
People with basic handwashing facilities including soap and water, rural (% of rural population)	[SH.STA.HYGN.RU.ZS]
People with basic handwashing facilities including soap and water, urban (% of urban population)	[SH.STA.HYGN.UR.ZS]
Physicians (per 1,000 people)	[SH.MED.PHYS.ZS]
Population ages 00-04, female (% of female population)	[SP.POP.0004.FE.5Y]
Population ages 00-04, male (% of male population)	[SP.POP.0004.MA.5Y]
Population ages 00-14 (% of total)	[SP.POP.0014.TO.ZS]
Population ages 00-14, female (% of total)	[SP.POP.0014.FE.ZS]
Population ages 00-14, male (% of total)	[SP.POP.0014.MA.ZS]
Population ages 00-14, total	[SP.POP.0014.TO]
Population ages 0-14, female	[SP.POP.0014.FE.IN]
Population ages 0-14, male	[SP.POP.0014.MA.IN]
Population ages 05-09, female (% of female population)	[SP.POP.0509.FE.5Y]
Population ages 05-09, male (% of male population)	[SP.POP.0509.MA.5Y]
Population ages 10-14, female (% of female population)	[SP.POP.1014.FE.5Y]
Population ages 15-19, female (% of female population)	[SP.POP.1519.FE.5Y]
Population ages 15-19, male (% of male population)	[SP.POP.1519.MA.5Y]
Population ages 15-64 (% of total)	[SP.POP.1564.TO.ZS]
Population ages 15-64, female	[SP.POP.1564.FE.IN]
Population ages 15-64, female (% of total)	[SP.POP.1564.FE.ZS]
Population ages 15-64, male	[SP.POP.1564.MA.IN]
Population ages 15-64, male (% of total)	[SP.POP.1564.MA.ZS]
Population ages 15-64, total	[SP.POP.1564.TO]
Population ages 20-24, female (% of female population)	[SP.POP.2024.FE.5Y]
Population ages 20-24, male (% of male population)	[SP.POP.2024.MA.5Y]
Population ages 25-29, female (% of female population)	[SP.POP.2529.FE.5Y]
Population ages 25-29, male (% of male population)	[SP.POP.2529.MA.5Y]
Population ages 30-34, female (% of female population)	[SP.POP.3034.FE.5Y]
Population ages 30-34, male (% of male population)	[SP.POP.3034.MA.5Y]
Population ages 35-39, female (% of female population)	[SP.POP.3539.FE.5Y]
Population ages 35-39, male (% of male population)	[SP.POP.3539.MA.5Y]
Population ages 40-44, female (% of female population)	[SP.POP.4044.FE.5Y]
Population ages 40-44, male (% of male population)	[SP.POP.4044.MA.5Y]
Population ages 45-49, female (% of female population)	[SP.POP.4549.FE.5Y]
Population ages 45-49, male (% of male population)	[SP.POP.4549.MA.5Y]
Population ages 50-54, female (% of female population)	[SP.POP.5054.FE.5Y]
Population ages 50-54, male (% of male population)	[SP.POP.5054.MA.5Y]
Population ages 55-59, female (% of female population)	[SP.POP.5559.FE.5Y]
Population ages 55-59, male (% of male population)	[SP.POP.5559.MA.5Y]
Population ages 60-64, female (% of female population)	[SP.POP.6064.FE.5Y]
Population ages 60-64, male (% of male population)	[SP.POP.6064.MA.5Y]
Population ages 65 and above (% of total)	[SP.POP.65UP.TO.ZS]
Population ages 65 and above, female	[SP.POP.65UP.FE.IN]
Population ages 65 and above, female (% of total)	[SP.POP.65UP.FE.ZS]
Population ages 65 and above, male	[SP.POP.65UP.MA.IN]

Population ages 65 and above, male (% of total)	[SP.POP.65UP.MA.ZS]
Population ages 65 and above, total	[SP.POP.65UP.TO]
Population ages 65-69, female (% of female population)	[SP.POP.6569.FE.5Y]
Population ages 65-69, male (% of male population)	[SP.POP.6569.MA.5Y]
Population ages 70-74, female (% of female population)	[SP.POP.7074.FE.5Y]
Population ages 70-74, male (% of male population)	[SP.POP.7074.MA.5Y]
Population ages 75-79, female (% of female population)	[SP.POP.7579.FE.5Y]
Population ages 75-79, male (% of male population)	[SP.POP.7579.MA.5Y]
Population ages 80 and older, female (% of female population)	[SP.POP.80UP.FE.5Y]
Population ages 80 and older, male (% of male population)	[SP.POP.80UP.MA.5Y]
Population ages10-14, male (% of male population)	[SP.POP.1014.MA.5Y]
Population growth (annual %)	[SP.POP.GROW]
Population, female	[SP.POP.TOTL.FE.IN]
Population, female (% of total)	[SP.POP.TOTL.FE.ZS]
Population, male	[SP.POP.TOTL.MA.IN]
Population, male (% of total)	[SP.POP.TOTL.MA.ZS]
Population, total	[SP.POP.TOTL]
Postnatal care coverage (% mothers)	[SH.STA.PNVC.ZS]
Poverty headcount ratio at national poverty line (% of population)	[SI.POV.NAHC]
Pregnant women receiving prenatal care (%)	[SH.STA.ANVC.ZS]
Pregnant women receiving prenatal care of at least four visits (% of pregnant women)	[SH.STA.ANV4.ZS]
Prevalence of anemia among children (% of children under 5)	[SH.ANM.CHLD.ZS]
Prevalence of anemia among non-pregnant women (% of women ages 15-49)	[SH.ANM.NPRG.ZS]
Prevalence of anemia among pregnant women (%)	[SH.PRG.ANEM]
Prevalence of anemia among women of reproductive age (% of women ages 15-49)	[SH.ANM.ALLW.ZS]
Prevalence of HIV, female (% ages 15-24)	[SH.HIV.1524.FE.ZS]
Prevalence of HIV, male (% ages 15-24)	[SH.HIV.1524.MA.ZS]
Prevalence of HIV, total (% of population ages 15-49)	[SH.DYN.AIDS.ZS]
Prevalence of overweight (% of adults)	[SH.STA.OWAD.ZS]
Prevalence of overweight (% of children under 5)	[SH.STA.OWGH.ZS]
Prevalence of overweight, female (% of children under 5)	[SH.STA.OWGH.FE.ZS]
Prevalence of overweight, female (% of female adults)	[SH.STA.OWAD.FE.ZS]
Prevalence of overweight, male (% of children under 5)	[SH.STA.OWGH.MA.ZS]
Prevalence of overweight, male (% of male adults)	[SH.STA.OWAD.MA.ZS]
Prevalence of severe wasting, weight for height (% of children under 5)	[SH.SVR.WAST.ZS]
Prevalence of severe wasting, weight for height, female (% of children under 5)	[SH.SVR.WAST.FE.ZS]
Prevalence of severe wasting, weight for height, male (% of children under 5)	[SH.SVR.WAST.MA.ZS]
Prevalence of syphilis (% of women attending antenatal care)	[SH.PRG.SYPH.ZS]
Prevalence of undernourishment (% of population)	[SN.ITK.DEFC.ZS]
Prevalence of wasting (% of children under 5)	[SH.STA.WAST.ZS]
Prevalence of wasting, female (% of children under 5)	[SH.STA.WAST.FE.ZS]
Prevalence of wasting, male (% of children under 5)	[SH.STA.WAST.MA.ZS]
Primary completion rate, female (% of relevant age group)	[SE.PRM.CMPT.FE.ZS]
Primary completion rate, male (% of relevant age group)	[SE.PRM.CMPT.MA.ZS]
Primary completion rate, total (% of relevant age group)	[SE.PRM.CMPT.ZS]
Probability of dying at age 5-14 years (per 1,000 children age 5)	[SH.DYN.0514]
Proportion of population pushed below the $1.90 ($ 2011 PPP) poverty line by out-of-pocket health care expenditure (%)	[SH.UHC.NOP1.ZS]
Proportion of population pushed below the $3.10 ($ 2011 PPP) poverty line by out-of-pocket health care expenditure (%)	[SH.UHC.NOP2.ZS]
Proportion of population spending more than 10% of household consumption or income on out-of-pocket health care expenditure (%)	[SH.UHC.OOPC.10.ZS]
Proportion of population spending more than 25% of household consumption or income on out-of-pocket health care expenditure (%)	[SH.UHC.OOPC.25.ZS]

Public spending on education, total (% of GDP)	[SE.XPD.TOTL.GD.ZS]
Ratio of school attendance of orphans to school attendance of non-orphans ages 10-14	[SE.ENR.ORPH]
Ratio of young literate females to males (% ages 15-24)	[SE.ADT.1524.LT.FM.ZS]
Risk of catastrophic expenditure for surgical care (% of people at risk)	[SH.SGR.CRSK.ZS]
Risk of impoverishing expenditure for surgical care (% of people at risk)	[SH.SGR.IRSK.ZS]
Rural population	[SP.RUR.TOTL]
Rural population (% of total population)	[SP.RUR.TOTL.ZS]
Rural population growth (annual %)	[SP.RUR.TOTL.ZG]
Rural poverty headcount ratio at national poverty lines (% of rural population)	[SI.POV.RUHC]
School enrollment, primary (% gross)	[SE.PRM.ENRR]
School enrollment, primary (% net)	[SE.PRM.NENR]
School enrollment, primary, female (% gross)	[SE.PRM.ENRR.FE]
School enrollment, primary, female (% net)	[SE.PRM.NENR.FE]
School enrollment, primary, male (% gross)	[SE.PRM.ENRR.MA]
School enrollment, primary, male (% net)	[SE.PRM.NENR.MA]
School enrollment, secondary (% gross)	[SE.SEC.ENRR]
School enrollment, secondary (% net)	[SE.SEC.NENR]
School enrollment, secondary, female (% gross)	[SE.SEC.ENRR.FE]
School enrollment, secondary, female (% net)	[SE.SEC.NENR.FE]
School enrollment, secondary, male (% gross)	[SE.SEC.ENRR.MA]
School enrollment, secondary, male (% net)	[SE.SEC.NENR.MA]
School enrollment, tertiary (% gross)	[SE.TER.ENRR]
School enrollment, tertiary, female (% gross)	[SE.TER.ENRR.FE]
Sex ratio at birth (male births per female births)	[SP.POP.BRTH.MF]
Share of women employed in the nonagricultural sector (% of total nonagricultural employment)	[SL.EMP.INSV.FE.ZS]
Smoking prevalence, females (% of adults)	[SH.PRV.SMOK.FE]
Smoking prevalence, males (% of adults)	[SH.PRV.SMOK.MA]
Smoking prevalence, total, ages 15+	[SH.PRV.SMOK]
Specialist surgical workforce (per 100,000 population)	[SH.MED.SAOP.P5]
Suicide mortality rate (per 100,000 population)	[SH.STA.SUIC.P5]
Suicide mortality rate, female (per 100,000 female population)	[SH.STA.SUIC.FE.P5]
Suicide mortality rate, male (per 100,000 male population)	[SH.STA.SUIC.MA.P5]
Survival to age 65, female (% of cohort)	[SP.DYN.TO65.FE.ZS]
Survival to age 65, male (% of cohort)	[SP.DYN.TO65.MA.ZS]
Teenage mothers (% of women ages 15-19 who have had children or are currently pregnant)	[SP.MTR.1519.ZS]
Total alcohol consumption per capita (liters of pure alcohol, projected estimates, 15+ years of age)	[SH.ALC.PCAP.LI]
Total alcohol consumption per capita, female (liters of pure alcohol, projected estimates, female 15+ years of age)	[SH.ALC.PCAP.FE.LI]
Total alcohol consumption per capita, male (liters of pure alcohol, projected estimates, male 15+ years of age)	[SH.ALC.PCAP.MA.LI]
Tuberculosis case detection rate (%, all forms)	[SH.TBS.DTEC.ZS]
Tuberculosis death rate (per 100,000 people)	[SH.TBS.MORT]
Tuberculosis treatment success rate (% of new cases)	[SH.TBS.CURE.ZS]
UHC service coverage index	[SH.UHC.SRVS.CV.XD]
Unemployment, female (% of female labor force)	[SL.UEM.TOTL.FE.ZS]
Unemployment, male (% of male labor force)	[SL.UEM.TOTL.MA.ZS]
Unemployment, total (% of total labor force)	[SL.UEM.TOTL.ZS]
Unmet need for contraception (% of married women ages 15-49)	[SP.UWT.TFRT]
Urban population	[SP.URB.TOTL]
Urban population (% of total)	[SP.URB.TOTL.IN.ZS]
Urban population growth (annual %)	[SP.URB.GROW]
Urban poverty headcount ratio at national poverty lines (% of urban population)	[SI.POV.URHC]

Use of insecticide-treated bed nets (% of under-5 population)	[SH.MLR.NETS.ZS]
Use of Intermittent Preventive Treatment of malaria, 2+ doses of SP/Fansidar (% of pregnant women)	[SH.MLR.SPF2.ZS]
Vitamin A supplementation coverage rate (% of children ages 6-59 months)	[SN.ITK.VITA.ZS]
Wanted fertility rate (births per woman)	[SP.DYN.WFRT]
Women who were first married by age 15 (% of women ages 20-24)	[SP.M15.2024.FE.ZS]
Women who were first married by age 18 (% of women ages 20-24)	[SP.M18.2024.FE.ZS]
Women's share of population ages 15+ living with HIV (%)	[SH.DYN.AIDS.FE.ZS]

Included variables are aggregated as Health, Nutrition, and Population Statistics by the World Bank (available from: https://databank.worldbank.org/source/health-nutrition-and-population-statistics). We used national-level aggregations of these indicators as of 2014 (the year for which the most complete data were available) as a basis for extrapolating incidence rate estimates to countries without DHS/MICS data via 10-fold cross-validation (as described in the **Methods**). We illustrate out-of-sample validation of the extrapolation approach based on these variables in **Extended data figure 6**.

Table S11: Country-specific estimates of the incidence of ARI and antibiotic-treated ARI among children ages 24-59 months preventable by 10- and 13-valent pneumococcal conjugate vaccines.

Country	Population ages 24-59 months	ARI attributable to all causes			ARI attributable to PCV10/13-type *Streptococcus pneumoniae*		
	Children (in 10,000s)	All cases, per 100 (95% CI)	Proportion treated with antibiotics, % (95% CI)	Antibiotic-treated cases, per 100 (95% CI)	Antibiotic-treated cases, per 100 (95% CI)		Antibiotic-treated cases preventable by vaccine direct effects, per 100 (95% CI)
					IPD probe estimate	*AOM probe estimate*	
**African Region**							
Algeria	275	104.2 (95.5-113.2)	46 (38-56.4)	47.9 (39.3-59.1)	11.8 (2-27.3)	17.1 (2.9-38.6)	9.5 (1.6-21.2)
Angola	300.8	120.3 (113.2-127.8)	35.1 (27-43.5)	42.3 (32.3-52.7)	10.4 (1.8-23.4)	15 (2.6-33.6)	8.3 (1.5-18.5)
Benin	101.4	167.4 (156.4-179)	40.4 (30.8-50)	67.6 (51.5-84.7)	16.7 (2.9-38)	24.1 (4.1-54.3)	13.3 (2.3-29.5)
Botswana	15	110.2 (100.9-120.4)	41.3 (34.3-49.4)	45.5 (37.5-55)	11.3 (2-25.5)	16.3 (2.9-36.7)	9 (1.7-20.2)
Burkina Faso	185.1	178.9 (166.6-192.1)	40 (31.3-48.5)	71.4 (55.5-87.8)	17.6 (3-39.7)	25.4 (4.4-57.1)	14.1 (2.4-31.2)
Burundi	107.4	194.8 (177.6-212.2)	42.7 (37-48.3)	83.1 (70.7-96.7)	20.5 (3.7-45.7)	29.7 (5.3-65.5)	16.5 (3-36)
Cabo Verde	3.3	114.6 (104.4-125.7)	45.5 (37.8-54.7)	52.1 (43.5-62.6)	12.9 (2.2-29)	18.6 (3.2-41.9)	10.3 (1.8-23)
Cameroon	219.1	151.2 (142.9-160)	42.3 (36-48.7)	63.8 (53.7-74.5)	15.8 (2.8-35.4)	22.9 (4-51.1)	12.6 (2.2-27.8)
Cent. African Rep.	42.6	169.1 (152.4-186.2)	38.5 (33.3-44.4)	65.1 (55.4-75.8)	16.1 (2.8-35.1)	23.3 (4.2-50.5)	12.9 (2.3-27.9)
Chad	150.4	171.2 (160.5-182.5)	39.7 (35.6-44.1)	67.9 (60-76.9)	16.8 (2.9-37.3)	24.4 (4.2-53.8)	13.5 (2.4-29.2)
Comoros	6.9	167.8 (155.5-180.3)	40.5 (25.6-55.3)	67.6 (42.9-93.6)	16.5 (2.8-38.4)	23.8 (4.1-55.2)	13.1 (2.3-30.4)
Congo, Rep.	47.9	137.7 (130.5-145)	40.5 (33.5-47.3)	55.7 (46-65.5)	13.7 (2.3-30.9)	19.9 (3.3-44.7)	11 (1.9-24.3)
Congo, Dem. Rep.	814.3	179.1 (165.8-192.9)	43.6 (38.7-48.2)	77.9 (68.4-88.1)	19.3 (3.4-42.9)	27.9 (4.9-61.7)	15.4 (2.8-33.9)
Cote d'Ivoire	218.2	154.1 (145-163.4)	37.7 (29.9-46)	58 (45.6-71.6)	14.3 (2.5-32.1)	20.7 (3.6-46.1)	11.4 (2-25.2)
Equatorial Guinea	10.2	109.9 (98.2-123.4)	42 (33.3-52.4)	46.3 (36.7-57.4)	11.3 (2-25.9)	16.4 (3-37.3)	9.1 (1.7-20.4)
Eritrea	44.7	159.4 (137.2-182.5)	45.3 (37.7-55.2)	72.6 (58.9-87.7)	18 (3.1-40)	26 (4.6-57.4)	14.4 (2.6-31.7)
Eswatini	10.6	130.7 (121.7-140.1)	37.7 (29-47.7)	49.6 (37.3-62.7)	12.2 (2.1-27.6)	17.6 (3.1-39.9)	9.8 (1.7-22)
Ethiopia	870.8	170.8 (159.9-182.3)	37 (30.3-44.2)	63.5 (51.6-76.5)	15.7 (2.8-34.9)	22.7 (4.1-50.3)	12.6 (2.2-27.5)
Gabon	15.5	99.3 (91.8-107.1)	35.4 (28.8-42)	35 (28.1-42.3)	8.6 (1.5-19.1)	12.5 (2.1-27.6)	6.9 (1.2-15.2)
Gambia, The	20.4	170.2 (158.5-182)	42.1 (34.8-49.7)	71.6 (58.5-85.5)	17.7 (3.1-39.6)	25.7 (4.5-56.7)	14.2 (2.6-31.2)
Ghana	235.8	143.4 (135.4-151.7)	43.9 (33.7-54.1)	62.9 (48.3-78.8)	15.4 (2.7-34.9)	22.3 (3.9-50.2)	12.3 (2.2-27.8)
Guinea	114	173.4 (161.5-185.7)	39.4 (32.2-46.9)	68.2 (55.3-82.3)	16.9 (2.9-37.7)	24.4 (4.3-54)	13.6 (2.3-29.6)
Guinea-Bissau	16.6	128.5 (118.5-138.6)	41.3 (32.1-50.5)	52.9 (40.8-65.9)	13 (2.2-29.4)	18.7 (3.2-42.4)	10.3 (1.8-23.5)
Kenya	419.3	148.4 (140.8-156.1)	38.8 (35.2-42.6)	57.6 (51.7-64)	14.3 (2.5-31.6)	20.7 (3.6-45.7)	11.5 (2-25.2)
Lesotho	16.3	144.1 (134.6-154)	40.8 (29.6-52.8)	59.4 (42.8-76.4)	14.5 (2.6-33.2)	20.9 (3.8-47.8)	11.5 (2.1-26.3)
Liberia	41.4	168.3 (156.5-180.5)	41.9 (35.7-48.7)	70.4 (59.1-83.1)	17.6 (3.1-38.8)	25.4 (4.5-55.3)	14.1 (2.5-30.6)
Madagascar	217.6	166.3 (152.5-180.5)	46.7 (39.8-54.5)	77.7 (65.5-91.7)	19.3 (3.4-42.8)	27.9 (4.9-61.7)	15.4 (2.8-33.6)
Malawi	173.1	191.6 (174.8-208.8)	44.9 (39.6-50.4)	86 (74.1-99)	21.4 (3.7-47.4)	30.8 (5.4-68)	17.1 (3.1-37.2)
Mali	191.5	172.3 (160.9-183.8)	33.7 (24.1-44.4)	58.7 (41.6-77.3)	14.4 (2.4-33.1)	20.7 (3.5-47.9)	11.4 (2-26.4)
Mauritania	37.3	130.5 (122.7-138.5)	38.8 (32.1-45.2)	50.6 (41.4-59.7)	12.5 (2.1-28)	18.1 (3-39.8)	10 (1.8-22.2)
Mauritius	4.2	105.3 (96.2-115.8)	43.6 (35.4-53.1)	45.9 (37-56.2)	11.3 (2-25.7)	16.4 (2.8-36.9)	9.1 (1.6-20)
Mozambique	282.1	186.3 (171.2-202.1)	41.9 (34.2-50.3)	78.2 (62.4-95)	19.4 (3.4-42.8)	28.1 (4.9-61.8)	15.5 (2.8-34.1)
Namibia	19.3	115.4 (107.7-123.6)	35.3 (27.6-43.9)	40.7 (31.4-51)	10 (1.7-22.7)	14.6 (2.5-32.6)	8.1 (1.4-18)
Niger	232.8	191.4 (176.1-207.5)	40.6 (33.3-47.8)	77.7 (63.4-92.7)	19.1 (3.2-43.4)	27.7 (4.7-62.1)	15.3 (2.7-34)
Nigeria	1804.2	147.2 (139.8-154.7)	43 (36.3-50)	63.2 (52.9-74.4)	15.7 (2.7-34.5)	22.6 (3.9-49.5)	12.5 (2.2-27.3)
Rwanda	103.6	173.7 (161.7-186)	41.9 (34.6-49)	72.6 (59.9-86.3)	18 (3.1-40.5)	26 (4.5-58.4)	14.3 (2.5-31.9)
Sao Tome and Principe	1.8	148.1 (137.2-159.5)	43.5 (32.9-54.1)	63.9 (47.9-81.9)	15.7 (2.6-36.3)	22.7 (3.7-52.2)	12.6 (2.1-28.3)
Senegal	145.7	153.5 (144.8-162.6)	38.9 (32.2-45.8)	59.7 (49.1-70.9)	14.7 (2.5-32.8)	21.3 (3.6-46.9)	11.7 (2-25.9)

Sierra Leone	67.3	183.8 (169.7-197.7)	41.3 (35.8-46.7)	75.7 (64.9-87.2)	18.7 (3.3-41.5)	27.2 (4.8-59.7)	15 (2.7-32.8)
South Africa	336.1	99.5 (91.9-107.2)	35.4 (21.7-50)	35.1 (22-50.3)	8.6 (1.4-20.5)	12.4 (2-29.5)	6.9 (1.1-16)
South Sudan	110	157.5 (139.2-178.9)	40.7 (33.5-50.7)	64.9 (52.3-77.1)	16 (2.7-36)	23.2 (4-51.8)	12.8 (2.3-28.4)
Tanzania, United Rep.	547.9	165.6 (155.3-176.2)	40.7 (32-49.6)	67.4 (52.7-82.8)	16.6 (2.9-37.3)	24 (4.2-53.8)	13.3 (2.4-29.3)
Togo	68.6	173.7 (161.3-186.6)	40.9 (32.5-50)	71.3 (56.1-87.9)	17.5 (3-40.2)	25.3 (4.3-57.4)	14.1 (2.5-31.7)
Uganda	436.5	170.9 (159.6-182.9)	49.2 (43.7-55.5)	84.1 (73.3-97.2)	20.9 (3.8-46.9)	30.3 (5.5-67.6)	16.8 (3-36.8)
Zambia	160.1	151.6 (143.7-160.1)	39.1 (32.9-45.6)	59.2 (49.6-69.8)	14.7 (2.5-32.6)	21.3 (3.7-47.1)	11.8 (2.1-25.7)
Zimbabwe	146.6	144.2 (135.9-152.9)	41.4 (32.4-51.4)	59.9 (46.4-74.4)	14.7 (2.7-33.2)	21.3 (3.9-48)	11.8 (2.1-26.3)
**Region of the Americas**							
Belize	2.3	117.3 (109.3-126)	37.7 (24.6-49.2)	43.6 (29.1-59)	10.7 (1.9-25)	15.5 (2.7-36)	8.5 (1.5-19.6)
Bolivia, Plurinational State	70.7	118.9 (112-126.3)	69.4 (59.9-79.4)	82.5 (70.4-95.7)	20.5 (3.6-45.3)	29.7 (5.2-65.4)	16.4 (2.9-36.1)
Brazil	884.7	103.4 (95.4-112.3)	42.4 (35.6-50.1)	43.9 (36.4-52.1)	10.9 (1.9-24.3)	15.7 (2.7-34.9)	8.7 (1.6-19.2)
Colombia	225.6	98.5 (92-105)	38 (33.4-42.6)	37.4 (32.4-42.6)	9.3 (1.6-20.5)	13.4 (2.3-29.5)	7.4 (1.3-16.2)
Costa Rica	21.4	101.4 (93.1-110.2)	41.9 (34.8-50)	42.5 (35.2-51)	10.5 (1.8-23.8)	15.1 (2.6-34.4)	8.4 (1.5-18.9)
Cuba	36.9	102.7 (95.4-110.2)	31.9 (25.6-38.4)	32.7 (26-39.9)	8.1 (1.4-18)	11.7 (2.1-26.1)	6.5 (1.1-14.4)
Dominica	0.4	105.1 (95.8-115.1)	43.3 (35.5-51.8)	45.5 (36.9-55.4)	11.2 (1.9-25.5)	16.2 (2.8-36.8)	9 (1.6-20)
Dominican Rep.	63.8	108.8 (101.9-116)	32.2 (28.7-36)	35 (30.6-39.8)	8.7 (1.5-19.1)	12.6 (2.2-27.7)	7 (1.2-15.2)
Ecuador	96.5	103 (95.2-111.7)	42.6 (36.6-49.6)	44 (37.3-51.6)	10.9 (1.9-24.4)	15.8 (2.8-35.1)	8.7 (1.6-19.3)
El Salvador	34.9	109.7 (102.8-116.9)	35.1 (29.8-40.6)	38.5 (32.3-45.2)	9.6 (1.7-21.1)	13.9 (2.4-30.4)	7.7 (1.4-16.7)
Grenada	0.6	105.8 (96-115.9)	45.1 (35.5-55.3)	47.6 (37.4-59.5)	11.7 (2-26.2)	17 (2.9-37.6)	9.4 (1.6-20.7)
Guatemala	117.4	127.7 (120.2-135.4)	38.1 (33.9-42.4)	48.6 (42.8-55.1)	12.1 (2-26.5)	17.5 (3-38.3)	9.7 (1.7-21)
Guyana	4.4	114.9 (106-124)	39.5 (28.2-51.3)	45.1 (32.4-58.7)	11.1 (1.9-25.5)	16 (2.8-36.7)	8.9 (1.6-19.9)
Haiti	74.2	163.9 (152.9-175)	41.5 (35.5-47.9)	68.1 (57.3-79.8)	17 (2.9-37.3)	24.5 (4.2-53.9)	13.5 (2.4-29.8)
Honduras	57.6	130.7 (123.7-138)	41 (36.8-45.4)	53.6 (47.6-60)	13.3 (2.3-29.5)	19.3 (3.3-42.5)	10.7 (1.9-23.3)
Jamaica	12.6	105.4 (96.7-114.7)	41 (34.5-48.1)	43.1 (36.3-51)	10.6 (1.9-24.1)	15.3 (2.8-34.5)	8.5 (1.6-18.8)
Mexico	685.7	102.2 (94.7-110.1)	45.4 (35.3-56.3)	46.5 (35.9-58.4)	11.5 (1.9-26.1)	16.6 (2.8-37.5)	9.2 (1.6-20.5)
Nicaragua	36.8	118.2 (109.2-127.9)	45.9 (37.7-55.9)	54.3 (44.6-66.3)	13.5 (2.4-29.9)	19.6 (3.5-42.7)	10.8 (1.9-23.4)
Paraguay	40.6	100.8 (93.9-108)	57.3 (49.5-65.4)	57.7 (49.1-66.8)	14.3 (2.5-31.3)	20.7 (3.6-45.4)	11.5 (2-25.1)
Peru	179.5	104.5 (97.7-111.6)	59.3 (51-67.5)	62 (52.5-71.9)	15.4 (2.6-33.4)	22.2 (3.8-48.1)	12.3 (2.1-26.7)
St. Lucia	0.6	105.4 (95.6-115.8)	42.7 (34.5-51.2)	45 (36.1-54.4)	11.1 (2-25.1)	16 (2.9-36.2)	8.8 (1.6-19.8)
St. Vincent and the Grenadines	0.5	107 (97.2-116.9)	42 (34.7-50.2)	45 (36.7-54.1)	11.1 (1.9-25.3)	16.1 (2.8-36.3)	8.9 (1.6-19.9)
Suriname	3	106.7 (97.4-117.6)	42.7 (35.4-51.5)	45.6 (37.4-55.3)	11.3 (2-25.2)	16.3 (2.9-36.4)	9 (1.6-19.9)
Venezuela, Bolivarian Rep.	178.4	103.5 (95.3-112.5)	44.7 (36.7-53.8)	46.3 (37.4-56)	11.4 (2-25.7)	16.5 (2.8-36.8)	9.2 (1.6-20)
**Eastern Mediterranean Region**							
Afghanistan	316.6	173.5 (160.9-186.1)	61.9 (58.5-65.3)	107.3 (98.3-117.4)	26.8 (4.6-58.7)	38.8 (6.7-84.6)	21.3 (3.8-47.1)
Djibouti	5.9	124.1 (112.2-137.7)	45.2 (36.8-54.5)	56 (46.1-67.4)	13.8 (2.5-31.2)	20.1 (3.7-44.6)	11 (2-24.3)
Egypt, Arab Rep.	694.3	126.2 (120.1-132.5)	58.3 (54.8-62)	73.6 (68-79.6)	18.3 (3.1-39.9)	26.5 (4.5-57.5)	14.7 (2.5-31.9)
Iran, Islamic Rep.	417.8	103.3 (95.2-112.1)	47 (39.6-55.3)	48.5 (40.7-57.5)	12 (2.1-27.2)	17.3 (3-39.2)	9.6 (1.7-21.3)
Iraq	327.5	113.5 (103.7-124.8)	47.2 (38.9-57.1)	53.7 (44.1-65.2)	13.2 (2.3-29.6)	19.1 (3.3-42.6)	10.6 (1.9-23.5)
Jordan	71.1	97.3 (91.3-103.6)	55.9 (50.1-61.7)	54.4 (47.8-61.3)	13.4 (2.4-29.6)	19.5 (3.4-42.4)	10.8 (1.9-23.5)
Lebanon	26.4	100.6 (91.4-110.5)	48.1 (40.2-57.2)	48.4 (40.4-57.5)	11.9 (2.1-26.8)	17.2 (3-38.4)	9.5 (1.7-21.3)
Libyan Arab Rep.	37.5	109 (97.1-126.4)	47.3 (38-58.6)	51.9 (41.9-64.3)	12.8 (2.2-29.3)	18.6 (3.2-42)	10.2 (1.8-22.6)
Morocco	201.9	115.9 (106.2-125.8)	49.1 (40.3-59.3)	57 (47.3-68.3)	13.9 (2.4-31.3)	20.2 (3.5-45.2)	11.2 (1.9-24.9)
Pakistan	1458.8	141.3 (134.1-148.9)	53.1 (49-57.1)	75 (68.2-82.3)	18.8 (3.2-40.5)	27.1 (4.6-58.7)	15 (2.6-32.6)
Palestinian Terr.	41	118.9 (111.9-126)	53.5 (48.8-58.5)	63.6 (57-70.8)	15.8 (2.7-34.4)	22.9 (4-49.6)	12.6 (2.2-27.6)
Somalia	147.9	138.4 (120.9-164.5)	43.5 (35.4-53.4)	60.7 (50.2-72.4)	15 (2.6-33.3)	21.7 (3.8-48.1)	12 (2.1-26.5)
Sudan	344.8	144.8 (131.5-158)	43.2 (36.4-50.8)	62.4 (52.8-73.2)	15.5 (2.7-34)	22.4 (4-48.9)	12.4 (2.2-26.9)
Tunisia	58.8	103.9 (95.4-113.2)	48.4 (40.2-57.8)	50.2 (41.8-60.4)	12.4 (2.1-28)	17.9 (3.1-40.2)	9.9 (1.7-21.9)

Yemen, Rep.	236.3	142.2 (134.1-150.3)	62.3 (58.4-66.1)	88.6 (81.5-95.7)	22 (3.8-48.5)	31.8 (5.5-69.8)	17.7 (3-38.2)
**European Region**							
Albania	9.7	108.9 (100.9-117.5)	48.6 (29.7-67.6)	52.6 (31.7-75.3)	12.7 (2.1-30.9)	18.4 (3.1-44.2)	10.2 (1.7-24.2)
Armenia	12.6	97.5 (89.1-106.7)	57.9 (36.8-78.9)	57 (34.5-79.5)	13.7 (2.3-32.3)	19.8 (3.3-46.6)	10.9 (1.8-25.5)
Azerbaijan	49.5	104.4 (96.1-113.1)	40 (24-58.5)	42.1 (24.7-61.7)	10.2 (1.7-25.4)	14.8 (2.5-36.4)	8.1 (1.4-19.8)
Belarus	34.2	101.8 (93.1-110.6)	41.7 (33.8-50.1)	42.5 (34.2-51.4)	10.5 (1.8-23.6)	15.2 (2.6-33.9)	8.4 (1.5-18.5)
Bosnia and Herzegovina	10.1	101.4 (92.1-111.9)	43.4 (35.4-53)	44.1 (35.3-54.5)	10.9 (1.9-24.6)	15.7 (2.7-35.5)	8.7 (1.5-19.2)
Bulgaria	21.1	100.6 (92-109.6)	42.7 (34.2-51.5)	42.9 (34.2-52.6)	10.6 (1.8-24)	15.3 (2.6-34.4)	8.4 (1.5-18.8)
Georgia	16.6	104.3 (95-113.7)	43.3 (35-53.4)	45.1 (36.2-56)	11.1 (1.9-25.4)	16.1 (2.7-36.5)	8.9 (1.5-19.9)
Kazakhstan	118.2	102.6 (94-111.4)	44.9 (36.3-53.7)	46 (37-55.5)	11.3 (1.9-25.6)	16.4 (2.8-36.8)	9.1 (1.6-20.1)
Kosovo	9	107.3 (99.4-115.8)	49.5 (34.7-66.3)	53.1 (37.3-71.1)	13.2 (2.2-30.4)	19 (3.2-43.7)	10.4 (1.8-24)
Kyrgyzstan	43.4	125 (116.7-133.8)	43.5 (21.7-68)	55.1 (26.9-85.1)	13.2 (1.8-33.4)	19.1 (2.7-48.2)	10.5 (1.5-26.3)
Moldova, Rep.	13.6	110.3 (100.3-120.7)	46.6 (37.5-57.4)	51.4 (41.2-63.5)	12.6 (2.2-28)	18.3 (3.1-40.5)	10.2 (1.7-22.2)
Montenegro	2.3	100.3 (91.9-109.7)	40 (20-70)	42 (19-69.6)	9.8 (1.4-26.8)	14.2 (2.1-38.8)	7.8 (1.1-20.9)
North Macedonia	6.8	100.9 (91.8-110.4)	44.9 (35.9-55.5)	45.2 (35.8-56.4)	11.1 (1.9-25.3)	16.1 (2.8-36.3)	8.9 (1.6-19.7)
Romania	60.3	103 (94.2-111.8)	42.7 (34.6-51.3)	44 (35.1-53.3)	10.8 (1.8-24.2)	15.7 (2.6-34.9)	8.7 (1.5-19)
Russian Fed.	541.7	100.5 (91.9-109.5)	43.3 (35.5-51.8)	43.5 (35.5-52.2)	10.7 (1.8-24.1)	15.5 (2.7-34.5)	8.6 (1.5-18.8)
Serbia	27.9	101.5 (92.9-110.5)	42.2 (33.8-51.9)	42.7 (34.1-53.2)	10.6 (1.9-24.2)	15.3 (2.7-34.5)	8.5 (1.5-18.7)
Tajikistan	68.4	133.9 (124.9-143.6)	55.6 (33.3-77.8)	77.9 (45.5-108.2)	18.7 (2.8-45.2)	27 (4.1-65)	15 (2.4-35.6)
Turkey	401	103.1 (94.6-112.4)	46.1 (37.9-55.1)	47.6 (38.7-57.8)	11.8 (2-27)	17 (3-38.9)	9.4 (1.7-20.9)
Turkmenistan	37.8	95 (87.9-102.3)	30 (10-70)	31.2 (8.9-66)	7.6 (0-24.2)	11 (0-34.6)	6.1 (0-18.8)
Ukraine	150.6	107.5 (98.6-116.8)	43.9 (35.6-53.2)	47.2 (38.1-57.5)	11.7 (2-26.3)	16.9 (2.9-37.2)	9.4 (1.7-20.5)
Uzbekistan	192.8	118.2 (108.3-129.3)	47.5 (37.5-58.8)	56.3 (44.4-68.8)	13.9 (2.4-30.9)	20.1 (3.4-44.1)	11.1 (1.9-24.1)
**Southeast Asia Region**							
Bangladesh	913.3	146.6 (139-154.4)	53.5 (46.5-60.5)	78.4 (67.5-90)	19.5 (3.4-43)	28.1 (4.9-62)	15.6 (2.8-34.4)
Bhutan	4.3	128.4 (116.2-140.9)	56.9 (46.3-69.2)	73 (60.1-88.2)	18.1 (3.2-40.4)	26.2 (4.6-58.1)	14.5 (2.6-32)
India	7399.5	136.8 (130.9-142.8)	51.5 (48.9-54.4)	70.5 (65.8-75.4)	17.6 (3-38.4)	25.4 (4.4-55.4)	14 (2.4-30.7)
Indonesia	1449.1	115.6 (109.7-121.8)	64 (56-72.2)	73.8 (63.9-84.5)	18.4 (3.2-40.4)	26.5 (4.6-58.5)	14.7 (2.6-32.1)
Maldives	2.2	89.5 (81.1-98.5)	57.9 (35-80)	52.2 (31.6-73.5)	12.6 (2.1-30.9)	18.2 (3-44.5)	10 (1.7-24.2)
Myanmar	273.6	153.7 (144.8-163.1)	66.9 (55.6-78.4)	102.9 (84.5-121.7)	25.5 (4.3-56.5)	36.9 (6.3-81.8)	20.3 (3.5-44.8)
Nepal	172.3	152.4 (142.7-162.6)	53.3 (37.8-68.9)	81.5 (57.7-105.4)	19.8 (3.5-45.1)	28.7 (5-65.2)	15.9 (2.7-35.8)
Sri Lanka	101.2	113.4 (104-124.2)	55.5 (45.1-67.9)	63 (51.5-77)	15.6 (2.7-35)	22.5 (3.9-50.4)	12.5 (2.2-27.4)
Thailand	234.7	114.3 (107.2-122)	58 (46.8-69.6)	66.2 (53.1-80.5)	16.3 (2.9-36.6)	23.6 (4.2-52.3)	13.1 (2.4-28.9)
Timor-Leste	12.1	140.6 (133.2-148.3)	65.5 (50.9-79.7)	92.4 (71.6-113.3)	22.8 (3.7-51.7)	33 (5.5-73.9)	18.1 (3-40.5)
**Western Pacific Region**							
American Samoa	0.3	108.5 (97-120.3)	47.3 (37.5-60.4)	51.1 (40.3-66.6)	12.7 (2.2-29.4)	18.3 (3.2-42.3)	10.1 (1.8-22.8)
Cambodia	106.2	153.4 (145-162.2)	68 (57.4-79.1)	104.4 (87.2-122.4)	25.7 (4.4-57.7)	37.2 (6.4-83)	20.6 (3.7-45.6)
China, People's Rep.	5134.2	104.5 (95.9-113.9)	47.9 (38.9-58.3)	50.1 (40.5-61.1)	12.4 (2.2-27.6)	17.9 (3.2-39.8)	9.9 (1.8-21.9)
Fiji	5.4	108.6 (99.2-118.9)	46.3 (37.9-56.1)	50.3 (40.7-61.2)	12.4 (2.1-28)	17.9 (3.1-40.1)	9.9 (1.7-22)
Kiribati	0.9	123.2 (111.7-136)	47.7 (38.5-59.5)	58.8 (47.5-73.3)	14.5 (2.6-33.3)	21 (3.8-48)	11.7 (2.1-26)
Lao People's Dem. Rep.	46.6	144.1 (136.6-152.2)	69.2 (55.4-82.8)	100 (78.9-120.3)	24.6 (4.2-55.2)	35.6 (6.1-79.4)	19.7 (3.4-43.5)
Malaysia	151.6	102.9 (94.3-111.8)	48.4 (40.2-58.5)	49.8 (41-60.6)	12.4 (2.1-28)	17.8 (3.1-40.3)	9.9 (1.7-21.9)
Marshall Islands	0.3	110.3 (100-122.5)	41.9 (34.3-51.8)	46.3 (37.6-57.3)	11.4 (2.1-25.8)	16.5 (3-37.1)	9.2 (1.7-20.5)
Micronesia, Fed.	3.1	125.8 (114.5-139.1)	49.1 (39.1-61.2)	61.8 (49.7-76.6)	15.4 (2.6-34.8)	22.2 (3.8-49.8)	12.3 (2.1-26.8)
Mongolia	20.6	109.2 (102.3-116.5)	51.1 (42.2-60.4)	55.9 (45.4-66.9)	13.9 (2.4-30.7)	20.1 (3.5-44.1)	11 (2-24.1)
Nauru	0.1	111.5 (100.3-124.4)	48 (38.6-61.6)	53.4 (42.5-69.9)	13.3 (2.3-30.7)	19.3 (3.3-44.1)	10.6 (1.9-24)
Papua New Guinea	60.9	140.7 (128.8-153.9)	48.8 (40-59.1)	68.7 (56.6-82.6)	17.1 (2.9-38.1)	24.8 (4.3-54.9)	13.7 (2.5-30)

Philippines	679.1	118.9 (113.1-125.2)	63.5 (52.1-75.5)	75.6 (61.2-90.6)	18.8 (3.2-41.4)	27.1 (4.7-59.8)	15 (2.6-32.7)
Samoa	1.5	113.2 (102.3-125.9)	48 (39.4-58.8)	54.4 (44.5-66.5)	13.4 (2.4-30.3)	19.4 (3.4-43.6)	10.8 (1.9-23.7)
Solomon Islands	5	141.7 (128.9-155.6)	47.9 (39.3-58.8)	68 (56-82.9)	16.9 (2.9-37.8)	24.4 (4.3-54.2)	13.6 (2.4-29.5)
Tonga	0.8	117.8 (106.4-130.8)	52.8 (41.3-67.2)	62.1 (49.1-78.7)	15.4 (2.7-35.6)	22.2 (3.9-51.1)	12.4 (2.2-27.4)
Tuvalu	0.1	105.9 (95.7-117.5)	44.6 (36.9-53.4)	47.3 (39.2-56.6)	11.7 (2-26.1)	16.8 (2.9-37.7)	9.4 (1.6-20.6)
Vanuatu	2.1	136.9 (122.6-152.2)	46.2 (36.9-58)	63.3 (51.5-77.8)	15.6 (2.6-35.1)	22.5 (3.9-50.2)	12.5 (2.2-27.9)
Viet Nam	463.9	121.9 (114.4-129.6)	59.6 (47.3-72)	72.5 (57.5-88.3)	17.8 (3.2-40.2)	25.7 (4.6-58)	14.3 (2.4-31.8)

Incidence rate estimates are presented as cases per 100 children annually in the eligible age range. Estimates are obtained from analyses of DHS/MICS surveys covering 944,173 children across 77 countries, as well as extrapolations based on 405 health, nutrition, and population indicators for all LMICs. Quantiles are obtained via 5,000 independent draws from the distribution of estimates.

Table S12: Country-specific estimates of the incidence of ARI and antibiotic-treated ARI among children ages 0-59 months preventable by 10- and 13-valent pneumococcal conjugate vaccines.

Country	Population ages 0-59 months	ARI attributable to all causes			ARI attributable to PCV10/13-type *Streptococcus pneumoniae*		
	Children (in 10,000s)	All cases, per 100 (95% CI)	Proportion treated with antibiotics, % (95% CI)	Antibiotic-treated cases, per 100 (95% CI)	Antibiotic-treated cases, per 100 (95% CI)		Antibiotic-treated cases preventable by vaccine direct effects, per 100 (95% CI)
					IPD probe estimate	*AOM probe estimate*	
**African Region**							
Algeria	466.4	118.8 (109.6-128.3)	46.5 (39.7-56.2)	55.3 (47-67)	6.1 (-0.8-14.4)	8.8 (-1.2-20.6)	4.9 (-0.7-11.1)
Angola	515.8	141.6 (135.2-148.2)	36.6 (31-42)	51.8 (43.8-59.7)	5.6 (-0.8-13.4)	8.2 (-1.2-19.1)	4.5 (-0.7-10.4)
Benin	173.9	195.6 (185.8-205.7)	41.4 (34.3-48.5)	81 (66.5-95.7)	8.8 (-1.3-20.9)	12.8 (-1.8-30)	7.1 (-1-16.2)
Botswana	25.6	128.1 (117.5-139.2)	41.9 (35.8-49.1)	53.6 (45.8-63)	5.9 (-0.8-13.9)	8.5 (-1.2-19.8)	4.7 (-0.7-10.6)
Burkina Faso	316.1	211.5 (200.7-223.1)	41.4 (35.6-47.3)	87.6 (74.8-101.3)	9.6 (-1.3-22.4)	13.9 (-2-32.4)	7.7 (-1.1-17.3)
Burundi	185.3	228.9 (214-245.1)	44.3 (40.4-48.3)	101.3 (90.5-113.3)	11.1 (-1.6-26.2)	16.1 (-2.4-37.5)	8.9 (-1.3-20.1)
Cabo Verde	5.5	130.6 (119.8-142.9)	46.5 (39.3-55)	60.7 (51.6-71.2)	6.6 (-0.9-15.8)	9.6 (-1.3-22.7)	5.3 (-0.8-12.2)
Cameroon	374.2	178.3 (170.9-185.9)	42.5 (38-46.7)	75.7 (67.3-84.3)	8.3 (-1.2-19.3)	12 (-1.7-27.9)	6.6 (-1-15)
Cent. African Rep.	72.8	197.2 (178.2-215.5)	40.2 (35.4-45.4)	79.1 (68.7-90.3)	8.6 (-1.3-20.3)	12.5 (-1.8-29.1)	7 (-1-15.8)
Chad	260.1	201.6 (192.2-211.6)	41.1 (38-44.2)	82.8 (75.3-90.5)	9.1 (-1.3-21.2)	13.1 (-1.9-30.3)	7.2 (-1.1-16.5)
Comoros	11.7	196 (185.6-207.2)	41 (31.7-51)	80.5 (61.9-100.3)	8.7 (-1.3-21.3)	12.6 (-1.8-30.7)	7 (-1-16.4)
Congo, Rep.	81.4	160.6 (154.2-167.1)	41.3 (36.8-46.1)	66.4 (58.5-74.4)	7.2 (-1-17.1)	10.5 (-1.5-24.4)	5.8 (-0.8-13.2)
Congo, Dem. Rep.	1409.9	208.6 (196.9-220.9)	44.6 (41.3-47.8)	92.9 (84.5-101.7)	10.1 (-1.5-23.6)	14.7 (-2.1-34.1)	8.2 (-1.2-18.3)
Cote d'Ivoire	376.5	182.6 (174.4-191)	39 (33.3-45.2)	71.2 (60-82.7)	7.7 (-1.1-18.5)	11.2 (-1.5-26.4)	6.2 (-0.9-14.3)
Equatorial Guinea	17.6	128.5 (116.1-143.5)	43.5 (35.6-52.7)	55.8 (45.8-67.7)	6.1 (-0.9-14.5)	8.9 (-1.3-20.6)	4.9 (-0.7-11.2)
Eritrea	74.4	186.9 (162.9-210.9)	46.1 (39-55.1)	85.9 (72-102)	9.4 (-1.4-22.4)	13.7 (-2-32.1)	7.5 (-1.1-17.3)
Eswatini	17.8	153.6 (145.9-162)	38.7 (32.3-45.5)	59.5 (49.3-70.6)	6.5 (-0.9-15.4)	9.4 (-1.3-22.1)	5.2 (-0.7-11.9)
Ethiopia	1490.1	201.8 (192-211.9)	38.9 (34-43.6)	78.5 (67.7-89.1)	8.5 (-1.2-20.1)	12.4 (-1.7-28.6)	6.9 (-1-15.5)
Gabon	26.7	115.7 (109.1-122.6)	36.3 (31.6-41.4)	42.1 (36.3-48.5)	4.6 (-0.7-10.8)	6.7 (-1-15.5)	3.7 (-0.5-8.4)
Gambia, The	35.2	201.2 (190.9-212.1)	42.6 (37.4-47.7)	85.7 (74.4-97.3)	9.3 (-1.4-21.9)	13.5 (-1.9-31.3)	7.5 (-1-17.1)
Ghana	402.3	166.9 (159.9-173.9)	43.2 (36.3-50.5)	72.1 (60-84.9)	7.8 (-1.1-18.6)	11.3 (-1.6-26.7)	6.3 (-0.9-14.4)
Guinea	194.9	205 (194.9-215.9)	40.6 (35.5-45.4)	83 (72.2-94.4)	9.1 (-1.3-21.3)	13.1 (-1.9-30.7)	7.3 (-1.1-16.7)
Guinea-Bissau	28.6	144.4 (136.3-153.1)	41.7 (34.1-49.4)	60.1 (49-72.2)	6.6 (-0.9-15.6)	9.5 (-1.3-22.4)	5.3 (-0.7-12)
Kenya	699.7	171.8 (165.1-178.7)	39.8 (37-42.6)	68.3 (63-73.9)	7.5 (-1.1-17.5)	10.8 (-1.5-25.2)	6 (-0.9-13.5)
Lesotho	28	170.4 (162.1-179)	41.6 (33.6-50)	71.2 (56.9-86)	7.8 (-1.1-18.5)	11.2 (-1.6-26.4)	6.2 (-0.9-14.3)
Liberia	70.5	197.9 (187.3-208.8)	43.1 (38.8-47.6)	85.3 (75.9-95.6)	9.4 (-1.4-21.9)	13.6 (-2-31.4)	7.5 (-1.1-16.9)
Madagascar	370	192.2 (177.6-207.5)	47.3 (41-54.4)	91 (78.6-104.2)	9.9 (-1.5-23.2)	14.3 (-2.1-33.4)	7.9 (-1.2-18)
Malawi	288.8	222.3 (208.5-237.9)	45.6 (41.6-49.5)	101.5 (90.7-113)	11.1 (-1.6-26.1)	16.1 (-2.3-37.4)	8.9 (-1.3-20.2)
Mali	327.4	203 (193.2-213.1)	36.1 (29.3-43.9)	73.6 (58.7-89.8)	8 (-1.1-18.9)	11.6 (-1.6-27.2)	6.4 (-0.9-14.8)
Mauritania	64	149.1 (142.4-155.9)	39.9 (34.7-45.1)	59.4 (51.3-67.9)	6.5 (-0.9-15.2)	9.4 (-1.3-21.9)	5.2 (-0.7-11.9)
Mauritius	6.9	118.6 (109.7-128.8)	44.6 (37.4-53.5)	53 (44.5-63)	5.8 (-0.8-13.8)	8.4 (-1.2-19.8)	4.6 (-0.7-10.7)
Mozambique	484.4	218.4 (205.2-232.5)	43.1 (37.3-49)	94 (80.4-108.6)	10.3 (-1.5-24.2)	14.8 (-2.1-34.6)	8.2 (-1.2-18.9)
Namibia	33.5	135.7 (129-142.8)	36.5 (30.6-42.7)	49.5 (41.1-58.5)	5.4 (-0.8-12.8)	7.8 (-1.1-18.4)	4.3 (-0.6-10)
Niger	406.6	226.1 (213.1-240.5)	41.6 (37-46.1)	93.9 (82.4-106.7)	10.3 (-1.5-24)	14.9 (-2.2-34.4)	8.2 (-1.2-18.8)
Nigeria	3110.9	172.8 (166.4-179.8)	42.7 (38-47.4)	73.7 (65.2-82.5)	8.1 (-1.1-18.8)	11.7 (-1.6-26.9)	6.5 (-0.9-14.6)
Rwanda	173.5	203 (192.7-214.2)	42.8 (37.9-47.6)	86.8 (76-97.7)	9.5 (-1.4-22.4)	13.7 (-2-32.2)	7.6 (-1.1-17.2)
Sao Tome and Principe	3.1	173 (163.9-182.8)	43.7 (35-52.6)	75.6 (60.5-91.7)	8.2 (-1.2-19.9)	11.8 (-1.7-28.4)	6.5 (-0.9-15.2)
Senegal	249.3	180.3 (172.6-188.1)	40.1 (35.4-45)	72.2 (63.3-81.9)	7.9 (-1.1-18.6)	11.4 (-1.6-26.7)	6.3 (-0.9-14.5)

Sierra Leone	113.5	216 (203.9-228.9)	42.8 (39.3-46.5)	92.4 (83.2-102.3)	10.2 (-1.4-23.7)	14.7 (-2.1-34.2)	8.1 (-1.2-18.3)
South Africa	566.4	114.3 (107.7-120.9)	36.8 (26.3-48.1)	42.4 (29.9-55.8)	4.6 (-0.6-11.3)	6.6 (-0.9-16.3)	3.6 (-0.5-8.9)
South Sudan	188.2	185 (165-209.6)	41.7 (35-50.5)	78.1 (64.6-90.5)	8.5 (-1.3-20.3)	12.3 (-1.8-28.7)	6.8 (-1-15.5)
Tanzania, United Rep.	941.9	194.8 (185.8-204.2)	41.6 (35.9-47.3)	81 (69.5-93.5)	8.7 (-1.3-20.8)	12.7 (-1.8-29.9)	7 (-1-16.2)
Togo	116.2	203.2 (192.2-214.7)	42.3 (35.9-48.4)	85.8 (72.6-99.8)	9.4 (-1.3-22.1)	13.6 (-1.9-31.6)	7.5 (-1-16.9)
Uganda	751.2	198.9 (188.8-209.3)	48.4 (44.3-52.6)	96.2 (86.9-105.9)	10.5 (-1.5-24.7)	15.2 (-2.2-35.4)	8.4 (-1.2-19.2)
Zambia	275	176.2 (169.1-183.7)	40.1 (35.5-44.9)	70.6 (62.1-79.6)	7.7 (-1.1-18.3)	11.2 (-1.6-26.4)	6.2 (-0.9-14.3)
Zimbabwe	250.6	167.1 (159.8-174.7)	40.5 (33.7-47.7)	67.9 (56-80.7)	7.4 (-1.1-17.5)	10.7 (-1.6-25)	5.9 (-0.8-13.4)
**Region of the Americas**							
Belize	3.9	132.3 (125.1-139.8)	38.5 (28.1-48.4)	50.7 (37.1-64.7)	5.5 (-0.8-13.5)	7.9 (-1.1-19.3)	4.4 (-0.6-10.5)
Bolivia, Plurinational State	118.6	137.8 (131.9-144.2)	68.3 (61.7-75.1)	94.2 (84.1-104.2)	10.3 (-1.5-24.1)	14.9 (-2.2-34.7)	8.2 (-1.2-18.7)
Brazil	1487.5	117.3 (109.2-125.6)	43.4 (37.6-50.5)	50.9 (43.9-59)	5.6 (-0.8-13.2)	8 (-1.1-18.9)	4.5 (-0.7-10.2)
Colombia	373.8	112.4 (107-118.1)	38.7 (35.5-42.2)	43.5 (39.3-47.9)	4.8 (-0.7-11.2)	6.9 (-1-16)	3.8 (-0.6-8.6)
Costa Rica	35	113.5 (105.6-121.9)	43.2 (36.9-50.5)	49 (41.9-57.3)	5.4 (-0.7-12.9)	7.7 (-1.1-18.4)	4.3 (-0.6-9.8)
Cuba	62.9	110.4 (104.2-116.7)	32.6 (27.1-38.5)	36 (29.6-42.8)	3.9 (-0.6-9.4)	5.7 (-0.8-13.5)	3.1 (-0.5-7.3)
Dominica	0.6	119.6 (109.9-130.2)	43.9 (37.1-51.6)	52.5 (44-62.3)	5.7 (-0.8-13.6)	8.3 (-1.2-19.7)	4.6 (-0.7-10.6)
Dominican Rep.	106.2	125.6 (119.4-131.8)	34.4 (31.6-37.3)	43.2 (39.2-47.5)	4.7 (-0.7-11)	6.8 (-1-15.8)	3.8 (-0.6-8.6)
Ecuador	161	116.5 (108.9-124.8)	44 (38.5-50)	51.3 (44.6-58.8)	5.6 (-0.8-13.1)	8.1 (-1.1-18.9)	4.5 (-0.6-10.1)
El Salvador	58	120.2 (114.5-126.2)	35.8 (31.2-40.3)	43 (37.3-49)	4.7 (-0.7-11)	6.8 (-1-15.8)	3.8 (-0.5-8.6)
Grenada	1	120.2 (110.3-130.8)	45.8 (37.2-54.9)	55 (44.4-66.3)	6 (-0.8-14.1)	8.7 (-1.2-20.5)	4.8 (-0.7-11)
Guatemala	199.4	148.2 (141.8-155)	39.4 (36.3-42.5)	58.4 (53.2-63.8)	6.4 (-0.9-14.9)	9.2 (-1.3-21.4)	5.1 (-0.8-11.6)
Guyana	7.5	134.4 (126.7-142.2)	39.8 (30.8-48.7)	53.6 (41.2-66.1)	5.8 (-0.9-14.2)	8.4 (-1.2-20.3)	4.6 (-0.7-10.9)
Haiti	123.8	190.2 (180.9-200)	41.9 (37.4-46.9)	79.8 (70.1-89.9)	8.7 (-1.3-20.4)	12.6 (-1.8-29.2)	7 (-1-15.8)
Honduras	95.5	152.6 (146.6-159.1)	42.1 (39.1-45.3)	64.3 (59.1-70.3)	7 (-1-16.5)	10.2 (-1.5-23.6)	5.6 (-0.8-12.8)
Jamaica	20.7	118.8 (110-128.4)	42.2 (36.3-48.6)	50.1 (43.1-57.8)	5.5 (-0.8-12.9)	8 (-1.1-18.5)	4.4 (-0.6-9.9)
Mexico	1153.3	115.5 (109.1-122.3)	47.5 (39.5-56.6)	55 (45.4-66.4)	6 (-0.9-14.4)	8.7 (-1.3-20.6)	4.8 (-0.7-11.2)
Nicaragua	60.6	134.2 (123-146)	47.6 (40.1-56.9)	64.1 (53.8-75.6)	7 (-1-16.4)	10.2 (-1.4-23.5)	5.6 (-0.8-12.9)
Paraguay	67.4	112.2 (106.2-118.3)	57.1 (51.2-63)	64 (56.5-71.7)	7 (-0.9-16.5)	10.1 (-1.4-23.7)	5.6 (-0.8-12.7)
Peru	302	119 (113.1-125.2)	58.9 (52.8-65.2)	70 (62.1-78.7)	7.6 (-1.1-17.9)	11.1 (-1.6-25.6)	6.1 (-0.9-14)
St. Lucia	1.1	120.2 (110.2-131.1)	43.7 (36.4-51.5)	52.4 (43.7-61.9)	5.7 (-0.8-13.6)	8.3 (-1.2-19.5)	4.6 (-0.7-10.5)
St. Vincent and the Grenadines	0.9	120.6 (110.9-131.1)	43.4 (36.6-51.2)	52.4 (44-61.9)	5.7 (-0.8-13.7)	8.3 (-1.2-19.6)	4.6 (-0.7-10.4)
Suriname	5	122.5 (111.9-134.5)	43.4 (36.7-51.3)	53.2 (45-62.9)	5.8 (-0.8-13.7)	8.4 (-1.2-19.6)	4.7 (-0.7-10.5)
Venezuela, Bolivarian Rep.	297.5	118 (109.8-128)	45.8 (38.6-53.9)	54.1 (45-63.9)	5.9 (-0.8-13.9)	8.6 (-1.2-20)	4.7 (-0.7-10.8)
**Eastern Mediterranean Region**							
Afghanistan	523.9	200.7 (190.1-211.6)	61.5 (58.9-64)	123.4 (115-132.1)	13.5 (-1.9-31.4)	19.6 (-2.7-44.9)	10.8 (-1.6-24.5)
Djibouti	10.1	145.8 (132.5-160.6)	45.9 (38.4-54.4)	67 (56.6-78.5)	7.3 (-1-17.3)	10.6 (-1.5-24.8)	5.8 (-0.8-13.3)
Egypt, Arab Rep.	1237.4	145.5 (140.3-151)	57.9 (55.3-60.7)	84.3 (79.6-89.4)	9.2 (-1.3-21.5)	13.4 (-1.9-30.9)	7.4 (-1.1-16.5)
Iran, Islamic Rep.	689.9	117 (108.9-126.1)	47.4 (41.1-54.7)	55.5 (48.4-63.8)	6 (-0.9-14.2)	8.8 (-1.3-20.3)	4.9 (-0.7-11)
Iraq	560.3	132.2 (120.8-145.4)	47.7 (39.9-56.7)	62.9 (52.9-75.1)	6.9 (-1-16.3)	9.9 (-1.4-23.3)	5.5 (-0.8-12.7)
Jordan	120.2	110.2 (105.1-115.7)	55.8 (51.6-60.3)	61.6 (56.1-67.4)	6.7 (-1-15.7)	9.7 (-1.4-22.7)	5.4 (-0.8-12.2)
Lebanon	46.1	113.3 (103.6-124.3)	48.9 (41.8-57.1)	55.4 (47.2-64.4)	6.1 (-0.9-14.3)	8.8 (-1.3-20.4)	4.9 (-0.7-11)
Libyan Arab Rep.	63	124.3 (111.5-143.7)	49 (40.4-59.2)	61.3 (50.8-73.5)	6.7 (-1-16.1)	9.7 (-1.4-23)	5.4 (-0.8-12.4)
Morocco	345.7	132.7 (121.8-143.8)	49.3 (41.5-58.6)	65.5 (56-76.6)	7.1 (-1-16.9)	10.3 (-1.5-24.3)	5.7 (-0.8-13.2)
Pakistan	2471.2	164.6 (158-171.4)	53.6 (50.4-56.7)	88.2 (81.9-94.7)	9.6 (-1.4-22.4)	14 (-2-32.3)	7.7 (-1.1-17.4)
Palestinian Terr.	69.9	127.2 (121.4-133.2)	53.1 (49.1-57.2)	67.5 (61.5-73.7)	7.4 (-1.1-17.2)	10.7 (-1.5-24.7)	5.9 (-0.9-13.2)
Somalia	255.4	162.8 (143-193.3)	44.2 (36.9-53.1)	72.8 (61.1-85.9)	7.9 (-1.1-18.9)	11.4 (-1.6-27.1)	6.3 (-0.9-14.7)
Sudan	585.9	169.4 (154.2-184.5)	44.1 (37.9-51)	74.6 (64.7-85.6)	8.2 (-1.2-19.1)	11.8 (-1.7-27.5)	6.5 (-1-14.9)
Tunisia	102.4	117.4 (108.6-127)	48.6 (41.5-57)	57 (49-66.6)	6.2 (-0.9-14.8)	9 (-1.3-21.2)	5 (-0.7-11.3)

Yemen, Rep.	401.7	166.2 (159.1-173.5)	61.6 (58.9-64.5)	102.3 (96.1-108.9)	11.2 (-1.6-26.1)	16.3 (-2.3-37.4)	9 (-1.3-20.4)
**European Region**							
Albania	17.2	125 (117.9-132.1)	49.2 (33.3-65)	61.4 (41.7-82)	6.6 (-0.9-16.9)	9.5 (-1.3-24.1)	5.2 (-0.7-12.8)
Armenia	20.6	111.5 (104.3-118.9)	55.6 (37-77.8)	63.8 (41.9-85.3)	6.8 (-1-17.5)	9.9 (-1.4-25.1)	5.5 (-0.8-13.6)
Azerbaijan	86.6	120.4 (113.3-127.7)	43 (30-56.4)	51.7 (35.7-68.8)	5.5 (-0.8-13.9)	8 (-1.1-20)	4.4 (-0.6-10.8)
Belarus	57.6	113.7 (105.5-122.4)	42.9 (35.8-50.7)	48.8 (40.7-57.8)	5.3 (-0.8-12.5)	7.7 (-1.1-17.9)	4.2 (-0.6-9.7)
Bosnia and Herzegovina	16.2	113.1 (104.5-123.4)	44.8 (37.7-53.2)	50.8 (42.2-60.6)	5.5 (-0.8-13.1)	8 (-1.1-18.8)	4.4 (-0.7-10.1)
Bulgaria	33.6	112 (103.8-121)	43.8 (36.1-51.9)	49.1 (40.4-58.3)	5.3 (-0.8-12.7)	7.7 (-1.1-18)	4.3 (-0.6-9.8)
Georgia	27.5	117.4 (108.4-126.8)	44.6 (36.9-53.5)	52.3 (43.4-62.9)	5.7 (-0.8-13.5)	8.3 (-1.2-19.6)	4.6 (-0.7-10.6)
Kazakhstan	199.1	116 (108-124.9)	45.7 (38.8-53.5)	53.1 (45-61.8)	5.8 (-0.8-13.6)	8.3 (-1.2-19.6)	4.6 (-0.7-10.5)
Kosovo	15	117.9 (110.9-125.2)	50 (37.6-63.2)	58.9 (44-75.1)	6.4 (-0.9-15.8)	9.3 (-1.4-22.6)	5.1 (-0.7-12.1)
Kyrgyzstan	74.8	143.8 (136.7-151.3)	44.8 (30.4-60.7)	65.3 (42.7-88.7)	7 (-0.9-17.6)	10.1 (-1.4-25.1)	5.6 (-0.8-13.7)
Moldova, Rep.	22.2	122.8 (112.5-134)	47.9 (39.7-57.5)	58.9 (48.9-70.4)	6.4 (-0.9-15.2)	9.3 (-1.3-21.8)	5.1 (-0.8-11.7)
Montenegro	3.7	108.6 (100.9-116.7)	42.9 (19-66.7)	46.6 (20.8-73.5)	4.9 (-0.7-13.7)	7 (-0.9-19.7)	3.9 (-0.5-10.6)
North Macedonia	11.6	112.9 (104.2-122.5)	45.9 (38.2-54.8)	51.8 (42.7-62.6)	5.6 (-0.8-13.5)	8.2 (-1.2-19.2)	4.5 (-0.7-10.4)
Romania	97.1	115.2 (106.7-123.9)	43.9 (36.6-51.5)	50.6 (41.8-59.7)	5.5 (-0.8-13.1)	7.9 (-1.1-18.7)	4.4 (-0.6-10.1)
Russian Fed.	935.5	113.1 (104.7-121.8)	44.4 (37.6-51.6)	50.1 (42.6-58.5)	5.5 (-0.8-12.9)	7.9 (-1.1-18.5)	4.4 (-0.6-9.9)
Serbia	46.8	113.2 (104.7-122.3)	43.5 (36.2-52.1)	49.3 (40.9-59)	5.4 (-0.8-12.7)	7.8 (-1.1-18.2)	4.3 (-0.6-9.9)
Tajikistan	116.5	153.6 (145.6-161.8)	59.5 (42.9-73.8)	90.4 (66.3-115.6)	9.9 (-1.5-23.9)	14.3 (-2.1-34.2)	7.9 (-1.1-18.3)
Turkey	674	115.5 (107.4-124.6)	47 (39.6-55)	54.2 (45.2-64)	5.9 (-0.8-14.2)	8.6 (-1.2-20.3)	4.7 (-0.7-10.8)
Turkmenistan	67.9	108.8 (102.6-115.2)	35.3 (17.6-64.7)	40.2 (18.6-69.7)	4.2 (-0.6-12.6)	6.1 (-0.9-18.2)	3.4 (-0.5-9.8)
Ukraine	241	120.5 (111.6-130.8)	45.1 (37.6-53.3)	54.4 (45.2-64.6)	5.9 (-0.8-13.9)	8.6 (-1.2-19.8)	4.7 (-0.7-10.7)
Uzbekistan	319.7	134.2 (123.4-146.6)	49 (39.5-58.8)	65.6 (53.8-77.9)	7.1 (-1-17)	10.3 (-1.5-24.4)	5.7 (-0.8-13.3)
**Southeast Asia Region**							
Bangladesh	1525.4	169.6 (162.9-176.5)	53.4 (48.3-58.7)	90.7 (81.3-100.2)	9.9 (-1.4-23.3)	14.4 (-2-33.3)	8 (-1.2-18)
Bhutan	7.1	147.5 (132.9-162.4)	57 (47.4-68.1)	84 (70.9-99.1)	9.2 (-1.3-21.9)	13.3 (-1.8-31.1)	7.4 (-1-16.7)
India	12141.5	158.6 (153.3-164.1)	51.9 (49.7-53.9)	82.2 (78-86.7)	9 (-1.3-21)	13.1 (-1.8-30.2)	7.2 (-1-16.2)
Indonesia	2459.2	134.2 (129-139.8)	62.6 (56.7-68.6)	83.9 (75.4-93.1)	9.2 (-1.3-21.6)	13.3 (-1.9-31)	7.4 (-1.1-16.8)
Maldives	3.8	103.3 (95.9-111.2)	57.7 (38.5-76.9)	60.1 (39.6-81.3)	6.4 (-0.9-16.4)	9.3 (-1.3-23.7)	5.2 (-0.7-12.8)
Myanmar	455.3	178.3 (170.3-186.5)	65.2 (56.6-73.7)	116.1 (100.5-132.2)	12.7 (-1.8-29.8)	18.3 (-2.5-42.4)	10.2 (-1.4-22.9)
Nepal	280.5	176.2 (167.7-185.4)	52.3 (42.2-61.8)	92.1 (74.2-109.4)	10.1 (-1.4-23.8)	14.5 (-2.1-34.1)	8 (-1.1-18.6)
Sri Lanka	164.3	128.6 (118.2-139.7)	55.3 (46.1-66.3)	71.2 (59.4-85.3)	7.8 (-1.1-18.2)	11.2 (-1.5-26.2)	6.1 (-0.9-14.2)
Thailand	384.4	126 (119.7-132.5)	57.8 (48.1-66.9)	72.7 (60.3-85.2)	7.9 (-1.1-18.7)	11.4 (-1.7-26.6)	6.3 (-0.9-14.5)
Timor-Leste	20.4	163.4 (157-170.2)	63.6 (53-73.9)	104.1 (86.1-121.4)	11.3 (-1.5-26.8)	16.4 (-2.2-38.5)	9.1 (-1.2-20.9)
**Western Pacific Region**							
American Samoa	0.5	123.8 (110.6-137)	47.7 (38.9-59.2)	58.9 (47.9-73.5)	6.5 (-0.9-15.7)	9.3 (-1.3-22.5)	5.2 (-0.7-12.2)
Cambodia	176.5	180.8 (173.4-188.7)	66.6 (58.7-74.3)	120.3 (105.6-135.9)	13.1 (-1.9-31.2)	19 (-2.7-44.7)	10.5 (-1.6-24.1)
China, People's Rep.	8588.5	118.4 (109.6-128.8)	47.9 (40-57)	56.8 (47.3-67.4)	6.2 (-0.9-14.8)	8.9 (-1.3-21.2)	5 (-0.7-11.4)
Fiji	8.8	124 (114.2-135.3)	47 (39.6-56)	58.5 (48.9-69.8)	6.4 (-0.9-15.1)	9.2 (-1.3-21.6)	5.1 (-0.7-11.8)
Kiribati	1.5	139.6 (127.1-154.1)	48.7 (40.3-58.7)	68.1 (56.3-81.3)	7.5 (-1-17.7)	10.8 (-1.5-25.4)	5.9 (-0.8-13.7)
Lao People's Dem. Rep.	77.2	166.8 (159.9-174)	67.7 (57.4-77.6)	112.8 (95.3-130.7)	12.3 (-1.8-29.4)	17.8 (-2.5-42)	9.8 (-1.4-22.6)
Malaysia	256.9	116.9 (107.8-125.7)	49.2 (42.2-57.8)	57.5 (48.8-68)	6.3 (-0.9-15)	9.1 (-1.3-21.4)	5 (-0.7-11.6)
Marshall Islands	0.5	124.7 (113.2-137.5)	42.2 (35-50.6)	52.7 (43.8-63)	5.7 (-0.8-13.6)	8.3 (-1.2-19.6)	4.6 (-0.7-10.6)
Micronesia, Fed.	5.1	143.4 (130.1-157.9)	49.8 (40.7-60.6)	71.4 (58.7-86.1)	7.8 (-1.1-18.5)	11.3 (-1.6-26.7)	6.3 (-0.9-14.5)
Mongolia	35.9	126.2 (120.2-132.4)	52 (45.1-58.5)	65.5 (56.4-74.5)	7.2 (-1-16.6)	10.4 (-1.5-23.8)	5.7 (-0.8-12.9)

Nauru	0.1	126.5 (114.5-139.3)	48.7 (40.6-62)	61.6 (51.3-78.8)	6.8 (-1-16.3)	9.9 (-1.4-23.4)	5.5 (-0.8-12.7)
Papua New Guinea	102.5	163.7 (149.9-178)	50 (41.7-59.4)	81.7 (68.8-95.7)	8.9 (-1.3-21.1)	13 (-1.9-30.4)	7.2 (-1-16.4)
Philippines	1143.4	135.6 (130.4-141)	62.6 (53.5-71.6)	84.9 (72.2-97.7)	9.3 (-1.3-21.8)	13.4 (-1.9-31.3)	7.4 (-1.1-16.9)
Samoa	2.4	127.4 (116-140.1)	48.2 (40.4-57.4)	61.5 (52.1-72.7)	6.7 (-1-16.1)	9.7 (-1.4-23)	5.4 (-0.8-12.3)
Solomon Islands	8.3	162.6 (148.1-178.4)	49.1 (41-58.7)	79.6 (67.4-94.3)	8.7 (-1.3-20.4)	12.6 (-1.8-29.2)	7 (-1-15.7)
Tonga	1.3	133.2 (121.7-146.4)	52.8 (42.1-65.6)	70.3 (56.8-86.6)	7.7 (-1-18.3)	11.1 (-1.5-26.3)	6.1 (-0.9-14.3)
Tuvalu	0.1	120.1 (109.8-132.3)	45.5 (38.4-53.6)	54.8 (46.5-64.4)	6 (-0.8-14.3)	8.6 (-1.2-20.5)	4.8 (-0.7-11.1)
Vanuatu	3.5	156.9 (139.9-175)	47 (38.6-58.7)	74.2 (62.4-88.5)	8.1 (-1.2-19.2)	11.7 (-1.7-27.6)	6.5 (-1-15)
Viet Nam	775.3	133.9 (127.4-140.7)	57.9 (47.1-67.8)	77.1 (63.3-91.5)	8.4 (-1.2-19.9)	12.1 (-1.7-28.5)	6.7 (-0.9-15.6)

Incidence rate estimates are presented as cases per 100 children annually in the eligible age range. Estimates are obtained from analyses of DHS/MICS surveys covering 944,173 children across 77 countries, as well as extrapolations based on 405 health, nutrition, and population indicators for all LMICs. Quantiles are obtained via 5,000 independent draws from the distribution of estimates.

Table S13: Country-specific estimates of the incidence of diarrhea and antibiotic-treated diarrhea among children ages 0-23 months preventable by rotavirus vaccination.

Country	Population ages 0-23 months	Diarrhea attributable to all causes			Diarrhea attributable to rotavirus	
	Children (in 10,000s)	All cases, per 100 (95% CI)	Proportion treated with antibiotics, % (95% CI)	Antibiotic-treated cases, per 100 (95% CI)	Antibiotic-treated cases, per 100 (95% CI)	Antibiotic-treated cases preventable by vaccine direct effects, per 100 (95% CI)
**African region**						
Algeria	191.3	211.5 (194.1-231)	19.3 (15.6-23.4)	41 (32.3-50.4)	10.1 (1.5-19.1)	6.6 (1.1-12.7)
Angola	215.1	299.7 (241.1-371.2)	15.4 (13.6-17.4)	46.2 (35.8-59.1)	11.2 (1.6-22.2)	7.6 (1.2-14.5)
Benin	72.6	343.5 (279-426)	17.2 (14.4-20.3)	59.2 (44.7-77.4)	10.9 (3.6-21.7)	6.1 (2.1-10.9)
Botswana	10.6	244.3 (218.4-274.3)	18.4 (14.8-22.7)	44.9 (35.3-56.8)	10.9 (1.6-21.2)	7.3 (1.2-13.8)
Burkina Faso	131	377.2 (304.2-468.5)	16.6 (14.8-18.5)	62.5 (49.1-79.9)	11.6 (3.9-22.7)	6.5 (2.2-11.4)
Burundi	77.9	405.8 (324.5-505.3)	16 (14.4-17.6)	64.9 (50.6-82)	11.9 (4.1-23.5)	6.7 (2.3-11.9)
Cabo Verde	2.2	234.1 (210.6-262.1)	18.3 (14.9-22.4)	43 (34-53.4)	10.6 (1.6-20.1)	7.1 (1.2-13.3)
Cameroon	155.1	318 (256.5-393)	19.6 (17.6-21.6)	62.2 (48.9-78.6)	15.1 (2.2-29.9)	10.2 (1.6-19.5)
Cent. African Rep.	30.2	358.6 (335.3-379.3)	17.6 (17-18.1)	63.1 (58.2-67.3)	11.7 (4.1-22.1)	6.5 (2.2-10.9)
Chad	109.6	371.9 (297.9-457.1)	16.4 (14.9-17.9)	60.9 (48.1-76.3)	11.2 (3.8-22.3)	6.3 (2.2-11)
Comoros	4.9	331.4 (267.1-411.4)	17.5 (14.2-20.9)	57.7 (42.7-77)	10.5 (3.5-21.4)	5.9 (2-10.7)
Congo, Rep.	33.5	302.6 (244-372.6)	17.9 (15.8-20)	54 (42.5-68.6)	13.4 (1.9-25.8)	8.8 (1.4-17.1)
Congo, Dem. Rep.	595.6	373.4 (301.3-466.7)	18.4 (16.7-20.1)	68.7 (54.1-87.7)	12.7 (4.4-25.2)	7.1 (2.4-12.5)
Cote d'Ivoire	158.3	329.2 (267.2-405.3)	17.1 (14.9-19.8)	56.4 (43.7-72.9)	13.9 (2-27.5)	9.2 (1.5-17.8)
Equatorial Guinea	7.4	266.7 (233.5-303.3)	19 (15.8-23.3)	50.7 (40.2-64.4)	12.3 (1.8-24.3)	8.3 (1.4-15.9)
Eritrea	29.7	328.2 (295.3-361)	19.7 (16.4-23.2)	64.5 (51.9-78.9)	11.9 (4-23.4)	6.7 (2.2-11.4)
Eswatini	7.2	271.1 (219-334.3)	16.2 (12.2-20.2)	43.6 (31.3-59.6)	10.6 (1.4-21.5)	7 (1.1-14)
Ethiopia	619.3	368.7 (293.2-453.8)	15.6 (13.2-18.1)	57.4 (43.4-74.4)	10.5 (3.5-21.3)	5.9 (2-10.6)
Gabon	11.2	222.4 (180.3-274.6)	15.3 (12.6-17.9)	33.9 (25.6-44.5)	8.2 (1.2-16.3)	5.5 (0.9-10.9)
Gambia, The	14.8	349.4 (281.2-433.8)	19.2 (16.9-21.6)	66.9 (52.5-86.2)	12.4 (4.2-24.4)	6.9 (2.4-12.4)
Ghana	166.5	301.9 (243.5-373.1)	20.9 (17.4-24.8)	63.1 (47.7-82.8)	15.4 (2.1-31)	10.3 (1.6-20.1)
Guinea	80.9	361.9 (292.2-445.5)	17.1 (14.3-20)	61.6 (47.1-80.2)	11.3 (3.8-22.7)	6.4 (2.2-11.5)
Guinea-Bissau	11.9	324.2 (260.4-401.4)	16.5 (13.3-19.8)	53.3 (39.2-71.5)	9.8 (3.3-19.9)	5.5 (1.9-10)
Kenya	280.4	314.1 (253.4-389.2)	16.3 (14.8-17.9)	51.2 (40.5-64.6)	12.7 (1.8-24.3)	8.3 (1.3-16.4)
Lesotho	11.7	297.4 (240.8-370.2)	16.3 (12.8-20.7)	48.7 (34.7-66.7)	11.8 (1.7-24)	7.9 (1.2-15.8)
Liberia	29	357.5 (289.4-442.9)	18.3 (16.1-20.6)	65.3 (51.4-83.1)	12 (4.2-23.9)	6.7 (2.3-11.9)
Madagascar	152.4	338.7 (307.3-370.4)	19.5 (16.9-22.7)	66.3 (55.8-78.8)	12.2 (4.2-23.4)	6.9 (2.4-11.7)
Malawi	115.7	388.2 (311.6-480.5)	16.9 (15.5-18.5)	65.6 (51.7-82.5)	12.1 (4.1-23.4)	6.8 (2.4-12)
Mali	135.9	366 (294.5-450.8)	17.1 (14.3-20.1)	62.2 (47.1-81.7)	11.4 (3.9-22.6)	6.4 (2.2-11.6)
Mauritania	26.7	316.3 (255.5-392.3)	16.7 (14.6-18.9)	52.8 (41.4-67.6)	13 (1.9-25.2)	8.6 (1.3-16.6)
Mauritius	2.7	213 (195.4-234.2)	18.2 (13.6-23.4)	38.8 (28.5-50.9)	9.4 (1.3-19)	6.3 (1-12.4)
Mozambique	202.3	389.6 (312.4-487)	17.8 (14.7-21.6)	69.5 (51.5-93.4)	12.8 (4.3-26)	7.2 (2.4-13)
Namibia	14.2	275.2 (221.4-340)	15.6 (13-18.6)	43.1 (32.1-56.9)	10.6 (1.5-20.8)	7 (1.1-13.7)
Niger	173.9	383.3 (308.8-472.3)	16.4 (14.4-18.5)	62.6 (48.9-79.4)	11.5 (3.9-23.1)	6.4 (2.2-11.3)
Nigeria	1306.7	325.9 (263.6-401.6)	21.1 (19.2-23.3)	68.7 (54.5-86.6)	17 (2.5-33)	11.2 (1.8-21.4)
Rwanda	69.8	337.9 (273-417.4)	16.5 (13.9-19.3)	56.1 (42.5-72.7)	10.3 (3.5-20.7)	5.7 (1.9-10.4)
Sao Tome and Principe	1.3	297.4 (240.3-370.5)	20.3 (14.6-26.8)	61.1 (41.7-86.3)	14.9 (2-31)	9.9 (1.5-20.1)
Senegal	103.5	327.6 (265.4-403.5)	17.2 (15.4-18.9)	56.3 (44.4-70.9)	10.4 (3.5-20.3)	5.8 (2-10.2)
Sierra Leone	46.2	377.7 (304.4-469)	17.2 (15.3-19.3)	65.2 (50.8-83.3)	12 (4.1-23.5)	6.7 (2.3-12)
South Africa	230.2	212.7 (172.4-264.1)	15.7 (11.5-20.4)	33.5 (23.2-46.9)	8.1 (1.1-17)	5.4 (0.9-11.1)
South Sudan	78.2	312.9 (273.7-347)	17.2 (14.3-19.9)	53.6 (42.7-64.4)	9.7 (3.4-19.2)	5.5 (1.8-9.5)

Tanzania, United Rep.	394	343.5 (278.5-426)	17.1 (14.8-19.4)	58.2 (45.5-75.1)	10.8 (3.6-21.3)	6 (2-10.7)
Togo	47.6	361.6 (291.3-445)	17.8 (15.2-20.5)	64.2 (49-83)	11.7 (4-23.4)	6.6 (2.3-11.8)
Uganda	314.7	342.8 (277.4-425.5)	21.4 (19.5-23.4)	73.6 (58-92.4)	13.5 (4.6-26.9)	7.6 (2.5-13.4)
Zambia	115	321.8 (260.4-397.8)	16.9 (15-18.8)	54.2 (42.7-68.8)	13.3 (1.9-26.4)	8.9 (1.4-17)
Zimbabwe	103.9	318.5 (256.2-398.6)	19 (16.3-21.9)	60.5 (46.3-78.6)	11.1 (3.8-22.3)	6.2 (2.1-11.1)
**Region of the Americas**						
Belize	1.6	250.4 (202.6-309.5)	14.1 (7.8-21.9)	36.4 (18.8-59.1)	8.5 (1.1-20.3)	5.7 (0.8-13.1)
Bolivia, Plurinational State	47.8	242.9 (195.9-300.3)	19.7 (17.1-22.7)	48 (37-61.6)	11.6 (1.6-22.9)	7.7 (1.2-15.3)
Brazil	602.8	214.2 (196.9-235.4)	15.9 (12.2-19.9)	33.9 (25.8-43.4)	8.3 (1.2-16.3)	5.5 (0.9-10.7)
Colombia	148.2	209.6 (169.7-259)	14.3 (12.7-16.1)	30 (23.6-38.3)	7.4 (1.1-14.5)	4.9 (0.8-9.5)
Costa Rica	13.7	203.3 (187.3-220.7)	16 (12.2-20)	32.6 (24.6-41.3)	8 (1.2-15.6)	5.3 (0.9-10.3)
Cuba	26	203.4 (163.4-250.8)	13 (7.1-21.1)	27.3 (14-45.2)	6.3 (0.8-15.1)	4.2 (0.6-10)
Dominica	0.3	216.2 (197.3-236.6)	17 (13-21.3)	36.7 (27.6-47.2)	8.9 (1.4-17.5)	5.9 (1-11.4)
Dominican Rep.	42.4	263.8 (212.7-326.5)	13.6 (12.2-15.2)	36 (28.3-45.5)	8.8 (1.3-17.5)	5.9 (0.9-11.3)
Ecuador	64.5	208.9 (192.8-228.2)	16.8 (13.8-20.5)	35.3 (28.3-43.8)	8.7 (1.2-16.8)	5.8 (0.9-10.9)
El Salvador	23.1	221.5 (178-273.8)	14.6 (11.2-17.9)	32.2 (23.5-43.4)	7.9 (1.1-16)	5.3 (0.8-10.3)
Grenada	0.4	214.6 (197.6-234.7)	18.6 (14.6-23.1)	40.1 (30.8-50.1)	9.8 (1.5-19)	6.5 (1.1-12.5)
Guatemala	82	241.8 (196.8-302.7)	14.7 (12.9-16.7)	35.7 (27.9-46)	8.7 (1.3-17.2)	5.8 (0.9-11.5)
Guyana	3.1	240 (195.1-296.9)	15.2 (9.8-21.4)	36 (22.3-53.9)	8.6 (1.1-18.7)	5.7 (0.9-12.2)
Haiti	49.5	344.2 (278.6-426.4)	16.4 (13.9-19.2)	56.7 (43.3-73.9)	10.4 (3.5-20.9)	5.9 (2-10.5)
Honduras	37.9	271 (218.5-333.6)	14.9 (13.1-16.9)	40.4 (31.3-51.7)	9.9 (1.4-19.3)	6.6 (1.1-12.8)
Jamaica	8	213.1 (195.5-233)	14.9 (11.4-18.9)	31.8 (24.2-41.2)	7.8 (1.1-15.2)	5.2 (0.8-9.9)
Mexico	467.6	224.1 (181.4-278.2)	13.2 (9.5-17.5)	29.6 (20.1-42.2)	7.2 (1-14.9)	4.8 (0.8-9.8)
Nicaragua	23.8	227 (206.6-253.7)	17.5 (14.5-21)	39.8 (32.3-49.5)	9.8 (1.5-19)	6.6 (1.1-12.3)
Paraguay	26.7	211.6 (170.1-259.8)	26.1 (20.4-31.8)	54.7 (39.8-74.1)	13.4 (1.9-27.4)	8.9 (1.4-17.6)
Peru	122.5	210.9 (170.7-260.1)	24.8 (21.1-28.8)	52.4 (40-67.9)	12.8 (1.8-25.3)	8.6 (1.4-16.6)
St. Lucia	0.5	217.3 (198.7-239)	16.6 (12.9-21.1)	36.2 (27.5-46.7)	8.9 (1.3-17.5)	5.9 (1-11.4)
St. Vincent and the Grenadines	0.3	213.6 (195.9-235.6)	16.5 (12.6-21)	35.4 (26.7-46.1)	8.6 (1.3-17.2)	5.7 (0.9-11.1)
Suriname	2	218.3 (199.1-240.6)	16.5 (12.5-21)	36.1 (27.1-46.5)	8.8 (1.3-17.5)	5.9 (0.9-11.3)
Venezuela, Bolivarian Rep.	119.1	217.6 (198.9-238.8)	16.1 (13.1-19.8)	35.3 (28.2-44.2)	8.7 (1.3-16.8)	5.8 (0.9-10.9)
**Eastern Mediterranean Region**						
Afghanistan	207.3	354.3 (285.5-438)	24.9 (23.4-26.4)	88 (70.8-109.9)	16.3 (5.5-31.7)	9 (3.1-16.2)
Djibouti	4.2	281.9 (249.5-315.9)	19 (15.8-22.8)	53.5 (43.2-65.9)	13.1 (2-25.6)	8.7 (1.4-16.7)
Egypt, Arab Rep.	543.1	233.7 (189.4-289)	25.1 (23.2-26.9)	58.5 (47.2-73.3)	14.4 (2.1-28.1)	9.6 (1.5-18.3)
Iran, Islamic Rep.	272.2	211.1 (194.3-230.1)	19.4 (15.5-23.5)	41.1 (32.1-50.6)	10.1 (1.5-19.4)	6.6 (1.1-12.7)
Iraq	232.8	243.7 (218.3-275.7)	22.1 (18.1-27.3)	54.3 (42.9-68.8)	13.4 (2-25.9)	8.9 (1.4-16.9)
Jordan	49.1	217.1 (175.3-268.1)	24.3 (21.9-26.8)	52.8 (41.6-66.8)	13 (1.9-25.1)	8.7 (1.4-16.7)
Lebanon	19.7	211.8 (193.2-234.6)	19.3 (15.2-23.9)	41.1 (31.5-52.2)	9.9 (1.5-19.8)	6.7 (1.1-12.8)
Libyan Arab Rep.	25.5	218.3 (198-245.4)	21.2 (16.5-26.5)	46.4 (34.7-60.4)	11.4 (1.7-22.2)	7.6 (1.2-14.8)
Morocco	143.7	221.9 (203.4-245.4)	22 (17.6-27)	49 (38.3-61)	12 (1.8-23.2)	8 (1.3-15.1)
Pakistan	1012.4	294.7 (239.4-366.4)	19.2 (17.2-21.2)	56.6 (45-71.7)	13.9 (2-27)	9.2 (1.4-18.1)
Palestinian Terr.	28.9	223.3 (180.4-276.9)	25.4 (21.4-29.4)	56.4 (42.9-74.4)	13.9 (2-27.7)	9.2 (1.5-18.1)
Somalia	107.5	311.1 (273.2-348.8)	17.4 (14.9-20.4)	54.1 (44.3-65.2)	10 (3.4-19.4)	5.6 (1.9-9.5)
Sudan	241.1	245.5 (228-264.8)	22 (20.2-24.8)	54.2 (48.1-62.5)	13.5 (2-25.3)	8.9 (1.4-16.8)
Tunisia	43.6	208.1 (191.1-225.9)	21 (16.3-25.5)	43.7 (33.5-54.1)	10.9 (1.6-20.8)	7.1 (1.1-13.5)
Yemen, Rep.	165.4	331.2 (268.3-412.8)	26.5 (24.8-28.2)	87.5 (70.2-110.7)	16.2 (5.3-31.5)	9 (3.1-16)
**European Region**						

Albania	7.5	209.8 (168.7-259.2)	9.8 (3.3-19.7)	20.4 (6.5-42.7)	4.6 (0.3-13.2)	3.1 (0.3-9)
Armenia	8	198 (159.3-245.3)	21.9 (12.5-37.5)	45.1 (22.1-75.8)	10.6 (1.2-25.4)	7 (1-16.6)
Azerbaijan	37.2	227.4 (184.7-280.3)	36.6 (28.5-44.7)	82.1 (59.9-110.4)	20.1 (2.8-40)	13.3 (2.1-26.9)
Belarus	23.4	208 (190-227.7)	18.8 (14.1-23.8)	39.1 (28.9-50.2)	9.6 (1.4-18.8)	6.3 (1-12.2)
Bosnia and Herzegovina	6.1	204.6 (187.2-223.4)	15.7 (11.7-20.1)	32.1 (23.7-41.6)	7.8 (1.1-15.3)	5.2 (0.8-10.2)
Bulgaria	12.5	205.4 (187.9-223.4)	17.5 (11.8-23.3)	36 (24.2-48.5)	8.6 (1.2-17.6)	5.7 (0.9-11.6)
Georgia	11	209.3 (191.9-229.2)	18.5 (13.4-23.7)	38.8 (28-50.6)	9.5 (1.4-18.7)	6.3 (1-12.4)
Kazakhstan	80.9	211.9 (194.2-232.8)	22.4 (17.6-27.8)	47.7 (36.5-60.4)	11.7 (1.7-22.9)	7.7 (1.3-14.9)
Kosovo	6	235.5 (189.9-291)	9.7 (3.2-17.7)	22.5 (7.5-44.9)	5.1 (0.4-14.5)	3.3 (0.4-9.5)
Kyrgyzstan	31.4	248.9 (202.1-308.1)	37.7 (30.4-46.4)	93.5 (69.2-125.4)	22.8 (3.1-45.8)	15.2 (2.4-30.2)
Moldova, Rep.	8.6	215.5 (196.4-237.8)	20.3 (15.8-25.6)	43.9 (33.4-56.3)	10.8 (1.6-21.1)	7.1 (1.2-13.8)
Montenegro	1.4	192.3 (154.8-238.8)	7.7 (0-23.1)	15.3 (0-52.8)	3.5 (0-15.4)	2.4 (0-10)
North Macedonia	4.8	205.4 (188.6-224.5)	17 (12.3-22.3)	35 (25-46.3)	8.4 (1.2-17.2)	5.6 (0.9-11)
Romania	36.9	206 (187.9-225)	18.1 (13-23.5)	37.3 (26.3-49.5)	9 (1.3-18.1)	6 (0.9-11.9)
Russian Fed.	393.8	211.2 (198.4-224.1)	20.7 (17.2-26.4)	43.8 (36.1-56.2)	10.8 (1.5-20.8)	7.2 (1.1-13.9)
Serbia	18.9	205.5 (188-224.5)	15.8 (11.2-20.9)	32.5 (23-43.5)	7.8 (1.1-15.9)	5.2 (0.8-10.4)
Tajikistan	48.1	299.6 (241.8-373.9)	33.8 (29.7-38.2)	101.6 (79.1-131.1)	18.6 (6.5-36.9)	10.5 (3.6-18.5)
Turkey	273	208.4 (191.3-227.7)	20.7 (16.4-25.2)	43.2 (33.6-53.5)	10.5 (1.6-20.2)	7.1 (1.1-13.3)
Turkmenistan	30.1	249.8 (202.5-307.7)	33.3 (21.4-45.2)	81.2 (49-119.8)	19.3 (2.6-42.3)	12.9 (1.9-27.3)
Ukraine	90.4	212 (193.5-234.5)	17.9 (13.5-22.8)	38 (28.3-49.3)	9.2 (1.4-18.5)	6.2 (1-12)
Uzbekistan	126.9	229.7 (209.2-256.1)	26.2 (21.4-31.9)	60.2 (48.3-75.7)	14.8 (2.2-28.4)	9.9 (1.6-18.7)
	7.5	209.8 (168.7-259.2)	9.8 (3.3-19.7)	20.4 (6.5-42.7)	4.6 (0.3-13.2)	3.1 (0.3-9)
**Southeast Asia Region**						
Bangladesh	612.1	303.2 (245.7-375.7)	18.3 (14.4-23.3)	55.9 (39.9-76.5)	13.5 (1.8-27.4)	9 (1.5-18.2)
Bhutan	2.8	244.5 (218.1-275.6)	22.8 (18.5-28.3)	56.1 (43.8-71.3)	13.8 (2-27)	9.2 (1.5-17.4)
India	4742.1	310.5 (251.4-381.6)	18.9 (18.1-19.8)	58.7 (47.6-72.7)	14.5 (2.1-27.9)	9.7 (1.6-18.2)
Indonesia	1010.2	257.5 (208.7-317.4)	28.9 (25.1-32.8)	74.3 (58.1-95)	18.1 (2.5-36)	12.1 (2-23.6)
Maldives	1.6	183.7 (147.9-226.6)	27.3 (18.2-38.2)	49.4 (30-73.5)	11.8 (1.5-25.7)	7.9 (1.3-16.6)
Myanmar	181.7	322.7 (260.7-398.3)	29.2 (23.7-34.6)	93.3 (70.2-125.1)	22.9 (3.3-46)	15.3 (2.4-30.3)
Nepal	108.2	320.1 (257.8-397)	18.4 (13.5-23.2)	58.1 (40.5-80.5)	10.6 (3.5-22.1)	5.9 (1.9-10.9)
Sri Lanka	63.1	224.5 (205.4-249.4)	23.4 (18.8-28.3)	52.9 (41.7-65.2)	12.8 (1.9-24.7)	8.6 (1.4-16)
Thailand	149.6	236.1 (190.8-292.4)	27.9 (21.8-34.2)	65.6 (47.4-89)	15.9 (2.2-32.5)	10.6 (1.7-21.1)
Timor-Leste	8.3	292.8 (236.6-360.9)	30.8 (25.9-36.8)	90.4 (68.5-118.6)	22.1 (3.2-44)	14.7 (2.4-28.9)
**Western Pacific Region**						
American Samoa	0.2	211.3 (192.6-235.5)	18.4 (13-24.4)	39 (26.6-53.7)	9.4 (1.3-19.6)	6.3 (1-12.9)
Cambodia	70.2	336.2 (273-415.8)	29.5 (24.8-34.8)	99 (75.2-130.1)	24.3 (3.4-48.3)	16.1 (2.5-31.4)
China, People's Rep.	3454.2	217.4 (197.3-241)	19.6 (15.6-24.1)	42.7 (33.2-53.7)	10.4 (1.5-20.4)	6.9 (1.1-13.3)
Fiji	3.4	217.9 (198.5-242.1)	21.6 (16.9-26.2)	46.9 (36.1-58.3)	11.4 (1.7-22.1)	7.6 (1.2-14.7)
Kiribati	0.6	251.3 (222.1-283.3)	19.9 (15.6-24.4)	49.8 (38.3-63)	12.3 (1.7-23.6)	8.1 (1.4-15.5)
Lao People's Dem. Rep.	30.6	275.3 (222.4-341.2)	20.5 (17.1-24.4)	56.6 (42.8-74.6)	13.9 (2-27.6)	9.2 (1.5-18.1)
Malaysia	105.4	209.1 (192.6-227.1)	22.6 (18.1-28.4)	47.4 (36.9-60.6)	11.6 (1.7-22.8)	7.7 (1.2-15)
Marshall Islands	0.2	233 (206.5-263)	15.7 (11.8-20.1)	36.7 (27.1-47.6)	8.9 (1.2-17.2)	5.9 (1-11.5)
Micronesia, Fed.	2	253 (224.4-287)	18.9 (14.8-23.1)	47.7 (36.4-60.8)	11.6 (1.6-22.7)	7.7 (1.3-14.8)
Mongolia	15.3	261.7 (211.7-323.9)	24.8 (20.6-29.1)	64.5 (48.5-84.9)	15.8 (2.2-31.4)	10.5 (1.7-20.5)
Nauru	<0.1	234.6 (209.6-265.8)	20.1 (15.9-25.4)	47.5 (36.4-62)	11.7 (1.7-22.8)	7.7 (1.3-14.9)
Papua New Guinea	41.5	285.7 (252.6-319.5)	20.5 (17.2-24.7)	58.7 (47.2-72.6)	14.4 (2.1-27.9)	9.6 (1.5-18.1)
Philippines	464.4	247.4 (197.9-305.1)	28.5 (23.3-33.6)	70 (52.2-92)	17.1 (2.4-33.9)	11.4 (1.8-22.3)
Samoa	0.9	224.4 (200.7-252.6)	19.7 (14.8-24.4)	44 (32.6-56.5)	10.7 (1.5-21.3)	7.1 (1.2-13.8)
Solomon Islands	3.3	273.9 (241.3-310.2)	20.3 (15.7-25.2)	55.4 (42.3-70.6)	13.6 (2-27)	9 (1.4-17.3)

Tonga	0.5	225.4 (203.3-252.1)	18.6 (13.6-23.8)	42 (30.5-54.6)	10.1 (1.5-20.4)	6.7 (1.1-13.1)
Tuvalu	<0.1	221.2 (200-248.5)	16.2 (12.3-20.6)	36.1 (26.7-46.9)	8.8 (1.2-17.4)	5.9 (1-11.4)
Vanuatu	1.4	247.5 (220.5-279.6)	19.7 (15.4-24.6)	48.9 (37.5-62.3)	12 (1.8-23.8)	7.9 (1.3-15.3)
Viet Nam	311.4	249.5 (201.1-309.9)	29.1 (22.2-36.8)	72.9 (51.1-102.1)	17.6 (2.4-36.5)	11.8 (1.8-24)

Incidence rate estimates are presented as cases per 100 children annually in the eligible age range. Estimates are obtained from analyses of DHS/MICS surveys covering 944,173 children across 77 countries, as well as extrapolations based on 405 health, nutrition, and population indicators for all LMICs. Quantiles are obtained via 5,000 independent draws from the distribution of estimates.

Table S14: Upper-bound country-specific estimates of the incidence of ARI and antibiotic-treated ARI among children ages 24-59 months preventable by 10- and 13-valent pneumococcal conjugate vaccines.

Country	Population ages 24-59 months	ARI attributable to all causes			ARI attributable to PCV10/13-type *Streptococcus pneumoniae*		
	Children (in 10,000s)	All cases, per 100 (95% CI)	Proportion treated with antibiotics, % (95% CI)	Antibiotic-treated cases, per 100 (95% CI)	Antibiotic-treated cases, per 100 (95% CI)		Antibiotic-treated cases preventable by vaccine direct effects, per 100 (95% CI)
					IPD probe estimate	*AOM probe estimate*	
**African Region**							
Algeria	275	188.8 (174.7-204.6)	46 (38-56.4)	87 (70.8-107.6)	21.5 (3.5-49)	31.1 (5.1-70.6)	17.2 (3-38.5)
Angola	300.8	218.1 (204.6-232.1)	35.1 (27-43.5)	76.5 (58.6-95.7)	18.7 (3.2-42.9)	27.1 (4.7-61)	15.1 (2.7-33.2)
Benin	101.4	303.6 (284.3-323.5)	40.4 (30.8-50)	122.5 (93-153.6)	30.3 (5-68.7)	43.8 (7.3-99.1)	24.1 (4.2-53.9)
Botswana	15	199.7 (183.5-217.8)	41.3 (34.3-49.4)	82.6 (68.3-98.6)	20.5 (3.6-46.1)	29.7 (5.3-66.7)	16.4 (3-36.7)
Burkina Faso	185.1	324.6 (302.1-347.3)	40 (31.3-48.5)	129.6 (100.9-159)	31.9 (5.3-71.6)	46.1 (7.8-103)	25.5 (4.4-56.4)
Burundi	107.4	353 (323.5-384.4)	42.7 (37-48.3)	150.5 (128.6-174.9)	37.4 (6.4-82.3)	54.1 (9.3-118.3)	29.8 (5.3-65.5)
Cabo Verde	3.3	207.4 (189.9-226.8)	45.5 (37.8-54.7)	94.4 (78.8-113.4)	23.3 (4-52)	33.7 (5.8-75)	18.7 (3.3-41.5)
Cameroon	219.1	274 (259.4-289.4)	42.3 (36-48.7)	115.6 (97.5-134.6)	28.7 (5-64)	41.5 (7.3-92.7)	22.9 (4-51.1)
Cent. African Rep.	42.6	306.4 (278.4-335.2)	38.5 (33.3-44.4)	118.2 (99.9-137.4)	29.3 (5.3-63.9)	42.3 (7.7-92.1)	23.5 (4.3-50.3)
Chad	150.4	310.3 (291.3-330.9)	39.7 (35.6-44.1)	123.1 (108.4-140.1)	30.6 (5.2-67.2)	44.1 (7.5-97.4)	24.5 (4.2-53.3)
Comoros	6.9	304.2 (283.1-324.6)	40.5 (25.6-55.3)	122.7 (78.2-169.8)	30 (4.9-69.7)	43.4 (7.1-99.8)	23.8 (4.1-55.2)
Congo, Rep.	47.9	249.6 (236.5-262.7)	40.5 (33.5-47.3)	100.9 (82.8-119.4)	25 (4-55.8)	36.1 (5.9-80.7)	20.1 (3.4-44.1)
Congo, Dem. Rep.	814.3	324.8 (301.4-348.9)	43.6 (38.7-48.2)	141.1 (123.7-159.7)	34.9 (6.1-76.9)	50.5 (8.9-110.9)	27.9 (5-61.2)
Cote d'Ivoire	218.2	279.3 (263.7-296)	37.7 (29.9-46)	105.1 (82.4-129.8)	25.9 (4.3-58)	37.5 (6.3-83.9)	20.7 (3.7-45.5)
Equatorial Guinea	10.2	199.5 (179.8-223.5)	42 (33.3-52.4)	83.7 (66.4-104.5)	20.5 (3.7-46.4)	29.8 (5.4-66.9)	16.6 (3.1-36.9)
Eritrea	44.7	288.3 (249.8-331.3)	45.3 (37.7-55.2)	131.4 (106.5-159.6)	32.6 (5.7-72.1)	47.2 (8.3-103.5)	26.1 (4.6-57.6)
Eswatini	10.6	236.7 (222.1-252.3)	37.7 (29-47.7)	89.7 (68.1-113.6)	22.2 (3.7-50)	32 (5.4-72.5)	17.7 (3.1-39.5)
Ethiopia	870.8	309.5 (290.5-329.6)	37 (30.3-44.2)	115.2 (93.1-138.5)	28.5 (5.1-63.6)	41.2 (7.5-91.8)	22.8 (4.1-50)
Gabon	15.5	180.1 (165.7-193.5)	35.4 (28.8-42)	63.6 (51-77.1)	15.6 (2.6-34.7)	22.6 (3.8-49.9)	12.5 (2.2-27.5)
Gambia, The	20.4	308.5 (288.1-329.1)	42.1 (34.8-49.7)	129.8 (105.3-155.8)	32.1 (5.6-71.6)	46.5 (8.1-102.9)	25.8 (4.7-55.8)
Ghana	235.8	259.9 (246.7-274.2)	43.9 (33.7-54.1)	114.1 (87.5-141.9)	28 (4.8-63.3)	40.5 (6.9-91.3)	22.3 (3.9-50.4)
Guinea	114	314.2 (293.5-335.3)	39.4 (32.2-46.9)	123.4 (99.4-148.6)	30.5 (5.2-69.5)	44.3 (7.6-99.4)	24.6 (4.3-53.6)
Guinea-Bissau	16.6	232.7 (215.6-251.6)	41.3 (32.1-50.5)	95.8 (73.4-119.8)	23.5 (4-53.3)	34 (5.8-76.4)	18.8 (3.2-42.1)
Kenya	419.3	269 (255.3-283.3)	38.8 (35.2-42.6)	104.5 (93.5-116.1)	25.9 (4.5-56.8)	37.5 (6.6-82.4)	20.9 (3.6-45.8)
Lesotho	16.3	261.2 (245.9-277.5)	40.8 (29.6-52.8)	107.4 (77.1-138.7)	26.3 (4.6-60.1)	38.1 (6.6-86.7)	21 (3.8-47.2)
Liberia	41.4	305 (284.9-325.3)	41.9 (35.7-48.7)	127.7 (107.1-150.2)	31.7 (5.5-70)	45.9 (8.1-101.2)	25.4 (4.5-55.4)
Madagascar	217.6	301.4 (277.5-327.9)	46.7 (39.8-54.5)	140.8 (119.2-164.6)	35 (6-77)	50.6 (8.7-111.7)	28 (5-61.3)
Malawi	173.1	347.2 (318.1-377.7)	44.9 (39.6-50.4)	155.8 (135-179.3)	38.8 (6.8-85.1)	56.1 (9.9-122.8)	31.1 (5.5-67.2)
Mali	191.5	312 (291.9-332.4)	33.7 (24.1-44.4)	106.4 (74.6-139.9)	26 (4.3-60.2)	37.6 (6.3-87.5)	20.6 (3.5-47.3)
Mauritania	37.3	236.5 (222.8-251.7)	38.8 (32.1-45.2)	91.4 (75.2-108.8)	22.7 (3.8-50.6)	32.8 (5.5-72.2)	18.2 (3.2-40)
Mauritius	4.2	190.9 (175.8-207.6)	43.6 (35.4-53.1)	83.2 (66.9-102.7)	20.5 (3.5-46.9)	29.8 (5-67.3)	16.4 (2.8-36.4)

Mozambique	282.1	337.4 (311.3-363.1)	41.9 (34.2-50.3)	141.6 (113.7-171.3)	35.1 (5.9-77.4)	50.9 (8.6-111.6)	28 (4.9-61.9)
Namibia	19.3	209.1 (195.4-223.5)	35.3 (27.6-43.9)	73.8 (56.5-92.5)	18.2 (3-41.2)	26.3 (4.4-59.5)	14.6 (2.5-32.9)
Niger	232.8	347.2 (320-375.2)	40.6 (33.3-47.8)	140.7 (114.6-169)	34.7 (6-79)	50.1 (8.6-113.7)	27.8 (4.8-62.1)
Nigeria	1804.2	266.8 (253-280.8)	43 (36.3-50)	114.8 (95.9-135.2)	28.4 (4.9-63.3)	41.1 (7.1-90.4)	22.7 (4-49.3)
Rwanda	103.6	314.9 (294.6-335.8)	41.9 (34.6-49)	132 (108.4-155.7)	32.7 (5.6-72.6)	47.1 (8.2-104.8)	26 (4.5-57.4)
Sao Tome and Principe	1.8	268.6 (251.4-286.9)	43.5 (32.9-54.1)	116.2 (86.9-147.2)	28.5 (4.6-65.4)	41.2 (6.7-94.5)	22.8 (3.8-51.4)
Senegal	145.7	278.3 (262.3-294.8)	38.9 (32.2-45.8)	108.2 (88.7-129.3)	26.7 (4.6-59.3)	38.7 (6.6-85.6)	21.3 (3.8-46.8)
Sierra Leone	67.3	333.1 (308-358)	41.3 (35.8-46.7)	137.3 (117.5-158.2)	33.9 (5.7-75.2)	49.1 (8.3-107.6)	27.3 (4.7-59.7)
South Africa	336.1	180.5 (167-193.9)	35.4 (21.7-50)	63.9 (39.3-91.9)	15.5 (2.5-37.3)	22.5 (3.6-53.6)	12.4 (2-29.3)
South Sudan	110	285.8 (250.7-323.2)	40.7 (33.5-50.7)	117.8 (95.4-139.8)	29 (5-65)	42 (7.2-93.9)	23.2 (4.1-51.2)
Tanzania, United Rep.	547.9	300.2 (282.4-318.2)	40.7 (32-49.6)	122.3 (95.5-150.1)	30.1 (5.2-67.9)	43.5 (7.6-97.9)	24.1 (4.3-52.9)
Togo	68.6	314.7 (293.4-337)	40.9 (32.5-50)	129.3 (101.8-159.6)	31.8 (5.2-73.1)	46 (7.6-105)	25.4 (4.4-57.5)
Uganda	436.5	309.7 (289.6-330.3)	49.2 (43.7-55.5)	152.5 (132.9-174.5)	38 (6.6-84.7)	55 (9.6-122)	30.4 (5.4-67.3)
Zambia	160.1	274.7 (260.6-290.2)	39.1 (32.9-45.6)	107.2 (89.6-126.5)	26.7 (4.6-59)	38.6 (6.7-84.5)	21.4 (3.8-46.7)
Zimbabwe	146.6	261.4 (246.7-276.1)	41.4 (32.4-51.4)	108.5 (84.3-135.3)	26.8 (4.7-59.8)	38.7 (6.9-86.1)	21.4 (3.9-47.6)
**Region of the Americas**							
Belize	2.3	212.6 (199-227.1)	37.7 (24.6-49.2)	78.8 (52.6-106.3)	19.4 (3.3-45.5)	28 (4.8-65.4)	15.4 (2.7-35.2)
Bolivia, Plurinational State	70.7	215.6 (203.2-228.8)	69.4 (59.9-79.4)	149.5 (128.1-173.6)	37.1 (6.5-82.2)	53.7 (9.4-118.2)	29.7 (5.2-65.2)
Brazil	884.7	187.3 (174-202)	42.4 (35.6-50.1)	79.4 (66.1-94.8)	19.7 (3.4-43.7)	28.4 (5-63.3)	15.7 (2.9-34.8)
Colombia	225.6	178.6 (165.9-190.9)	38 (33.4-42.6)	67.7 (58.3-77.3)	16.8 (2.9-37.2)	24.3 (4.2-53.4)	13.5 (2.4-29.4)
Costa Rica	21.4	183.7 (170.7-197.5)	41.9 (34.8-50)	77.2 (63.5-92)	19.1 (3.2-43.4)	27.6 (4.7-62.8)	15.2 (2.7-34.5)
Cuba	36.9	186.2 (173-199.8)	31.9 (25.6-38.4)	59.3 (47.2-72.4)	14.7 (2.4-33.1)	21.2 (3.5-47.5)	11.7 (2-26.1)
Dominica	0.4	190.3 (175.2-207.3)	43.3 (35.5-51.8)	82.5 (66.8-100.1)	20.3 (3.4-46.6)	29.3 (4.9-66.9)	16.2 (2.8-36.6)
Dominican Rep.	63.8	197.3 (184.1-211)	32.2 (28.7-36)	63.4 (55.5-72.4)	15.8 (2.7-34.9)	22.8 (3.9-50.3)	12.6 (2.2-27.6)
Ecuador	96.5	186.9 (173.8-200.9)	42.6 (36.6-49.6)	79.6 (67.5-93.2)	19.8 (3.5-44)	28.6 (5-63.2)	15.8 (2.8-35)
El Salvador	34.9	198.7 (186.7-212.4)	35.1 (29.8-40.6)	69.8 (58.4-82.2)	17.5 (3-38.8)	25.3 (4.3-55.6)	14 (2.4-30.4)
Grenada	0.6	191.3 (175.8-208.4)	45.1 (35.5-55.3)	86.1 (67.9-106.8)	21.3 (3.7-47.6)	30.8 (5.3-68.5)	17 (3-37.6)
Guatemala	117.4	231.3 (217.5-246.2)	38.1 (33.9-42.4)	88 (77.5-99.7)	21.9 (3.7-48.1)	31.6 (5.4-69.7)	17.5 (3.1-38.3)
Guyana	4.4	208.4 (193.2-223.3)	39.5 (28.2-51.3)	81.8 (58.8-106.7)	20.1 (3.3-46.5)	29.1 (4.8-66.6)	16.1 (2.8-35.9)
Haiti	74.2	296.7 (278-315.7)	41.5 (35.5-47.9)	123.2 (103.9-143.7)	30.7 (5.3-68)	44.4 (7.6-98.3)	24.5 (4.4-54)
Honduras	57.6	237 (224-250.1)	41 (36.8-45.4)	97.1 (86.4-108.9)	24.2 (4.1-52.9)	35.1 (5.9-76.3)	19.4 (3.3-42.2)
Jamaica	12.6	190.9 (176.6-206.6)	41 (34.5-48.1)	78.1 (65.7-92.8)	19.3 (3.4-43.1)	28 (4.9-61.9)	15.5 (2.8-34)
Mexico	685.7	185.4 (171.8-199.9)	45.4 (35.3-56.3)	84.1 (65.2-105.3)	20.8 (3.4-47.5)	30.1 (5-68.3)	16.7 (2.8-37.3)
Nicaragua	36.8	214.2 (196.7-234)	45.9 (37.7-55.9)	98.4 (80.9-119.7)	24.6 (4.3-54.5)	35.5 (6.2-78.1)	19.6 (3.4-42.6)
Paraguay	40.6	182.8 (170.2-195.6)	57.3 (49.5-65.4)	104.8 (88.6-121.4)	25.9 (4.5-56.9)	37.5 (6.5-81.9)	20.7 (3.6-45.4)
Peru	179.5	189.6 (176.7-202.4)	59.3 (51-67.5)	112.4 (94.6-130)	27.8 (4.7-60.8)	40.2 (6.9-87.3)	22.3 (3.8-48.8)
St. Lucia	0.6	190.8 (174.5-208.2)	42.7 (34.5-51.2)	81.4 (65.8-98.2)	20.1 (3.6-45.7)	29 (5.2-65.8)	16 (2.9-35.9)
St. Vincent and the Grenadines	0.5	194 (178-210.8)	42 (34.7-50.2)	81.6 (66.9-98.2)	20.2 (3.5-45.6)	29.2 (5.1-65.6)	16.1 (2.9-35.9)
Suriname	3	193.3 (177.9-213)	42.7 (35.4-51.5)	82.7 (67.7-100.6)	20.5 (3.5-45.9)	29.7 (5.1-66.4)	16.5 (2.9-36.3)
Venezuela, Bolivarian Rep.	178.4	187.3 (173.3-204.1)	44.7 (36.7-53.8)	83.8 (67.8-101.8)	20.7 (3.6-46.5)	29.9 (5.2-67.2)	16.6 (3-36.4)
**Eastern Mediterranean Region**							
Afghanistan	316.6	314.4 (292-337.2)	61.9 (58.5-65.3)	194.4 (178-212.4)	48.6 (8.3-105.3)	70.3 (12-152.7)	38.8 (6.7-85)

Djibouti	5.9	224.9 (203.7-249.4)	45.2 (36.8-54.5)	101.6 (83.3-121.5)	25.2 (4.5-55.8)	36.3 (6.6-80.4)	20.2 (3.6-44.3)
Egypt, Arab Rep.	694.3	228.8 (217.3-240.6)	58.3 (54.8-62)	133.4 (123.3-144.5)	33.3 (5.4-72.5)	48.2 (7.9-104.7)	26.5 (4.4-57.8)
Iran, Islamic Rep.	417.8	187.2 (173.8-202.1)	47 (39.6-55.3)	88.1 (73.2-104.5)	21.7 (3.7-49.4)	31.4 (5.4-70.9)	17.4 (3-38.8)
Iraq	327.5	205.4 (187.1-227.9)	47.2 (38.9-57.1)	97.1 (79.6-118.1)	24.1 (4.1-53.5)	34.8 (5.9-76.7)	19.3 (3.3-42.5)
Jordan	71.1	176.5 (165-188.2)	55.9 (50.1-61.7)	98.5 (86.5-111.2)	24.4 (4.2-53.5)	35.4 (6.1-77.3)	19.5 (3.4-42.7)
Lebanon	26.4	182.2 (167.3-198.6)	48.1 (40.2-57.2)	87.7 (73.4-104.4)	21.6 (3.7-48.5)	31.2 (5.3-69.7)	17.3 (3.1-38.6)
Libyan Arab Rep.	37.5	197.4 (177.2-227.3)	47.3 (38-58.6)	94 (75.5-116.1)	23.2 (3.9-52.5)	33.7 (5.7-75.8)	18.6 (3.3-41.2)
Morocco	201.9	209.9 (192.7-229.2)	49.1 (40.3-59.3)	103.3 (85.1-124.7)	25.4 (4.4-57.1)	36.7 (6.4-82.3)	20.2 (3.6-45.1)
Pakistan	1458.8	256.2 (242.9-270.3)	53.1 (49-57.1)	135.9 (123.2-149)	34.1 (5.8-74.2)	49.3 (8.4-106.3)	27.2 (4.7-58.8)
Palestinian Terr.	41	215.5 (203.3-228.5)	53.5 (48.8-58.5)	115.2 (103.4-128.3)	28.7 (4.9-62.4)	41.4 (7.2-89.9)	23 (4-50.2)
Somalia	147.9	250.5 (220.3-297.5)	43.5 (35.4-53.4)	110.2 (90.6-131.1)	27.3 (4.6-60.7)	39.3 (6.7-87.6)	21.7 (3.8-48.1)
Sudan	344.8	262.7 (235.8-288.3)	43.2 (36.4-50.8)	112.8 (95.6-133.1)	28 (4.9-61.7)	40.6 (7.1-89)	22.4 (4-48.9)
Tunisia	58.8	187.8 (174-203.5)	48.4 (40.2-57.8)	91 (75-109.8)	22.5 (3.9-51.1)	32.5 (5.7-73.3)	18 (3.1-39.6)
Yemen, Rep.	236.3	257.5 (243.2-273.1)	62.3 (58.4-66.1)	160.4 (147.4-173.5)	40 (6.8-87.6)	57.9 (10-126.7)	32 (5.5-70.2)
**European Region**							
Albania	9.7	197.4 (184.2-212.2)	48.6 (29.7-67.6)	95.2 (57.3-136.7)	23.1 (3.8-56)	33.3 (5.5-80.7)	18.4 (3-43.3)
Armenia	12.6	176.6 (162.8-191.4)	57.9 (36.8-78.9)	103.4 (62-143.8)	24.8 (4-59.3)	35.9 (5.8-85.2)	19.8 (3.3-46.5)
Azerbaijan	49.5	189.3 (175.2-202.8)	40 (24-58.5)	76.2 (44.8-112)	18.5 (3-46.2)	26.7 (4.4-66.3)	14.7 (2.5-36.1)
Belarus	34.2	184.5 (171.3-199.2)	41.7 (33.8-50.1)	77.2 (61.7-93)	19.1 (3.2-42.7)	27.5 (4.7-61.2)	15.2 (2.6-33.8)
Bosnia and Herzegovina	10.1	183.7 (168.3-202.1)	43.4 (35.4-53)	80 (63.7-99.3)	19.8 (3.4-44.6)	28.5 (4.9-64.1)	15.8 (2.8-34.8)
Bulgaria	21.1	182.2 (169.2-196.6)	42.7 (34.2-51.5)	77.7 (62.2-95.2)	19.2 (3.3-43.7)	27.7 (4.8-63.2)	15.4 (2.7-33.9)
Georgia	16.6	188.7 (174.5-203.5)	43.3 (35-53.4)	81.8 (65.7-101.7)	20.2 (3.4-45.8)	29.3 (4.8-65.6)	16.2 (2.8-35.8)
Kazakhstan	118.2	185.9 (172-201.1)	44.9 (36.3-53.7)	83.4 (67.2-100.3)	20.7 (3.4-46.3)	29.9 (4.9-66.4)	16.4 (2.8-36.8)
Kosovo	9	194.4 (180.9-208.8)	49.5 (34.7-66.3)	96.2 (67.5-129.1)	23.7 (4-55.2)	34.3 (5.8-79.8)	18.9 (3.2-43.8)
Kyrgyzstan	43.4	226.7 (212.8-242.1)	43.5 (21.7-68)	99.7 (48.6-154)	23.9 (3.4-60.6)	34.5 (4.9-87.9)	18.9 (2.8-47.4)
Moldova, Rep.	13.6	200 (182.4-218.8)	46.6 (37.5-57.4)	93.1 (74.4-114.6)	23 (3.9-51.5)	33.3 (5.7-73.7)	18.4 (3.1-40.4)
Montenegro	2.3	182 (167.7-197.1)	40 (20-70)	76 (34.1-127)	17.8 (2.5-49)	25.7 (3.7-70.3)	14.2 (2-37.8)
North Macedonia	6.8	182.9 (168.4-198.6)	44.9 (35.9-55.5)	82 (64.5-102.5)	20.2 (3.5-45.9)	29.1 (5-65.8)	16.2 (2.8-36.3)
Romania	60.3	186.7 (172.2-201.4)	42.7 (34.6-51.3)	79.7 (63.3-96.5)	19.7 (3.3-44)	28.5 (4.7-63.5)	15.7 (2.7-34.8)
Russian Fed.	541.7	182 (168.5-196.4)	43.3 (35.5-51.8)	78.8 (64.4-94.3)	19.5 (3.3-43.8)	28.2 (4.8-63)	15.5 (2.8-34.4)
Serbia	27.9	183.8 (170.2-198.3)	42.2 (33.8-51.9)	77.4 (61.6-96.4)	19.2 (3.3-44.2)	27.7 (4.8-63.5)	15.3 (2.7-34)
Tajikistan	68.4	242.5 (226.8-259.8)	55.6 (33.3-77.8)	140.6 (81.8-196.3)	34 (5.2-81.8)	49.3 (7.6-117.2)	27.1 (4.3-63.7)
Turkey	401	186.8 (173.2-202.1)	46.1 (37.9-55.1)	86.2 (70.2-104.9)	21.2 (3.8-48.4)	30.8 (5.5-69.7)	17 (3.1-38)
Turkmenistan	37.8	172.2 (159.7-185.2)	30 (10-70)	56.1 (16.1-120)	13.8 (0-44.1)	19.9 (0-63.3)	10.9 (0-34.1)
Ukraine	150.6	194.7 (178.7-211.7)	43.9 (35.6-53.2)	85.4 (69.5-104.5)	21.2 (3.5-47.4)	30.6 (5.1-68.4)	16.9 (2.9-37.3)
Uzbekistan	192.8	214.7 (194.9-235.3)	47.5 (37.5-58.8)	102.1 (81.3-125.7)	25.1 (4.2-55.9)	36.4 (6.1-80.8)	20.1 (3.4-43.7)
**Southeast Asia Region**							
Bangladesh	913.3	265.7 (251.6-280)	53.5 (46.5-60.5)	142.1 (122-162.4)	35.2 (6.2-78.5)	50.9 (9-113.4)	28.1 (5-62.9)
Bhutan	4.3	232.7 (209.7-258.2)	56.9 (46.3-69.2)	132.3 (108.6-159.5)	32.9 (5.6-72.8)	47.7 (8.1-104.9)	26.3 (4.5-57.7)
India	7399.5	248.1 (236.3-260.2)	51.5 (48.9-54.4)	127.8 (119.1-137.4)	32 (5.4-69.8)	46.3 (7.9-100.4)	25.5 (4.3-55.8)
Indonesia	1449.1	209.5 (197.5-221.6)	64 (56-72.2)	133.9 (115.7-153.2)	33.2 (5.6-73.4)	48.1 (8.1-105.9)	26.7 (4.7-58.7)
Maldives	2.2	162.5 (146.5-177.8)	57.9 (35-80)	94.9 (57-132.6)	22.7 (3.7-55)	32.9 (5.4-79.8)	18.1 (3-43.6)

Myanmar	273.6	278.5 (263.3-294.9)	66.9 (55.6-78.4)	186.7 (153.5-220.1)	46.4 (7.7-103)	67.1 (11.2-148.6)	37 (6.4-81.1)
Nepal	172.3	276.5 (260-294.5)	53.3 (37.8-68.9)	148 (105.3-192.5)	36 (6.3-82.1)	52.1 (9.1-118.4)	28.7 (5-65)
Sri Lanka	101.2	205.8 (188.3-224.7)	55.5 (45.1-67.9)	114.1 (92.6-140)	28.2 (4.8-63.4)	40.9 (7-91.2)	22.5 (4.1-49.7)
Thailand	234.7	207.1 (193.6-221.8)	58 (46.8-69.6)	120.2 (95.2-146.2)	29.5 (5.1-66.3)	42.7 (7.5-95.2)	23.7 (4.2-53.1)
Timor-Leste	12.1	254.8 (241-268.8)	65.5 (50.9-79.7)	167.4 (129.4-205.3)	41.5 (6.8-93)	59.9 (9.8-134.4)	32.9 (5.6-73.4)
**Western Pacific Region**							
American Samoa	0.3	196.8 (177.2-216.7)	47.3 (37.5-60.4)	92.6 (73.4-119)	23 (3.9-52.5)	33.3 (5.7-75.5)	18.3 (3.2-41)
Cambodia	106.2	278.1 (263-293.8)	68 (57.4-79.1)	188.9 (158.1-221.1)	46.5 (8.1-104.4)	67.4 (11.6-150.4)	37.2 (6.5-82.4)
China, People’s Rep.	5134.2	189 (174.6-205.6)	47.9 (38.9-58.3)	90.7 (73.5-110.7)	22.6 (3.8-50.1)	32.6 (5.6-72.1)	18 (3.2-39.8)
Fiji	5.4	196.8 (181.2-213.3)	46.3 (37.9-56.1)	91.1 (74-111.8)	22.5 (3.8-51)	32.4 (5.5-73.4)	17.9 (3.1-39.9)
Kiribati	0.9	223.4 (202.7-246.3)	47.7 (38.5-59.5)	106.6 (86.2-133.1)	26.4 (4.7-60.4)	38.2 (6.9-86.8)	21.1 (3.9-47.5)
Lao People’s Dem. Rep.	46.6	261.3 (247.3-276.6)	69.2 (55.4-82.8)	181.4 (142.8-218.8)	44.8 (7.6-100.4)	64.7 (11-145)	35.8 (6.1-79.2)
Malaysia	151.6	186.4 (172.1-200.8)	48.4 (40.2-58.5)	90.3 (74.4-109.6)	22.4 (3.8-51.3)	32.4 (5.6-73.5)	17.9 (3.1-39.8)
Marshall Islands	0.3	200 (182-220.4)	41.9 (34.3-51.8)	83.8 (67.8-103.8)	20.7 (3.7-47.3)	30 (5.4-67.1)	16.7 (3.1-37.1)
Micronesia, Fed.	3.1	228.1 (207.7-252.1)	49.1 (39.1-61.2)	112 (90.1-139.1)	27.7 (4.7-63)	40.2 (6.9-90.3)	22.3 (3.8-48.9)
Mongolia	20.6	198 (186-211.1)	51.1 (42.2-60.4)	101.3 (82.8-121.3)	25.1 (4.3-56)	36.3 (6.3-80.5)	20 (3.5-44)
Nauru	0.1	202.3 (183.7-222.7)	48 (38.6-61.6)	96.7 (77-126.7)	24.2 (4.1-55.4)	35.1 (5.9-80)	19.3 (3.5-43.5)
Papua New Guinea	60.9	254.8 (231.3-281.1)	48.8 (40-59.1)	124.3 (103.1-149.7)	31 (5.3-69.6)	44.8 (7.7-100.4)	24.8 (4.4-54.3)
Philippines	679.1	215.7 (204.6-227.1)	63.5 (52.1-75.5)	136.9 (110.6-163.9)	34.1 (5.7-75.5)	49.2 (8.3-109)	27.2 (4.7-59.4)
Samoa	1.5	205 (187.5-224.6)	48 (39.4-58.8)	98.4 (81-120.4)	24.4 (4.2-54.6)	35.4 (6.1-79.1)	19.6 (3.5-43.3)
Solomon Islands	5	257.2 (232.6-283.2)	47.9 (39.3-58.8)	123.1 (101.2-150.6)	30.6 (5.2-68.3)	44.3 (7.6-98)	24.6 (4.3-53.6)
Tonga	0.8	213.8 (195.4-234)	52.8 (41.3-67.2)	112.7 (88.9-143)	28.1 (4.8-63.9)	40.6 (7-91.9)	22.5 (4-50.1)
Tuvalu	0.1	192.1 (175.1-210.2)	44.6 (36.9-53.4)	85.6 (71.3-102.7)	21.2 (3.7-47.6)	30.6 (5.3-68.8)	16.9 (2.9-37.9)
Vanuatu	2.1	247.8 (222.5-276.8)	46.2 (36.9-58)	114.5 (93.4-141.2)	28.2 (4.9-64.2)	40.8 (7.1-91.7)	22.5 (3.9-50.6)
Viet Nam	463.9	220.9 (208.4-234.5)	59.6 (47.3-72)	131.7 (104.4-160)	32.3 (5.6-73.2)	46.8 (8.1-105.8)	18.3 (3.2-41)

Incidence rate estimates are presented as cases per 100 children annually in the eligible age range. Estimates are obtained from analyses of DHS/MICS surveys covering 944,173 children across 77 countries, as well as extrapolations based on 405 health, nutrition, and population indicators for all LMICs. Quantiles are obtained via 5,000 independent draws from the distribution of estimates.

Table S15: Lower-bound country-specific estimates of the incidence of ARI and antibiotic-treated ARI among children ages 24-59 months preventable by 10- and 13-valent pneumococcal conjugate vaccines.

Country	Population ages 24-59 months	ARI attributable to all causes			ARI attributable to PCV10/13-type *Streptococcus pneumoniae*		
	Children (in 10,000s)	All cases, per 100 (95% CI)	Proportion treated with antibiotics, % (95% CI)	Antibiotic-treated cases, per 100 (95% CI)	Antibiotic-treated cases, per 100 (95% CI)		Antibiotic-treated cases preventable by vaccine direct effects, per 100 (95% CI)
					IPD probe estimate	*AOM probe estimate*	
**African Region**							
Algeria	275	57.4 (52.7-62.5)	46 (38-56.4)	26.4 (21.6-32.5)	6.5 (1.1-15.1)	9.5 (1.5-21.6)	5.2 (0.9-11.8)
Angola	300.8	66.4 (62.4-70.4)	35.1 (27-43.5)	23.4 (17.9-29.1)	5.7 (1-12.9)	8.3 (1.4-18.6)	4.6 (0.8-10.2)
Benin	101.4	92.3 (85.9-99.4)	40.4 (30.8-50)	37.3 (28.4-46.8)	9.2 (1.6-20.9)	13.3 (2.3-30)	7.3 (1.3-16.3)
Botswana	15	60.8 (55.3-66.9)	41.3 (34.3-49.4)	25.1 (20.6-30.4)	6.2 (1.1-14.2)	9 (1.6-20.4)	5 (0.9-11.1)
Burkina Faso	185.1	98.6 (91.6-106.5)	40 (31.3-48.5)	39.4 (30.6-48.6)	9.7 (1.6-21.8)	14.1 (2.4-31.3)	7.7 (1.3-17.2)
Burundi	107.4	107.4 (97.4-117.8)	42.7 (37-48.3)	45.8 (39-53.5)	11.3 (2-25.3)	16.4 (2.9-36.1)	9.1 (1.6-19.9)
Cabo Verde	3.3	63.2 (57.4-69.8)	45.5 (37.8-54.7)	28.8 (24-34.6)	7.1 (1.2-16)	10.3 (1.8-23.1)	5.7 (1-12.6)
Cameroon	219.1	83.4 (78.6-88.6)	42.3 (36-48.7)	35.2 (29.6-41.3)	8.7 (1.5-19.4)	12.6 (2.2-27.9)	7 (1.2-15.6)
Cent. African Rep.	42.6	93.3 (83.9-102.7)	38.5 (33.3-44.4)	35.9 (30.5-41.9)	8.9 (1.6-19.8)	12.9 (2.3-28.2)	7.1 (1.3-15.4)
Chad	150.4	94.4 (88.3-101.2)	39.7 (35.6-44.1)	37.5 (33.3-42.3)	9.3 (1.6-20.5)	13.4 (2.3-29.6)	7.4 (1.3-16.1)
Comoros	6.9	92.5 (85.2-100.4)	40.5 (25.6-55.3)	37.3 (23.6-51.8)	9.2 (1.5-21.4)	13.2 (2.2-30.9)	7.2 (1.3-16.7)
Congo, Rep.	47.9	75.9 (71.8-80.4)	40.5 (33.5-47.3)	30.7 (25.3-36.1)	7.6 (1.2-17.1)	10.9 (1.8-24.8)	6.1 (1-13.5)
Congo, Dem. Rep.	814.3	98.8 (91.2-107.1)	43.6 (38.7-48.2)	43 (37.7-48.8)	10.6 (1.9-23.6)	15.4 (2.7-33.9)	8.5 (1.5-18.7)
Cote d'Ivoire	218.2	85 (79.6-90.7)	37.7 (29.9-46)	32 (25-39.3)	7.9 (1.4-17.7)	11.4 (2-25.6)	6.3 (1.1-13.9)
Equatorial Guinea	10.2	60.6 (54.3-67.8)	42 (33.3-52.4)	25.5 (20.1-31.9)	6.3 (1.1-14.1)	9.1 (1.6-20.4)	5 (0.9-11.3)
Eritrea	44.7	88.1 (76.4-99.7)	45.3 (37.7-55.2)	40 (32.5-48.3)	9.9 (1.7-21.9)	14.4 (2.5-31.6)	7.9 (1.4-17.6)
Eswatini	10.6	72.1 (66.5-78.1)	37.7 (29-47.7)	27.3 (20.6-34.9)	6.8 (1.2-15.2)	9.8 (1.7-21.9)	5.4 (1-12.1)
Ethiopia	870.8	94.2 (87.9-101.1)	37 (30.3-44.2)	35 (28.4-42)	8.7 (1.5-19.3)	12.5 (2.2-27.8)	7 (1.2-15.2)
Gabon	15.5	54.8 (50.6-59.3)	35.4 (28.8-42)	19.3 (15.6-23.5)	4.8 (0.8-10.6)	6.9 (1.2-15.2)	3.8 (0.7-8.3)
Gambia, The	20.4	93.8 (87.1-101.3)	42.1 (34.8-49.7)	39.5 (31.9-47.2)	9.8 (1.7-21.7)	14.1 (2.5-31.2)	7.8 (1.4-17.1)
Ghana	235.8	79.1 (74.2-84)	43.9 (33.7-54.1)	34.6 (26.3-43.6)	8.5 (1.5-19.4)	12.2 (2.1-27.9)	6.8 (1.2-15.5)
Guinea	114	95.6 (88.9-103.1)	39.4 (32.2-46.9)	37.6 (30.4-45.5)	9.3 (1.6-20.9)	13.5 (2.3-30.1)	7.5 (1.3-16.4)
Guinea-Bissau	16.6	70.9 (65.1-76.6)	41.3 (32.1-50.5)	29.1 (22.5-36.3)	7.2 (1.2-16.2)	10.3 (1.7-23.2)	5.7 (1-13)
Kenya	419.3	81.8 (77.5-86.4)	38.8 (35.2-42.6)	31.8 (28.5-35.3)	7.9 (1.4-17.5)	11.4 (2-25.2)	6.3 (1.1-14)
Lesotho	16.3	79.5 (73.6-85.8)	40.8 (29.6-52.8)	32.7 (23.5-42.2)	8 (1.4-18.3)	11.6 (2.1-26.4)	6.4 (1.1-14.5)
Liberia	41.4	92.8 (85.8-100.3)	41.9 (35.7-48.7)	38.8 (32.7-45.9)	9.7 (1.6-21.3)	14 (2.4-30.7)	7.7 (1.4-16.9)
Madagascar	217.6	91.8 (84-100)	46.7 (39.8-54.5)	42.8 (36.1-50.6)	10.7 (1.8-23.9)	15.4 (2.7-34.3)	8.5 (1.5-19)
Malawi	173.1	105.6 (96.1-116.1)	44.9 (39.6-50.4)	47.4 (40.8-54.9)	11.7 (2-25.9)	17 (2.9-37.3)	9.5 (1.6-20.9)
Mali	191.5	95 (88.6-102.1)	33.7 (24.1-44.4)	32.4 (22.9-42.6)	7.9 (1.3-18.2)	11.5 (2-26.3)	6.3 (1.1-14.4)
Mauritania	37.3	72 (67.4-76.6)	38.8 (32.1-45.2)	27.9 (23-33)	6.9 (1.1-15.3)	10 (1.7-22)	5.5 (1-12.3)
Mauritius	4.2	58.1 (53.4-63.6)	43.6 (35.4-53.1)	25.3 (20.4-31)	6.2 (1.1-14.1)	9 (1.6-20.3)	5 (0.9-11.1)
Mozambique	282.1	102.7 (94-112.3)	41.9 (34.2-50.3)	43.1 (34.5-52.7)	10.6 (1.8-23.7)	15.4 (2.6-34.2)	8.5 (1.5-19.1)
Namibia	19.3	63.8 (59.2-68.4)	35.3 (27.6-43.9)	22.5 (17.4-28.4)	5.6 (0.9-12.6)	8 (1.4-18)	4.5 (0.8-10)
Niger	232.8	105.5 (96.9-115)	40.6 (33.3-47.8)	42.9 (34.8-51.3)	10.5 (1.8-23.9)	15.3 (2.6-34)	8.4 (1.4-18.8)
Nigeria	1804.2	81.2 (77.1-85.4)	43 (36.3-50)	34.9 (29.2-41.2)	8.7 (1.5-19.3)	12.5 (2.2-27.6)	6.9 (1.2-15.1)
Rwanda	103.6	95.7 (88.7-103.2)	41.9 (34.6-49)	40.1 (33-47.5)	9.9 (1.7-22.3)	14.4 (2.5-32)	7.9 (1.4-17.6)
Sao Tome and Principe	1.8	81.6 (74.9-88.9)	43.5 (32.9-54.1)	35.3 (26.2-45.1)	8.7 (1.4-20.2)	12.5 (2-29.1)	6.9 (1.2-15.9)
Senegal	145.7	84.6 (79.8-90)	38.9 (32.2-45.8)	32.9 (27.1-39.2)	8.1 (1.4-18)	11.7 (2-25.8)	6.5 (1.1-14.3)

Sierra Leone	67.3	101.3 (93.4-109.6)	41.3 (35.8-46.7)	41.8 (35.8-48.3)	10.3 (1.7-22.9)	14.9 (2.5-33)	8.3 (1.4-18.2)
South Africa	336.1	54.8 (50.4-59.4)	35.4 (21.7-50)	19.4 (12-27.7)	4.7 (0.7-11.3)	6.9 (1.1-16.2)	3.8 (0.6-8.8)
South Sudan	110	86.8 (77.2-99.1)	40.7 (33.5-50.7)	35.8 (28.7-42.8)	8.8 (1.5-19.9)	12.8 (2.1-28.3)	7 (1.3-15.7)
Tanzania, United Rep.	547.9	91.4 (85.2-97.8)	40.7 (32-49.6)	37.2 (29.2-46.1)	9.2 (1.6-20.5)	13.2 (2.3-29.7)	7.3 (1.3-16.2)
Togo	68.6	95.8 (88.6-103.9)	40.9 (32.5-50)	39.4 (30.7-48.6)	9.7 (1.6-22.2)	14 (2.4-31.7)	7.7 (1.3-17.6)
Uganda	436.5	94.3 (87.9-101.7)	49.2 (43.7-55.5)	46.4 (40.1-54.1)	11.6 (2-26)	16.7 (2.9-37.5)	9.3 (1.6-20.4)
Zambia	160.1	83.6 (79-88.7)	39.1 (32.9-45.6)	32.7 (27.3-38.4)	8.1 (1.4-17.9)	11.8 (2-26)	6.5 (1.1-14.2)
Zimbabwe	146.6	79.5 (74.6-84.9)	41.4 (32.4-51.4)	33 (25.4-41.2)	8.1 (1.4-18.4)	11.8 (2.1-26.5)	6.5 (1.2-14.4)
**Region of the Americas**							
Belize	2.3	64.7 (60-70)	37.7 (24.6-49.2)	24.1 (16-32.9)	5.9 (1-14)	8.5 (1.4-20)	4.7 (0.8-10.9)
Bolivia, Plurinational State	70.7	65.6 (61.6-69.9)	69.4 (59.9-79.4)	45.5 (38.8-52.5)	11.3 (2-25.1)	16.3 (2.9-36.1)	9.1 (1.6-20)
Brazil	884.7	57 (52.7-61.8)	42.4 (35.6-50.1)	24.2 (20-28.8)	6 (1.1-13.5)	8.7 (1.5-19.5)	4.8 (0.9-10.6)
Colombia	225.6	54.3 (50.9-57.8)	38 (33.4-42.6)	20.6 (17.9-23.5)	5.1 (0.9-11.3)	7.4 (1.3-16.4)	4.1 (0.7-9)
Costa Rica	21.4	55.9 (51.4-60.7)	41.9 (34.8-50)	23.5 (19.3-28.1)	5.8 (1-13.2)	8.4 (1.4-19)	4.6 (0.8-10.4)
Cuba	36.9	56.7 (52.5-61)	31.9 (25.6-38.4)	18 (14.4-22)	4.5 (0.8-10.1)	6.5 (1.1-14.4)	3.6 (0.6-8)
Dominica	0.4	57.9 (52.8-63.7)	43.3 (35.5-51.8)	25 (20.3-30.5)	6.2 (1.1-14.2)	8.9 (1.5-20.2)	4.9 (0.9-11.1)
Dominican Rep.	63.8	60 (56.3-64)	32.2 (28.7-36)	19.4 (17-21.9)	4.8 (0.8-10.6)	7 (1.2-15.2)	3.8 (0.7-8.4)
Ecuador	96.5	56.8 (52.6-61.4)	42.6 (36.6-49.6)	24.2 (20.6-28.5)	6.1 (1-13.5)	8.7 (1.5-19.3)	4.8 (0.9-10.6)
El Salvador	34.9	60.5 (56.6-64.7)	35.1 (29.8-40.6)	21.3 (17.9-24.9)	5.3 (0.9-11.7)	7.7 (1.3-16.8)	4.2 (0.8-9.2)
Grenada	0.6	58.3 (53.2-63.8)	45.1 (35.5-55.3)	26.3 (20.6-32.5)	6.5 (1.1-14.6)	9.4 (1.6-21.1)	5.2 (0.9-11.5)
Guatemala	117.4	70.5 (66.2-74.6)	38.1 (33.9-42.4)	26.8 (23.6-30.4)	6.7 (1.1-14.6)	9.7 (1.6-21.1)	5.3 (0.9-11.5)
Guyana	4.4	63.3 (57.9-69.1)	39.5 (28.2-51.3)	24.9 (17.9-32.6)	6.1 (1.1-14.1)	8.9 (1.5-20.3)	4.9 (0.8-11.1)
Haiti	74.2	90.4 (84-97.6)	41.5 (35.5-47.9)	37.6 (31.5-44.1)	9.3 (1.6-20.6)	13.5 (2.3-29.9)	7.4 (1.3-16.4)
Honduras	57.6	72.1 (68.1-76.3)	41 (36.8-45.4)	29.5 (26.1-33.2)	7.4 (1.2-16.1)	10.7 (1.8-23.4)	5.9 (1-12.9)
Jamaica	12.6	58.1 (53.3-63.6)	41 (34.5-48.1)	23.8 (19.9-28.2)	5.9 (1-13.3)	8.5 (1.5-19.1)	4.7 (0.8-10.4)
Mexico	685.7	56.4 (52.2-60.8)	45.4 (35.3-56.3)	25.6 (19.6-32.2)	6.3 (1-14.2)	9.2 (1.5-20.5)	5.1 (0.8-11.3)
Nicaragua	36.8	65.2 (59.1-71.9)	45.9 (37.7-55.9)	29.9 (24.5-36.8)	7.5 (1.3-16.4)	10.8 (1.9-23.7)	5.9 (1.1-12.8)
Paraguay	40.6	55.6 (51.7-59.9)	57.3 (49.5-65.4)	31.8 (27.1-37.1)	7.9 (1.4-17.2)	11.4 (2-24.8)	6.3 (1.1-13.8)
Peru	179.5	57.6 (53.9-61.6)	59.3 (51-67.5)	34.1 (28.9-39.6)	8.4 (1.4-18.7)	12.2 (2.1-26.9)	6.8 (1.2-14.9)
St. Lucia	0.6	58.1 (53.1-63.5)	42.7 (34.5-51.2)	24.8 (19.9-29.8)	6.1 (1.1-13.9)	8.8 (1.5-20)	4.9 (0.9-11)
St. Vincent and the Grenadines	0.5	59 (53.8-64.7)	42 (34.7-50.2)	24.8 (20.4-30)	6.1 (1.1-13.9)	8.9 (1.6-20)	4.9 (0.9-10.9)
Suriname	3	58.8 (53.7-64.8)	42.7 (35.4-51.5)	25.2 (20.7-30.6)	6.2 (1.1-13.8)	9 (1.6-19.8)	5 (0.9-11)
Venezuela, Bolivarian Rep.	178.4	57.1 (52.5-62.2)	44.7 (36.7-53.8)	25.6 (20.7-31)	6.3 (1.1-14.1)	9.1 (1.6-20.2)	5 (0.9-11)
**Eastern Mediterranean Region**							
Afghanistan	316.6	95.7 (88.6-103.4)	61.9 (58.5-65.3)	59.2 (54-64.9)	14.8 (2.4-32.2)	21.4 (3.6-46.4)	11.8 (2-25.8)
Djibouti	5.9	68.5 (61.9-76.4)	45.2 (36.8-54.5)	30.9 (25.3-37.2)	7.7 (1.4-17.1)	11.1 (2-24.5)	6.1 (1.1-13.4)
Egypt, Arab Rep.	694.3	69.6 (66.1-73.1)	58.3 (54.8-62)	40.6 (37.5-44)	10.1 (1.7-22.1)	14.7 (2.5-31.8)	8.1 (1.4-17.7)
Iran, Islamic Rep.	417.8	56.9 (52.5-61.8)	47 (39.6-55.3)	26.8 (22.5-32)	6.6 (1.1-15.1)	9.6 (1.6-21.8)	5.3 (0.9-11.8)
Iraq	327.5	62.6 (56.8-69.4)	47.2 (38.9-57.1)	29.6 (24.1-36.1)	7.3 (1.2-16.4)	10.6 (1.8-23.6)	5.9 (1-13)
Jordan	71.1	53.7 (50.3-57.2)	55.9 (50.1-61.7)	30 (26.4-33.9)	7.4 (1.3-16.2)	10.8 (1.9-23.3)	5.9 (1-13)
Lebanon	26.4	55.5 (50.5-61.1)	48.1 (40.2-57.2)	26.7 (22.2-31.8)	6.6 (1.1-14.9)	9.5 (1.6-21.5)	5.3 (0.9-12)
Libyan Arab Rep.	37.5	60.1 (53.3-69.6)	47.3 (38-58.6)	28.6 (22.9-35.8)	7.1 (1.2-16.2)	10.3 (1.7-23.3)	5.7 (1-12.6)
Morocco	201.9	63.9 (58.2-70.7)	49.1 (40.3-59.3)	31.4 (26-37.7)	7.7 (1.3-17.5)	11.2 (1.9-25)	6.2 (1.1-13.7)
Pakistan	1458.8	78 (73.8-82.4)	53.1 (49-57.1)	41.4 (37.5-45.4)	10.4 (1.8-22.5)	15 (2.6-32.3)	8.3 (1.4-18)
Palestinian Terr.	41	65.6 (61.4-69.8)	53.5 (48.8-58.5)	35.1 (31.3-39.1)	8.7 (1.5-19)	12.6 (2.1-27.4)	7 (1.2-15.3)
Somalia	147.9	76.2 (66.9-91.1)	43.5 (35.4-53.4)	33.5 (27.7-40.2)	8.3 (1.4-18.6)	12 (2.1-26.8)	6.6 (1.2-14.6)
Sudan	344.8	79.8 (71.6-88.1)	43.2 (36.4-50.8)	34.4 (29-40.6)	8.5 (1.5-18.7)	12.4 (2.1-27)	6.8 (1.2-14.8)
Tunisia	58.8	57.2 (52.7-62.3)	48.4 (40.2-57.8)	27.7 (23-33.3)	6.8 (1.2-15.6)	9.8 (1.7-22.3)	5.5 (0.9-12.1)

Yemen, Rep.	236.3	78.5 (73.8-83)	62.3 (58.4-66.1)	48.9 (44.8-53.1)	12.2 (2-26.7)	17.6 (2.9-38.4)	9.7 (1.7-21.4)
**European Region**							
Albania	9.7	60 (55.3-65.3)	48.6 (29.7-67.6)	29 (17.2-41.4)	7 (1.2-17.2)	10.1 (1.7-24.6)	5.6 (1-13.4)
Armenia	12.6	53.8 (48.6-59.5)	57.9 (36.8-78.9)	31.4 (18.9-44.1)	7.6 (1.2-18.2)	10.9 (1.8-26.1)	6 (1-14.3)
Azerbaijan	49.5	57.6 (52.5-63.2)	40 (24-58.5)	23.2 (13.6-34.3)	5.6 (0.9-14.2)	8.2 (1.3-20.4)	4.5 (0.7-11)
Belarus	34.2	56.2 (51.8-60.9)	41.7 (33.8-50.1)	23.5 (18.8-28.4)	5.8 (1-12.9)	8.4 (1.4-18.7)	4.6 (0.8-10.3)
Bosnia and Herzegovina	10.1	55.9 (51.3-61.3)	43.4 (35.4-53)	24.3 (19.6-30)	6 (1-13.6)	8.7 (1.5-19.5)	4.8 (0.8-10.6)
Bulgaria	21.1	55.4 (51-60.3)	42.7 (34.2-51.5)	23.6 (18.9-29)	5.8 (1-13.3)	8.4 (1.4-19.1)	4.7 (0.8-10.4)
Georgia	16.6	57.5 (52.6-62.6)	43.3 (35-53.4)	24.9 (20-31.1)	6.2 (1-14)	8.9 (1.5-20.1)	4.9 (0.9-11)
Kazakhstan	118.2	56.6 (52.2-61.2)	44.9 (36.3-53.7)	25.4 (20.4-30.7)	6.3 (1.1-14.3)	9 (1.5-20.3)	5 (0.9-11.2)
Kosovo	9	59.2 (54.4-64.4)	49.5 (34.7-66.3)	29.3 (20.3-39.6)	7.2 (1.2-17)	10.4 (1.8-24.7)	5.8 (1-13.4)
Kyrgyzstan	43.4	69 (63.8-74.3)	43.5 (21.7-68)	30.4 (14.8-46.8)	7.3 (1-18.3)	10.5 (1.5-26.6)	5.8 (0.8-14.4)
Moldova, Rep.	13.6	60.9 (55.5-66.9)	46.6 (37.5-57.4)	28.4 (22.7-35.1)	7 (1.2-15.5)	10.1 (1.7-22.4)	5.6 (0.9-12.3)
Montenegro	2.3	55.3 (50.2-61.2)	40 (20-70)	23.1 (10.4-38.7)	5.4 (0.8-15.1)	7.8 (1.1-21.8)	4.3 (0.6-11.6)
North Macedonia	6.8	55.7 (51.1-60.4)	44.9 (35.9-55.5)	24.9 (19.8-31.3)	6.1 (1-14)	8.9 (1.5-20)	4.9 (0.9-10.9)
Romania	60.3	56.9 (52.1-61.7)	42.7 (34.6-51.3)	24.3 (19.3-29.3)	6 (1-13.4)	8.7 (1.5-19.3)	4.8 (0.8-10.5)
Russian Fed.	541.7	55.5 (50.8-60.3)	43.3 (35.5-51.8)	24 (19.6-28.7)	5.9 (1-13.3)	8.6 (1.4-19.1)	4.7 (0.9-10.5)
Serbia	27.9	55.9 (51.4-60.7)	42.2 (33.8-51.9)	23.6 (18.8-29.3)	5.9 (1-13.3)	8.5 (1.4-19.2)	4.7 (0.8-10.4)
Tajikistan	68.4	73.9 (68.7-79.7)	55.6 (33.3-77.8)	42.9 (25-60)	10.4 (1.6-25.3)	15 (2.3-36.2)	8.3 (1.3-19.5)
Turkey	401	56.9 (52.1-62.3)	46.1 (37.9-55.1)	26.2 (21.1-32.1)	6.5 (1.1-14.8)	9.4 (1.6-21.4)	5.2 (0.9-11.7)
Turkmenistan	37.8	52.5 (48.2-56.5)	30 (10-70)	17.3 (4.9-36.5)	4.2 (0-13.4)	6.1 (0-19.4)	3.3 (0-10.4)
Ukraine	150.6	59.3 (54.2-64.6)	43.9 (35.6-53.2)	26 (21.1-31.8)	6.4 (1.1-14.6)	9.3 (1.5-20.8)	5.2 (0.9-11.4)
Uzbekistan	192.8	65.2 (59.2-71.9)	47.5 (37.5-58.8)	31 (24.3-38.1)	7.6 (1.3-17.1)	11 (1.9-24.4)	6.1 (1.1-13.4)
**Southeast Asia Region**							
Bangladesh	913.3	80.8 (76.4-85.5)	53.5 (46.5-60.5)	43.2 (37-49.7)	10.7 (1.9-23.8)	15.5 (2.7-34.3)	8.5 (1.5-19)
Bhutan	4.3	70.8 (63.4-78.6)	56.9 (46.3-69.2)	40.2 (33.2-48.8)	10 (1.7-22.2)	14.4 (2.5-32.1)	8 (1.4-17.5)
India	7399.5	75.5 (72.3-78.7)	51.5 (48.9-54.4)	38.9 (36.3-41.7)	9.7 (1.6-21.1)	14 (2.4-30.6)	7.8 (1.3-17)
Indonesia	1449.1	63.8 (60.7-67.1)	64 (56-72.2)	40.7 (35.5-46.5)	10.1 (1.7-22.2)	14.6 (2.4-31.9)	8.1 (1.4-17.6)
Maldives	2.2	49.3 (44.7-54.6)	57.9 (35-80)	28.8 (17.2-40.5)	7 (1.1-16.6)	10.1 (1.7-24)	5.5 (0.9-13.3)
Myanmar	273.6	84.8 (79.5-90.7)	66.9 (55.6-78.4)	56.7 (46.8-67.2)	14.1 (2.3-31.5)	20.3 (3.4-45.4)	11.2 (2-24.8)
Nepal	172.3	84 (78.1-90.2)	53.3 (37.8-68.9)	44.9 (32.1-58.5)	10.9 (1.9-25)	15.8 (2.7-36.2)	8.8 (1.5-19.7)
Sri Lanka	101.2	62.6 (56.8-68.9)	55.5 (45.1-67.9)	34.7 (28.2-42.6)	8.6 (1.5-19.3)	12.4 (2.1-27.9)	6.9 (1.2-15.1)
Thailand	234.7	63.1 (59.2-67.2)	58 (46.8-69.6)	36.5 (29.2-44.5)	9 (1.6-20.1)	13 (2.3-29.1)	7.2 (1.3-16)
Timor-Leste	12.1	77.5 (73.4-82.1)	65.5 (50.9-79.7)	50.9 (39.2-62.3)	12.6 (2.1-28.4)	18.2 (3-40.7)	10 (1.7-22.3)
**Western Pacific Region**							
American Samoa	0.3	59.8 (53.5-66.6)	47.3 (37.5-60.4)	28.2 (22.2-37.1)	7 (1.2-16.2)	10.1 (1.7-23)	5.6 (1-12.5)
Cambodia	106.2	84.6 (79.7-89.9)	68 (57.4-79.1)	57.5 (47.9-67.5)	14.2 (2.4-31.7)	20.6 (3.5-45.8)	11.4 (2-25.2)
China, People’s Rep.	5134.2	57.7 (52.8-62.9)	47.9 (38.9-58.3)	27.6 (22.4-33.8)	6.8 (1.2-15.3)	9.9 (1.7-22)	5.5 (1-12.1)
Fiji	5.4	59.9 (54.6-66.1)	46.3 (37.9-56.1)	27.7 (22.4-34.2)	6.8 (1.2-15.6)	9.9 (1.7-22.5)	5.4 (1-12.3)
Kiribati	0.9	68 (61.4-75.3)	47.7 (38.5-59.5)	32.5 (26.1-39.8)	8 (1.5-18.4)	11.6 (2.1-26.4)	6.4 (1.2-14.5)
Lao People’s Dem. Rep.	46.6	79.5 (75.2-84.2)	69.2 (55.4-82.8)	55.1 (43.5-66.6)	13.6 (2.3-30.3)	19.7 (3.4-43.8)	10.9 (1.9-24.2)
Malaysia	151.6	56.7 (51.9-61.9)	48.4 (40.2-58.5)	27.4 (22.4-33.7)	6.8 (1.2-15.4)	9.9 (1.7-22.3)	5.4 (1-12.2)
Marshall Islands	0.3	60.9 (55.3-67)	41.9 (34.3-51.8)	25.5 (20.8-31.4)	6.3 (1.1-14.4)	9.1 (1.6-20.6)	5 (0.9-11.4)
Micronesia, Fed.	3.1	69.5 (62.6-77.2)	49.1 (39.1-61.2)	34.1 (27.1-42.6)	8.5 (1.4-19.2)	12.2 (2-27.4)	6.8 (1.2-14.9)
Mongolia	20.6	60.2 (56.2-64.5)	51.1 (42.2-60.4)	30.8 (25.1-36.9)	7.6 (1.3-17.1)	11 (1.9-24.5)	6.1 (1.1-13.4)
Nauru	0.1	61.5 (55.6-68.5)	48 (38.6-61.6)	29.4 (23.4-38.4)	7.4 (1.3-17)	10.7 (1.8-24.4)	5.9 (1-13.3)
Papua New Guinea	60.9	77.6 (70.2-85.6)	48.8 (40-59.1)	37.9 (30.9-46)	9.4 (1.6-21.2)	13.7 (2.4-30.4)	7.5 (1.3-16.5)

Philippines	679.1	65.6 (62.3-69.2)	63.5 (52.1-75.5)	41.7 (33.8-49.8)	10.4 (1.8-22.9)	15 (2.6-32.7)	8.3 (1.4-18.1)
Samoa	1.5	62.4 (56.6-68.9)	48 (39.4-58.8)	30 (24.4-36.8)	7.4 (1.3-16.7)	10.7 (1.8-24)	5.9 (1.1-13.1)
Solomon Islands	5	78.3 (70.5-86.1)	47.9 (39.3-58.8)	37.5 (30.6-45.8)	9.3 (1.6-20.6)	13.5 (2.3-29.7)	7.5 (1.3-16.3)
Tonga	0.8	65 (58.9-71.6)	52.8 (41.3-67.2)	34.3 (27.2-43.4)	8.5 (1.5-19.5)	12.3 (2.1-28.1)	6.8 (1.2-15.2)
Tuvalu	0.1	58.4 (52.9-64.5)	44.6 (36.9-53.4)	26.1 (21.5-31.3)	6.4 (1.1-14.6)	9.3 (1.6-21)	5.2 (0.9-11.5)
Vanuatu	2.1	75.6 (67-84.7)	46.2 (36.9-58)	34.9 (28.1-43.2)	8.6 (1.5-19.6)	12.4 (2.1-27.9)	6.9 (1.2-15.5)
Viet Nam	463.9	67.2 (62.6-72)	59.6 (47.3-72)	40 (31.7-48.8)	9.8 (1.7-22.2)	14.2 (2.4-32)	7.9 (1.4-17.5)

Incidence rate estimates are presented as cases per 100 children annually in the eligible age range. Estimates are obtained from analyses of DHS/MICS surveys covering 944,173 children across 77 countries, as well as extrapolations based on 405 health, nutrition, and population indicators for all LMICs. Quantiles are obtained via 5,000 independent draws from the distribution of estimates.

Table S16: Upper-bound country-specific estimates of the incidence of ARI and antibiotic-treated ARI among children ages 0-59 months preventable by 10- and 13-valent pneumococcal conjugate vaccines.

Country	Population ages 0-59 months	ARI attributable to all causes			ARI attributable to PCV10/13-type *Streptococcus pneumoniae*		
	Children (in 10,000s)	All cases, per 100 (95% CI)	Proportion treated with antibiotics, % (95% CI)	Antibiotic-treated cases, per 100 (95% CI)	Antibiotic-treated cases, per 100 (95% CI)		Antibiotic-treated cases preventable by vaccine direct effects, per 100 (95% CI)
					IPD probe estimate	*AOM probe estimate*	
**African Region**							
Algeria	466.4	211.3 (197-227.6)	46.5 (39.7-56.2)	98.2 (83.6-119.7)	10.8 (-1.5-25.7)	15.7 (-2.2-36.9)	8.6 (-1.3-19.8)
Angola	515.8	252 (240.3-263.7)	36.6 (31-42)	92.2 (77.6-106.9)	10 (-1.5-23.8)	14.5 (-2.2-34.3)	8.1 (-1.2-18.4)
Benin	173.9	348 (330.6-366.6)	41.4 (34.3-48.5)	144 (118.7-171.1)	15.7 (-2.3-37.3)	22.7 (-3.3-53.5)	12.5 (-1.8-28.9)
Botswana	25.6	227.8 (210.1-247.5)	41.9 (35.8-49.1)	95.4 (82.2-111.9)	10.5 (-1.5-24.6)	15.2 (-2.1-35.4)	8.4 (-1.2-19.1)
Burkina Faso	316.1	376.2 (356.5-397.6)	41.4 (35.6-47.3)	156 (132.5-180.1)	17.1 (-2.5-40.2)	24.7 (-3.6-57.9)	13.7 (-2-31.1)
Burundi	185.3	407 (380.6-436.6)	44.3 (40.4-48.3)	180.1 (161.2-201)	19.8 (-2.8-46.5)	28.8 (-4.1-66.6)	15.9 (-2.3-36)
Cabo Verde	5.5	232.3 (213.9-253.5)	46.5 (39.3-55)	107.9 (91.8-127.4)	11.8 (-1.7-28.1)	17 (-2.4-40.2)	9.4 (-1.3-21.8)
Cameroon	374.2	317.3 (304.3-331.5)	42.5 (38-46.7)	134.6 (119.5-150.2)	14.7 (-2.1-34.6)	21.2 (-3-50)	11.8 (-1.8-26.8)
Cent. African Rep.	72.8	350.7 (318.2-383)	40.2 (35.4-45.4)	140.9 (121.9-161.3)	15.3 (-2.3-36.1)	22.2 (-3.3-51.5)	12.4 (-1.9-28.1)
Chad	260.1	358.6 (341.3-378)	41.1 (38-44.2)	147.3 (134.2-161.4)	16.1 (-2.4-37.8)	23.3 (-3.4-53.9)	12.9 (-1.9-29.4)
Comoros	11.7	348.7 (331-368.4)	41 (31.7-51)	143 (110.2-178.7)	15.5 (-2.2-38.1)	22.5 (-3.3-55)	12.4 (-1.8-29.3)
Congo, Rep.	81.4	285.7 (274-297.5)	41.3 (36.8-46.1)	118.2 (104.2-132.6)	12.9 (-1.9-30.3)	18.7 (-2.7-43.6)	10.3 (-1.5-23.6)
Congo, Dem. Rep.	1409.9	371.2 (349.9-393.5)	44.6 (41.3-47.8)	165.2 (150.5-181.6)	18.1 (-2.6-42.1)	26.2 (-3.8-60.7)	14.5 (-2.1-33.1)
Cote d'Ivoire	376.5	325 (310.2-340.4)	39 (33.3-45.2)	126.7 (107.5-148)	13.8 (-1.9-32.7)	19.9 (-2.7-46.9)	11 (-1.5-25.4)
Equatorial Guinea	17.6	228.6 (207.7-253.4)	43.5 (35.6-52.7)	99.3 (81.7-120.4)	10.8 (-1.6-26)	15.7 (-2.3-37)	8.7 (-1.3-20.1)
Eritrea	74.4	332.1 (289.5-377.9)	46.1 (39-55.1)	153.1 (127.5-181.9)	16.8 (-2.5-40.2)	24.2 (-3.6-57.4)	13.4 (-2-31)
Eswatini	17.8	273.4 (260.8-287.3)	38.7 (32.3-45.5)	105.9 (87.8-125.6)	11.5 (-1.6-27.7)	16.7 (-2.3-39.2)	9.2 (-1.3-21.3)
Ethiopia	1490.1	359.3 (341.2-377.2)	38.9 (34-43.6)	139.5 (120.3-158.8)	15.2 (-2.2-36)	22 (-3.1-51.4)	12.2 (-1.8-27.7)
Gabon	26.7	206 (194.3-218)	36.3 (31.6-41.4)	74.8 (64.3-86.1)	8.2 (-1.2-19.3)	11.9 (-1.7-27.5)	6.5 (-1-15)
Gambia, The	35.2	358 (340-377.6)	42.6 (37.4-47.7)	152.5 (132.3-173.5)	16.5 (-2.4-39.3)	23.9 (-3.5-56.4)	13.3 (-1.8-30.6)
Ghana	402.3	297 (284.6-309.2)	43.2 (36.3-50.5)	128.4 (107-150.9)	13.9 (-2-33)	20.1 (-2.9-47.6)	11.1 (-1.6-25.7)
Guinea	194.9	364.4 (346.5-384.8)	40.6 (35.5-45.4)	147.7 (127.7-167.9)	16.2 (-2.4-38.5)	23.4 (-3.5-55.1)	12.9 (-2-29.8)
Guinea-Bissau	28.6	257.1 (242.5-272.3)	41.7 (34.1-49.4)	107.2 (87.1-128.3)	11.7 (-1.7-27.8)	16.9 (-2.4-40)	9.3 (-1.3-21.7)
Kenya	699.7	305.7 (293.2-318.9)	39.8 (37-42.6)	121.5 (111.9-132)	13.3 (-1.9-31.4)	19.2 (-2.8-45)	10.6 (-1.6-24.3)
Lesotho	28	303.3 (289.7-317.9)	41.6 (33.6-50)	126.7 (101.4-153.2)	13.8 (-2-32.9)	19.9 (-2.9-46.9)	11 (-1.6-25.2)
Liberia	70.5	352.1 (333.6-371.8)	43.1 (38.8-47.6)	152.1 (135.2-170.9)	16.7 (-2.5-38.9)	24.2 (-3.6-56)	13.3 (-1.9-30.5)
Madagascar	370	342 (316.5-367.8)	47.3 (41-54.4)	161.7 (139.8-185)	17.6 (-2.6-41.6)	25.4 (-3.7-59.8)	14 (-2.1-32.1)

Malawi	288.8	395.2 (370.8-423.6)	45.6 (41.6-49.5)	180.3 (161.5-201)	19.7 (-2.8-47)	28.6 (-4.2-67.1)	15.8 (-2.3-36.3)
Mali	327.4	361 (343-380.1)	36.1 (29.3-43.9)	130.8 (104.9-159.5)	14.2 (-2-33.8)	20.5 (-2.9-48.8)	11.4 (-1.6-26.4)
Mauritania	64	265.4 (253.4-277.1)	39.9 (34.7-45.1)	105.8 (91.1-121)	11.5 (-1.7-27.2)	16.7 (-2.4-39)	9.3 (-1.3-21.3)
Mauritius	6.9	210.7 (195.7-228.8)	44.6 (37.4-53.5)	94.3 (79-112.6)	10.3 (-1.5-24.7)	14.9 (-2.1-35.5)	8.3 (-1.2-19.1)
Mozambique	484.4	388.7 (365.3-413.7)	43.1 (37.3-49)	167.3 (143.3-193.4)	18.3 (-2.7-43.5)	26.4 (-3.9-62.3)	14.6 (-2.2-33.7)
Namibia	33.5	241.5 (229.7-254.3)	36.5 (30.6-42.7)	88.1 (73.5-103.9)	9.6 (-1.4-22.8)	13.9 (-2-32.7)	7.7 (-1.2-17.8)
Niger	406.6	402.4 (379.2-428.5)	41.6 (37-46.1)	167.1 (147-189.5)	18.3 (-2.6-43.2)	26.5 (-3.8-61.6)	14.7 (-2.1-33.3)
Nigeria	3110.9	307.5 (295.4-321.1)	42.7 (38-47.4)	131.2 (116.3-146.9)	14.3 (-2-33.6)	20.7 (-2.9-48)	11.5 (-1.7-26.1)
Rwanda	173.5	361.1 (343.1-380.5)	42.8 (37.9-47.6)	154.2 (136.1-174)	16.9 (-2.4-39.5)	24.4 (-3.5-56.7)	13.5 (-2-31)
Sao Tome and Principe	3.1	307.8 (293.3-323.7)	43.7 (35-52.6)	134.5 (108.1-162.6)	14.7 (-2.2-35.4)	21.1 (-3.1-50.6)	11.7 (-1.7-27.5)
Senegal	249.3	321 (306.4-335.4)	40.1 (35.4-45)	128.6 (112.5-145.9)	14 (-2-33.3)	20.3 (-3-47.4)	11.3 (-1.6-25.8)
Sierra Leone	113.5	384.1 (362.6-408.6)	42.8 (39.3-46.5)	164.6 (148.2-183.2)	18.1 (-2.5-42.3)	26.2 (-3.7-61.1)	14.4 (-2.1-32.9)
South Africa	566.4	203.4 (192.1-214.7)	36.8 (26.3-48.1)	75.5 (53.3-98.4)	8.2 (-1.1-20.1)	11.8 (-1.6-29)	6.5 (-0.9-15.8)
South Sudan	188.2	329.6 (290.6-374.2)	41.7 (35-50.5)	138.7 (115.5-161)	15.2 (-2.3-35.8)	22 (-3.3-51)	12.2 (-1.8-27.6)
Tanzania, United Rep.	941.9	346.6 (330.4-363.8)	41.6 (35.9-47.3)	144.1 (123.6-166.4)	15.6 (-2.4-36.9)	22.6 (-3.4-53.2)	12.5 (-1.8-29.1)
Togo	116.2	361.5 (342.1-382.9)	42.3 (35.9-48.4)	152.8 (128.6-177.5)	16.8 (-2.3-39)	24.1 (-3.4-55.7)	13.4 (-1.8-30.2)
Uganda	751.2	353.9 (335.7-373.1)	48.4 (44.3-52.6)	171.3 (154.8-188.6)	18.7 (-2.7-44)	27.1 (-4-63)	15 (-2.2-34.4)
Zambia	275	313.4 (300.8-327.7)	40.1 (35.5-44.9)	125.7 (110.3-141.4)	13.8 (-2-32.3)	20 (-2.8-46.7)	11 (-1.6-25.3)
Zimbabwe	250.6	297.5 (284.5-311.1)	40.5 (33.7-47.7)	120.9 (100-143.6)	13.2 (-1.9-30.9)	19.1 (-2.7-44.3)	10.6 (-1.5-24.1)
**Region of the Americas**							
Belize	3.9	235.3 (223.1-247.8)	38.5 (28.1-48.4)	90.3 (66-114.9)	9.8 (-1.3-24)	14.2 (-1.9-34.5)	7.8 (-1.1-18.7)
Bolivia, Plurinational State	118.6	245.2 (234.7-256.5)	68.3 (61.7-75.1)	167.5 (149.8-186.7)	18.3 (-2.8-43.1)	26.6 (-4-62.1)	14.7 (-2.3-33.4)
Brazil	1487.5	208.4 (195.4-222.5)	43.4 (37.6-50.5)	90.7 (78-105.3)	9.9 (-1.4-23.5)	14.3 (-2.1-33.4)	7.9 (-1.2-18.2)
Colombia	373.8	200.1 (190.3-210.5)	38.7 (35.5-42.2)	77.3 (69.9-85.4)	8.4 (-1.2-20.1)	12.2 (-1.8-28.8)	6.8 (-1-15.3)
Costa Rica	35	201.9 (188.9-215.5)	43.2 (36.9-50.5)	87.2 (74.3-101.5)	9.5 (-1.3-22.9)	13.8 (-1.9-33)	7.6 (-1.1-17.6)
Cuba	62.9	196.4 (185.6-207.5)	32.6 (27.1-38.5)	64.2 (52.7-76.2)	7 (-1-16.8)	10.1 (-1.5-24.2)	5.6 (-0.8-13)
Dominica	0.6	212.9 (196.8-231.1)	43.9 (37.1-51.6)	93.4 (79-110.9)	10.2 (-1.5-24.3)	14.8 (-2.2-35)	8.2 (-1.2-18.9)
Dominican Rep.	106.2	223.5 (212.1-234.9)	34.4 (31.6-37.3)	77 (69.5-84.5)	8.4 (-1.2-19.8)	12.2 (-1.7-28.3)	6.7 (-1-15.4)
Ecuador	161	207.2 (194.3-221.2)	44 (38.5-50)	91.3 (78.9-104.7)	9.9 (-1.4-23.6)	14.4 (-2-33.9)	8 (-1.1-18.1)
El Salvador	58	214 (203.8-224.4)	35.8 (31.2-40.3)	76.6 (66.4-87.2)	8.4 (-1.2-19.5)	12.1 (-1.7-28.1)	6.7 (-1-15.1)
Grenada	1	213.8 (197.1-231.6)	45.8 (37.2-54.9)	97.7 (79.4-118.6)	10.6 (-1.5-25.2)	15.4 (-2.2-36.1)	8.5 (-1.2-19.6)
Guatemala	199.4	263.7 (251.8-276.3)	39.4 (36.3-42.5)	103.9 (94.7-113.2)	11.4 (-1.6-26.6)	16.5 (-2.4-38.2)	9.1 (-1.4-20.6)
Guyana	7.5	239.2 (226.2-252.1)	39.8 (30.8-48.7)	95.1 (73.2-118)	10.3 (-1.6-25.2)	14.9 (-2.2-36)	8.2 (-1.2-19.4)
Haiti	123.8	338.3 (321.9-355.9)	41.9 (37.4-46.9)	141.9 (125.2-160)	15.5 (-2.2-36.3)	22.5 (-3.2-52)	12.4 (-1.8-28.1)
Honduras	95.5	271.5 (260.2-283.3)	42.1 (39.1-45.3)	114.4 (105-124.4)	12.5 (-1.9-29.3)	18 (-2.6-41.9)	9.9 (-1.5-22.7)
Jamaica	20.7	211.5 (196.3-228.3)	42.2 (36.3-48.6)	89.2 (76.7-103.1)	9.8 (-1.3-22.9)	14.2 (-1.9-33)	7.8 (-1.1-17.7)
Mexico	1153.3	205.6 (194.1-217.6)	47.5 (39.5-56.6)	98.1 (80.4-117.6)	10.8 (-1.5-25.5)	15.6 (-2.3-36.5)	8.6 (-1.2-19.8)
Nicaragua	60.6	238.5 (219.4-260.4)	47.6 (40.1-56.9)	113.9 (96-135)	12.4 (-1.8-29.6)	18 (-2.6-42.5)	10 (-1.4-22.9)

Paraguay	67.4	199.4 (189.5-210.3)	57.1 (51.2-63)	113.8 (101.1-127.5)	12.4 (-1.7-29.7)	18 (-2.5-42.3)	9.9 (-1.4-22.9)
Peru	302	211.7 (201.1-222.7)	58.9 (52.8-65.2)	124.7 (110.1-139.7)	13.6 (-2-32)	19.7 (-2.9-45.7)	10.9 (-1.6-24.9)
St. Lucia	1.1	213.8 (197.1-232.3)	43.7 (36.4-51.5)	93.3 (78.4-110)	10.2 (-1.5-24.2)	14.8 (-2.1-34.7)	8.2 (-1.2-18.8)
St. Vincent and the Grenadines	0.9	214.4 (198.1-232.3)	43.4 (36.6-51.2)	93 (78.2-110.4)	10.2 (-1.4-24.2)	14.7 (-2.1-34.9)	8.1 (-1.2-18.8)
Suriname	5	217.9 (200.5-239.1)	43.4 (36.7-51.3)	94.7 (80-111.6)	10.3 (-1.5-24.7)	14.9 (-2.2-35.2)	8.3 (-1.2-18.8)
Venezuela, Bolivarian Rep.	297.5	209.8 (195.4-227.8)	45.8 (38.6-53.9)	96.5 (79.7-113.8)	10.5 (-1.5-24.9)	15.2 (-2.1-35.8)	8.4 (-1.2-19.4)
**Eastern Mediterranean Region**							
Afghanistan	523.9	356.8 (338.2-377.1)	61.5 (58.9-64)	219.5 (204.8-234.5)	24.1 (-3.5-56)	34.9 (-5.1-80.5)	19.2 (-2.8-43.4)
Djibouti	10.1	259.5 (236.3-285.4)	45.9 (38.4-54.4)	119.2 (100.3-139.6)	13 (-1.8-31)	18.8 (-2.6-44.6)	10.4 (-1.4-23.8)
Egypt, Arab Rep.	1237.4	258.9 (248.9-269.4)	57.9 (55.3-60.7)	150 (141-159.7)	16.4 (-2.3-38.3)	23.7 (-3.4-55)	13.1 (-1.9-29.9)
Iran, Islamic Rep.	689.9	208.3 (194.9-222.7)	47.4 (41.1-54.7)	98.8 (85.5-113.3)	10.8 (-1.6-25.3)	15.6 (-2.3-36.2)	8.6 (-1.3-19.6)
Iraq	560.3	235.3 (214.3-259.9)	47.7 (39.9-56.7)	112.2 (94.3-133.7)	12.2 (-1.8-28.9)	17.6 (-2.6-41.7)	9.8 (-1.4-22.7)
Jordan	120.2	196.2 (186.8-205.9)	55.8 (51.6-60.3)	109.7 (99.7-120.2)	12 (-1.7-28.3)	17.4 (-2.5-40.6)	9.5 (-1.4-21.6)
Lebanon	46.1	201.7 (186.3-219.3)	48.9 (41.8-57.1)	98.7 (84.8-114.5)	10.8 (-1.6-25.5)	15.6 (-2.3-36.6)	8.7 (-1.3-19.6)
Libyan Arab Rep.	63	221.2 (199-255.6)	49 (40.4-59.2)	108.9 (90.5-131.2)	11.9 (-1.7-28.6)	17.2 (-2.5-41.2)	9.5 (-1.4-22.1)
Morocco	345.7	236.3 (216.9-257.8)	49.3 (41.5-58.6)	116.4 (99.6-136.8)	12.7 (-1.8-30.2)	18.3 (-2.6-43.1)	10.2 (-1.5-23.3)
Pakistan	2471.2	292.9 (280-306)	53.6 (50.4-56.7)	156.9 (146.1-168.3)	17.1 (-2.5-40)	24.8 (-3.6-57.6)	13.7 (-2-31.1)
Palestinian Terr.	69.9	226.2 (216.4-237.2)	53.1 (49.1-57.2)	120.1 (109.6-131.1)	13.1 (-1.9-30.7)	19 (-2.7-44)	10.5 (-1.6-23.6)
Somalia	255.4	289.6 (255.4-342.4)	44.2 (36.9-53.1)	129.6 (108.9-152.7)	14.1 (-2.1-33.8)	20.4 (-3-48.5)	11.3 (-1.7-26.1)
Sudan	585.9	301.7 (273.6-330.2)	44.1 (37.9-51)	132.9 (115-152.4)	14.5 (-2.1-34.1)	21 (-3-48.6)	11.6 (-1.7-26.3)
Tunisia	102.4	208.9 (195.4-224.8)	48.6 (41.5-57)	101.4 (87.1-119)	11.1 (-1.6-26.4)	16 (-2.3-37.7)	8.8 (-1.3-20.5)
Yemen, Rep.	401.7	295.9 (282.1-309.6)	61.6 (58.9-64.5)	182.3 (170.8-194.1)	19.9 (-2.8-46.8)	28.9 (-4.1-66.8)	16 (-2.3-36.3)
**European Region**							
Albania	17.2	222.4 (210.5-234.6)	49.2 (33.3-65)	109.3 (74.3-146.1)	11.7 (-1.6-29.8)	16.9 (-2.3-42.6)	9.3 (-1.3-23)
Armenia	20.6	198.4 (186.9-210.1)	55.6 (37-77.8)	113.7 (74.7-151.8)	12.1 (-1.8-30.9)	17.5 (-2.6-44.6)	9.7 (-1.4-24)
Azerbaijan	86.6	214.2 (202.7-226.1)	43 (30-56.4)	91.9 (64-122.2)	9.8 (-1.4-24.8)	14.3 (-2-35.7)	7.9 (-1.1-19.3)
Belarus	57.6	202 (188.6-216.6)	42.9 (35.8-50.7)	86.8 (72.2-102.7)	9.5 (-1.4-22.6)	13.6 (-2-32.2)	7.6 (-1.1-17.5)
Bosnia and Herzegovina	16.2	201.1 (187-219.5)	44.8 (37.7-53.2)	90.5 (75-108.3)	9.8 (-1.4-23.6)	14.3 (-2-34)	7.9 (-1.2-18.2)
Bulgaria	33.6	199.3 (186.6-212.9)	43.8 (36.1-51.9)	87.4 (72.1-103.9)	9.5 (-1.4-22.6)	13.7 (-2-32.3)	7.6 (-1.1-17.5)
Georgia	27.5	209 (194.7-224.8)	44.6 (36.9-53.5)	93.1 (77.1-112.4)	10.2 (-1.5-24.3)	14.7 (-2.2-35.1)	8.1 (-1.2-19.1)
Kazakhstan	199.1	206.4 (193.4-221.5)	45.7 (38.8-53.5)	94.4 (79.7-110.4)	10.2 (-1.5-24.3)	14.8 (-2.1-35)	8.2 (-1.2-18.8)
Kosovo	15	210 (198.8-221.3)	50 (37.6-63.2)	104.9 (78.5-133.3)	11.4 (-1.6-27.9)	16.4 (-2.3-39.8)	9.1 (-1.3-21.6)
Kyrgyzstan	74.8	255.8 (243.5-268.9)	44.8 (30.4-60.7)	116 (76.1-158.9)	12.4 (-1.7-31.4)	18 (-2.5-45.3)	10 (-1.3-24.5)
Moldova, Rep.	22.2	218.5 (200.4-238.1)	47.9 (39.7-57.5)	104.7 (86.8-124.8)	11.4 (-1.7-27)	16.6 (-2.4-38.8)	9.1 (-1.3-21.1)
Montenegro	3.7	193 (180.7-205.9)	42.9 (19-66.7)	82.8 (37.1-130.3)	8.7 (-1.1-24.6)	12.5 (-1.6-35.4)	6.9 (-0.9-19.1)
North Macedonia	11.6	200.9 (186.5-216.5)	45.9 (38.2-54.8)	92.3 (76.2-111)	10 (-1.5-24)	14.5 (-2.1-34.4)	8 (-1.2-18.8)
Romania	97.1	204.9 (190.8-219.3)	43.9 (36.6-51.5)	89.8 (74.2-106.6)	9.8 (-1.4-23.2)	14.2 (-2-33.3)	7.8 (-1.1-17.9)
Russian Fed.	935.5	201.1 (187.4-215)	44.4 (37.6-51.6)	89.1 (75.7-104.2)	9.7 (-1.3-23.1)	14.1 (-1.9-33.2)	7.8 (-1.1-17.8)
Serbia	46.8	201.2 (187.8-215.9)	43.5 (36.2-52.1)	87.8 (72.4-105.3)	9.5 (-1.4-22.9)	13.8 (-2-32.7)	7.6 (-1.1-17.8)

Tajikistan	116.5	273.1 (259.2-288.4)	59.5 (42.9-73.8)	160.8 (117.9-205.1)	17.4 (-2.6-42.6)	25.2 (-3.7-60.9)	13.9 (-2.1-32.6)
Turkey	674	205.5 (192.7-220.4)	47 (39.6-55)	96.5 (80.9-113.6)	10.5 (-1.5-25.1)	15.3 (-2.2-35.9)	8.5 (-1.3-19.5)
Turkmenistan	67.9	193.6 (183-204.6)	35.3 (17.6-64.7)	71.4 (33-124)	7.5 (-1-22.8)	10.9 (-1.5-32.7)	6 (-0.9-17.5)
Ukraine	241	214.4 (198.4-232.8)	45.1 (37.6-53.3)	96.8 (80.5-115.5)	10.5 (-1.5-24.8)	15.2 (-2.1-35.2)	8.4 (-1.2-19.2)
Uzbekistan	319.7	238.8 (218.5-262.2)	49 (39.5-58.8)	116.6 (95.7-138.8)	12.7 (-1.8-30.4)	18.3 (-2.6-43.5)	10.1 (-1.4-23.6)
**Southeast Asia Region**							
Bangladesh	1525.4	301.8 (289.3-314.7)	53.4 (48.3-58.7)	161.5 (144.9-178.2)	17.6 (-2.5-41.4)	25.5 (-3.7-59.2)	14.1 (-2.1-32)
Bhutan	7.1	262.5 (235.7-290.3)	57 (47.4-68.1)	149.5 (126.5-175.7)	16.3 (-2.3-38.8)	23.6 (-3.3-55.3)	13 (-1.9-30.2)
India	12141.5	282.3 (271.2-293.2)	51.9 (49.7-53.9)	146.2 (138.7-154.8)	16 (-2.2-37.2)	23.2 (-3.2-53.5)	12.8 (-1.9-29.3)
Indonesia	2459.2	239 (228.9-249.3)	62.6 (56.7-68.6)	149.4 (134.3-165.5)	16.4 (-2.3-38.4)	23.7 (-3.4-55.3)	13.1 (-2-29.8)
Maldives	3.8	183.8 (171.2-197.7)	57.7 (38.5-76.9)	107 (70-143.6)	11.5 (-1.6-29.3)	16.6 (-2.3-42.1)	9.2 (-1.4-22.6)
Myanmar	455.3	317.2 (303.1-331.8)	65.2 (56.6-73.7)	206.7 (178.2-235)	22.6 (-3.3-53)	32.8 (-4.6-75.5)	18.1 (-2.5-41.4)
Nepal	280.5	313.6 (298.3-329.9)	52.3 (42.2-61.8)	163.8 (131.4-195.4)	17.9 (-2.5-42.8)	25.9 (-3.7-61.2)	14.3 (-2-33.3)
Sri Lanka	164.3	228.9 (210.6-249.4)	55.3 (46.1-66.3)	126.7 (105.5-152)	13.8 (-1.9-32.7)	19.9 (-2.8-47.1)	11 (-1.6-25.3)
Thailand	384.4	224.3 (212.7-235.6)	57.8 (48.1-66.9)	129.7 (107.6-151.4)	14 (-2-33.2)	20.3 (-2.9-47.8)	11.2 (-1.6-25.9)
Timor-Leste	20.4	290.8 (278.8-303.6)	63.6 (53-73.9)	185 (153.3-216.8)	20.3 (-2.8-47.8)	29.3 (-4-69.1)	16.2 (-2.2-37.2)
**Western Pacific Region**							
American Samoa	0.5	220.8 (197.6-243.9)	47.7 (38.9-59.2)	104.8 (85.6-130.6)	11.6 (-1.6-27.9)	16.8 (-2.3-40.2)	9.2 (-1.4-21.7)
Cambodia	176.5	321.8 (307.8-336.7)	66.6 (58.7-74.3)	214.1 (188.1-241.6)	23.5 (-3.4-55.2)	33.9 (-4.9-79.3)	18.7 (-2.9-42.8)
China, People's Rep.	8588.5	210.6 (195.7-228.6)	47.9 (40-57)	101 (84.6-119.5)	11 (-1.6-26.3)	15.9 (-2.4-37.3)	8.8 (-1.3-20.3)
Fiji	8.8	220.8 (204-240.1)	47 (39.6-56)	104 (87.2-124.4)	11.3 (-1.6-27)	16.4 (-2.3-38.6)	9 (-1.3-21)
Kiribati	1.5	248.3 (225.3-273.1)	48.7 (40.3-58.7)	121 (100.4-145.5)	13.2 (-1.8-31.4)	19.2 (-2.7-45.2)	10.6 (-1.5-24.3)
Lao People's Dem. Rep.	77.2	296.6 (284.3-310.1)	67.7 (57.4-77.6)	200.5 (169.2-233.4)	21.9 (-3.1-52.6)	31.6 (-4.6-75.5)	17.5 (-2.6-40.6)
Malaysia	256.9	207.7 (193-223.4)	49.2 (42.2-57.8)	102.3 (86.9-120.9)	11.2 (-1.6-26.4)	16.1 (-2.3-37.9)	8.9 (-1.3-20.5)
Marshall Islands	0.5	222.1 (202.5-244.4)	42.2 (35-50.6)	93.5 (78.4-112.1)	10.3 (-1.5-24.3)	14.8 (-2.1-35.1)	8.2 (-1.2-19.1)
Micronesia, Fed.	5.1	254.9 (232.6-279.9)	49.8 (40.7-60.6)	127 (104.6-153.3)	13.9 (-2.1-33)	20.2 (-3-47.5)	11.2 (-1.6-25.9)
Mongolia	35.9	224.5 (213.9-235.4)	52 (45.1-58.5)	116.4 (100.7-132.7)	12.8 (-1.8-29.8)	18.5 (-2.7-42.5)	10.2 (-1.5-23.1)
Nauru	0.1	224.6 (205.2-246.9)	48.7 (40.6-62)	109.5 (91.2-141.1)	12.1 (-1.8-29)	17.5 (-2.6-41.5)	9.7 (-1.4-22.5)
Papua New Guinea	102.5	291.1 (265.6-318.4)	50 (41.7-59.4)	145.4 (122.4-169.8)	15.9 (-2.3-37.8)	23.1 (-3.4-53.7)	12.7 (-1.9-29)
Philippines	1143.4	241.3 (231.8-251.1)	62.6 (53.5-71.6)	150.9 (128.4-174.1)	16.5 (-2.3-39)	23.8 (-3.4-55.7)	13.2 (-1.9-30)
Samoa	2.4	226.7 (207.4-247.7)	48.2 (40.4-57.4)	109.4 (92.6-129.3)	11.9 (-1.8-28.5)	17.3 (-2.5-41.1)	9.6 (-1.4-22)
Solomon Islands	8.3	289.2 (262.5-317.7)	49.1 (41-58.7)	141.8 (119.6-168.8)	15.4 (-2.3-36)	22.4 (-3.3-51.6)	12.4 (-1.8-28)
Tonga	1.3	236.7 (217.5-259)	52.8 (42.1-65.6)	125.1 (101.2-154.4)	13.6 (-1.9-32.6)	19.7 (-2.8-46.6)	10.9 (-1.6-25.2)
Tuvalu	0.1	213.8 (197.3-233.6)	45.5 (38.4-53.6)	97.5 (82.4-114.7)	10.6 (-1.5-25.3)	15.4 (-2.1-36.3)	8.5 (-1.2-19.9)

Vanuatu	3.5	278.9 (248.9-311.6)	47 (38.6-58.7)	132 (110.9-156.8)	14.4 (-2.2-34.4)	20.9 (-3.1-49.6)	11.5 (-1.8-26.7)
Viet Nam	775.3	238.3 (227.4-249.5)	57.9 (47.1-67.8)	137.6 (112.2-163.2)	14.9 (-2.2-35.9)	21.6 (-3.1-51.4)	11.9 (-1.7-27.7)

Incidence rate estimates are presented as cases per 100 children annually in the eligible age range. Estimates are obtained from analyses of DHS/MICS surveys covering 944,173 children across 77 countries, as well as extrapolations based on 405 health, nutrition, and population indicators for all LMICs. Quantiles are obtained via 5,000 independent draws from the distribution of estimates.

Table S17: Lower-bound country-specific estimates of the incidence of ARI and antibiotic-treated ARI among children ages 0-59 months preventable by 10- and 13-valent pneumococcal conjugate vaccines.

Country	Population ages 0-59 months	ARI attributable to all causes			ARI attributable to PCV10/13-type *Streptococcus pneumoniae*		
	Children (in 10,000s)	All cases, per 100 (95% CI)	Proportion treated with antibiotics, % (95% CI)	Antibiotic-treated cases, per 100 (95% CI)	Antibiotic-treated cases, per 100 (95% CI)		Antibiotic-treated cases preventable by vaccine direct effects, per 100 (95% CI)
					IPD probe estimate	*AOM probe estimate*	
**African Region**							
Algeria	466.4	66.7 (61.6-72.5)	46.5 (39.7-56.2)	31.1 (26.3-37.7)	3.4 (-0.5-8.1)	4.9 (-0.7-11.6)	2.7 (-0.4-6.3)
Angola	515.8	79.6 (75.9-83.3)	36.6 (31-42)	29.1 (24.6-33.6)	3.2 (-0.5-7.5)	4.6 (-0.7-10.8)	2.5 (-0.4-5.8)
Benin	173.9	110 (104.3-115.7)	41.4 (34.3-48.5)	45.5 (37.4-53.7)	5 (-0.7-11.7)	7.2 (-1-16.8)	4 (-0.6-9.1)
Botswana	25.6	71.9 (66-78.6)	41.9 (35.8-49.1)	30.1 (25.8-35.5)	3.3 (-0.5-7.8)	4.8 (-0.7-11.1)	2.6 (-0.4-6)
Burkina Faso	316.1	118.9 (113-125.3)	41.4 (35.6-47.3)	49.2 (41.8-56.9)	5.4 (-0.8-12.7)	7.8 (-1.1-18.3)	4.3 (-0.6-9.8)
Burundi	185.3	128.7 (120.2-137.7)	44.3 (40.4-48.3)	57 (50.5-63.7)	6.3 (-0.9-14.8)	9.1 (-1.3-21.2)	5 (-0.8-11.3)
Cabo Verde	5.5	73.3 (67.2-80.4)	46.5 (39.3-55)	34.1 (29-40.3)	3.7 (-0.5-8.9)	5.4 (-0.8-12.7)	3 (-0.4-6.8)
Cameroon	374.2	100.2 (95.9-104.5)	42.5 (38-46.7)	42.5 (37.7-47.5)	4.7 (-0.7-10.9)	6.7 (-0.9-15.7)	3.7 (-0.5-8.5)
Cent. African Rep.	72.8	110.8 (100.1-120.8)	40.2 (35.4-45.4)	44.5 (38.6-50.7)	4.9 (-0.7-11.4)	7 (-1-16.4)	3.9 (-0.6-8.9)
Chad	260.1	113.3 (107.8-118.9)	41.1 (38-44.2)	46.5 (42.5-50.8)	5.1 (-0.7-12)	7.4 (-1.1-17)	4.1 (-0.6-9.3)
Comoros	11.7	110.2 (104-116.8)	41 (31.7-51)	45.1 (34.6-56.5)	4.9 (-0.7-12)	7.1 (-1-17.3)	3.9 (-0.6-9.3)
Congo, Rep.	81.4	90.2 (86.6-94.2)	41.3 (36.8-46.1)	37.3 (32.9-41.8)	4 (-0.6-9.7)	5.9 (-0.8-13.8)	3.2 (-0.5-7.5)
Congo, Dem. Rep.	1409.9	117.3 (110.7-124.3)	44.6 (41.3-47.8)	52.2 (47.4-57.2)	5.7 (-0.8-13.5)	8.3 (-1.2-19.3)	4.6 (-0.7-10.5)
Cote d'Ivoire	376.5	102.6 (97.9-107.4)	39 (33.3-45.2)	40 (33.8-46.6)	4.3 (-0.6-10.4)	6.3 (-0.9-14.9)	3.5 (-0.5-8)
Equatorial Guinea	17.6	72.1 (65.4-80.4)	43.5 (35.6-52.7)	31.3 (25.7-38.1)	3.4 (-0.5-8.2)	5 (-0.7-11.7)	2.7 (-0.4-6.4)
Eritrea	74.4	105.1 (92-118.5)	46.1 (39-55.1)	48.4 (40.5-57.1)	5.3 (-0.8-12.6)	7.7 (-1.1-18.2)	4.2 (-0.6-9.8)
Eswatini	17.8	86.3 (81.5-91.4)	38.7 (32.3-45.5)	33.4 (27.7-39.7)	3.7 (-0.5-8.7)	5.3 (-0.7-12.4)	2.9 (-0.4-6.7)
Ethiopia	1490.1	113.4 (108.1-119.2)	38.9 (34-43.6)	44.1 (38.4-50)	4.8 (-0.7-11.4)	7 (-1-16.3)	3.9 (-0.6-8.7)
Gabon	26.7	65 (61.1-69.1)	36.3 (31.6-41.4)	23.6 (20.4-27.2)	2.6 (-0.4-6.1)	3.7 (-0.6-8.8)	2.1 (-0.3-4.7)
Gambia, The	35.2	113 (107-119.4)	42.6 (37.4-47.7)	48.2 (42-54.6)	5.2 (-0.8-12.3)	7.6 (-1.1-17.6)	4.2 (-0.6-9.6)
Ghana	402.3	93.8 (89.8-98)	43.2 (36.3-50.5)	40.6 (33.7-47.9)	4.4 (-0.6-10.4)	6.4 (-0.9-15)	3.5 (-0.5-8.1)
Guinea	194.9	115.3 (109.5-121.5)	40.6 (35.5-45.4)	46.7 (40.5-53)	5.1 (-0.7-12)	7.4 (-1.1-17.4)	4.1 (-0.6-9.4)
Guinea-Bissau	28.6	81.2 (76.5-86.1)	41.7 (34.1-49.4)	33.8 (27.5-40.3)	3.7 (-0.5-8.8)	5.3 (-0.7-12.5)	3 (-0.4-6.8)
Kenya	699.7	96.5 (92.8-100.4)	39.8 (37-42.6)	38.4 (35.5-41.5)	4.2 (-0.6-9.8)	6.1 (-0.9-14.1)	3.4 (-0.5-7.7)
Lesotho	28	95.7 (90.5-101)	41.6 (33.6-50)	39.9 (32.1-48.6)	4.4 (-0.6-10.4)	6.3 (-0.9-14.9)	3.5 (-0.5-8)
Liberia	70.5	111.3 (105-117.5)	43.1 (38.8-47.6)	48 (42.5-53.7)	5.3 (-0.8-12.3)	7.6 (-1.1-17.6)	4.2 (-0.6-9.6)
Madagascar	370	108 (99.5-117)	47.3 (41-54.4)	51.1 (44-58.9)	5.6 (-0.8-13.1)	8.1 (-1.2-18.9)	4.5 (-0.7-10.1)
Malawi	288.8	125.1 (117-133.8)	45.6 (41.6-49.5)	57.1 (51-63.3)	6.2 (-0.9-14.8)	9 (-1.3-21.1)	5 (-0.7-11.5)
Mali	327.4	114.2 (108.5-119.8)	36.1 (29.3-43.9)	41.4 (32.9-50.4)	4.5 (-0.6-10.7)	6.5 (-0.9-15.5)	3.6 (-0.5-8.4)
Mauritania	64	83.8 (79.9-87.8)	39.9 (34.7-45.1)	33.4 (28.8-38.1)	3.7 (-0.5-8.6)	5.3 (-0.8-12.3)	2.9 (-0.4-6.7)
Mauritius	6.9	66.6 (62-72.2)	44.6 (37.4-53.5)	29.8 (25.1-35.5)	3.3 (-0.5-7.8)	4.7 (-0.7-11.1)	2.6 (-0.4-6)
Mozambique	484.4	122.8 (115-131)	43.1 (37.3-49)	52.9 (45.2-61.1)	5.8 (-0.8-13.7)	8.4 (-1.2-19.6)	4.6 (-0.7-10.6)
Namibia	33.5	76.3 (72.3-80.5)	36.5 (30.6-42.7)	27.8 (23.2-32.7)	3 (-0.4-7.2)	4.4 (-0.6-10.3)	2.4 (-0.4-5.6)
Niger	406.6	127.1 (119.6-135.1)	41.6 (37-46.1)	52.8 (46.3-59.5)	5.7 (-0.8-13.5)	8.3 (-1.2-19.5)	4.6 (-0.7-10.6)
Nigeria	3110.9	97.1 (93.6-100.9)	42.7 (38-47.4)	41.5 (36.8-46.4)	4.5 (-0.6-10.6)	6.6 (-0.9-15.1)	3.6 (-0.5-8.2)
Rwanda	173.5	114.1 (108.1-120.6)	42.8 (37.9-47.6)	48.8 (42.9-55.1)	5.3 (-0.8-12.5)	7.7 (-1.1-18)	4.3 (-0.6-9.8)
Sao Tome and Principe	3.1	97.2 (91.5-103.4)	43.7 (35-52.6)	42.5 (33.7-51.8)	4.6 (-0.7-11.2)	6.7 (-1-16.1)	3.7 (-0.5-8.6)
Senegal	249.3	101.3 (97-105.6)	40.1 (35.4-45)	40.6 (35.6-45.9)	4.4 (-0.6-10.5)	6.4 (-0.9-15.1)	3.5 (-0.5-8.1)

Sierra Leone	113.5	121.4 (114.4-128.7)	42.8 (39.3-46.5)	52 (46.8-57.6)	5.7 (-0.8-13.4)	8.3 (-1.2-19.3)	4.6 (-0.7-10.4)
South Africa	566.4	64.3 (60.3-68.3)	36.8 (26.3-48.1)	23.8 (16.7-31.2)	2.6 (-0.3-6.3)	3.7 (-0.5-9.1)	2 (-0.3-5)
South Sudan	188.2	103.9 (93-117.4)	41.7 (35-50.5)	43.9 (36.3-50.8)	4.8 (-0.7-11.3)	7 (-1-16.1)	3.8 (-0.6-8.7)
Tanzania, United Rep.	941.9	109.5 (104.4-114.8)	41.6 (35.9-47.3)	45.5 (39.2-52.4)	4.9 (-0.7-11.8)	7.1 (-1-16.9)	3.9 (-0.6-9.1)
Togo	116.2	114.2 (107.9-120.6)	42.3 (35.9-48.4)	48.2 (40.7-55.9)	5.3 (-0.7-12.4)	7.7 (-1.1-17.7)	4.2 (-0.6-9.6)
Uganda	751.2	111.7 (106.2-117.6)	48.4 (44.3-52.6)	54.1 (48.7-59.9)	5.9 (-0.8-14)	8.5 (-1.2-20)	4.7 (-0.7-10.7)
Zambia	275	99 (94.9-103.2)	40.1 (35.5-44.9)	39.7 (34.9-44.7)	4.3 (-0.6-10.3)	6.3 (-0.9-14.7)	3.5 (-0.5-8)
Zimbabwe	250.6	93.9 (89.6-98.4)	40.5 (33.7-47.7)	38.1 (31.4-45.3)	4.2 (-0.6-9.9)	6 (-0.9-14.2)	3.3 (-0.5-7.6)
**Region of the Americas**							
Belize	3.9	74.3 (70-78.9)	38.5 (28.1-48.4)	28.5 (21-36.4)	3.1 (-0.4-7.5)	4.5 (-0.6-10.9)	2.5 (-0.4-6)
Bolivia, Plurinational State	118.6	77.5 (74-81.2)	68.3 (61.7-75.1)	52.9 (47.2-58.6)	5.8 (-0.8-13.7)	8.4 (-1.2-19.6)	4.6 (-0.7-10.6)
Brazil	1487.5	65.8 (61.6-70.5)	43.4 (37.6-50.5)	28.6 (24.7-33.2)	3.1 (-0.4-7.4)	4.5 (-0.6-10.7)	2.5 (-0.4-5.7)
Colombia	373.8	63.1 (60-66.3)	38.7 (35.5-42.2)	24.4 (22-27.1)	2.7 (-0.4-6.3)	3.8 (-0.6-9.1)	2.1 (-0.3-4.9)
Costa Rica	35	63.7 (59.6-68.3)	43.2 (36.9-50.5)	27.6 (23.5-32.1)	3 (-0.4-7.2)	4.4 (-0.6-10.3)	2.4 (-0.4-5.6)
Cuba	62.9	62.1 (58.4-65.7)	32.6 (27.1-38.5)	20.3 (16.7-24.1)	2.2 (-0.3-5.3)	3.2 (-0.5-7.7)	1.8 (-0.3-4.1)
Dominica	0.6	67.2 (61.8-73.2)	43.9 (37.1-51.6)	29.5 (24.9-35.1)	3.2 (-0.5-7.7)	4.7 (-0.7-11)	2.6 (-0.4-6)
Dominican Rep.	106.2	70.6 (67.1-74)	34.4 (31.6-37.3)	24.3 (22-26.6)	2.7 (-0.4-6.2)	3.8 (-0.6-8.9)	2.1 (-0.3-4.8)
Ecuador	161	65.5 (61.3-70)	44 (38.5-50)	28.8 (25.1-33)	3.1 (-0.4-7.5)	4.6 (-0.6-10.7)	2.5 (-0.4-5.8)
El Salvador	58	67.6 (64.2-71)	35.8 (31.2-40.3)	24.1 (20.9-27.5)	2.6 (-0.4-6.2)	3.8 (-0.5-8.9)	2.1 (-0.3-4.8)
Grenada	1	67.5 (62.3-73.4)	45.8 (37.2-54.9)	30.9 (25-37.3)	3.4 (-0.5-8)	4.9 (-0.7-11.5)	2.7 (-0.4-6.2)
Guatemala	199.4	83.3 (79.7-87.2)	39.4 (36.3-42.5)	32.8 (29.9-35.9)	3.6 (-0.5-8.4)	5.2 (-0.8-12)	2.9 (-0.4-6.5)
Guyana	7.5	75.5 (70.8-80.4)	39.8 (30.8-48.7)	30 (23.1-37.3)	3.2 (-0.5-8)	4.7 (-0.7-11.4)	2.6 (-0.4-6.2)
Haiti	123.8	106.9 (101.5-112.6)	41.9 (37.4-46.9)	44.9 (39.5-50.6)	4.9 (-0.7-11.4)	7.1 (-1-16.5)	3.9 (-0.6-8.9)
Honduras	95.5	85.8 (82.3-89.3)	42.1 (39.1-45.3)	36.1 (33.1-39.4)	4 (-0.6-9.3)	5.7 (-0.8-13.3)	3.2 (-0.5-7.2)
Jamaica	20.7	66.7 (61.9-72.5)	42.2 (36.3-48.6)	28.2 (24.1-32.5)	3.1 (-0.4-7.2)	4.5 (-0.6-10.4)	2.5 (-0.4-5.6)
Mexico	1153.3	64.9 (61.2-68.8)	47.5 (39.5-56.6)	30.9 (25.4-37.3)	3.4 (-0.5-8.1)	4.9 (-0.7-11.6)	2.7 (-0.4-6.3)
Nicaragua	60.6	75.4 (68.5-83.1)	47.6 (40.1-56.9)	35.9 (30.2-42.8)	3.9 (-0.6-9.4)	5.7 (-0.8-13.3)	3.1 (-0.4-7.2)
Paraguay	67.4	63.1 (59.5-66.7)	57.1 (51.2-63)	36 (31.8-40.4)	3.9 (-0.6-9.3)	5.7 (-0.8-13.4)	3.1 (-0.4-7.2)
Peru	302	66.8 (63.5-70.4)	58.9 (52.8-65.2)	39.4 (34.8-44.2)	4.3 (-0.6-10.1)	6.3 (-0.9-14.5)	3.4 (-0.5-7.8)
St. Lucia	1.1	67.5 (62.1-73.2)	43.7 (36.4-51.5)	29.5 (24.7-34.8)	3.2 (-0.5-7.6)	4.6 (-0.7-10.9)	2.6 (-0.4-5.9)
St. Vincent and the Grenadines	0.9	67.7 (62.4-73.8)	43.4 (36.6-51.2)	29.4 (24.8-34.9)	3.2 (-0.5-7.7)	4.7 (-0.7-11)	2.6 (-0.4-5.9)
Suriname	5	68.9 (63-75.7)	43.4 (36.7-51.3)	29.9 (25.2-35.3)	3.3 (-0.5-7.7)	4.7 (-0.7-11.1)	2.6 (-0.4-6)
Venezuela, Bolivarian Rep.	297.5	66.3 (61.6-71.8)	45.8 (38.6-53.9)	30.5 (25.3-35.8)	3.3 (-0.5-7.8)	4.8 (-0.7-11.3)	2.6 (-0.4-6.1)
**Eastern Mediterranean Region**							
Afghanistan	523.9	112.8 (106.8-118.9)	61.5 (58.9-64)	69.3 (64.7-74.2)	7.6 (-1.1-17.8)	11 (-1.6-25.7)	6.1 (-0.9-13.9)
Djibouti	10.1	81.9 (74.4-90.5)	45.9 (38.4-54.4)	37.7 (31.8-44.1)	4.1 (-0.6-9.8)	5.9 (-0.8-14)	3.3 (-0.4-7.5)
Egypt, Arab Rep.	1237.4	81.8 (79-84.8)	57.9 (55.3-60.7)	47.4 (44.7-50.2)	5.2 (-0.8-12.1)	7.5 (-1.1-17.3)	4.1 (-0.6-9.4)
Iran, Islamic Rep.	689.9	65.8 (61.3-70.9)	47.4 (41.1-54.7)	31.2 (27.1-36)	3.4 (-0.5-8.1)	4.9 (-0.7-11.5)	2.7 (-0.4-6.2)
Iraq	560.3	74.2 (67.6-81.9)	47.7 (39.9-56.7)	35.3 (29.8-42.5)	3.9 (-0.6-9.2)	5.6 (-0.8-13.2)	3.1 (-0.4-7.2)
Jordan	120.2	62 (58.9-65.1)	55.8 (51.6-60.3)	34.6 (31.5-37.9)	3.8 (-0.6-9)	5.5 (-0.8-12.9)	3 (-0.5-6.9)
Lebanon	46.1	63.6 (58.2-69.9)	48.9 (41.8-57.1)	31.1 (26.6-36.2)	3.4 (-0.5-8.1)	5 (-0.7-11.6)	2.7 (-0.4-6.2)
Libyan Arab Rep.	63	69.9 (62.5-80.8)	49 (40.4-59.2)	34.4 (28.5-41.5)	3.8 (-0.5-9)	5.4 (-0.8-13)	3 (-0.4-7)
Morocco	345.7	74.5 (68.2-81.7)	49.3 (41.5-58.6)	36.8 (31.4-43.2)	4 (-0.6-9.6)	5.8 (-0.8-13.7)	3.2 (-0.5-7.4)
Pakistan	2471.2	92.4 (88.9-96.3)	53.6 (50.4-56.7)	49.6 (46-53.2)	5.4 (-0.8-12.6)	7.9 (-1.1-18.2)	4.3 (-0.6-9.8)
Palestinian Terr.	69.9	71.5 (68-75)	53.1 (49.1-57.2)	37.9 (34.6-41.3)	4.1 (-0.6-9.7)	6 (-0.9-13.8)	3.3 (-0.5-7.5)
Somalia	255.4	91.6 (80.4-108.5)	44.2 (36.9-53.1)	40.9 (34.4-48)	4.5 (-0.7-10.6)	6.4 (-0.9-15.2)	3.6 (-0.5-8.3)
Sudan	585.9	95.2 (86-104.4)	44.1 (37.9-51)	41.9 (36.3-48.1)	4.6 (-0.7-10.7)	6.6 (-1-15.3)	3.7 (-0.5-8.4)
Tunisia	102.4	66 (61.3-71.5)	48.6 (41.5-57)	32.1 (27.5-37.4)	3.5 (-0.5-8.3)	5.1 (-0.7-11.9)	2.8 (-0.4-6.4)

Yemen, Rep.	401.7	93.4 (89.6-97.3)	61.6 (58.9-64.5)	57.5 (54.1-61.1)	6.3 (-0.9-14.7)	9.1 (-1.3-21)	5 (-0.7-11.4)
**European Region**							
Albania	17.2	70.2 (65.9-74.7)	49.2 (33.3-65)	34.4 (23.4-46.4)	3.7 (-0.5-9.5)	5.3 (-0.8-13.5)	2.9 (-0.4-7.3)
Armenia	20.6	62.7 (58-67.5)	55.6 (37-77.8)	35.9 (23.7-48.2)	3.8 (-0.6-9.9)	5.5 (-0.8-14.2)	3.1 (-0.5-7.6)
Azerbaijan	86.6	67.6 (63.2-72.1)	43 (30-56.4)	29 (20.2-38.6)	3.1 (-0.4-7.9)	4.5 (-0.6-11.3)	2.5 (-0.4-6.1)
Belarus	57.6	63.8 (59.6-68.6)	42.9 (35.8-50.7)	27.4 (22.7-32.4)	3 (-0.4-7.1)	4.3 (-0.6-10.2)	2.4 (-0.3-5.5)
Bosnia and Herzegovina	16.2	63.6 (59.2-68.9)	44.8 (37.7-53.2)	28.5 (23.8-34)	3.1 (-0.5-7.4)	4.5 (-0.7-10.7)	2.5 (-0.4-5.7)
Bulgaria	33.6	63 (58.6-67.8)	43.8 (36.1-51.9)	27.5 (22.7-32.9)	3 (-0.4-7.2)	4.3 (-0.6-10.3)	2.4 (-0.4-5.5)
Georgia	27.5	66 (61.1-71.1)	44.6 (36.9-53.5)	29.4 (24.3-35.3)	3.2 (-0.5-7.7)	4.6 (-0.7-11.1)	2.6 (-0.4-6)
Kazakhstan	199.1	65.2 (61-69.9)	45.7 (38.8-53.5)	29.8 (25.3-34.8)	3.2 (-0.5-7.7)	4.7 (-0.7-11.1)	2.6 (-0.4-5.9)
Kosovo	15	66.2 (61.8-70.9)	50 (37.6-63.2)	33 (24.8-42.2)	3.6 (-0.5-8.8)	5.2 (-0.7-12.6)	2.9 (-0.4-6.8)
Kyrgyzstan	74.8	80.8 (76.6-85.1)	44.8 (30.4-60.7)	36.6 (24.2-50.1)	3.9 (-0.5-9.9)	5.7 (-0.8-14.2)	3.1 (-0.4-7.7)
Moldova, Rep.	22.2	69 (63.4-75.1)	47.9 (39.7-57.5)	33 (27.5-39.5)	3.6 (-0.5-8.5)	5.2 (-0.8-12.3)	2.9 (-0.4-6.7)
Montenegro	3.7	61.1 (56.1-66.3)	42.9 (19-66.7)	26.1 (11.8-41.5)	2.7 (-0.4-7.7)	4 (-0.5-11.1)	2.2 (-0.3-6)
North Macedonia	11.6	63.5 (58.9-68.4)	45.9 (38.2-54.8)	29.1 (24-35)	3.2 (-0.5-7.5)	4.6 (-0.7-10.8)	2.5 (-0.4-5.9)
Romania	97.1	64.7 (60-69.5)	43.9 (36.6-51.5)	28.4 (23.6-33.6)	3.1 (-0.4-7.3)	4.5 (-0.6-10.5)	2.5 (-0.4-5.7)
Russian Fed.	935.5	63.6 (58.8-68.2)	44.4 (37.6-51.6)	28.1 (23.9-33)	3.1 (-0.4-7.3)	4.5 (-0.6-10.5)	2.5 (-0.4-5.6)
Serbia	46.8	63.6 (59.1-68.6)	43.5 (36.2-52.1)	27.7 (23.1-33.1)	3 (-0.4-7.3)	4.4 (-0.6-10.4)	2.4 (-0.3-5.6)
Tajikistan	116.5	86.4 (81.8-91)	59.5 (42.9-73.8)	51 (37.1-64.8)	5.5 (-0.8-13.5)	8 (-1.2-19.2)	4.4 (-0.7-10.4)
Turkey	674	64.9 (60.4-69.8)	47 (39.6-55)	30.5 (25.4-35.9)	3.3 (-0.5-8)	4.8 (-0.7-11.4)	2.7 (-0.4-6.2)
Turkmenistan	67.9	61.1 (57.5-65)	35.3 (17.6-64.7)	22.8 (10.4-39)	2.4 (-0.3-7.1)	3.4 (-0.5-10.3)	1.9 (-0.3-5.5)
Ukraine	241	67.7 (62.6-73.6)	45.1 (37.6-53.3)	30.5 (25.4-36.5)	3.3 (-0.5-7.9)	4.8 (-0.7-11.2)	2.7 (-0.4-6.1)
Uzbekistan	319.7	75.4 (69.1-82.7)	49 (39.5-58.8)	36.8 (30.2-44)	4 (-0.6-9.6)	5.8 (-0.8-13.8)	3.2 (-0.5-7.5)
**Southeast Asia Region**							
Bangladesh	1525.4	95.3 (91.5-99.2)	53.4 (48.3-58.7)	51 (45.7-56.4)	5.6 (-0.8-13)	8 (-1.2-18.7)	4.5 (-0.6-10.2)
Bhutan	7.1	82.9 (74.2-91.9)	57 (47.4-68.1)	47.2 (39.8-55.8)	5.1 (-0.7-12.3)	7.5 (-1-17.4)	4.1 (-0.6-9.4)
India	12141.5	89.2 (86.4-92)	51.9 (49.7-53.9)	46.2 (43.8-48.6)	5.1 (-0.7-11.9)	7.3 (-1-17)	4.1 (-0.6-9.2)
Indonesia	2459.2	75.4 (72.5-78.5)	62.6 (56.7-68.6)	47.2 (42.4-52.3)	5.2 (-0.7-12.2)	7.5 (-1.1-17.4)	4.1 (-0.6-9.5)
Maldives	3.8	58 (53.8-62.7)	57.7 (38.5-76.9)	33.8 (22.1-45.6)	3.6 (-0.5-9.3)	5.3 (-0.7-13.3)	2.9 (-0.4-7.2)
Myanmar	455.3	100.2 (95.5-105)	65.2 (56.6-73.7)	65.3 (56.2-74.2)	7.1 (-1-16.8)	10.4 (-1.4-24)	5.7 (-0.8-13)
Nepal	280.5	99 (94-104.4)	52.3 (42.2-61.8)	51.8 (41.7-61.7)	5.7 (-0.8-13.5)	8.2 (-1.2-19.4)	4.5 (-0.6-10.5)
Sri Lanka	164.3	72.2 (66.5-78.8)	55.3 (46.1-66.3)	40 (33.6-47.8)	4.4 (-0.6-10.2)	6.3 (-0.9-14.8)	3.5 (-0.5-8)
Thailand	384.4	70.8 (67.3-74.6)	57.8 (48.1-66.9)	40.9 (34.1-47.8)	4.4 (-0.7-10.4)	6.4 (-1-15.1)	3.6 (-0.5-8.1)
Timor-Leste	20.4	91.9 (88.2-95.6)	63.6 (53-73.9)	58.4 (48.3-68.4)	6.4 (-0.9-15.1)	9.2 (-1.2-21.7)	5.1 (-0.7-11.8)
**Western Pacific Region**							
American Samoa	0.5	69.5 (62.5-76.8)	47.7 (38.9-59.2)	33.1 (27-41.6)	3.6 (-0.5-8.8)	5.3 (-0.8-12.7)	2.9 (-0.4-6.9)
Cambodia	176.5	101.6 (97.5-106)	66.6 (58.7-74.3)	67.7 (59.1-76.1)	7.4 (-1.1-17.5)	10.7 (-1.6-25.2)	5.9 (-0.9-13.6)
China, People's Rep.	8588.5	66.5 (61.6-72.3)	47.9 (40-57)	32 (26.6-37.9)	3.5 (-0.5-8.3)	5 (-0.7-12)	2.8 (-0.4-6.4)
Fiji	8.8	69.7 (64-76.5)	47 (39.6-56)	32.9 (27.3-39.3)	3.6 (-0.5-8.5)	5.2 (-0.7-12.2)	2.9 (-0.4-6.6)
Kiribati	1.5	78.3 (71.2-86.5)	48.7 (40.3-58.7)	38.3 (31.5-45.9)	4.2 (-0.6-9.9)	6.1 (-0.8-14.3)	3.4 (-0.5-7.7)
Lao People's Dem. Rep.	77.2	93.8 (89.9-97.7)	67.7 (57.4-77.6)	63.4 (53.7-73.3)	6.9 (-1-16.5)	10 (-1.4-23.6)	5.5 (-0.8-12.9)
Malaysia	256.9	65.7 (60.6-70.7)	49.2 (42.2-57.8)	32.4 (27.2-38.2)	3.5 (-0.5-8.4)	5.1 (-0.7-12)	2.8 (-0.4-6.5)
Marshall Islands	0.5	70.1 (63.8-77)	42.2 (35-50.6)	29.5 (24.8-35.5)	3.2 (-0.5-7.7)	4.7 (-0.7-11.1)	2.6 (-0.4-6)
Micronesia, Fed.	5.1	80.6 (73-88.9)	49.8 (40.7-60.6)	40.2 (33-48.5)	4.4 (-0.7-10.5)	6.4 (-0.9-15)	3.5 (-0.5-8.1)
Mongolia	35.9	70.9 (67.5-74.6)	52 (45.1-58.5)	36.8 (31.9-42)	4 (-0.6-9.4)	5.8 (-0.8-13.5)	3.2 (-0.5-7.3)
Nauru	0.1	71.1 (64.4-78.2)	48.7 (40.6-62)	34.6 (28.7-44.5)	3.8 (-0.6-9.2)	5.5 (-0.8-13.2)	3.1 (-0.4-7.1)
Papua New Guinea	102.5	91.9 (83.9-100.8)	50 (41.7-59.4)	45.9 (38.5-54.3)	5 (-0.7-11.9)	7.3 (-1.1-17)	4 (-0.6-9.2)

Philippines	1143.4	76.2 (73.3-79.3)	62.6 (53.5-71.6)	47.7 (40.5-55.1)	5.2 (-0.7-12.3)	7.5 (-1.1-17.7)	4.2 (-0.6-9.6)
Samoa	2.4	71.6 (65.3-78.4)	48.2 (40.4-57.4)	34.5 (29.2-41.1)	3.8 (-0.6-9)	5.4 (-0.8-12.9)	3 (-0.4-6.9)
Solomon Islands	8.3	91.5 (83.2-100.2)	49.1 (41-58.7)	44.8 (37.9-53.1)	4.9 (-0.7-11.5)	7.1 (-1-16.4)	3.9 (-0.6-8.9)
Tonga	1.3	74.9 (68.8-81.9)	52.8 (42.1-65.6)	39.6 (32-48.8)	4.3 (-0.6-10.3)	6.2 (-0.9-14.8)	3.4 (-0.5-8)
Tuvalu	0.1	67.5 (61.9-74.1)	45.5 (38.4-53.6)	30.8 (26.2-36.1)	3.4 (-0.5-8.1)	4.9 (-0.7-11.6)	2.7 (-0.4-6.3)
Vanuatu	3.5	88.1 (78.2-98.8)	47 (38.6-58.7)	41.7 (35-49.7)	4.6 (-0.7-10.9)	6.6 (-1-15.6)	3.6 (-0.5-8.4)
Viet Nam	775.3	75.2 (71.2-79.5)	57.9 (47.1-67.8)	43.4 (35.4-51.5)	4.7 (-0.7-11.3)	6.8 (-1-16.3)	3.8 (-0.5-8.9)

Incidence rate estimates are presented as cases per 100 children annually in the eligible age range. Estimates are obtained from analyses of DHS/MICS surveys covering 944,173 children across 77 countries, as well as extrapolations based on 405 health, nutrition, and population indicators for all LMICs. Quantiles are obtained via 5,000 independent draws from the distribution of estimates.

Table S18: Upper-bound country-specific estimates of the incidence of diarrhea and antibiotic-treated diarrhea among children ages 0-23 months preventable by rotavirus vaccination.

Country	Population ages 0-23 months	Diarrhea attributable to all causes			Diarrhea attributable to rotavirus	
	Children (in 10,000s)	All cases, per 100 (95% CI)	Proportion treated with antibiotics, % (95% CI)	Antibiotic-treated cases, per 100 (95% CI)	Antibiotic-treated cases, per 100 (95% CI)	Antibiotic-treated cases preventable by vaccine direct effects, per 100 (95% CI)
**African region**						
Algeria	191.3	557 (510.2-609.4)	18.9 (14.9-23.6)	105.2 (82.8-132.5)	22.5 (0-45.1)	17.3 (0-34.7)
Angola	215.1	785.6 (735-840.7)	15.4 (13.2-17.8)	121.4 (102.1-141.8)	25.9 (0-50.5)	20 (0-38.5)
Benin	72.6	905 (844-971.4)	17.2 (13.9-20.7)	155.9 (124.3-190)	32.2 (5.9-98.4)	15.5 (3.3-30.1)
Botswana	10.6	638.5 (579.8-701)	18.4 (14.4-24.2)	117.4 (91.6-155.2)	25.3 (0-50.7)	19.5 (0-38.7)
Burkina Faso	131	992.2 (921.2-1069.2)	16.6 (14.5-18.8)	164.8 (140.9-191.1)	34.3 (6-99.2)	16.6 (3.3-30.6)
Burundi	77.9	1066.9 (980.6-1158.7)	16 (14.1-17.9)	170.4 (145.8-196.8)	35.7 (6.3-101.9)	17.1 (3.5-31.5)
Cabo Verde	2.2	612.4 (555.5-679)	19.7 (15.1-25.2)	120.4 (92.6-156.1)	25.9 (0-51.3)	20 (0-39.4)
Cameroon	155.1	835.3 (782.7-890.4)	19.6 (17.2-22.1)	163.3 (141.4-186.9)	34.8 (0-68.4)	27.1 (0-52.6)
Cent. African Rep.	30.2	910.9 (839.8-986)	15.5 (12.8-18)	140.8 (114.7-166.8)	29.4 (4.9-85.9)	14.2 (2.9-26.5)
Chad	109.6	976.2 (907.9-1046.5)	16.4 (14.6-18.2)	160.4 (139.5-182.1)	33.3 (5.9-98.9)	16 (3.3-29.5)
Comoros	4.9	871.7 (809.9-938.3)	17.5 (13.5-21.8)	152.3 (116.1-191.4)	31.5 (5.6-95.7)	15.2 (3-28.9)
Congo, Rep.	33.5	796.5 (744.4-846.6)	17.9 (15.4-20.5)	142.3 (120.9-165.2)	30.4 (0-59.1)	23.5 (0-45.7)
Congo, Dem. Rep.	595.6	982.1 (907.1-1063.9)	18.4 (16.4-20.3)	180.3 (158-206)	37.4 (6.7-109.9)	18.1 (3.7-33.2)
Cote d'Ivoire	158.3	864.7 (811.4-921.7)	17.1 (14.5-20.2)	148.4 (123-177)	31.5 (0-61.9)	24.3 (0-47.7)
Equatorial Guinea	7.4	656.4 (591.4-732.9)	20.4 (15.4-26.3)	133.7 (101.5-172.3)	28.5 (0-57.7)	22 (0-44.4)
Eritrea	29.7	870.4 (786.7-959.8)	19.6 (16.2-24.1)	170.4 (141.1-209.2)	35.9 (6.3-104.8)	17.2 (3.4-32.6)
Eswatini	7.2	712.4 (665.6-762.8)	16.2 (11.8-21)	115.3 (82.3-151.8)	24.1 (0-50.7)	18.6 (0-38.7)
Ethiopia	619.3	965 (900.6-1034.1)	15.6 (12.9-18.6)	151 (121.5-182.1)	31.2 (5.7-92.2)	15.1 (3.2-28.4)
Gabon	11.2	585.1 (548.6-625.2)	15.3 (12.1-18.5)	89.1 (69.7-109.6)	19 (0-38.2)	14.7 (0-29.4)
Gambia, The	14.8	922.4 (855.5-990.7)	19.2 (16.5-21.9)	176.8 (148.9-206.8)	36.4 (6.4-107.1)	17.7 (3.6-32.8)
Ghana	166.5	794.7 (744.4-848.2)	20.9 (16.8-25.7)	166.6 (130.8-206.7)	35.6 (0-71.3)	27.4 (0-55.1)
Guinea	80.9	951.5 (886.3-1020.1)	17.1 (13.9-20.5)	162.5 (131.1-196.9)	33.8 (6-100)	16.2 (3.2-30.7)
Guinea-Bissau	11.9	852.8 (791.7-916.9)	16.5 (12.7-20.5)	140.8 (107.5-177)	29 (5.3-86.7)	14 (2.8-26.9)
Kenya	280.4	824.9 (773-880.2)	16.3 (14.5-18.2)	134.7 (117.6-153)	28.9 (0-56.2)	22.4 (0-43.1)
Lesotho	11.7	784.3 (731.1-840.8)	16.3 (11.9-21.6)	129 (91.9-170.6)	27.3 (0-56.1)	21.2 (0-43.1)
Liberia	29	942.4 (875.1-1014.4)	18.3 (15.8-21.1)	172.3 (145.6-201.3)	35.6 (6.4-106)	17.3 (3.5-32.5)
Madagascar	152.4	892.7 (819.6-967.9)	18.1 (15.2-21.6)	161.5 (136.3-193.4)	34.2 (5.6-97.6)	16.3 (3.1-30.7)
Malawi	115.7	1018.1 (938.4-1107.4)	16.9 (15.2-18.8)	172.4 (150.6-196.6)	35.9 (6.3-106.4)	17.3 (3.5-31.8)
Mali	135.9	963 (893-1038.5)	17.1 (13.6-20.6)	163.9 (130.4-201.5)	34.2 (6-100.9)	16.3 (3.2-31.1)
Mauritania	26.7	829.5 (776.7-886)	16.7 (14.3-19.3)	138.7 (117.1-162.2)	29.6 (0-58)	22.8 (0-44.2)
Mauritius	2.7	561.9 (519.1-609.5)	18 (13-23.9)	101.5 (72-134.5)	21.4 (0-43.7)	16.6 (0-33.8)
Mozambique	202.3	1023.3 (943.4-1106.6)	17.8 (14.1-22.1)	183.1 (140.3-229.3)	37.7 (6.7-114.3)	18.2 (3.5-35.3)
Namibia	14.2	724.8 (676.2-777.3)	15.6 (12.4-19.2)	113.7 (88.8-141)	24.2 (0-48.6)	18.7 (0-37.3)
Niger	173.9	1011 (935.1-1091.9)	16.4 (14.1-18.9)	165.5 (138.9-194.5)	34.2 (6.2-102.2)	16.5 (3.5-31.3)
Nigeria	1306.7	857.3 (803.6-912.1)	21.1 (18.9-23.7)	181.3 (159.7-205.4)	39.1 (0-75.1)	30.1 (0-57.8)
Rwanda	69.8	887.9 (828.1-950.2)	16.5 (13.6-19.9)	147.1 (118.5-179.1)	30.6 (5.4-91.4)	14.8 (3-27.4)
Sao Tome and Principe	1.3	783 (731-841.7)	20.3 (13.8-27.6)	161.4 (107-221.4)	33.7 (0-71.1)	25.9 (0-54.5)
Senegal	103.5	860.6 (804.4-918.3)	17.2 (15-19.3)	148.1 (127.3-169.4)	30.5 (5.5-89)	14.8 (3.1-27.3)
Sierra Leone	46.2	992.8 (917.5-1072.2)	17.2 (14.9-19.7)	171.2 (144.8-200.2)	35.1 (6.3-104.1)	17.1 (3.4-32)
South Africa	230.2	560.6 (525-598.2)	15.7 (11-21.5)	88.4 (59.7-121.6)	18.6 (0-39.3)	14.4 (0-30)
South Sudan	78.2	841.5 (725.3-937.6)	17.3 (14.2-21.3)	145.5 (120-171.4)	30.4 (5-88.8)	14.7 (2.9-27)

Tanzania, United Rep.	394	906.3 (846.1-969.6)	17.1 (14.2-19.8)	154 (127.8-182.6)	32 (5.9-95.3)	15.4 (3.1-28.9)
Togo	47.6	950.9 (881-1023.5)	17.8 (14.7-21.2)	169.4 (138-204)	35.1 (6.4-102.3)	16.8 (3.5-31.4)
Uganda	314.7	905.7 (840.4-972)	21.4 (19.2-23.7)	193.7 (169.9-219.9)	40.3 (7.5-117.9)	19.4 (3.9-35.8)
Zambia	115	849.4 (794.3-906.1)	16.9 (14.7-19.2)	143.1 (122.8-165.1)	30.7 (0-59.8)	23.7 (0-45.8)
Zimbabwe	103.9	838.3 (774.5-908.9)	19 (15.8-22.4)	159.3 (130-191.8)	33.2 (6-98.3)	15.9 (3.3-30.4)
**Region of the Americas**						
Belize	1.6	659 (616.2-704.9)	14.1 (6.2-23.4)	95.8 (41.6-158.5)	19.5 (0-47.6)	15 (0-37.3)
Bolivia, Plurinational State	47.8	637 (601.1-676.9)	19.7 (16.6-23.2)	125.8 (105-149.7)	26.9 (0-53.1)	20.7 (0-40.8)
Brazil	602.8	564.1 (520.5-613.6)	16.8 (12.7-21.5)	94.8 (71.2-121.6)	20.1 (0-41.2)	15.5 (0-31.3)
Colombia	148.2	551.3 (519.4-586.1)	14.3 (12.4-16.4)	79.2 (67.5-91.9)	17.1 (0-33.1)	13.2 (0-25.4)
Costa Rica	13.7	538.6 (498.1-581.2)	15.3 (11.3-19.5)	82 (60.4-105.6)	17.4 (0-35.5)	13.5 (0-27.5)
Cuba	26	532.3 (496.1-569.3)	13 (5.8-22.5)	71.8 (31.2-119.2)	14.3 (0-36.3)	11.1 (0-27.8)
Dominica	0.3	582.6 (531-643.4)	16.2 (11.8-20.9)	94.7 (68.2-124)	20.1 (0-40.9)	15.4 (0-31.6)
Dominican Rep.	42.4	695.1 (641-748.4)	13.6 (11.9-15.5)	94.6 (80.9-109.4)	20.3 (0-39.8)	15.6 (0-30.6)
Ecuador	64.5	554.6 (513.5-600.2)	16.9 (13.8-20.7)	93.9 (75.7-115.2)	20.1 (0-39.6)	15.5 (0-30.3)
El Salvador	23.1	582.9 (548.2-619)	14.6 (10.9-18.5)	85.2 (62.4-109.6)	18.1 (0-37.1)	14 (0-28.4)
Grenada	0.4	570.5 (526-624)	17.8 (12.3-23.8)	101.5 (70.8-136.3)	21.4 (0-44.6)	16.5 (0-34)
Guatemala	82	636.9 (600.6-676.8)	14.7 (12.6-17)	94.1 (79.5-110)	20.1 (0-39.4)	15.5 (0-30)
Guyana	3.1	631 (587.8-674)	15.2 (8.9-22.3)	95.1 (55.1-140.8)	19.8 (0-44.4)	15.2 (0-34.2)
Haiti	49.5	905.7 (844.1-972.6)	16.4 (13.5-19.7)	149.1 (121.4-181.2)	30.9 (5.5-93.2)	14.9 (3-28.4)
Honduras	37.9	712.9 (669.6-758.2)	14.9 (12.7-17.3)	106.4 (89.8-124.8)	22.8 (0-44.6)	17.7 (0-34.2)
Jamaica	8	569.4 (522.9-620.2)	14.4 (10.9-18.3)	81.9 (62.1-105.1)	17.6 (0-35.4)	13.6 (0-27.3)
Mexico	467.6	590.6 (551.1-632.4)	13.2 (8.9-18.4)	77.7 (52.2-109.4)	16.2 (0-35.4)	12.5 (0-27)
Nicaragua	23.8	618.6 (563.4-680)	18.7 (14.9-23.9)	115.7 (92-147.6)	24.9 (0-50.7)	19.3 (0-39.1)
Paraguay	26.7	555.5 (521.3-592.2)	26.1 (19.4-33.2)	145 (106.2-186.4)	30.8 (0-62.4)	23.7 (0-48.6)
Peru	122.5	552.7 (519.7-586.9)	24.8 (20.5-29.6)	137.2 (112.3-165)	29.4 (0-57.8)	22.7 (0-44.5)
St. Lucia	0.5	574.3 (524.8-629.4)	15.7 (11-21)	90.5 (63-121.9)	19.1 (0-39.8)	14.8 (0-30.4)
St. Vincent and the Grenadines	0.3	577.1 (526.1-636.3)	15.5 (11.3-20.4)	89.8 (64.9-119.8)	19 (0-38.8)	14.8 (0-29.7)
Suriname	2	584.5 (530-644.1)	15.5 (11.1-20.6)	90.3 (64.3-121)	19.2 (0-39.3)	14.7 (0-30.3)
Venezuela, Bolivarian Rep.	119.1	572.5 (525-623.6)	17.6 (13.5-22.7)	100.8 (76.6-131.1)	21.5 (0-43.3)	16.6 (0-33.1)
**Eastern Mediterranean Region**						
Afghanistan	207.3	932.7 (866.5-1001.6)	24.9 (23.1-26.7)	231.7 (209.3-257)	48.2 (8.5-140.2)	23.2 (4.7-42.8)
Djibouti	4.2	737.8 (673.2-807.3)	19.2 (15.1-24.1)	141.5 (111.7-177.8)	30.5 (0-60.7)	23.6 (0-46.4)
Egypt, Arab Rep.	543.1	615.5 (579.6-654.2)	25.1 (22.8-27.3)	154.2 (137.5-171.4)	33.1 (0-63.9)	25.6 (0-49)
Iran, Islamic Rep.	272.2	551.5 (508.3-598.5)	18.9 (14.9-23.4)	104.7 (81.9-129.9)	22.3 (0-44.8)	17.2 (0-34)
Iraq	232.8	638.1 (579.3-706)	21.5 (17-27.6)	137.2 (108.4-174)	29.3 (0-59.1)	22.7 (0-45.2)
Jordan	49.1	572.3 (536.5-608.7)	24.3 (21.5-27.3)	139.3 (120.3-159)	29.9 (0-57.7)	23.2 (0-44.2)
Lebanon	19.7	550.7 (503.5-602.6)	19.6 (14.4-25)	107.9 (78.3-140.1)	22.9 (0-46.6)	17.8 (0-36.2)
Libyan Arab Rep.	25.5	599.8 (533.5-693.9)	20.2 (15-26)	121.3 (92.7-155.7)	26 (0-53)	20.1 (0-40.5)
Morocco	143.7	594 (540.6-658.9)	21 (16.4-26.7)	124.7 (97.9-159.1)	26.8 (0-54.2)	20.7 (0-41.7)
Pakistan	1012.4	778.8 (730.3-828.1)	19.2 (16.9-21.6)	149.7 (129.5-170.7)	32 (0-62.5)	24.8 (0-48)
Palestinian Terr.	28.9	589.1 (551.6-627.9)	25.4 (20.4-30.3)	149.4 (119.1-179.6)	31.7 (0-62.6)	24.6 (0-48.8)
Somalia	107.5	775.1 (692.4-921.2)	18.2 (14.8-22)	142.2 (117.1-172.2)	30 (5.2-86.9)	14.5 (2.8-27)
Sudan	241.1	801.1 (727.3-876.3)	18.1 (15.1-21.8)	145.5 (120.1-173.8)	31 (0-61.3)	24 (0-47.3)
Tunisia	43.6	541.3 (498.6-586.7)	20.1 (15.3-26.1)	108.7 (82.3-141.8)	23.1 (0-47.2)	17.9 (0-36.4)
Yemen, Rep.	165.4	873.8 (817.5-932.6)	26.5 (24.5-28.5)	231 (209-255.2)	47.9 (8.5-139.8)	23.2 (4.7-42.2)
**European Region**						

Albania	7.5	551.6 (515.9-588.6)	9.8 (1.6-21.3)	54.2 (9.3-121.4)	10.4 (0-33.6)	8 (0-26.2)
Armenia	8	521.4 (487.1-556.7)	21.9 (9.4-37.5)	118.9 (49-204)	23.8 (0-62.9)	18.3 (0-48.5)
Azerbaijan	37.2	598.8 (560.5-640)	36.6 (26.8-46.3)	217.3 (158.6-281.2)	45.5 (0-94.8)	35.5 (0-73.3)
Belarus	23.4	550.1 (508.9-594.3)	17 (11.8-22.7)	93.4 (64.9-127)	19.8 (0-41)	15.2 (0-31.3)
Bosnia and Herzegovina	6.1	534.1 (489.1-586.2)	16.1 (11.4-21.8)	86.2 (60.3-117.8)	18.2 (0-38.2)	14.1 (0-29.4)
Bulgaria	12.5	539.7 (497.7-586.9)	16.5 (10.9-22.7)	88.7 (58.8-122.5)	18.8 (0-39.4)	14.5 (0-30.4)
Georgia	11	551.9 (507.2-604.6)	18.3 (12.4-25.2)	101.1 (67.8-141)	21.4 (0-45.8)	16.4 (0-35.1)
Kazakhstan	80.9	558.2 (515.3-607.1)	22.3 (16.9-29.1)	124.9 (92.7-164.1)	26.7 (0-54)	20.6 (0-41.5)
Kosovo	6	618.2 (574.8-664.7)	9.7 (3.2-21)	59.7 (18.7-127.6)	11.3 (0-35.1)	8.7 (0-27.2)
Kyrgyzstan	31.4	655.1 (609.3-702)	37.7 (29-47.8)	246.5 (184.4-316.9)	52 (0-106.3)	40.3 (0-82.2)
Moldova, Rep.	8.6	577.3 (527.8-629.3)	19.8 (14.4-26.6)	114.3 (82.9-154.3)	24.3 (0-49.9)	18.7 (0-38.6)
Montenegro	1.4	505.8 (466.5-548.7)	7.7 (0-30.8)	39.3 (0-156.5)	8.4 (0-40.2)	6.5 (0-31.1)
North Macedonia	4.8	536.4 (492.1-583.9)	15.4 (10.3-21.8)	82.8 (55.2-117.1)	17.6 (0-37.8)	13.5 (0-29)
Romania	36.9	544.2 (501.8-588.8)	18.1 (13-24.1)	98.4 (70.3-131.7)	20.9 (0-42.8)	16.2 (0-33)
Russian Fed.	393.8	552.7 (507.9-602.8)	17.9 (13.5-22.9)	98.7 (74.1-127.1)	20.9 (0-43.1)	16.2 (0-33.2)
Serbia	18.9	534.2 (491.9-583.5)	16 (10.9-22.3)	85.6 (57.6-120.6)	18.1 (0-38.9)	14 (0-29.7)
Tajikistan	48.1	791 (732-853.5)	33.8 (29.1-39)	267.7 (226.2-312)	55.6 (10.1-165.1)	26.7 (5.5-49.5)
Turkey	273	549.6 (506.6-595.1)	19.3 (14.7-24)	106.1 (79.9-133.7)	22.6 (0-45.2)	17.4 (0-34.7)
Turkmenistan	30.1	660.9 (611.3-711.7)	33.3 (19-47.6)	213.7 (121.7-318.4)	44.1 (0-100)	34 (0-77.3)
Ukraine	90.4	567.2 (521-617.3)	18.2 (13-23.5)	103.1 (73.9-135.4)	21.7 (0-45.1)	16.8 (0-34.5)
Uzbekistan	126.9	626.4 (572.3-688)	26.7 (20.6-34.7)	167.6 (129-219.5)	36.2 (0-72.4)	27.7 (0-56)
**Southeast Asia Region**						
Bangladesh	612.1	801.8 (753.1-855.7)	18.3 (13.4-23.8)	147.4 (104.9-193)	31.1 (0-64.1)	24 (0-49)
Bhutan	2.8	690 (620.6-764.7)	22.1 (16.5-28.5)	152.8 (115-195)	32.6 (0-66.4)	25.3 (0-51)
India	4742.1	814.1 (764.5-866.5)	18.9 (18-20)	154.1 (141.7-167.5)	33.2 (0-64.2)	25.6 (0-48.8)
Indonesia	1010.2	677 (635-721.7)	28.9 (24.5-33.5)	195.6 (163.6-230.8)	41.6 (0-81.9)	32 (0-63.2)
Maldives	1.6	482 (446-518.6)	27.3 (16.4-40)	130.5 (74.7-195.2)	27 (0-60.2)	20.8 (0-46.1)
Myanmar	181.7	846.8 (791.8-903.7)	29.2 (22.7-35.6)	246.2 (190.4-304.9)	52.2 (0-105)	40.3 (0-81)
Nepal	108.2	844.2 (784.1-907.4)	18.4 (13-24.3)	153.3 (106.8-206.3)	31.5 (5.8-96.5)	15.1 (3.1-30.7)
Sri Lanka	63.1	599.8 (550.9-656.4)	24.9 (18.9-32)	148.8 (113-192.3)	31.6 (0-65.7)	24.5 (0-50.2)
Thailand	149.6	621.3 (580.4-664)	27.9 (20.7-35.5)	173.1 (126.9-223.3)	36.6 (0-75.2)	28.2 (0-58.2)
Timor-Leste	8.3	770 (722.8-820.2)	30.8 (24.9-37.8)	237.8 (189.4-294.4)	50.9 (0-101.4)	39.3 (0-77.3)
**Western Pacific Region**						
American Samoa	0.2	596.7 (536.1-668.3)	18.6 (13.5-25)	111.2 (79.3-151.5)	23.7 (0-49.7)	18.2 (0-37.7)
Cambodia	70.2	883.6 (826.6-942.5)	29.5 (23.8-35.6)	260.3 (207.8-318.5)	55.6 (0-112.4)	42.9 (0-85.7)
China, People’s Rep.	3454.2	574.8 (525.8-634.3)	18.8 (14.2-24)	108.6 (81.8-138.8)	23.3 (0-46.4)	17.9 (0-35.8)
Fiji	3.4	595.8 (541-655.5)	20.5 (15.2-26.6)	122.1 (90.5-159.7)	25.9 (0-52.5)	20 (0-40.3)
Kiribati	0.6	678.7 (603.3-763.5)	19.9 (15.1-25.5)	135.1 (101.6-174.5)	29 (0-59.4)	22.3 (0-45.4)
Lao People’s Dem. Rep.	30.6	727.1 (681.7-774.8)	20.5 (16.5-25.4)	149.5 (118.7-185.2)	31.8 (0-63.1)	24.6 (0-48.9)
Malaysia	105.4	553.2 (507.2-602.3)	21.6 (16.6-27.7)	119.5 (91.2-154.4)	25.4 (0-51.9)	19.5 (0-40.4)
Marshall Islands	0.2	610 (551.2-679.2)	15.2 (10.8-20.7)	93.1 (65.7-124.4)	19.9 (0-40.8)	15.3 (0-31.6)
Micronesia, Fed.	2	661.7 (599.6-735.8)	22 (16.7-28.7)	145.8 (111.1-193.2)	31.5 (0-62.8)	24.2 (0-48.3)
Mongolia	15.3	688 (647.2-731.7)	24.8 (19.5-29.8)	169.8 (134.3-207.8)	36.3 (0-72.4)	28 (0-55.5)
Nauru	<0.1	609.3 (554.9-673.1)	22.7 (17.1-30)	138.3 (105-181.9)	29.5 (0-60.8)	22.9 (0-46.3)
Papua New Guinea	41.5	772.9 (703.4-849)	20.2 (16.3-25)	155.7 (124.9-194.5)	33.6 (0-66.5)	26 (0-51.3)
Philippines	464.4	649.6 (609.7-691)	28.5 (22.4-34.5)	184.4 (144.1-228.1)	39.2 (0-79.5)	30.2 (0-60.5)
Samoa	0.9	579 (528.1-635.4)	20.1 (14.9-25.9)	116.5 (86.4-151.5)	24.6 (0-50.3)	19.1 (0-38.5)
Solomon Islands	3.3	736.5 (664.7-813.3)	18.9 (14.8-24)	139.3 (108.7-177)	29.9 (0-59.9)	23 (0-46.4)

Tonga	0.5	608 (554.3-668.9)	18.3 (12.7-24.4)	111.7 (76.4-150)	23.8 (0-48.9)	18.2 (0-37.8)
Tuvalu	<0.1	591.4 (534.1-656)	16.4 (11.8-21.4)	96.7 (70.8-125.2)	20.5 (0-41.4)	16 (0-32.1)
Vanuatu	1.4	713.1 (643.1-789.3)	19.9 (14.9-26.1)	142.1 (105.9-184.7)	30.1 (0-61.9)	23.5 (0-47.7)
Viet Nam	311.4	660.1 (616.9-707.6)	29.1 (20.5-38.5)	193.9 (134.3-258.7)	40.4 (0-85.5)	31.3 (0-65.8)

Incidence rate estimates are presented as cases per 100 children annually in the eligible age range. Estimates are obtained from analyses of DHS/MICS surveys covering 944,173 children across 77 countries, as well as extrapolations based on 405 health, nutrition, and population indicators for all LMICs. Quantiles are obtained via 5,000 independent draws from the distribution of estimates.

Table S19: Lower-bound country-specific estimates of the incidence of diarrhea and antibiotic-treated diarrhea among children ages 0-23 months preventable by rotavirus vaccination.

Country	Population ages 0-23 months	Diarrhea attributable to all causes			Diarrhea attributable to rotavirus	
	Children (in 10,000s)	All cases, per 100 (95% CI)	Proportion treated with antibiotics, % (95% CI)	Antibiotic-treated cases, per 100 (95% CI)	Antibiotic-treated cases, per 100 (95% CI)	Antibiotic-treated cases preventable by vaccine direct effects, per 100 (95% CI)
**African region**						
Algeria	191.3	211.5 (194.1-231)	19.3 (15.6-23.4)	41 (32.3-50.4)	10.1 (1.5-19.1)	6.6 (1.1-12.7)
Angola	215.1	299.7 (241.1-371.2)	15.4 (13.6-17.4)	46.2 (35.8-59.1)	11.2 (1.6-22.2)	7.6 (1.2-14.5)
Benin	72.6	343.5 (279-426)	17.2 (14.4-20.3)	59.2 (44.7-77.4)	10.9 (3.6-21.7)	6.1 (2.1-10.9)
Botswana	10.6	244.3 (218.4-274.3)	18.4 (14.8-22.7)	44.9 (35.3-56.8)	10.9 (1.6-21.2)	7.3 (1.2-13.8)
Burkina Faso	131	377.2 (304.2-468.5)	16.6 (14.8-18.5)	62.5 (49.1-79.9)	11.6 (3.9-22.7)	6.5 (2.2-11.4)
Burundi	77.9	405.8 (324.5-505.3)	16 (14.4-17.6)	64.9 (50.6-82)	11.9 (4.1-23.5)	6.7 (2.3-11.9)
Cabo Verde	2.2	234.1 (210.6-262.1)	18.3 (14.9-22.4)	43 (34-53.4)	10.6 (1.6-20.1)	7.1 (1.2-13.3)
Cameroon	155.1	318 (256.5-393)	19.6 (17.6-21.6)	62.2 (48.9-78.6)	15.1 (2.2-29.9)	10.2 (1.6-19.5)
Cent. African Rep.	30.2	358.6 (335.3-379.3)	17.6 (17-18.1)	63.1 (58.2-67.3)	11.7 (4.1-22.1)	6.5 (2.2-10.9)
Chad	109.6	371.9 (297.9-457.1)	16.4 (14.9-17.9)	60.9 (48.1-76.3)	11.2 (3.8-22.3)	6.3 (2.2-11)
Comoros	4.9	331.4 (267.1-411.4)	17.5 (14.2-20.9)	57.7 (42.7-77)	10.5 (3.5-21.4)	5.9 (2-10.7)
Congo, Rep.	33.5	302.6 (244-372.6)	17.9 (15.8-20)	54 (42.5-68.6)	13.4 (1.9-25.8)	8.8 (1.4-17.1)
Congo, Dem. Rep.	595.6	373.4 (301.3-466.7)	18.4 (16.7-20.1)	68.7 (54.1-87.7)	12.7 (4.4-25.2)	7.1 (2.4-12.5)
Cote d'Ivoire	158.3	329.2 (267.2-405.3)	17.1 (14.9-19.8)	56.4 (43.7-72.9)	13.9 (2-27.5)	9.2 (1.5-17.8)
Equatorial Guinea	7.4	266.7 (233.5-303.3)	19 (15.8-23.3)	50.7 (40.2-64.4)	12.3 (1.8-24.3)	8.3 (1.4-15.9)
Eritrea	29.7	328.2 (295.3-361)	19.7 (16.4-23.2)	64.5 (51.9-78.9)	11.9 (4-23.4)	6.7 (2.2-11.4)
Eswatini	7.2	271.1 (219-334.3)	16.2 (12.2-20.2)	43.6 (31.3-59.6)	10.6 (1.4-21.5)	7 (1.1-14)
Ethiopia	619.3	368.7 (293.2-453.8)	15.6 (13.2-18.1)	57.4 (43.4-74.4)	10.5 (3.5-21.3)	5.9 (2-10.6)
Gabon	11.2	222.4 (180.3-274.6)	15.3 (12.6-17.9)	33.9 (25.6-44.5)	8.2 (1.2-16.3)	5.5 (0.9-10.9)
Gambia, The	14.8	349.4 (281.2-433.8)	19.2 (16.9-21.6)	66.9 (52.5-86.2)	12.4 (4.2-24.4)	6.9 (2.4-12.4)
Ghana	166.5	301.9 (243.5-373.1)	20.9 (17.4-24.8)	63.1 (47.7-82.8)	15.4 (2.1-31)	10.3 (1.6-20.1)
Guinea	80.9	361.9 (292.2-445.5)	17.1 (14.3-20)	61.6 (47.1-80.2)	11.3 (3.8-22.7)	6.4 (2.2-11.5)
Guinea-Bissau	11.9	324.2 (260.4-401.4)	16.5 (13.3-19.8)	53.3 (39.2-71.5)	9.8 (3.3-19.9)	5.5 (1.9-10)
Kenya	280.4	314.1 (253.4-389.2)	16.3 (14.8-17.9)	51.2 (40.5-64.6)	12.7 (1.8-24.3)	8.3 (1.3-16.4)
Lesotho	11.7	297.4 (240.8-370.2)	16.3 (12.8-20.7)	48.7 (34.7-66.7)	11.8 (1.7-24)	7.9 (1.2-15.8)
Liberia	29	357.5 (289.4-442.9)	18.3 (16.1-20.6)	65.3 (51.4-83.1)	12 (4.2-23.9)	6.7 (2.3-11.9)
Madagascar	152.4	338.7 (307.3-370.4)	19.5 (16.9-22.7)	66.3 (55.8-78.8)	12.2 (4.2-23.4)	6.9 (2.4-11.7)
Malawi	115.7	388.2 (311.6-480.5)	16.9 (15.5-18.5)	65.6 (51.7-82.5)	12.1 (4.1-23.4)	6.8 (2.4-12)
Mali	135.9	366 (294.5-450.8)	17.1 (14.3-20.1)	62.2 (47.1-81.7)	11.4 (3.9-22.6)	6.4 (2.2-11.6)
Mauritania	26.7	316.3 (255.5-392.3)	16.7 (14.6-18.9)	52.8 (41.4-67.6)	13 (1.9-25.2)	8.6 (1.3-16.6)
Mauritius	2.7	213 (195.4-234.2)	18.2 (13.6-23.4)	38.8 (28.5-50.9)	9.4 (1.3-19)	6.3 (1-12.4)
Mozambique	202.3	389.6 (312.4-487)	17.8 (14.7-21.6)	69.5 (51.5-93.4)	12.8 (4.3-26)	7.2 (2.4-13)
Namibia	14.2	275.2 (221.4-340)	15.6 (13-18.6)	43.1 (32.1-56.9)	10.6 (1.5-20.8)	7 (1.1-13.7)
Niger	173.9	383.3 (308.8-472.3)	16.4 (14.4-18.5)	62.6 (48.9-79.4)	11.5 (3.9-23.1)	6.4 (2.2-11.3)
Nigeria	1306.7	325.9 (263.6-401.6)	21.1 (19.2-23.3)	68.7 (54.5-86.6)	17 (2.5-33)	11.2 (1.8-21.4)
Rwanda	69.8	337.9 (273-417.4)	16.5 (13.9-19.3)	56.1 (42.5-72.7)	10.3 (3.5-20.7)	5.7 (1.9-10.4)
Sao Tome and Principe	1.3	297.4 (240.3-370.5)	20.3 (14.6-26.8)	61.1 (41.7-86.3)	14.9 (2-31)	9.9 (1.5-20.1)
Senegal	103.5	327.6 (265.4-403.5)	17.2 (15.4-18.9)	56.3 (44.4-70.9)	10.4 (3.5-20.3)	5.8 (2-10.2)
Sierra Leone	46.2	377.7 (304.4-469)	17.2 (15.3-19.3)	65.2 (50.8-83.3)	12 (4.1-23.5)	6.7 (2.3-12)
South Africa	230.2	212.7 (172.4-264.1)	15.7 (11.5-20.4)	33.5 (23.2-46.9)	8.1 (1.1-17)	5.4 (0.9-11.1)
South Sudan	78.2	312.9 (273.7-347)	17.2 (14.3-19.9)	53.6 (42.7-64.4)	9.7 (3.4-19.2)	5.5 (1.8-9.5)

Tanzania, United Rep.	394	343.5 (278.5-426)	17.1 (14.8-19.4)	58.2 (45.5-75.1)	10.8 (3.6-21.3)	6 (2-10.7)
Togo	47.6	361.6 (291.3-445)	17.8 (15.2-20.5)	64.2 (49-83)	11.7 (4-23.4)	6.6 (2.3-11.8)
Uganda	314.7	342.8 (277.4-425.5)	21.4 (19.5-23.4)	73.6 (58-92.4)	13.5 (4.6-26.9)	7.6 (2.5-13.4)
Zambia	115	321.8 (260.4-397.8)	16.9 (15-18.8)	54.2 (42.7-68.8)	13.3 (1.9-26.4)	8.9 (1.4-17)
Zimbabwe	103.9	318.5 (256.2-398.6)	19 (16.3-21.9)	60.5 (46.3-78.6)	11.1 (3.8-22.3)	6.2 (2.1-11.1)
**Region of the Americas**						
Belize	1.6	250.4 (202.6-309.5)	14.1 (7.8-21.9)	36.4 (18.8-59.1)	8.5 (1.1-20.3)	5.7 (0.8-13.1)
Bolivia, Plurinational State	47.8	242.9 (195.9-300.3)	19.7 (17.1-22.7)	48 (37-61.6)	11.6 (1.6-22.9)	7.7 (1.2-15.3)
Brazil	602.8	214.2 (196.9-235.4)	15.9 (12.2-19.9)	33.9 (25.8-43.4)	8.3 (1.2-16.3)	5.5 (0.9-10.7)
Colombia	148.2	209.6 (169.7-259)	14.3 (12.7-16.1)	30 (23.6-38.3)	7.4 (1.1-14.5)	4.9 (0.8-9.5)
Costa Rica	13.7	203.3 (187.3-220.7)	16 (12.2-20)	32.6 (24.6-41.3)	8 (1.2-15.6)	5.3 (0.9-10.3)
Cuba	26	203.4 (163.4-250.8)	13 (7.1-21.1)	27.3 (14-45.2)	6.3 (0.8-15.1)	4.2 (0.6-10)
Dominica	0.3	216.2 (197.3-236.6)	17 (13-21.3)	36.7 (27.6-47.2)	8.9 (1.4-17.5)	5.9 (1-11.4)
Dominican Rep.	42.4	263.8 (212.7-326.5)	13.6 (12.2-15.2)	36 (28.3-45.5)	8.8 (1.3-17.5)	5.9 (0.9-11.3)
Ecuador	64.5	208.9 (192.8-228.2)	16.8 (13.8-20.5)	35.3 (28.3-43.8)	8.7 (1.2-16.8)	5.8 (0.9-10.9)
El Salvador	23.1	221.5 (178-273.8)	14.6 (11.2-17.9)	32.2 (23.5-43.4)	7.9 (1.1-16)	5.3 (0.8-10.3)
Grenada	0.4	214.6 (197.6-234.7)	18.6 (14.6-23.1)	40.1 (30.8-50.1)	9.8 (1.5-19)	6.5 (1.1-12.5)
Guatemala	82	241.8 (196.8-302.7)	14.7 (12.9-16.7)	35.7 (27.9-46)	8.7 (1.3-17.2)	5.8 (0.9-11.5)
Guyana	3.1	240 (195.1-296.9)	15.2 (9.8-21.4)	36 (22.3-53.9)	8.6 (1.1-18.7)	5.7 (0.9-12.2)
Haiti	49.5	344.2 (278.6-426.4)	16.4 (13.9-19.2)	56.7 (43.3-73.9)	10.4 (3.5-20.9)	5.9 (2-10.5)
Honduras	37.9	271 (218.5-333.6)	14.9 (13.1-16.9)	40.4 (31.3-51.7)	9.9 (1.4-19.3)	6.6 (1.1-12.8)
Jamaica	8	213.1 (195.5-233)	14.9 (11.4-18.9)	31.8 (24.2-41.2)	7.8 (1.1-15.2)	5.2 (0.8-9.9)
Mexico	467.6	224.1 (181.4-278.2)	13.2 (9.5-17.5)	29.6 (20.1-42.2)	7.2 (1-14.9)	4.8 (0.8-9.8)
Nicaragua	23.8	227 (206.6-253.7)	17.5 (14.5-21)	39.8 (32.3-49.5)	9.8 (1.5-19)	6.6 (1.1-12.3)
Paraguay	26.7	211.6 (170.1-259.8)	26.1 (20.4-31.8)	54.7 (39.8-74.1)	13.4 (1.9-27.4)	8.9 (1.4-17.6)
Peru	122.5	210.9 (170.7-260.1)	24.8 (21.1-28.8)	52.4 (40-67.9)	12.8 (1.8-25.3)	8.6 (1.4-16.6)
St. Lucia	0.5	217.3 (198.7-239)	16.6 (12.9-21.1)	36.2 (27.5-46.7)	8.9 (1.3-17.5)	5.9 (1-11.4)
St. Vincent and the Grenadines	0.3	213.6 (195.9-235.6)	16.5 (12.6-21)	35.4 (26.7-46.1)	8.6 (1.3-17.2)	5.7 (0.9-11.1)
Suriname	2	218.3 (199.1-240.6)	16.5 (12.5-21)	36.1 (27.1-46.5)	8.8 (1.3-17.5)	5.9 (0.9-11.3)
Venezuela, Bolivarian Rep.	119.1	217.6 (198.9-238.8)	16.1 (13.1-19.8)	35.3 (28.2-44.2)	8.7 (1.3-16.8)	5.8 (0.9-10.9)
**Eastern Mediterranean Region**						
Afghanistan	207.3	354.3 (285.5-438)	24.9 (23.4-26.4)	88 (70.8-109.9)	16.3 (5.5-31.7)	9 (3.1-16.2)
Djibouti	4.2	281.9 (249.5-315.9)	19 (15.8-22.8)	53.5 (43.2-65.9)	13.1 (2-25.6)	8.7 (1.4-16.7)
Egypt, Arab Rep.	543.1	233.7 (189.4-289)	25.1 (23.2-26.9)	58.5 (47.2-73.3)	14.4 (2.1-28.1)	9.6 (1.5-18.3)
Iran, Islamic Rep.	272.2	211.1 (194.3-230.1)	19.4 (15.5-23.5)	41.1 (32.1-50.6)	10.1 (1.5-19.4)	6.6 (1.1-12.7)
Iraq	232.8	243.7 (218.3-275.7)	22.1 (18.1-27.3)	54.3 (42.9-68.8)	13.4 (2-25.9)	8.9 (1.4-16.9)
Jordan	49.1	217.1 (175.3-268.1)	24.3 (21.9-26.8)	52.8 (41.6-66.8)	13 (1.9-25.1)	8.7 (1.4-16.7)
Lebanon	19.7	211.8 (193.2-234.6)	19.3 (15.2-23.9)	41.1 (31.5-52.2)	9.9 (1.5-19.8)	6.7 (1.1-12.8)
Libyan Arab Rep.	25.5	218.3 (198-245.4)	21.2 (16.5-26.5)	46.4 (34.7-60.4)	11.4 (1.7-22.2)	7.6 (1.2-14.8)
Morocco	143.7	221.9 (203.4-245.4)	22 (17.6-27)	49 (38.3-61)	12 (1.8-23.2)	8 (1.3-15.1)
Pakistan	1012.4	294.7 (239.4-366.4)	19.2 (17.2-21.2)	56.6 (45-71.7)	13.9 (2-27)	9.2 (1.4-18.1)
Palestinian Terr.	28.9	223.3 (180.4-276.9)	25.4 (21.4-29.4)	56.4 (42.9-74.4)	13.9 (2-27.7)	9.2 (1.5-18.1)
Somalia	107.5	311.1 (273.2-348.8)	17.4 (14.9-20.4)	54.1 (44.3-65.2)	10 (3.4-19.4)	5.6 (1.9-9.5)
Sudan	241.1	245.5 (228-264.8)	22 (20.2-24.8)	54.2 (48.1-62.5)	13.5 (2-25.3)	8.9 (1.4-16.8)
Tunisia	43.6	208.1 (191.1-225.9)	21 (16.3-25.5)	43.7 (33.5-54.1)	10.9 (1.6-20.8)	7.1 (1.1-13.5)
Yemen, Rep.	165.4	331.2 (268.3-412.8)	26.5 (24.8-28.2)	87.5 (70.2-110.7)	16.2 (5.3-31.5)	9 (3.1-16)
**European Region**						

Albania	7.5	209.8 (168.7-259.2)	9.8 (3.3-19.7)	20.4 (6.5-42.7)	4.6 (0.3-13.2)	3.1 (0.3-9)
Armenia	8	198 (159.3-245.3)	21.9 (12.5-37.5)	45.1 (22.1-75.8)	10.6 (1.2-25.4)	7 (1-16.6)
Azerbaijan	37.2	227.4 (184.7-280.3)	36.6 (28.5-44.7)	82.1 (59.9-110.4)	20.1 (2.8-40)	13.3 (2.1-26.9)
Belarus	23.4	208 (190-227.7)	18.8 (14.1-23.8)	39.1 (28.9-50.2)	9.6 (1.4-18.8)	6.3 (1-12.2)
Bosnia and Herzegovina	6.1	204.6 (187.2-223.4)	15.7 (11.7-20.1)	32.1 (23.7-41.6)	7.8 (1.1-15.3)	5.2 (0.8-10.2)
Bulgaria	12.5	205.4 (187.9-223.4)	17.5 (11.8-23.3)	36 (24.2-48.5)	8.6 (1.2-17.6)	5.7 (0.9-11.6)
Georgia	11	209.3 (191.9-229.2)	18.5 (13.4-23.7)	38.8 (28-50.6)	9.5 (1.4-18.7)	6.3 (1-12.4)
Kazakhstan	80.9	211.9 (194.2-232.8)	22.4 (17.6-27.8)	47.7 (36.5-60.4)	11.7 (1.7-22.9)	7.7 (1.3-14.9)
Kosovo	6	235.5 (189.9-291)	9.7 (3.2-17.7)	22.5 (7.5-44.9)	5.1 (0.4-14.5)	3.3 (0.4-9.5)
Kyrgyzstan	31.4	248.9 (202.1-308.1)	37.7 (30.4-46.4)	93.5 (69.2-125.4)	22.8 (3.1-45.8)	15.2 (2.4-30.2)
Moldova, Rep.	8.6	215.5 (196.4-237.8)	20.3 (15.8-25.6)	43.9 (33.4-56.3)	10.8 (1.6-21.1)	7.1 (1.2-13.8)
Montenegro	1.4	192.3 (154.8-238.8)	7.7 (0-23.1)	15.3 (0-52.8)	3.5 (0-15.4)	2.4 (0-10)
North Macedonia	4.8	205.4 (188.6-224.5)	17 (12.3-22.3)	35 (25-46.3)	8.4 (1.2-17.2)	5.6 (0.9-11)
Romania	36.9	206 (187.9-225)	18.1 (13-23.5)	37.3 (26.3-49.5)	9 (1.3-18.1)	6 (0.9-11.9)
Russian Fed.	393.8	211.2 (198.4-224.1)	20.7 (17.2-26.4)	43.8 (36.1-56.2)	10.8 (1.5-20.8)	7.2 (1.1-13.9)
Serbia	18.9	205.5 (188-224.5)	15.8 (11.2-20.9)	32.5 (23-43.5)	7.8 (1.1-15.9)	5.2 (0.8-10.4)
Tajikistan	48.1	299.6 (241.8-373.9)	33.8 (29.7-38.2)	101.6 (79.1-131.1)	18.6 (6.5-36.9)	10.5 (3.6-18.5)
Turkey	273	208.4 (191.3-227.7)	20.7 (16.4-25.2)	43.2 (33.6-53.5)	10.5 (1.6-20.2)	7.1 (1.1-13.3)
Turkmenistan	30.1	249.8 (202.5-307.7)	33.3 (21.4-45.2)	81.2 (49-119.8)	19.3 (2.6-42.3)	12.9 (1.9-27.3)
Ukraine	90.4	212 (193.5-234.5)	17.9 (13.5-22.8)	38 (28.3-49.3)	9.2 (1.4-18.5)	6.2 (1-12)
Uzbekistan	126.9	229.7 (209.2-256.1)	26.2 (21.4-31.9)	60.2 (48.3-75.7)	14.8 (2.2-28.4)	9.9 (1.6-18.7)
	7.5	209.8 (168.7-259.2)	9.8 (3.3-19.7)	20.4 (6.5-42.7)	4.6 (0.3-13.2)	3.1 (0.3-9)
**Southeast Asia Region**						
Bangladesh	612.1	303.2 (245.7-375.7)	18.3 (14.4-23.3)	55.9 (39.9-76.5)	13.5 (1.8-27.4)	9 (1.5-18.2)
Bhutan	2.8	244.5 (218.1-275.6)	22.8 (18.5-28.3)	56.1 (43.8-71.3)	13.8 (2-27)	9.2 (1.5-17.4)
India	4742.1	310.5 (251.4-381.6)	18.9 (18.1-19.8)	58.7 (47.6-72.7)	14.5 (2.1-27.9)	9.7 (1.6-18.2)
Indonesia	1010.2	257.5 (208.7-317.4)	28.9 (25.1-32.8)	74.3 (58.1-95)	18.1 (2.5-36)	12.1 (2-23.6)
Maldives	1.6	183.7 (147.9-226.6)	27.3 (18.2-38.2)	49.4 (30-73.5)	11.8 (1.5-25.7)	7.9 (1.3-16.6)
Myanmar	181.7	322.7 (260.7-398.3)	29.2 (23.7-34.6)	93.3 (70.2-125.1)	22.9 (3.3-46)	15.3 (2.4-30.3)
Nepal	108.2	320.1 (257.8-397)	18.4 (13.5-23.2)	58.1 (40.5-80.5)	10.6 (3.5-22.1)	5.9 (1.9-10.9)
Sri Lanka	63.1	224.5 (205.4-249.4)	23.4 (18.8-28.3)	52.9 (41.7-65.2)	12.8 (1.9-24.7)	8.6 (1.4-16)
Thailand	149.6	236.1 (190.8-292.4)	27.9 (21.8-34.2)	65.6 (47.4-89)	15.9 (2.2-32.5)	10.6 (1.7-21.1)
Timor-Leste	8.3	292.8 (236.6-360.9)	30.8 (25.9-36.8)	90.4 (68.5-118.6)	22.1 (3.2-44)	14.7 (2.4-28.9)
**Western Pacific Region**						
American Samoa	0.2	211.3 (192.6-235.5)	18.4 (13-24.4)	39 (26.6-53.7)	9.4 (1.3-19.6)	6.3 (1-12.9)
Cambodia	70.2	336.2 (273-415.8)	29.5 (24.8-34.8)	99 (75.2-130.1)	24.3 (3.4-48.3)	16.1 (2.5-31.4)
China, People's Rep.	3454.2	217.4 (197.3-241)	19.6 (15.6-24.1)	42.7 (33.2-53.7)	10.4 (1.5-20.4)	6.9 (1.1-13.3)
Fiji	3.4	217.9 (198.5-242.1)	21.6 (16.9-26.2)	46.9 (36.1-58.3)	11.4 (1.7-22.1)	7.6 (1.2-14.7)
Kiribati	0.6	251.3 (222.1-283.3)	19.9 (15.6-24.4)	49.8 (38.3-63)	12.3 (1.7-23.6)	8.1 (1.4-15.5)
Lao People's Dem. Rep.	30.6	275.3 (222.4-341.2)	20.5 (17.1-24.4)	56.6 (42.8-74.6)	13.9 (2-27.6)	9.2 (1.5-18.1)
Malaysia	105.4	209.1 (192.6-227.1)	22.6 (18.1-28.4)	47.4 (36.9-60.6)	11.6 (1.7-22.8)	7.7 (1.2-15)
Marshall Islands	0.2	233 (206.5-263)	15.7 (11.8-20.1)	36.7 (27.1-47.6)	8.9 (1.2-17.2)	5.9 (1-11.5)
Micronesia, Fed.	2	253 (224.4-287)	18.9 (14.8-23.1)	47.7 (36.4-60.8)	11.6 (1.6-22.7)	7.7 (1.3-14.8)
Mongolia	15.3	261.7 (211.7-323.9)	24.8 (20.6-29.1)	64.5 (48.5-84.9)	15.8 (2.2-31.4)	10.5 (1.7-20.5)
Nauru	<0.1	234.6 (209.6-265.8)	20.1 (15.9-25.4)	47.5 (36.4-62)	11.7 (1.7-22.8)	7.7 (1.3-14.9)
Papua New Guinea	41.5	285.7 (252.6-319.5)	20.5 (17.2-24.7)	58.7 (47.2-72.6)	14.4 (2.1-27.9)	9.6 (1.5-18.1)
Philippines	464.4	247.4 (197.9-305.1)	28.5 (23.3-33.6)	70 (52.2-92)	17.1 (2.4-33.9)	11.4 (1.8-22.3)
Samoa	0.9	224.4 (200.7-252.6)	19.7 (14.8-24.4)	44 (32.6-56.5)	10.7 (1.5-21.3)	7.1 (1.2-13.8)
Solomon Islands	3.3	273.9 (241.3-310.2)	20.3 (15.7-25.2)	55.4 (42.3-70.6)	13.6 (2-27)	9 (1.4-17.3)

Tonga	0.5	225.4 (203.3-252.1)	18.6 (13.6-23.8)	42 (30.5-54.6)	10.1 (1.5-20.4)	6.7 (1.1-13.1)
Tuvalu	<0.1	221.2 (200-248.5)	16.2 (12.3-20.6)	36.1 (26.7-46.9)	8.8 (1.2-17.4)	5.9 (1-11.4)
Vanuatu	1.4	247.5 (220.5-279.6)	19.7 (15.4-24.6)	48.9 (37.5-62.3)	12 (1.8-23.8)	7.9 (1.3-15.3)
Viet Nam	311.4	249.5 (201.1-309.9)	29.1 (22.2-36.8)	72.9 (51.1-102.1)	17.6 (2.4-36.5)	11.8 (1.8-24)

Incidence rate estimates are presented as cases per 100 children annually in the eligible age range. Estimates are obtained from analyses of DHS/MICS surveys covering 944,173 children across 77 countries, as well as extrapolations based on 405 health, nutrition, and population indicators for all LMICs. Quantiles are obtained via 5,000 independent draws from the distribution of estimates.

Table S20: Total antibiotic consumption attributable to vaccine-serotype *Streptococcus pneumoniae* among children ages 24-59 months in LMICs, absent use of PCV10/13.

Vaccine efficacy input estimate	Measure	Setting	Primary est. (95% CI)	Lower bound est. (95% CI)	Upper bound est. (95% CI)
Efficacy against vaccine-serotype IPD (81.2%)					
	Incidence per 100 children annually				
		Low income countries	18.6 (3.2, 40.4)	10.3 (1.8, 22.3)	33.8 (5.9, 72.7)
		Lower-middle income countries	17.4 (3.0, 37.8)	9.6 (1.6, 20.9)	31.6 (5.4, 68.8)
		Upper middle income countries	12.0 (2.1, 26.5)	6.6 (1.2, 14.7)	21.8 (3.8, 48.0)
		All low/middle income countries	15.9 (2.8, 34.7)	8.8 (1.5, 19.1)	29.0 (5.0, 63.0)
	Total annual cases (in millions)				
		Low income countries	11.6 (2.0, 25.2)	6.4 (1.1, 13.9)	21.1 (3.7, 45.5)
		Lower-middle income countries	32.1 (5.5, 69.8)	17.7 (3.0, 38.6)	58.2 (10.1, 126.9)
		Upper middle income countries	13.2 (2.3, 29.1)	7.3 (1.3, 16.1)	23.9 (4.2, 52.7)
		All low/middle income countries	56.9 (9.9, 123.8)	31.4 (5.4, 68.2)	103.3 (17.9, 224.9)
Efficacy against vaccine-serotype AOM (55.6%)					
	Incidence per 100 children annually				
		Low income countries	26.8 (4.7, 58.3)	14.8 (2.6, 32.1)	48.8 (8.6, 105.5)
		Lower-middle income countries	25.2 (4.3, 54.4)	13.9 (2.4, 30.2)	45.7 (7.9, 99.5)
		Upper middle income countries	17.3 (3.1, 38.0)	9.6 (1.7, 21.0)	31.5 (5.6, 69.1)
		All low/middle income countries	23.1 (4.0, 49.9)	12.7 (2.2, 27.6)	41.9 (7.2, 91.2)
	Total annual cases (in millions)				
		Low income countries	16.8 (2.9, 36.4)	9.3 (1.6, 20.1)	30.5 (5.4, 66.0)
		Lower-middle income countries	46.4 (8.0, 100.3)	25.7 (4.4, 55.7)	84.2 (14.5, 183.5)
		Upper middle income countries	19.0 (3.4, 41.7)	10.5 (1.8, 23.1)	34.6 (6.1, 75.9)
		All low/middle income countries	82.3 (14.4, 178.1)	45.4 (7.8, 98.6)	149.4 (25.9, 325.3)

Estimates are pooled from country-specific incidence estimates for all and antibiotic-treated diarrhea, as listed in **Table S10** (primary estimates), **Table S13** (upper bound estimates), and **Table S14** (lower bound estimates). Quantiles are obtained via 5,000 independent draws from the distribution of estimates.

Table S21: Total antibiotic consumption attributable to vaccine-serotype *Streptococcus pneumoniae* among children ages 0-59 months in LMICs, absent use of PCV10/13.

Vaccine efficacy input estimate	Measure	Setting	Primary est. (95% CI)	Lower bound est. (95% CI)	Upper bound est. (95% CI)
Efficacy against vaccine-serotype IPD (81.2%)					
	Incidence per 100 children annually				
		Low income countries	9.7 (–1.4, 22.4)	5.4 (–0.8, 12.7)	17.2 (–2.5, 40.0)
		Lower-middle income countries	8.9 (–1.2, 20.6)	5.0 (–0.7, 11.6)	15.8 (–2.2, 36.6)
		Upper-middle income countries	6.0 (–0.9, 14.2)	3.4 (–0.5, 8.0)	10.8 (–1.6, 25.5)
		All low/middle income countries	8.2 (–1.1, 19.0)	4.6 (–0.7, 10.7)	14.5 (–2.1, 33.7)
	Total annual cases (in millions)				
		Low income countries	10.4 (–1.5, 24.0)	5.8 (–0.8, 13.6)	18.4 (–2.6, 42.8)
		Lower-middle income countries	27.4 (–3.9, 63.7)	15.4 (–2.2, 36.0)	48.8 (–6.9, 113.3)
		Upper-middle income countries	11.1 (–1.6, 26.2)	6.3 (–0.9, 14.8)	19.8 (–2.9, 46.9)
		All low/middle income countries	49.0 (–6.8, 114.0)	27.5 (–3.9, 64.0)	87.0 (–12.4, 202.6)
Efficacy against vaccine-serotype AOM (55.6%)					
	Incidence per 100 children annually				
		Low income countries	14.0 (–2.0, 32.3)	7.9 (–1.1, 18.2)	24.9 (–3.6, 57.6)
		Lower-middle income countries	12.8 (–1.8, 29.5)	7.2 (–1.0, 16.6)	22.8 (–3.3, 52.5)
		Upper-middle income countries	8.7 (–1.3, 20.3)	4.9 (–0.7, 11.4)	15.6 (–2.3, 36.2)
		All low/middle income countries	11.8 (–1.7, 27.1)	6.6 (–1.0, 15.3)	21.0 (–3.0, 48.4)
	Total annual cases (in millions)				
		Low income countries	15.0 (–2.1, 34.5)	8.4 (–1.2, 19.5)	26.6 (–3.8, 61.5)
		Lower-middle income countries	39.7 (–5.5, 91.3)	22.3 (–3.2, 51.4)	70.6 (–10.1, 162.2)
		Upper-middle income countries	16.1 (–2.4, 37.4)	9.1 (–1.3, 21.0)	28.7 (–4.2, 66.6)
		All low/middle income countries	70.7 (–10.0, 162.9)	39.8 (–5.7, 92.1)	126.0 (–18.1, 290.6)

Estimates are pooled from country-specific incidence estimates for all and antibiotic-treated diarrhea, as listed in **Table S11** (primary estimates), **Table S15** (upper bound estimates), and **Table S16** (lower bound estimates). Quantiles are obtained via 5,000 independent draws from the distribution of estimates.

Table S22: Total antibiotic consumption attributable to rotavirus among children ages 0-23 months in LMICs, absent use of rotavirus vaccines.

Measure	Setting	Primary estimate (95% CI)	Lower bound estimate (95% CI)	Upper bound estimate (95% CI)
Incidence per 100 children annually				
	Low income countries	22.3 (3.9, 65.0)	13.8 (2.4, 39.9)	36.0 (6.2, 101.5)
	Lower-middle income countries	21.5 (0.0, 41.9)	13.4 (0.0, 25.8)	35.0 (0.0, 66.8)
	Upper middle income countries	14.2 (0.0, 28.1)	8.8 (0.0, 17.3)	23.1 (0.0, 45.2)
	All low/middle income countries	19.7 (4.6, 36.8)	12.2 (2.9, 22.5)	31.8 (7.4, 58.5)
Total annual cases (in millions)				
	Low income countries	9.9 (1.7, 28.8)	6.1 (1.0, 17.7)	16.0 (2.8, 45.0)
	Lower-middle income countries	26.7 (0.0, 52.2)	16.7 (0.0, 32.1)	43.7 (0.0, 83.2)
	Upper middle income countries	10.6 (0.0, 20.9)	6.6 (0.0, 12.9)	17.2 (0.0, 33.7)
	All low/middle income countries	47.9 (11.1, 89.5)	29.7 (7.0, 54.7)	77.5 (18.1, 142.5)

Estimates are pooled from country-specific incidence estimates for all and antibiotic-treated diarrhea, as listed in **Table S12** (primary estimates), **Table S17** (upper bound estimates), and **Table S18** (lower bound estimates). Quantiles are obtained via 5,000 independent draws from the distribution of estimates.

Table S23: Country-level PCV10/13 and rotavirus vaccine coverage estimates.

Country	PCV10/13		Rotavirus vaccines	
	Implementation in national immunization program	≥3 dose coverage estimate, %	Implementation in national immunization program	Complete coverage estimate (2 or 3 doses, depending on product), %
Afghanistan	Yes	84	Yes	60
Albania	Yes	99	No	0
Algeria	No	0	No	0
American Samoa	Yes	77.5	Yes	71.8
Angola	Yes	82	Yes	75
Armenia	Yes	92	Yes	93
Azerbaijan	Yes	95	No	0
Bangladesh	Yes	99	No	0
Belarus	Yes	98	No	0
Belize	No	0	No	0
Benin	Yes	75	No	0
Bhutan	No	0	No	0
Bolivia	Yes	83	Yes	87
Bosnia and Herzegovina	No	0	No	0
Botswana	Yes	77	Yes	74
Brazil	Yes	84	Yes	80
Bulgaria	Yes	89	Yes	31
Burkina Faso	Yes	91	Yes	91
Burundi	Yes	90	Yes	92
Cabo Verde	No	0	No	0
Cambodia	Yes	93	No	0
Cameroon	Yes	79	Yes	78
Central African Republic	Yes	73	No	0
Chad	No	0	No	0
China	No	0	No	0
Colombia	Yes	94	Yes	90
Comoros	No	0	No	0
Congo (Republic of)	Yes	83	Yes	83
Congo (Democratic Republic of)	Yes	94	No	0
Costa Rica	Yes	96	No	0
Cote d'Ivoire	Yes	94	Yes	72
Cuba	No	0	No	0
Djibouti	Yes	84	Yes	87
Dominica	No	0	No	0
Dominican Republic	Yes	70	Yes	82
Ecuador	Yes	85	Yes	85
Egypt	No	0	No	0
El Salvador	Yes	75	Yes	82
Equatorial Guinea	No	0	No	0
Eritrea	Yes	95	Yes	96
Eswatini	Yes	88	Yes	90
Ethiopia	Yes	94	Yes	93
Fiji	Yes	86	Yes	86
Gabon	No	0	No	0
Gambia	Yes	93	Yes	93
Georgia	Yes	81	Yes	79
Ghana	Yes	96	Yes	94
Grenada	No	0	No	0
Guatemala	Yes	85	Yes	87
Guinea	No	0	No	0
Guinea Bissau	Yes	82	Yes	85
Guyana	Yes	91	Yes	91
Haiti	Yes	1	Yes	73
Honduras	Yes	91	Yes	92
India	Yes	44	Yes	73
Indonesia	Yes	8	No	0
Iran (Islamic Republic of)	No	0	No	0
Iraq	Yes	32	Yes	60
Jamaica	Yes	44	No	0
Jordan	No	0	Yes	93
Kazakhstan	Yes	95	No	0
Kenya	Yes	81	Yes	78

Kiribati	Yes	94	Yes	97
Kosovo	Yes	92.5	Yes	76.3
Kyrgyzstan	Yes	92	No	0
Lao People's Democratic Republic	Yes	83	No	0
Lebanon	Yes	82	No	0
Lesotho	Yes	83	Yes	70
Liberia	Yes	97	Yes	87
Libyan Arab Jamahiriya	Yes	96	Yes	97
Madagascar	Yes	91	Yes	89
Malawi	Yes	92	Yes	90
Malaysia	No	0	No	0
Maldives	No	0	No	0
Mali	Yes	63	Yes	60
Marshall Islands	Yes	67	Yes	42
Mauritania	Yes	88	Yes	89
Mauritius	Yes	96	Yes	95
Mexico	Yes	88	Yes	77
Micronesia (Federated States of)	Yes	67	Yes	52
Moldova (Republic of)	Yes	94	Yes	75
Mongolia	Yes	98	No	0
Montenegro	No	0	No	0
Morocco	Yes	99	Yes	99
Mozambique	Yes	90	Yes	90
Myanmar	Yes	91	No	0
Namibia	Yes	81	Yes	92
Nauru	No	0	No	0
Nepal	Yes	82	No	0
Nicaragua	Yes	99	Yes	99
Niger	Yes	79	Yes	89
Nigeria	Yes	58	No	0
North Macedonia	No	0	No	0
Pakistan	Yes	72	Yes	58
Palestinian Territory	Yes	80.1	Yes	80.8
Papua New Guinea	Yes	43	No	0
Paraguay	Yes	79	Yes	79
Peru	Yes	82	Yes	85
Philippines	Yes	60	Yes	82
Romania	No	0	No	0
Russian Federation	Yes	82	No	0
Rwanda	Yes	97	Yes	98
Samoa	No	0	No	0
Sao Tome and Principe	Yes	95	Yes	95
Senegal	Yes	81	Yes	63
Serbia	No	0	No	0
Sierra Leone	Yes	94	Yes	96
Solomon Islands	Yes	84	No	0
Somalia	No	0	No	0
South Africa	Yes	83	Yes	80
South Sudan	No	0	No	0
Sri Lanka	No	0	No	0
St. Lucia	No	0	No	0
St. Vincent and the Grenadines	No	0	No	0
Sudan	Yes	93	Yes	94
Suriname	No	0	No	0
Tajikistan	No	0	Yes	96
Tanzania	Yes	98	Yes	98
Thailand (United Republic of)	No	0	Yes	73
Timor-Leste	No	0	No	0
Togo	Yes	88	Yes	89
Tonga	No	0	No	0
Tunisia	No	0	No	0
Turkey	Yes	97	No	0
Turkmenistan	No	0	No	0
Tuvalu	No	0	No	0
Uganda	Yes	64	Yes	36
Ukraine	No	0	No	0

Uzbekistan	Yes	96	Yes	84
Vanuatu	No	0	No	0
Venezuela (Bolivarian Republic of)	Yes	0	Yes	18
Vietnam	No	0	No	0
Yemen	Yes	79	Yes	79
Zambia	Yes	90	Yes	91
Zimbabwe	Yes	89	Yes	90

Estimates of current vaccine coverage (as of 2018) provide the basis for estimating direct effects of existing PCV10/13 and rotavirus vaccination programs (**Figure 4**).

Table S24: Total antibiotic consumption preventable by 10- and 13-valent pneumococcal conjugate vaccines among children ages 24-59 months in LMICs.

Estimated effect	Setting	Primary est. (95% CI)	Lower bound est. (95% CI)	Upper bound est. (95% CI)
Cases (in millions) prevented annually by PCV10/13 direct effects under existing coverage				
	Low income countries	7.3 (1.3, 15.9)	4.0 (0.7, 8.8)	13.2 (2.3, 28.8)
	Lower-middle income countries	13.3 (2.3, 29.1)	7.3 (1.3, 16.1)	24.2 (4.2, 52.6)
	Upper middle income countries	3.2 (0.6, 6.9)	1.7 (0.3, 3.8)	5.8 (1.0, 12.7)
	All low/middle income countries	23.8 (4.2, 52.0)	13.1 (2.2, 28.7)	43.1 (7.4, 94.1)
Additional annual cases (in millions) preventable by PCV10/13 direct effects through expansion to universal coverage				
	Low income countries	2.0 (0.4, 4.4)	1.1 (0.2, 2.4)	3.6 (0.6, 7.9)
	Lower-middle income countries	12.3 (2.2, 26.8)	6.8 (1.2, 14.8)	22.3 (3.8, 48.6)
	Upper middle income countries	7.3 (1.3, 16.1)	4.1 (0.7, 8.9)	13.3 (2.4, 29.3)
	All low/middle income countries	21.7 (3.8, 47.5)	12.0 (2.1, 26.2)	39.4 (6.8, 86.0)
Total annual cases (in millions) preventable by PCV10/13 direct effects under a scenario of universal coverage, relative to no vaccination				
	Low income countries	9.3 (1.7, 20.3)	5.1 (0.9, 11.2)	16.8 (2.9, 36.6)
	Lower-middle income countries	25.6 (4.5, 55.9)	14.1 (2.4, 30.9)	46.5 (8.0, 101.3)
	Upper middle income countries	10.5 (1.9, 22.9)	5.8 (1.0, 12.8)	19.2 (3.4, 41.8)
	All low/middle income countries	45.5 (8.1, 99.3)	25.0 (4.3, 54.9)	82.5 (14.1, 180.5)

Estimates are aggregated from country-specific estimates presented in **Table S11** (primary estimate), **Tables S14** (upper bound estimate), and **Table S15** (lower bound estimate). Analyses use direct effect estimates presented in **Table S3**. Country-specific coverage of vaccines is presented in **Table S23**. Primary estimates listed in this table are plotted in **Figure 4** in the Main Text. Quantiles are obtained via 5,000 independent draws from the distribution of estimates.

Table S25: Total antibiotic consumption preventable by 10- and 13-valent pneumococcal conjugate vaccines among children ages 0-59 months in LMICs.

Estimated effect	Setting	Primary est. (95% CI)	Lower bound est. (95% CI)	Upper bound est. (95% CI)
Cases (in millions) prevented annually by PCV10/13 direct effects under existing coverage				
	Low income countries	6.5 (–0.9, 14.6)	3.7 (–0.5, 8.3)	11.6 (–1.7, 26.3)
	Lower-middle income countries	11.4 (–1.7, 25.7)	6.4 (–0.9, 14.5)	20.3 (–3.0, 45.9)
	Upper middle income countries	2.8 (–0.4, 6.2)	1.5 (–0.2, 3.5)	4.9 (–0.7, 11.0)
	All low/middle income countries	20.7 (–3.0, 46.3)	11.6 (–1.7, 26.3)	36.7 (–5.4, 83.3)
Additional annual cases (in millions) preventable by PCV10/13 direct effects through expansion to universal coverage				
	Low income countries	1.8 (–0.3, 4.0)	1.0 (–0.1, 2.3)	3.2 (–0.5, 7.2)
	Lower-middle income countries	10.5 (–1.5, 23.5)	5.9 (–0.9, 13.3)	18.6 (–2.7, 42.4)
	Upper middle income countries	6.2 (–0.9, 14.0)	3.5 (–0.5, 7.9)	11.0 (–1.6, 25.1)
	All low/middle income countries	18.4 (–2.7, 41.4)	10.3 (–1.6, 23.4)	32.8 (–4.9, 74.1)
Total annual cases (in millions) preventable by PCV10/13 direct effects under a scenario of universal coverage, relative to no vaccination				
	Low income countries	8.3 (–1.2, 18.6)	4.7 (–0.7, 10.6)	14.7 (–2.2, 33.5)
	Lower-middle income countries	21.9 (–3.2, 49.1)	12.3 (–1.8, 27.8)	38.9 (–5.7, 88.1)
	Upper middle income countries	9.0 (–1.3, 20.2)	5.0 (–0.7, 11.4)	15.9 (–2.4, 36.3)
	All low/middle income countries	39.1 (–5.7, 88.0)	21.9 (–3.3, 49.7)	69.4 (–10.3, 157.2)

Estimates are aggregated from country-specific estimates presented in **Table S12** (primary estimate), **Tables S16** (upper bound estimate), and **Table S17** (lower bound estimate). Analyses use direct effect estimates presented in **Table S3**. Country-specific coverage of vaccines is presented in **Table S23**. Quantiles are obtained via 5,000 independent draws from the distribution of estimates.

Table S26: Total antibiotic consumption preventable by rotavirus vaccination among children ages 0-23 months in LMICs.

**Estimated effect**	**Setting**	**Primary est. (95% CI)**	**Lower bound est. (95% CI)**	**Upper bound est. (95% CI)**
Cases (in millions) prevented annually by rotavirus vaccine direct effects under existing coverage				
	Low income countries	3.8 (1.3, 6.4)	2.3 (0.8, 3.9)	6.1 (2.0, 10.1)
	Lower-middle income countries	8.2 (2.1, 14.4)	5.1 (1.3, 9.0)	13.3 (3.3, 23.2)
	Upper middle income countries	1.7 (0.4, 3.0)	1.0 (0.3, 1.9)	2.7 (0.6, 4.8)
	All low/middle income countries	13.6 (3.6, 23.7)	8.5 (2.4, 14.6)	22.2 (5.9, 37.9)
Additional annual cases (in millions) preventable by rotavirus vaccine direct effects through expansion to universal coverage				
	Low income countries	2.5 (0.9, 4.1)	1.5 (0.6, 2.5)	4.0 (1.5, 6.5)
	Lower-middle income countries	9.9 (2.4, 17.5)	6.2 (1.5, 10.7)	16.1 (3.8, 27.9)
	Upper middle income countries	6.0 (0.5, 11.3)	3.7 (0.3, 7.0)	9.7 (0.9, 18.2)
	All low/middle income countries	18.3 (4.2, 32.6)	11.4 (2.6, 20.0)	29.9 (6.9, 51.9)
Total annual cases (in millions) preventable by rotavirus vaccine direct effects under a scenario of universal coverage, relative to no vaccination				
	Low income countries	6.2 (2.1, 10.4)	3.8 (1.4, 6.4)	10.1 (3.6, 16.5)
	Lower-middle income countries	18.1 (4.4, 31.8)	11.3 (2.8, 19.6)	29.5 (7.2, 51.0)
	Upper middle income countries	7.7 (0.9, 14.2)	4.7 (0.6, 8.9)	12.5 (1.6, 22.9)
	All low/middle income countries	32.0 (7.9, 56.2)	20.0 (4.9, 34.5)	51.9 (12.8, 89.9)

Estimates are aggregated from country-specific estimates presented in **Table S13** (primary estimate), **Tables S18** (upper bound estimate), and **Table S19** (lower bound estimate). Analyses use direct effect estimates presented in **Table S5**. Country-specific coverage of vaccines is presented in **Table S23**. Primary estimates listed in this table are plotted in **Figure 4** in the Main Text. Quantiles are obtained via 5,000 independent draws from the distribution of estimates.

Table S27: Information extracted for estimation of ARI and diarrhea incidence.

Risk factor	Measurement/values	Missing data (%)
Child age	Continuous; 0 to 59 months	0.5%
PCV10/13 doses	Discrete; 0 to ≥3 doses	20.4%
Pentavalent vaccine doses	Discrete; 0 to ≥3 doses	41.7%
Inactivated polio vaccine doses	Discrete; 0 to ≥3 doses	42.8%
Rotavirus vaccine doses	Discrete; 0 to ≥3 doses	22.9%
Survey day	Discrete; 0 to 31	0%
Survey month	Discrete; calendar months	0%
Year	Discrete; 2006 to 2018	0%
Sex	Male, Female	0.4%
Within-country wealth index	Continuous	19.1%
Household access to electricity	Binary; household has electricity	5.2%
Household cooking fuel[1]	Factor; household use of electricity, LPG, natural gas, biogas, kerosene, coal/lignite, charcoal, wood, straw/shrubs/grass, animal dung, or agricultural crop residue as a primary fuel source	8.1%
Household access to sanitation[2]	Factor; household use of flush/pour flush toilet to piped sewer system or septic tank, ventilated pit latrine or pit latrine with or without slab, composting toilet, bucket, hanging toilet or latrine, or open defecation	5.8%
Household access to improved water[3]	Factor; household use of piped water (into dwelling, compound, neighbor's plot, or public tap/standpipe), tube well or borehole, protected or unprotected dug well, protected or unprotected spring water, collected rain water, tanker truck, cart with small tank/drum, or surface water	1.1%
Urban setting	Binary; urban or rural setting	0.6%
Maternal education	Factor; Highest education level completed by mother as preschool, primary, secondary, or higher education	2.2%
Nutritional status	Continuous; weight-for-height Z score	30.2%
Non-DHS variables		
GBD region [144]	Factor; Andean Latin America, Caribbean, Central Asia, Central Europe, Central Latin America, Central Sub-Saharan Africa, Eastern Europe, Eastern Sub-Saharan Africa, North Africa/Middle East, South Asia, Southeast Asia, Southern Sub-Saharan Africa, Tropical Latin America, Western Sub-Saharan Africa	0%
Country GDP per capita	Continuous; $237.44 to $16028.25	0%
Antibiotic access [15]	Continuous; defined daily doses per capital of retail sales for all antibiotics	67.9%

1. Household cooking fuels were collapsed to solid and non-solid fuel sources for analysis.
2. Household sanitation sources were collapsed to improved sanitation, unimproved sanitation, and open defecation for analysis.
3. Household water access sources were collapsed to improved and unimproved water sources for analysis.
4. GBD region was collapsed to Central and Eastern Europe region, Middle East and Central Asia region, Central Latin America and Caribbean region, South America region (including Andean Latin America and Tropical Latin America GBD regions), South and Southeast Asia region, and Sub-Saharan Africa region for analysis.

Table S28: Fitted model parameters for ARI incidence among children ages 24-59 months.

Exposure	Unit measurement	Hazard ratio
Weight for height		
	Per Z-score increase	0.990 (0.970, 1.011)
Age		
	Per year increase	0.874 (0.855, 0.896)
Urban residence		
	Relative to rural residence	0.931 (0.896, 0.968)
Exposure to solid cooking fuels		
	Relative to no exposure to solid cooking fuels	1.222 (1.157, 1.283)
Maternal education[1]		
	Relative to mother with no education complete	
	Mother with primary education complete	1.233 (1.166, 1.307)
	Mother with secondary education complete	1.117 (1.055, 1.184)
	Mother with post-secondary education complete	1.000 (0.928, 1.080)
Year		
	Per year since 2006	0.984 (0.973, 0.995)
Country GDP per capita		
	Per log increase in USD/capita	0.880 (0.849, 0.914)

Parameters relate to the model described in Eq. 4; selection of variables listed in **Table S27** is described in the **Methods**. Estimates are obtained from analyses of DHS/MICS surveys covering 566,508 children across 77 countries. Quantiles are obtained via 5,000 independent draws from the distribution of estimates.

1. Because under-reporting by mothers without any education completed was suspected to contribute to the pattern in estimated effects, we assumed risk among children whose mothers had completed no education was equivalent to the estimated risk among mothers with primary education complete.

Table S29: Fitted model parameters for ARI incidence among children ages 0-59 months.

Exposure	Unit measurement	Hazard ratio
Weight for height		
	Per Z-score increase	0.983 (0.969, 0.997)
Age		
	Per year increase	0.877 (0.868, 0.887)
Urban residence		
	Relative to rural residence	0.916 (0.889, 0.945)
Exposure to solid cooking fuels		
	Relative to no exposure to solid cooking fuels	1.212 (1.166, 1.259)
Maternal education[1]		
	Relative to mother with no education complete	
	Mother with primary education complete	1.282 (1.127, 1.340)
	Mother with secondary education complete	1.129 (1.108 1.180)
	Mother with post-secondary education complete	1.006 (0.948, 1.065)
Year		
	Per year since 2006	0.983 (0.975, 0.991)
Country GDP per capita		
	Per log increase in USD/capita	0.882 (0.857, 0.908)

Parameters relate to the model described in Eq. 4; selection of variables listed in **Table S27** is described in the **Methods**. Estimates are obtained from analyses of DHS/MICS surveys covering 944,173 children across 77 countries. Quantiles are obtained via 5,000 independent draws from the distribution of estimates.

1. Because under-reporting by mothers without any education completed was suspected to contribute to the pattern in estimated effects, we assumed risk among children whose mothers had completed no education was equivalent to the estimated risk among mothers with primary education complete.

Table S30: Fitted model parameters for diarrhea incidence among children ages 0-23 months.

Exposure	Unit measurement	Hazard ratio
Weight for height		
	Per Z-score increase	0.909 (0.901, 0.917)
Age		
	Per year increase	1.073 (1.041, 1.106)
Urban residence		
	Relative to rural residence	0.999 (0.989, 1.000)
Access to electricity in household		
	Relative to no electricity in household	0.924 (0.896, 0.953)
Maternal education[1,2]		
	Relative to mother with no education complete	
	Mother with primary education complete	1.166 (1.131, 1.120)
	Mother with secondary education complete	1.073 (1.041, 1.106)
	Mother with post-secondary education complete	0.942 (0.901, 0.986)
Wealth index (within country)		
	Per 1 standard-deviation change	
	Mother with no education complete	1.016 (0.995, 1.039)
	Mother with primary education complete	0.991 (0.970, 1.013)
	Mother with secondary education complete	0.979 (0.956, 0.999)
	Mother with post-secondary education complete	0.941 (0.953, 0.977)
Year		
	Per year since 2006	0.985 (0.978, 0.991)

Parameters relate to the model described in Eq. 4; selection of variables listed in **Table S27** is described in the **Materials and Methods**. We illustrate parameter estimates describing the conditional effect of water and sanitation covariates across countries of differing wealth status (in terms of GDP per capita) in **Extended data figure 3**. Estimates are obtained from analyses of DHS/MICS surveys covering 377,665 children across 77 countries. Quantiles are obtained via 5,000 independent draws from the distribution of estimates

1. Values are presented for the effect of maternal education assuming a wealth index equal to 0 (due to the interaction between these parameters).
2. Because under-reporting by mothers without any education completed was suspected to contribute to the pattern in estimated effects, we assumed risk among children whose mothers had completed no education was equivalent to the estimated risk among mothers with primary education complete.

Table S31: Fitted model parameters for antibiotic treatment of ARI among children ages 24-59 months.

Exposure	Unit measurement	Risk ratio
Wealth index (within country)		
	Per 1 standard-deviation change	1.069 (1.043, 1.095)
Age		
	Per year increase	0.973 (0.951, 0.994)
Urban residence		
	Relative to rural residence	0.995 (0.954, 1.038)
Concomitant fever		
	Relative to no fever	1.096 (1.051, 1.139)
Maternal education		
	Relative to mother with no education complete	
	Mother with primary education complete	1.125 (1.060, 1.191)
	Mother with secondary education complete	1.187 (1.125, 1.252)
	Mother with post-secondary education complete	1.078 (1.004, 1.160)
Country antibiotic sales		
	Per log increase in defined daily doses per 1000 population	
	Central Asia and Middle East region	1.040 (1.010, 1.071)
	Central Latin America and Caribbean region	0.923 (0.889, 0.959)
	Eastern Europe region	0.854 (0.251, 3.276)
	South America region	0.989 (0.969, 1.008)
	South and Southeast Asia region	1.083 (1.027, 1.144)
	Sub-Saharan Africa region	0.981 (0.969, 0.993)
Year		
	Per year since 2006	0.964 (0.952, 0.975)

Parameters relate to the model described in Eq. 5; selection of variables listed in **Table S27** is described in the **Methods**. We illustrate region-specific associations with antibiotic treatment probability in **Extended data figure 4**. Estimates are obtained from analyses of DHS/MICS surveys covering 566,508 children across 77 countries. Quantiles are obtained via 5,000 independent draws from the distribution of estimates.

Table S32: Fitted model parameters for antibiotic treatment of ARI among children ages 0-59 months.

Exposure	Unit measurement	Risk ratio
Wealth index (within country)		
	Per 1 standard-deviation change	1.051 (1.033, 1.070)
Age		
	Per year increase	0.987 (0.978, 0.997)
Urban residence		
	Relative to rural residence	1.016 (0.984, 1.049)
Concomitant fever		
	Relative to no fever	1.100 (1.067, 1.132)
Maternal education		
	Relative to mother with no education complete	
	Mother with primary education complete	1.115 (1.068, 1.162)
	Mother with secondary education complete	1.157 (1.113, 1.203)
	Mother with post-secondary education complete	1.061 (1.004, 1.121)
Country antibiotic sales		
	Per log increase in defined daily doses per 1000 population	
	Central Asia and Middle East region	1.032 (1.011, 1.054)
	Central Latin America and Caribbean region	0.922 (0.895, 0.950)
	Eastern Europe region	0.918 (0.360, 2.098)
	South America region	0.990 (0.975, 1.004)
	South and Southeast Asia region	1.078 (1.037, 1.121)
	Sub-Saharan Africa region	0.985 (0.977, 0.994)
Year		
	Per year since 2006	0.963 (0.955, 0.971)

Parameters relate to the model described in Eq. 5; selection of variables listed in **Table S27** is described in the **Methods**. Associations of region and country GDP per capita with probability of antibiotic treatment are similar to those plotted in **Extended data figure 4** for ARI cases at ages 24-59 months. Estimates are obtained from analyses of DHS/MICS surveys covering 944,173 children across 77 countries. Quantiles are obtained via 5,000 independent draws from the distribution of estimates.

Table S33: Fitted model parameters for antibiotic treatment of diarrhea among children ages 0-23 months.

Exposure	Unit measurement	Risk ratio
Weight for height		
	Per Z-score increase	0.936 (0.921, 0.950)
Age		
	Per year increase	1.097 (1.063, 1.132)
Wealth index (within country)		
	Per 1 standard-deviation change	1.017 (0.995, 1.039)
Country GDP per capita[1]		
	Per log increase	
	Rural settings	0.849 (0.816, 0.883)
	Urban settings	0.987 (0.954, 1.021)
Urban residence[2]		
	Relative to rural residence	1.041 (1.002, 1.081)
Access to electricity in household		
	Relative to no electricity in household	1.126 (1.075, 1.180)
Maternal education		
	Relative to mother with no education complete	
	Mother with primary education complete	1.121 (1.072, 1.173)
	Mother with secondary education complete	1.216 (1.163, 1.272)
	Mother with post-secondary education complete	1.098 (1.027, 1.176)
Country antibiotic sales		
	Per log increase in defined daily doses per 1000 population	
	Central Asia and Middle East region	0.961 (0.947, 0.976)
	Central Latin America and Caribbean region	1.017 (0.956, 1.086)
	Eastern Europe region	1.050 (0.190, 6.724)
	South America region	1.035 (1.014, 1.056)
	South and Southeast Asia region	1.097 (1.053, 1.145)
	Sub-Saharan Africa region	0.972 (0.964, 0.980)
Year		
	Per year since 2006	0.912 (0.904, 0.920)

Parameters relate to the model described in Eq. 5; selection of variables listed in **Table S27** is described in the **Methods**. Estimates are obtained from analyses of DHS/MICS surveys covering 377,665 children across 77 countries. Quantiles are obtained via 5,000 independent draws from the distribution of estimates

1. The separate interaction of GDP per capita and region as determinants of the likelihood of antibiotic treatment is plotted in **Extended data figure 5**.
2. The effect of urban residence for a child residing in a country with a per-capita GDP of $2426.07 (population-weighted LMIC mean), accounting for the interaction of urban residence with country GDP per capita.
