# Supplementary material for: Childhood vaccines and antibiotic use in low- and middle-income countries
Source: Nature. 2020 Apr 29;581(7806):94–9. doi: 10.1038/s41586-020-2238-4 (PMC7332418; doi:10.1038/s41586-020-2238-4)
Supplement: Supplementary file 2 — Reporting Summary [file 41586_2020_2238_MOESM2_ESM.pdf]

nature research

Corresponding author(s): Lewnard

Last updated by author(s): Feb 24, 2020

Reporting Summary

Nature Research wishes to improve the reproducibility of the work that we publish. This form provides structure for consistency and transparency in reporting. For further information on Nature Research policies, see Authors & Referees and the Editorial Policy Checklist.

Statistics

For all statistical analyses, confirm that the following items are present in the figure legend, table legend, main text, or Methods section.

n/a	Confirmed	
☐	☒	The exact sample size (n) for each experimental group/condition, given as a discrete number and unit of measurement
☐	☒	A statement on whether measurements were taken from distinct samples or whether the same sample was measured repeatedly
☐	☒	The statistical test(s) used AND whether they are one- or two-sided<br>*Only common tests should be described solely by name; describe more complex techniques in the Methods section.*
☐	☒	A description of all covariates tested
☐	☒	A description of any assumptions or corrections, such as tests of normality and adjustment for multiple comparisons
☐	☒	A full description of the statistical parameters including central tendency (e.g. means) or other basic estimates (e.g. regression coefficient) AND variation (e.g. standard deviation) or associated estimates of uncertainty (e.g. confidence intervals)
☐	☒	For null hypothesis testing, the test statistic (e.g. F, t, r) with confidence intervals, effect sizes, degrees of freedom and P value noted<br>*Give P values as exact values whenever suitable.*
☒	☐	For Bayesian analysis, information on the choice of priors and Markov chain Monte Carlo settings
☐	☒	For hierarchical and complex designs, identification of the appropriate level for tests and full reporting of outcomes
☐	☒	Estimates of effect sizes (e.g. Cohen's d, Pearson's r), indicating how they were calculated

Our web collection on statistics for biologists contains articles on many of the points above.

Software and code

Policy information about availability of computer code

Data collection	Our analysis used publicly-available data sources from the MICS and DHS surveys. MICS data are publicly available at http://mics.unicef.org/. DHS data are available upon request at https://dhsprogram.com/Data/.
Data analysis	Analyses were conducted in R (version 3.5.1). We used the "metafor" package (for meta-analysis); the "survival" package (for fitting of conditional logistic regression models); the "Amelia II" package (for multiple imputation); and the "caret" and "gbm" packages (for cross-validation analyses). Analysis code is available at https://github.com/joelewnard/global-abx/

For manuscripts utilizing custom algorithms or software that are central to the research but not yet described in published literature, software must be made available to editors/reviewers. We strongly encourage code deposition in a community repository (e.g. GitHub). See the Nature Research guidelines for submitting code & software for further information.

Data

Policy information about availability of data

All manuscripts must include a data availability statement. This statement should provide the following information, where applicable:

- Accession codes, unique identifiers, or web links for publicly available datasets
- A list of figures that have associated raw data
- A description of any restrictions on data availability

Analysis code is available from http://github.com/joelewnard to replicate all parts of the analysis and generation of figures. MICS data are publicly available at http://mics.unicef.org/. DHS data are available upon request at https://dhsprogram.com/Data/. Files posted to the author’s github repository above include reduced versions of the DHS and MICS datasets necessary to replicate analyses.

Field-specific reporting

Please select the one below that is the best fit for your research. If you are not sure, read the appropriate sections before making your selection.

☐ Life sciences ☒ Behavioural & social sciences ☐ Ecological, evolutionary & environmental sciences

For a reference copy of the document with all sections, see nature.com/documents/nr-reporting-summary-flat.pdf

Behavioural & social sciences study design

All studies must disclose on these points even when the disclosure is negative.

Study description	We conducted a case-control study of the association of pneumococcal conjugate vaccine and rotavirus vaccine receipt with mother-reported acute respiratory infection and diarrhea and related care-seeking among children under five years of age in low- and middle-income countries, and estimated the vaccine-preventable burden of antibiotic use associated with these infections.
Research sample	Children under five years of age residing in low- and middle-income countries, whose mothers responded to Demographic Health Survey (DHS) and Multiple Indicator Cluster Survey (MICS) rounds. Children under five years old are of interest as the population at greatest risk of antibiotic-treated ARI and diarrhea episodes which may be preventable by pneumococcal conjugate vaccines and rotavirus vaccines. The DHS and MICS surveys address household composition and risk factors, health outcomes, and heatlhcare utilization among household occupants. The design of the DHS and MICS sampling strategies provides a nationally representative selection of households, enabling valid estimation of incidence rates and probabilities of care seeking or antibiotic receipt, given illness; data were available from 77 countries, in total, for our analyses. The DHS/MICS studies provide the largest sample of individual-level observations on ARI and diarrhea outcomes, vaccination status, and risk factors among children in LMICs.
Sampling strategy	DHS and MICS use a probability-based geographic sampling scheme to select clusters within countries (or provinces of countries), and households within clusters; as our analyses were of a secondary nature, we could not dictate statistical power by direct enrollment of study subjects, and used data available from all children. For the case-control study, data were available on 65,815 children, including 5342 ARI cases and 9944 diarrhea cases. Based on the number of children available for analysis, we expected sufficient statistical power to estimate vaccine direct effects against all-cause disease endpoints under a 1:3 matched case-control design. Our analysis of risk factor data to estimate incidence rates included all 944,173 children for whom data were available from DHS/MICS surveys beginning in 2006; in this large sample, we likewise expected sufficient statistical power would be available for estimation of the effects of individual risk factors on ARI and diarrhea outcomes.
Data collection	We conducted secondary analyses of the DHS and MICS datasets; data collection for the original surveys is conducted using standardized, pre-piloted questionnaires and is described elsewhere (see https://dhsprogram.com/ and http://mics.unicef.org/).
Timing	We used data collected in surveys administered from January 1, 2006 to December 31, 2018. For the case-control study, we used data collected from January 1, 2015 onward; prior surveys did not collect card-confirmed rotavirus and/or pneumococcal vaccination status.
Data exclusions	For the case-control study, we excluded children from whom the following data were unavailable: outcome (ARI or diarrhea and related care-seeking), exposure (vaccination status), and covariates used in the matching procedure (including age, visit timing, country, urbanicity, household wealth quintile, and pentavalent vaccine doses received). For other analyses (development of the burden model), we limited data to the most recent DHS/MICS survey round undertaken in each country and did not exclude children with missing data; missing variables were multiply imputed using the Amelia II package in R.
Non-participation	Our analyses included data from all mothers who consented to participate in DHS/MICS survey rounds; participation in the original surveys was voluntary, and respondents were free to decline to participate or to refuse to answer any question. We conducted multiple imputation to analyze answers to questions for which answers were not available (because the respondent refused to answer or was unsure of the answer).
Randomization	Our study was not randomized. For the case-control analyses, we controlled for the following variables via exact matching: age, visit timing, country, urbanicity, household wealth quintile, and pentavalent vaccine doses received. In addition, we validated estimates by testing for vaccine effects against negative control conditions using the same analysis framework (PCV effects against diarrhea outcomes, and rotavirus vaccine effects against ARI outcomes). Our burden model aimed to estimate incidence rates as a function of multiple individual risk factors, for which causal inference was not the focus of analyses.

Reporting for specific materials, systems and methods

We require information from authors about some types of materials, experimental systems and methods used in many studies. Here, indicate whether each material, system or method listed is relevant to your study. If you are not sure if a list item applies to your research, read the appropriate section before selecting a response.

Materials & experimental systems

n/a	Involved in the study
☒	☐ Antibodies
☒	☐ Eukaryotic cell lines
☒	☐ Palaeontology
☒	☐ Animals and other organisms
☒	☐ Human research participants
☒	☐ Clinical data

Methods

n/a	Involved in the study
☒	☐ ChIP-seq
☒	☐ Flow cytometry
☒	☐ MRI-based neuroimaging
